# Perinatal Obesity Induces Hepatic Growth Restriction with Increased DNA Damage Response, Senescence, and Dysregulated Igf-1-Akt-Foxo1 Signaling in Male Offspring of Obese Mice

**DOI:** 10.3390/ijms23105609

**Published:** 2022-05-17

**Authors:** Philipp Kasper, Jaco Selle, Christina Vohlen, Rebecca Wilke, Celien Kuiper-Makris, Oleksiy Klymenko, Inga Bae-Gartz, Charlotte Schömig, Alexander Quaas, Björn Schumacher, Münevver Demir, Martin Bürger, Sonja Lang, Anna Martin, Hans-Michael Steffen, Tobias Goeser, Jörg Dötsch, Miguel A. Alejandre Alcazar

**Affiliations:** 1 Clinic for Gastroenterology and Hepatology, University Hospital Cologne, Faculty of Medicine, University of Cologne, 50937 Cologne, Germany; philipp.kasper@uk-koeln.de (P.K.); martin.buerger@uk-koeln.de (M.B.); sonja.lang@uk-koeln.de (S.L.); anna.martin@uk-koeln.de (A.M.); hans-michael.steffen@uk-koeln.de (H.-M.S.); tobias.goeser@uk-koeln.de (T.G.); 2 Department of Pediatric and Adolescent Medicine, Translational Experimental Pediatrics—Experimental Pulmonology, University Hospital Cologne, Faculty of Medicine, University of Cologne, 50937 Cologne, Germany; jaco.selle@uk-koeln.de (J.S.); christina.vohlen@uk-koeln.de (C.V.); rebecca.wilke@uk-koeln.de (R.W.); centina.kuiper-makris@uk-koeln.de (C.K.-M.); 3 Department of Pediatric and Adolescent Medicine, University Hospital Cologne, Faculty of Medicine, University of Cologne, 50937 Cologne, Germany; inga.bae-gartz@uk-koeln.de (I.B.-G.); charlotte.schoemig@uk-koeln.de (C.S.); joerg.doetsch@uk-koeln.de (J.D.); 4 The German Centre for Lung Research (DZL), Marburg Lung Centre (UGMLC), Institute for Lung Health, Justus-Liebig University Giessen, 35392 Giessen, Germany; oleksiyklymenko@yahoo.com; 5 Department of Pathology, University Hospital Cologne, Faculty of Medicine, University of Cologne, 50937 Cologne, Germany; alexander.quaas@uk-koeln.de; 6 Cologne Excellence Cluster for Stress Responses in Ageing-Associated Diseases (CECAD), University Hospital Cologne, Faculty of Medicine, University of Cologne, 50937 Cologne, Germany; bjoern.schumacher@uni-koeln.de; 7 University Hospital Cologne, Faculty of Medicine, Institute for Genome Stability in Aging and Disease, University of Cologne, 50937 Cologne, Germany; 8 Department of Hepatology and Gastroenterology, Campus Virchow Clinic, Charité Campus Mitte, Charité University Medicine Berlin, 10117 Berlin, Germany; muenevver.demir@charite.de; 9 Center for Molecular Medicine Cologne (CMMC), University Hospital Cologne, Faculty of Medicine, University of Cologne, 50937 Cologne, Germany

**Keywords:** metabolic programming, liver, maternal obesity, cellular senescence, aging

## Abstract

Maternal obesity predisposes for hepato-metabolic disorders early in life. However, the underlying mechanisms causing early onset dysfunction of the liver and metabolism remain elusive. Since obesity is associated with subacute chronic inflammation and accelerated aging, we test the hypothesis whether maternal obesity induces aging processes in the developing liver and determines thereby hepatic growth. To this end, maternal obesity was induced with high-fat diet (HFD) in C57BL/6N mice and male offspring were studied at the end of the lactation [postnatal day 21 (P21)]. Maternal obesity induced an obese body composition with metabolic inflammation and a marked hepatic growth restriction in the male offspring at P21. Proteomic and molecular analyses revealed three interrelated mechanisms that might account for the impaired hepatic growth pattern, indicating prematurely induced aging processes: (1) Increased DNA damage response (γH2AX), (2) significant upregulation of hepatocellular senescence markers (Cdnk1a, Cdkn2a); and (3) inhibition of hepatic insulin/insulin-like growth factor (IGF)-1-AKT-p38-FoxO1 signaling with an insufficient proliferative growth response. In conclusion, our murine data demonstrate that perinatal obesity induces an obese body composition in male offspring with hepatic growth restriction through a possible premature hepatic aging that is indicated by a pathologic sequence of inflammation, DNA damage, senescence, and signs of a possibly insufficient regenerative capacity.

## 1. Introduction

The prevalence of obesity is rapidly rising worldwide and has become a major public health burden over the past decades [1]. The obesity-associated pathologies are diverse and comprise metabolic and cardiovascular as well as hepatic diseases [2]. It is alarming that the continuously increasing obesity rate can be observed across all age groups of the population, including children, adolescents, and women of reproductive age [3]. Recent studies from Europe and the United States estimate that approximately one-third of all women of childbearing age are overweight or obese prior to becoming pregnant [4,5]. Unfortunately, this trend has been aggravated by the ongoing coronavirus disease 2019 (COVID-19) pandemic, during which a further incremental rise in maternal pre-pregnancy body mass index (BMI) is currently observed [6]. Since maternal obesity during pregnancy has adverse short and long-term health consequences for both mother and child, this condition represents a concerning public health issue.

Accumulating clinical and experimental evidence demonstrates that maternal obesity causes an altered genetic, hormonal, and metabolic micro-environment for the developing fetus and thereby adversely influences fetal growth and organ development [5,7]. For example, infants of obese mothers are at an increased risk for poor neonatal outcomes including congenital abnormalities or fetal growth alterations, and are more susceptible to cardio-metabolic disorders such as obesity, insulin resistance, hypertension, or dyslipidemia later in life [8,9]. Experimental studies support the notion that intrauterine and early life metabolic environment interferes with developmental processes and thereby determines organ structure, function, and susceptibility for diseases in the offspring. This condition has been coined as the concept of developmental origins of health and diseases (DOHaD), also known as the fetal programming hypothesis [10].

While previous studies have demonstrated that maternal obesity has a detrimental impact on the development of the cardiovascular, pulmonary, and renal system with subsequent risk for chronic diseases later in life, the impact of maternal obesity on liver development and liver growth remains elusive [11,12]. The liver acts as the central regulator of energy homeostasis by orchestrating numerous metabolic processes, e.g., glycolysis, gluconeogenesis, and fatty acid metabolism. Disruption of the liver development and maturation as a result of metabolic imbalance early in life, may have long-lasting adverse metabolic consequences rendering the liver more susceptible to chronic diseases in later life [13]. White adipose tissue (WAT) is a highly active endocrine organ that is significantly involved in metabolism and health through the storage of fatty acids and the production of cytokines and hormones [14]. Chronic subacute inflammation as a feature of obesity is a central pathomechanism of obesity-associated diseases. Inflammation induces insulin resistance and promotes the development of cardiovascular as well as chronic hepatic diseases, such as non-alcoholic fatty liver disease, and converges with aging processes [14,15]. For instance, our research group has recently demonstrated that perinatal obesity causes hepatic steatosis and imbalanced metabolic signaling later in life [16]. However, the early postnatal molecular mechanisms by which maternal obesity determines liver health remain poorly understood.

In the present study, we therefore investigated the molecular liver network in 3-week-old male murine offspring of obese and lean dams using a proteomic approach, followed by gene expression analysis, immunoblot, and immunohistochemistry. Since our experiments are primarily focused on a murine model, translation to humans is strongly limited. However, our overall goal was to identify early onset molecular mechanisms that disrupt hepatic development and may serve as new targets to prevent or reverse hepatic dysfunction and to attenuate obesity-mediated liver pathologies. Interestingly, maternal obesity caused a marked hepatic growth restriction in male offspring at postnatal day 21 (P21). Subsequent molecular analysis determined a WAT-linked liver intrinsic inflammatory response together with the activation of aging-related processes such as an increased DNA damage response (DDR) and senescence in the liver, indicating a possible premature aging of the liver.

## 2. Results

### 2.1. Perinatal Obesity Induces an Obese Body Composition and Hepatic Growth Restriction in the Male Offspring

**Figure 1:**
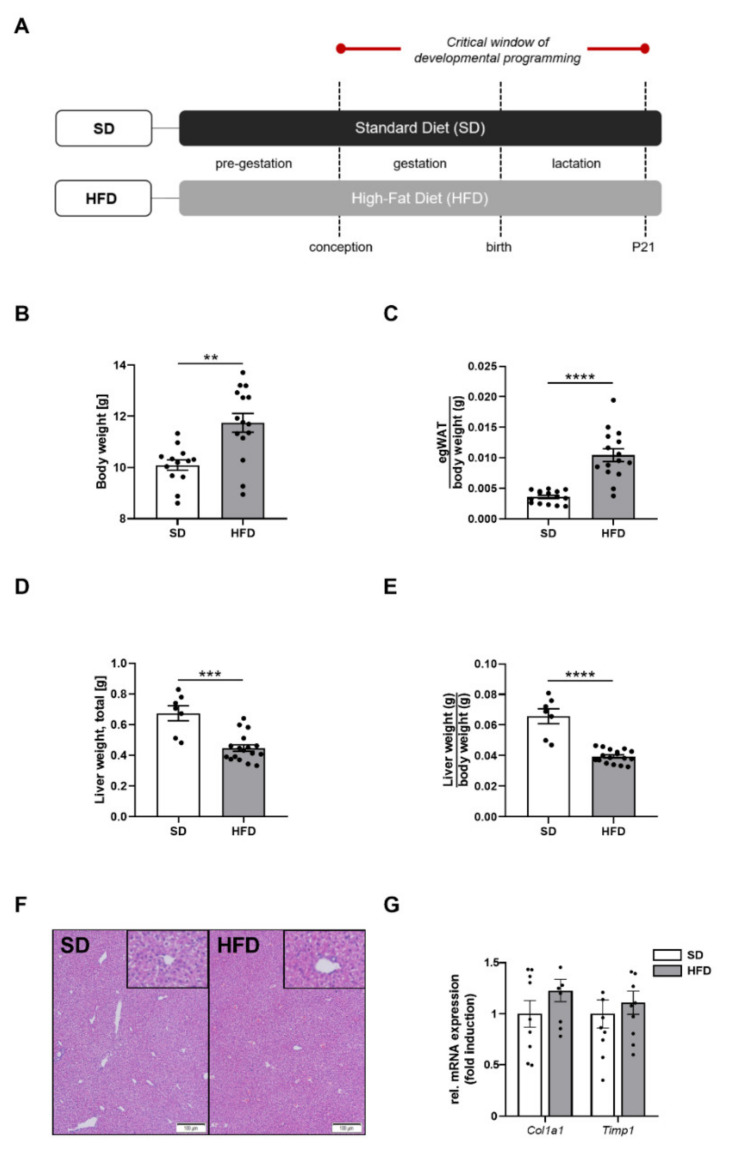
Maternal obesity alters offspring body composition and mediates hepatic growth restriction in early life. (**A**) Experimental design: HFD, dams received high-fat diet prior to mating as well as during pregnancy and lactation; SD, lean control group received standard laboratory chow. (**B**) Offspring’s total body weight at postnatal day (P) 21 (HFD: n = 16; SD: n = 15). (**C**) Offspring’s epigonadal fat pad weight, normalized to body weight at P21 (HFD: n = 16; SD: n = 15). (**D**) Total liver weight of the offspring at P21 (HFD: n = 16; SD: n = 7). (**E**) Relative liver weight, normalized to body weight of the offspring at P21 (HFD: n = 16, SD: n = 7). (**F**) Representative histological H&E stained liver sections of the offspring at P21. Magnification: 20×. (**G**) Assessment of genes encoding for markers of hepatic fibrogenesis (*Col1a1*, *Timp1*) using qPCR at P21. Data are presented as mean ± SEM; ** *p* < 0.01, *** *p* < 0.001, **** *p* < 0.0001. BW, body weight; Col1a1, collagen type Iα1; egWAT, epigonadal white adipose tissue; g, gram; HFD, high-fat diet; P, postnatal day; qPCR, quantitative real-time polymerase chain reaction; SD, standard diet; Timp1, TIMP metallopeptidase inhibitor 1.

To investigate the effects of perinatal maternal obesity on the offspring, we first established an HFD-induced obesity cohort of dams and subsequently analyzed the offspring body composition at P21, representing a critical time point of developmental programming. At P21, male offspring of obese dams exhibited a significantly increased total body weight (Figure 1B, *p* < 0.01) and an increased egWAT/body weight ratio when compared to offspring of lean dams, indicating an obese body composition in the offspring after maternal obesity (Figure 1C, *p* < 0.001).

Strikingly, both the absolute liver weight and liver/body weight ratio were significantly reduced by approximately 40% in male offspring of obese dams when compared to control offspring at P21 (Figure 1D, *p* < 0.001 and Figure 1E, *p* < 0.0001). Additional histological analysis of the liver samples at this time point did not reveal any marked changes in terms of an altered histological liver architecture, steatosis or fibrosis between both groups (Figure 1F). Similarly, expression of genes encoding for markers of hepatic fibrogenesis (*Col1a1*, *Timp1*) were unchanged between the two groups (Figure 1G).

### 2.2. Perinatal Obesity Induces a Specific Liver Proteome Signature and Changes Hepatic Molecular Processes in the Offspring

**Figure 2:**
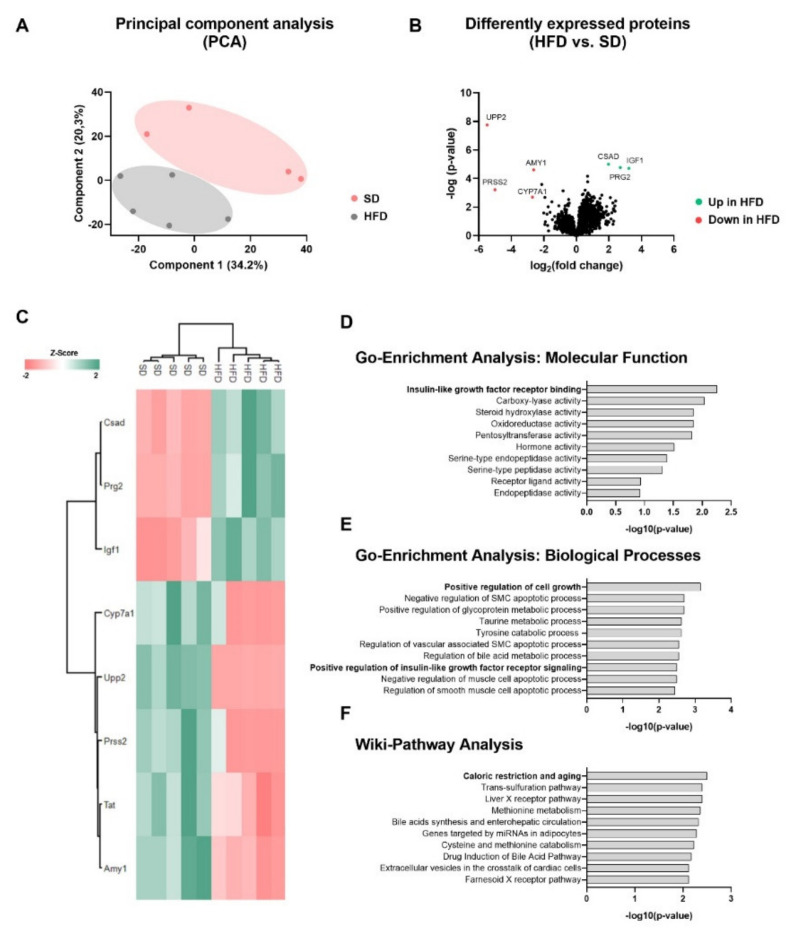
Perinatal obesity determines hepatic proteome signature in the offspring at postnatal day (P) 21. (**A**) Principal component analysis (PCA) with group specific clustering of protein expression variances between SD and HFD samples. (**B**) Volcano plot of univariate statistical analysis results from liver tissue samples of SD and HFD offspring. A volcano plot based on the fold change (Log2) and p value (−Log10) of all proteins identified in both groups. Green and red dots indicate the up- and downregulated proteins, respectively, that showed statistically significant changes using FDR and *q*-value < 0.05 for statistical analysis. (**C**) Heatmap of unsupervised 2-dimensional hierarchical clustering of the proteome profile of significantly altered proteins. The normalized Z-Score of protein abundance is depicted by a pseudocolor scale with red showing negative difference, white showing equal expression and green positive differential expression compared with the values of each protein. (**D**–**F**) Functional enrichment analysis to identify functional protein sets, including molecular functions (**D**), biological processes (**E**) and affected Wiki-pathways (**F**) of liver proteins that displayed significantly changed levels among SD and HFD offspring at P21 (n = 5 per group). Amy1; Alpha-amylase 1; Csad, cysteine sulfinic acid decarboxylase; Cyp7a1, cholesterol 7-alpha-monooxygenase; HFD, high-fat diet; Igf1, insulin like growth factor; Prg2, proteoglycan 2; Prss2, protease serine 2 preproprotein; SD, standard diet; Upp2, uridine phosphorylase 2.

To obtain comprehensive insights into the molecular networks underlying hepatic growth restriction, P21-liver samples were next subjected to proteomic analysis in order to identify the liver proteome signature of offspring of obese dams. Here, a total of 3824 proteins were identified and quantified with an FDR of 0.01 (see Supplementary Table S4). A principal component analysis (PCA) was generated showing a clearly different clustering between both groups of offspring HFD vs. SD (Figure 2A). A total of eight proteins were significantly altered from the abundance of all identified proteins between both groups using rigorous statistical criteria (FDR corrected *q*-value < 0.05). Notably, the most upregulated proteins among the differentially expressed proteins in male offspring of obese dams were IGF-1 (insulin-like growth factor 1), a key regulator of cell growth and differentiation [17,18], CSAD (cysteine sulfinic acid decarboxylase), as well as PRG2 (bone marrow proteoglycan), whereas UPP2 (uridine phosphorylase 2) and CYP7A1 (cytochrome P450 7A1) were among the most significantly downregulated proteins in male offspring of obese dams (Figure 2B). Hierarchical clustering of the significantly altered proteins revealed a heat map with a distinct liver protein expression pattern in the offspring (Figure 2C). To analyze the regulated pathways of the differentially expressed proteins, data were subjected to the EnrichR database to obtain insights into the most altered biological processes using gene ontology analyses (GO-Terms) and the strongest regulated Wiki pathways (Figure 2D–F). The analysis of differential expressed proteins revealed insulin-like growth factor signaling (GO-Enrichment analysis for molecular function), regulation of cell growth (GO-Enrichment analysis for biological processes), and caloric restriction and aging (Wiki pathway analysis) to be the most regulated processes in the liver at P21 after perinatal obesity (Figure 2D–F). A list of significantly altered pathways in the offspring by maternal diet is provided in Supplementary Table S5.

### 2.3. Perinatal Obesity Mediates Altered Insulin and Insulin-Like Growth Factor 1 Signaling in the Livers of the Male Offspring

**Figure 3:**
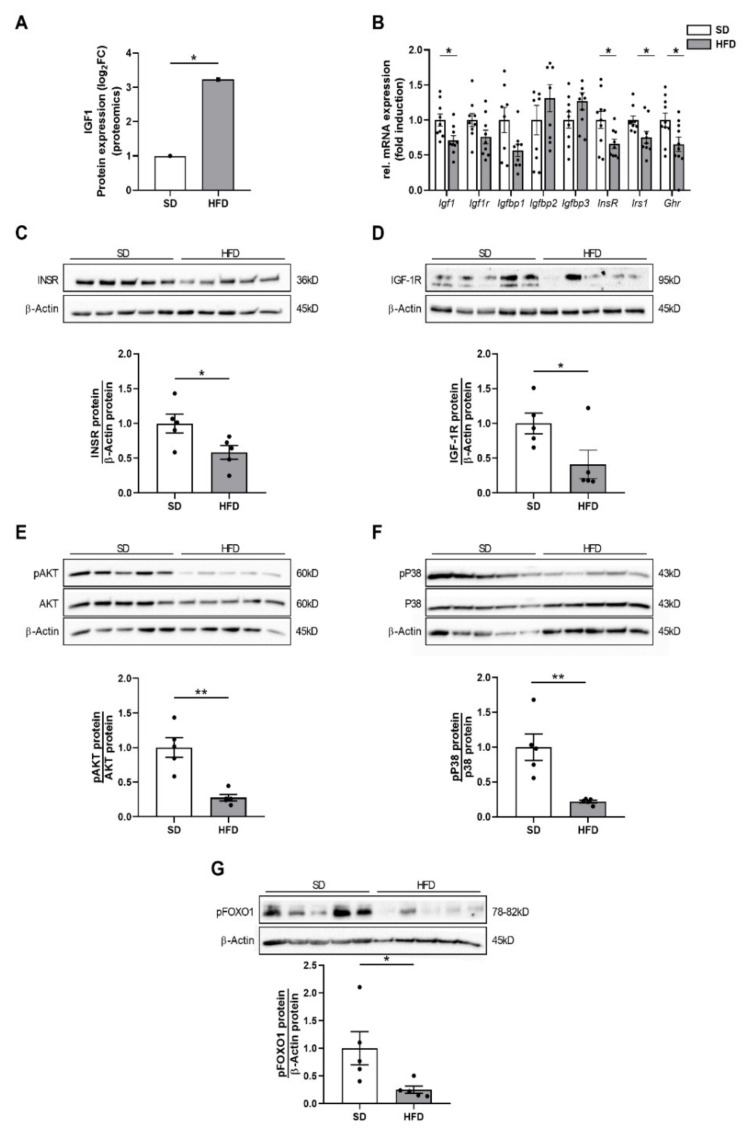
Perinatal obesity inhibits hepatic insulin/IGF-1 signaling. (**A**) IGF-1 protein expression in proteomic analysis at P21. (**B**) Measurement of genes encoding for IGF-1 (*Igf1*), IGF-1 receptor (*Igf1r*), insulin-like growth factor-binding proteins 1–3 (*Igfbp1-3*), insulin receptor (*Insr*), insulin receptor substrate (*Irs1*), and growth hormone receptor (*Ghr*) using quantitative real-time PCR (n = 10 per group). (**C,D**) Assessment of insulin receptor (INSR; **C**) and IGF-1 receptor (IGF-1R; **D**) protein abundance in liver homogenates using immunoblots. (E-G) Assessment of insulin/IGF-1 signaling pathway using immunoblot: phosphorylated AKT (pAKT) and total AKT protein expression (**E**); phosphorylated P38 (pP38) and total P38 MAP kinase protein expression (**F**); and phosphorylated FoxO1 protein abundance (**G**). β-Actin served as loading control; pAKT and pP38 were related to total AKT and total P38, respectively; pFoxO1 was related to β-Actin. Densitometric analyses are displayed below the respective immunoblots. Immunoblots: SD group (n = 5), HFD group (n = 5). Data are presented as mean ± SEM; * *p* < 0.05, ** *p* < 0.01. FoxO1, Forkhead-Box-O-1 transcription factor; HFD, high-fat diet; IGF-1, insulin-like growth factor 1; Igfbp, insulin-like growth factor-binding protein; Igfr, insulin-like growth factor receptor, Insr, insulin receptor; Irs1, insulin-receptor substrate 1; p, phosphorylated; P38, mitogen-activated protein kinase (MAPK) p38; SD, standard diet.

We next aimed to elucidate the mechanistic link between the molecular networks identified in the proteomic analysis and the marked hepatic growth restriction after maternal obesity. IGF-1, representing the most upregulated protein in the proteomic analysis, is an important mediator of tissue growth and cell proliferation (Figure 3A). Interestingly, the intracellular signaling of IGF-1 converges with the insulin/AKT/p38 pathway that is well-known to promote growth [17]. The significant upregulation of IGF-1 in livers of male offspring of obese dams together with hyperinsulinemia at P21, which has been observed in our prior studies [19], led us to investigate IGF-1 upstream mediators as well as the insulin and IGF-1 downstream signaling pathway in the liver. 

First, we found an upregulation of the insulin-like growth factor-binding proteins 2 (*Igfbp2*) 2 and 3 (*Igfbp3*) in the HFD offspring of obese dams by trend, which was consistent with the increased IGF-1 expression in the proteomic analysis. Furthermore, expression of genes encoding for IGF-1 (*Igf1*, *p* < 0.05), IGF-1 receptor (*Igf1r*, *p* = 0.0927), insulin receptor (*Insr*, *p* < 0.05), insulin receptor substrate (Irs1, *p* < 0.05), and growth hormone receptor (*Ghr*, *p* < 0.05) were downregulated (Figure 3B).

Second, assessment of protein expression using immunoblots showed a significant downregulation of the insulin receptor (INSR) and of the IGF-1 receptor (IGF-1R) in livers of male offspring of obese dams at P21, indicating a reduced hepatic insulin and IGF-1 signaling (Figure 3C,D, *p* < 0.05). This reduced INSR and IGF-1R protein abundance was accompanied by a significantly decreased phosphorylation of major downstream targets, including AKT (Figure 3E, *p* < 0.05), p38 mitogen-activated protein (MAP) kinase (Figure 3F, *p* < 0.01), and FOXO1 (Forkhead Box O1, Figure 3F, *p* < 0.05). These findings indicate an inhibition of hepatic IGF-1/insulin receptor downstream signaling, which is centrally involved in mediating liver growth and hepatic regenerative capacity, in male offspring of obese dams.

### 2.4. Perinatal Obesity Induces Expression of Inflammatory Markers in Egwat and Activation of Hepatic STAT3 Signaling in the Offspring

**Figure 4:**
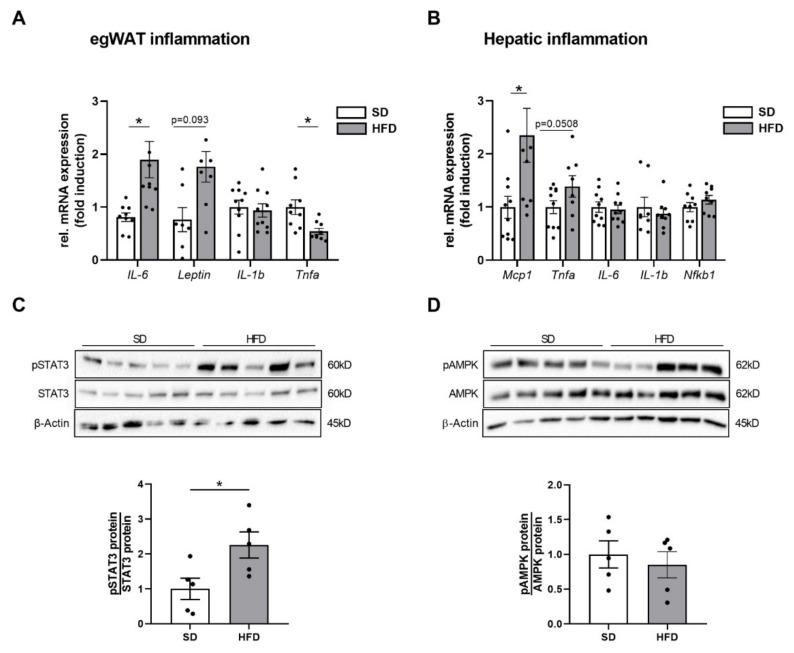
Perinatal obesity increases gene expression of inflammatory cytokines in egWAT and activates hepatic STAT3 signaling. (**A,B**) Assessment of the expression of genes encoding for inflammatory cytokines and chemokines at postnatal day (P) 21 using quantitative real-time PCR: egWAT (*Il6*, *Lep*, *Tnfa*, *Il1b* (n = 10 per group), **A**) and liver (*Mcp1*, *Tnfa*, *Il6*, *Nfkb1* (n = 10 per group), **B**). (**C**) Phosphorylated and total STAT3 (**D**) as well as phosphorylated and total AMPK protein expression were assessed by immunoblot; densitometric summary data show pSTAT3 and pAMPK relative to total STAT3 and AMPK, respectively. Densitometric analyses are displayed below the respective immunoblots. Immunoblots: SD group (n = 5), HFD group (n = 5). β-Actin served as loading control. Data are presented as mean ± SEM; * *p* < 0.05. AMPK, adenosine monophosphate-activated protein kinase; HFD, high-fat diet; Il6, interleukin 6; Il1b, interleukin 1b; Lep, leptin; Mcp1, monocyte chemoattractant protein 1; Nfkb1, Nuclear factor kappa B subunit 1; SD, standard diet; STAT3, signal transducer and activator of transcription 3; Tnfa, tumor necrosis factor alpha.

Since systemic inflammation is an important mediator of impaired insulin and IGF-1 signaling in obesity [20], we next assessed systemic inflammatory markers. WAT is considered the major source of inflammatory mediators and adipocytokines that cause a subacute inflammatory state and ultimately has systemic effects on other organs [14]. Since egWAT mass was significantly increased in male offspring of obese dams, we analyzed the inflammatory expression profile of egWAT using qPCR and found an increased mRNA expression of interleukin-6 (*Il6*; *p* < 0.05), whereas leptin (*Lep*; *p* = 0.093) was slightly increased, interleukin1-b (*Il1b*) unaltered and tumor necrosis factor alpha (*Tnfa*; *p* < 0.05) significantly downregulated (Figure 4A). We next queried, if the adipocytokine IL-6 might have a systemic effect on the liver and cause a hepatic inflammatory response. To this end, key determinants of hepatic inflammation were assessed using qPCR. The analysis revealed an increased expression of genes encoding for the monocyte chemoattractant protein-1 (*Mcp1*) and *Tnfa*, whereas the expression levels of other proinflammatory cytokines such as *Il6*, *Il1b*, and *Nfkb1* were not altered in livers of male offspring after maternal obesity at P21 (Figure 4B, *p* < 0.05). Since IL-6 activates the intracellular signal transducer and activator of transcription 3 (STAT3), a key regulator of proliferation and inflammation, we next assessed phosphorylation of STAT3 (pSTAT3) in livers and found a significant activation of hepatic STAT3 after maternal obesity (STAT3, Figure 4C, *p* < 0.05). This finding might indicate a possible metabolic inter-organ crosstalk and suggested an egWAT-liver communication with an activation of the IL-6/STAT3 axis that should be investigated in more detail. In addition to inflammatory processes, an activation of the IL-6/STAT3 pathway also plays an important role in the regenerative response of the liver to injury [21]. Concomitantly, protein expression levels of phosphorylated AMPK, which is also crucial for an intact liver regeneration upon activation [22], was not altered between both groups (Figure 4D).

### 2.5. Hepatic Growth Restriction after Perinatal Obesity Is Associated with DNA Damage Response and Expression of Cellular Senescence Markers

Reduction in liver weight normally occurs due to caloric restriction or during aging. Likewise, insulin/IGF-1 signaling is associated with cellular aging processes and was identified to be among the top regulated processes in the proteomic analysis of livers after maternal obesity in the present study [18]. Therefore, we next studied DNA damage and genomic instability, cellular senescence, and mitochondrial dysfunction, all recognized as hallmark processes of aging [23].

**Figure 5:**
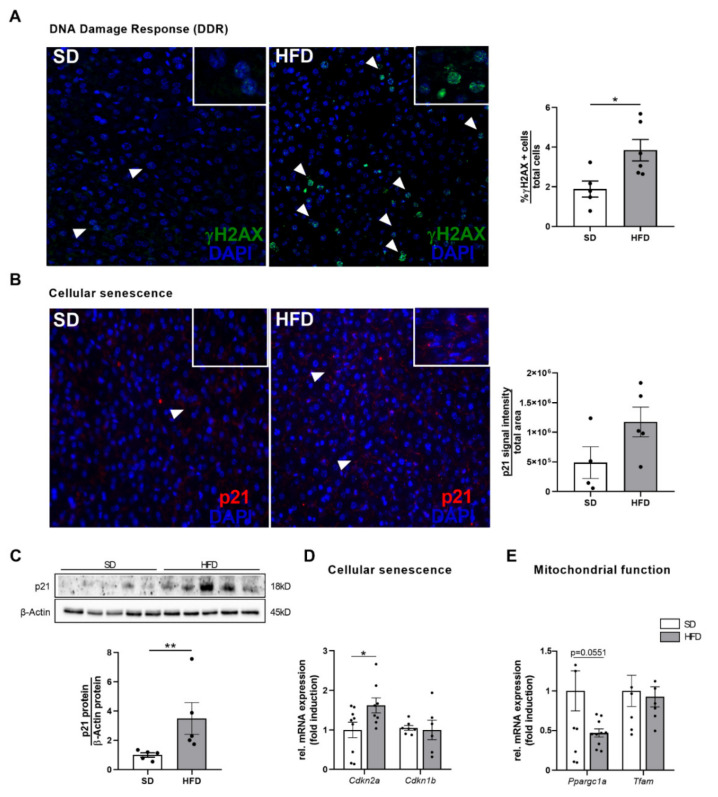
Perinatal obesity causes increased expression of hepatic markers of DNA damage response (DDR) and cellular senescence. (**A**) Assessment of hepatocellular DDR using immunofluorescence staining for γH2AX. Representative images (×10 magnification) of γH2AX + cells, white arrows depict γH2AX stained cells. (**B**) Quantification data of γH2AX + cells relative to all (DAPI) cells (n = 5 per group). (**C**) Assessment of p21 as marker of hepatocellular senescence using immunofluorescence staining. Representative images for p21 + cells (×10 magnification), white arrows depict p21 stained cells. (**D**) Quantification of p21 by measuring the p21 integrated density of the positive immunoflourescence signal per area (n = 5 per group). (**E**) Immunoblot shows the p21 protein abundance in total liver homogenate. Densitometric analysis is displayed below the respective representative immunoblots, n = 5 per groups, β-Actin served as loading control. (**F**) Measurement of genes encoding for markers of hepatocellular senescence (*Cdkn2a (p16), Cdkn1b (p27)*) using quantitative real-time PCR (n = 10 per group). (G) Assessment of gene expression of markers of mitochondrial biogenesis (*Ppargc1a* (Pgc1α), *Tfam*) using quantitative real-time PCR (n = 10 per group). Data are presented as mean ± SEM; * *p* < 0.05, ** *p* < 0.01. HFD, high fat diet; p21, cyclin-dependent kinase inhibitor 1A, p27; cyclin-dependent kinase inhibitor 1B; Ppargc1a, peroxisome proliferator-activated receptor gamma coactivator 1-alpha; Tfam, mitochondrial transcription factor A; SD, standard diet.

First, γH2AX, as a sensitive molecular marker of DNA damage (e.g., DNA double-strand breaks) and repair [24], was analyzed by immunofluorescence staining. Here, HFD offspring showed a significantly increased number of γH2AX positive foci, indicating a significant increase in hepatocelluar DNA damage response (DDR) due to perinatal obesity (Figure 5A, *p* < 0.05). Interestingly, the yH2AX positive cells were preferentially found in the centrilobular area, which is exposed to higher levels of oxidative stress [25]. Since DNA damage is intimately linked to the initiation of a cell cycle arrest and the onset of a senescent phenotype, we next measured important cell cycle inhibitors and markers of cellular senescence including the cyclin-dependent kinase inhibitors 2A (p16/CDKN2A) and 1A (p21/CDKN1A). The hepatic p21 protein abundance as a crucial negative regulator of cell cycle progression was significantly increased in male offspring of obese dams at P21 (*p* < 0.05). Immunofluorescent staining supported this finding showing a marked trend of higher p21 intensity in livers of HFD than in livers of SD offspring (Figure 5B,C). The higher expression of p21 after perinatal obesity was associated with a significant upregulation of the gene encoding for p16 (*Cdkn2a*), which may be essential for a maintenance of the senescent cell cycle arrest (Figure 5D, *p* < 0.05). Focusing on markers of mitochondrial dysfunction, we found a downregulation of the peroxisome proliferator-activated receptor gamma co-activator 1α (*Ppargc1a*, Figure 5E, *p* = 0.0551), as a key transcriptional regulator of mitochondrial biogenesis, whereas mitochondrial transcription factor A (*Tfam*) mRNA was unchanged between SD and HFD offspring (Figure 5E).

### 2.6. Hepatic Growth Restriction in HFD Offspring of Obese Dams Is Related to Markers of Increased Cell Proliferation

DNA damage and cell cycle arrest are in a reciprocal regulatory relationship with cell proliferation in order to maintain cellular homeostasis and mediate tissue regeneration. To determine whether the observed hepatic growth restriction-associated changes were counterbalanced by an adequate compensatory proliferative response, analyses of different markers of hepatocellular proliferation, growth, and apoptosis were performed. 

**Figure 6:**
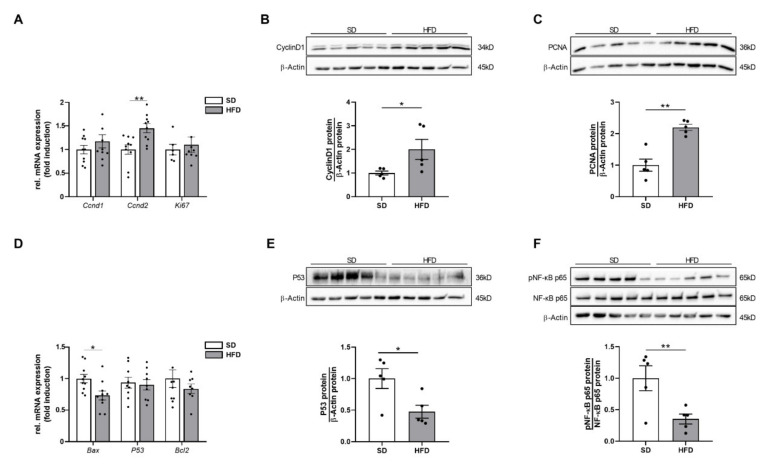
Hepatic growth restriction after perinatal obesity is related to increased expression of proliferative markers. (**A**) Assessment of gene expression of markers of cell proliferation and cell cycle regulation (*Ccnd1*, *Ccnd2*, *Ki67*) in livers at P21 using quantitative RT-PCR. (**B,C**) Immunoblots showing protein abundance of Cyclin D1 (**B**) and PCNA (**C**) in livers at P21. (**D**) Measurement of gene expression of apoptotic markers (*Bax*, *P53*, *Bcl2*) using quantitative RT-PCR. (**E,F**) Assessment of P53 (E), and phosphorylated as well as total NF-κB p65 (**F**) protein abundance in livers at P21 (n = 10 per group). Immunoblots: SD group (n = 5), HFD group (n = 5). β-Actin served as loading control. Densitometric analyses are displayed below the respective immunoblots. Data are presented as mean ± SEM; * *p* < 0.05, ** *p* < 0.01. Bax, bcl-2-associated x protein; Bcl2; B-cell lymphoma 2; Ccnd1, cyclin D1; Ccnd2; cyclin D2; HFD; high-fat diet; NF-κB, nuclear factor kappa B; p; phosphorylated; P53, tumor protein p53; PCNA, proliferating cell nuclear antigen; SD, standard diet.

First, the expression of different cyclins was investigated. Cyclins are important in the regulation of cell cycle progression, DNA damage sensing, and initiation of DNA damage repair [26]. Here, gene expression of *Ccnd2* and cyclin D1 protein abundance were significantly upregulated in HFD offspring when compared to lean controls (Figure 6A, *p* < 0.05; Figure 6B, *p* < 0.01). In line with these findings, proliferating cell nuclear antigen (PCNA) protein, another important indicator of cell proliferation, was also significantly increased in the male offspring of obese dams at P21 (Figure 6C, *p* < 0.01), further supporting the notion of a possible ongoing hepatic regenerative activity. 

Conversely, the mRNA expression level of *Bax*, an apoptosis-related marker, was significantly downregulated in qPCR analysis (Figure 6D, *p* < 0.05). Similarly, the protein expression of p53 (Figure 6E, *p* < 0.05), as a major mediator of cellular apoptosis, and of phosphorylated NF-kappaB p65, which may promote apoptotic processes [27], was significantly downregulated (Figure 6F, *p* < 0.01).

The aforementioned proliferation markers (cyclin D1, cyclin D2, PCNA) are controlled by p38 MAP kinase, AKT, and STAT3 signaling [28]. Prior studies demonstrated that IL-6-mediated STAT3 activation induces hepatocyte proliferation and enhances restoration of liver mass and liver regeneration after injury [21,29,30]. Since hepatic IL-6/STAT3 signaling is active after perinatal obesity in the present study, it might be mechanistically linked to pro-proliferative and anti-apoptotic processes in the liver at P21. In contrast, the other central signaling pathways, AKT and p38 MAP kinase, which are also responsible for adequate liver regeneration, were deactivated, possibly accounting for an inadequate and insufficient regenerative response.

## 3. Discussion

The present study demonstrates that HFD-induced maternal obesity causes an obese body composition and hepatic growth restriction in the male offspring in early life. This is accompanied by changes in the hepatic protein network and alterations of key molecular processes as identified by the proteomic analysis. Molecular analyses indicate that the impaired hepatic growth pattern might be mechanistically linked to (1) an altered insulin/IGF-1 AKT-p38-pFoxO1 downstream signaling, (2) an activation of hepatocellular senescence and increased DNA damage response, as well as (3) an inadequate compensatory proliferative growth response. These aging-related processes are intimately linked to a possible inflammatory egWAT-liver axis with an activation of IL-6/STAT3 signaling. Collectively, we provide initial evidence that perinatal obesity induces aging-related processes and could lead to premature liver aging with hepatic growth restriction in the offspring and may ultimately increase the susceptibility to chronic liver disease later in life.

Maternal obesity represents an emerging public health burden in Western countries. Specifically, accumulating evidence indicates that an exposure to an adverse early life metabolic environment increases the offspring’s risk for obesity and chronic liver diseases later in life [13]. In line with previous clinical and experimental studies [31], the present study confirmed that maternal HFD-induced obesity is associated with an obese body composition in the male offspring already in early life, indicating a vicious intergenerational cycle of obesity. Moreover, offspring of obese dams exhibited a significantly reduced liver weight, despite their increased body weight and obese body composition. A reduction in liver weight is often a result of disturbances of hepatic blood supply, congenital abnormalities or follows acute hepatocellular injury with a severe loss of hepatocyte mass [32], for all of which no evidence was found in the present histological analysis. However, reduced liver size has also been associated with intrauterine growth restriction (IUGR), which is often caused by maternal undernutrition or dietary restriction during pregnancy [33] as well as maternal obesity [34]. IUGR is defined as the inability of the fetus to exploit its genetically determined growth potential [34]. A recent study from Lewandowska and colleagues demonstrated that the risk for IUGR is up to three times higher in infants of obese mothers than in those of mothers with normal pre-pregnancy BMI [35]. IUGR in obese mothers is mainly attributed to a placental dysfunction caused by maternal proinflammatory adipocytokines and metabolic hormones, resulting in a restricted placental nutrient supply to the developing fetus [34]. While previous studies have shown that maternal obesity can be associated with reduced kidney growth and altered cardiac organ structure in the offspring due to IUGR [36,37], information regarding the effect of maternal obesity on liver growth and development remain scarce. Based on the findings of the present study, it can be assumed that maternal obesity-associated IUGR seems to be another potential risk factor for impaired liver growth. However, in order to get deeper insights into the underlying mechanisms in the present experimental model, the maternal metabolism as well as the placental function should be examined more closely in future studies.

Reduced liver size with progressive loss of liver volume is closely associated with aging [23]. While in humans’, liver weight decreases successively by 20-40% after the age of 40. Lessard-Beaudoin et al. also observed a progressive reduction in liver weight in C57BL/6N mice during physiological aging [38]. These structural changes are associated with a decreased hepatic blood flow, declined drug metabolism, diminished hepatobiliary functions, and a loss of functional liver cell mass [38]. Interestingly, we observed a marked reduction in liver weight by almost 40% at the age of just three weeks (P21) after maternal obesity, indicating an aberrant liver development with obesity-associated hepatic growth restriction. The decreased liver weight in male offspring of obese dams was related to altered biological processes that are centrally involved in aging, supporting the notion of an early postnatal activation of premature aging mechanisms in the liver. Those hallmarks of aging comprise (1) an increased number of γH2AX (+) cells as an indicator of accumulation of DNA damage, (2) upregulation of senescence markers such as p21 and p16, and (3) deregulation of metabolic signaling pathways (insulin/IGF-1-AKT-pFoxO1 signaling).

**(1)**  **DNA damage response in the liver after perinatal obesity**

γH2AX represents a sensitive molecular marker of DDR and reflects an early cellular response to DNA double-strand breaks [24]. DNA double-strand breaks induce terminal cell cycle arrest and cellular senescence. Mice harboring DNA double-strand repair defects display multiple symptoms of premature aging and reveal a reduced lifespan [39]. In a recent study by White and colleagues, the authors demonstrated that induction of DNA double-strand breaks in the liver of young mice (3-month-old) using an adenoviral vector construct containing a restriction endonuclease was associated with symptoms of premature aging similar to those seen in untreated livers of physiologically aged control mice [40]. Interestingly, the young mice recovered partly from the observed aging characteristics (e.g., γH2AX-positive cells number) at 2-month follow-up, possibly due to a sufficient cellular regenerative capacity. 

Accumulating experimental and clinical evidence demonstrate that (diet-induced) obesity may also induce DNA damage and inhibit DNA repair mechanisms [41], thereby triggering premature aging processes. In a rodent model, HFD significantly increased hepatic γH2AX positive cells; analogous to this, human data revealed an elevated level of hepatic DNA damage in children with overweight or obesity when compared to lean controls [41,42]. Similar to this study, our data demonstrate an early accumulation of DDR in the liver after maternal obesity, indicating possible perinatal obesity-induced premature aging of the liver. However, further studies are required to test, whether the early postnatal genomic instability with hepatic growth restriction persists, aggravates, or even reverses later in life due to regenerative processes.

**(2)**  **Cellular senescence in the liver after perinatal obesity**

In the absence of an adequate repair, DNA damaged cells can either undergo apoptosis or become senescent. Cellular senescence is a state of irreversible cell-cycle arrest, which can be induced by a variety of stressful insults, including telomere dysfunction, genotoxic and oxidative stress or inflammation [43]. While senescent cells are not able to proliferate, they remain metabolically active [43,44]. Senescent cells secrete a variable set of factors, including cytokines, chemokines, angiogenic factors, and growth modulators, termed as the senescence-associated secretory phenotype (SASP) [44]. These factors allow the senescent cell to communicate with their microenvironment, and thereby arresting the cell-cycle, inducing senescence in adjacent cells, and engaging the innate immune system for clearance of senescent cells [44].

Focusing on the liver, cellular senescence contributes to the development and progression of age-related liver diseases and is closely associated with impaired liver regeneration [25,44,45]. Senescent cells have been found in the livers of HFD-fed mice and might be driven mainly by stress-induced DNA damage [25]. An accumulation of senescent cells has been shown to promote hepatic fat accumulation and steatosis, a pathologic condition related to decreased mitochondrial capacity to oxidize fatty acids [46]. In the present study, senescent markers such as p16 and p21 were significantly upregulated, whereas *Ppargc1* was reduced (*p*-value = 0.0551), indicating an early onset of senescence. However, male offspring of obese dams did not show signs of steatosis at P21. Therefore, it can be speculated that such young animals still had sufficient compensatory cellular fatty acid oxidation capacity to counteract increased hepatic fat accumulation. Future studies should investigate hepatic lipid metabolism in more detail, and test if hepatic fat accumulation manifests during the further life course.

**(3)**  **Dysregulated insulin/IGF-1-AKT-pFoxO1 signaling in the liver after perinatal obesity**

The insulin/IGF-1 signaling pathway represents a highly conserved pathway which integrates growth signals with nutrient status and regulates mitogenesis and survival of different liver cell types [47]. In particular, IGFs play a crucial role in cell proliferation, differentiation, and metabolism. Several transgenic mouse models have demonstrated that IGF-1 is required for regular tissue growth and normal fetal development [17]. The liver is the predominant source of circulating IGF-1 (70–80%) which consequently can mediate auto-, para-, and endocrine effects [47]. IGF-1 primarily signals through the IGF-1R, a transmembrane tyrosine kinase that structurally and functionally resembles the INSR, but also signals through the INSR itself [29]. The present study shows that a significant reduction of both IGF-1R and INSR protein abundance in the liver of obese offspring at P21 was related to decreased activation of their intracellular AKT-FoxO1 signaling cascade. These findings suggest a receptor-mediated insulin/IGF-1 resistance with altered downstream signaling as a possible underlying cause of the hepatic growth restriction with inadequate regenerative and proliferative response after perinatal obesity. 

These results are in line with the study findings from Beyer et al., who identified an oxidative stress-mediated insulin/IGF-1 resistance with impaired liver regeneration in mice after partial hepatectomy [48]. Similar to the present study, the authors found an impaired activation of AKT and p38 signaling with reduced proliferation of hepatocytes and inadequate compensatory liver growth. Interestingly, mice with a liver-specific IGF-1R knockout also displayed a strong impairment in hepatocyte proliferation and regeneration after partial hepatectomy [49]. Furthermore, a recent study by Morales-Garza et al. demonstrated that IGF-1-deficient mice showed a reduced total liver weight on postnatal day 11, whereas subcutaneous IGF-1 administration attenuated this growth retardation, highlighting the important role of IGF-1 in liver growth [50]. However, these data are only in part comparable to our present experimental model, in which we expose the liver during fetal development and during the postnatal period to an adverse metabolic microenvironment. Interestingly, there are few studies confirming that the intrauterine nutrient supply and metabolism determine liver structure and function. For example, Tosh et al. reported that newborn rodents with IUGR due to intrauterine nutrient restriction exhibited significantly lower liver weight and reduced plasma IGF-1 levels, whereas hepatic IGF-1 protein levels were significantly increased [51]. Further mechanistic analysis revealed epigenetic changes with an altered hepatic histone methylation status in the IGF-1 region that might result in disruption of hepatocellular exocytosis of IGF-1. Since maternal obesity may cause IUGR and mediates adverse transgenerational effects [34], epigenetic alterations could in fact underly the changes in IGF-1 mRNA and protein abundance and should be analyzed in future studies. Moreover, the differences in IGF-1 mRNA and protein abundance could be caused by post-translational regulation or protein degradation.

**(4)**  **Activation of an inflammatory egWAT- liver axis after perinatal obesity might trigger aging processes**

Obesity-related alterations of insulin/IGF-1 signaling as well as premature aging processes are significantly caused by a prevailing systemic inflammation, the latter being known as ‘inflamm-aging’. Inflamm-aging is a chronic subacute inflammatory response that is intimately linked to the progression of aging and age-related pathologies [15]. Overactive WAT in obese conditions produces a variety of pro-inflammatory cytokines, notable amongst those is IL-6, and triggers thereby chronic inflammation. In the present study, we show an almost two-fold increased *Il6* expression in egWAT of male offspring of obese dams at P21 that was related to marked activation of STAT3 signaling in the liver, indicating a possible inter-organ crosstalk, and suggesting an egWAT-liver axis. Inflammation and IL-6 do not only trigger DDR and senescent processes, but also regulate insulin/IGF-1 signaling. However, while it has been shown that certain proinflammatory cytokines such as TNFα and IL-1β induce hepatic IGF-1 resistance [52], the contribution of IL-6 signaling in obesity-induced inflammation to hepatic insulin and IGF-1 downstream signaling remains controversial [21]. Previous studies demonstrated that IL-6 triggers hepatic insulin resistance by inhibiting insulin signaling in vivo and in vitro and could thereby be involved in obesity-related hepatic insulin resistance [53]. In contrast, IL-6 simultaneously plays an essential role in mediating and initiating liver regeneration [21]. For example, IL-6 is crucial for hepatocyte homeostasis and IL-6-induced STAT3 signaling is responsible for the G_0_ to G_1_ cell-cycle transition of hepatocytes resulting in hepatocyte proliferation and liver tissue regeneration [29]. However, in order to better evaluate a possible egWAT-liver inter-organ crosstalk via IL-6-induced STAT3 signaling, the plasma concentrations of IL-6 should be additionally analyzed in future studies.

Together with an activation of the hepatic IL-6-STAT3 signaling pathway, we also detected a significant increase in proliferation markers including PCNA and cyclin D1. In particular, cyclin D1 represents an important marker as it simultaneously acts as a sensor for DNA damage [26]. Thus, increased expression of cyclin D1 could also be interpreted as a consequence of the detectable increased DDR. However, the detectable proliferative response seems to be insufficient to induce adequate and sufficient catch-up growth of the liver.

**Figure 7:**
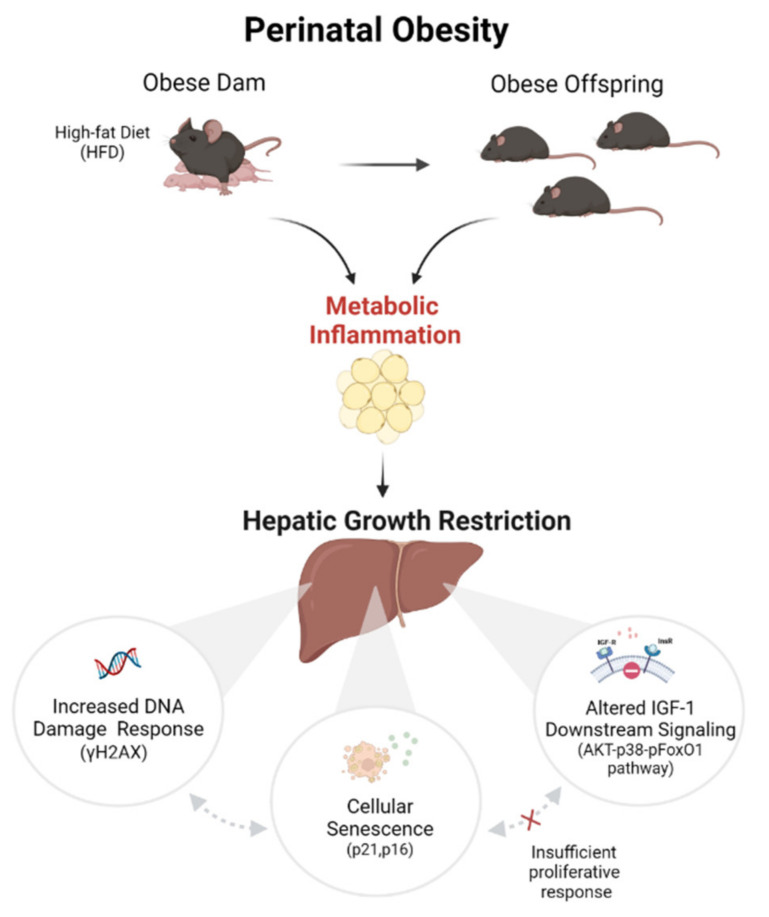
Working model. Proposed working model illustrating possible mechanisms by which perinatal obesity causes hepatic growth restriction: HFD-induced maternal obesity induces an obese body composition with increased expression of *Il6* in white adipose tissue (WAT). This WAT-related inflammatory response is linked to active hepatic STAT3 signaling and hepatic growth restriction in the offspring in early life (postnatal day 21, P21). Based on an unbiased proteomic analysis we identified three molecular mechanisms that might contribute to the hepatic growth restriction after perinatal obesity: (1) an increased DNA damage response (DDR), (2) hepatocellular senescence, and (3) inhibition of insulin/IGF-1-mediated AKT-pFoxO1 signaling, resulting in an inadequate compensatory proliferative growth response. These findings let us speculate that perinatal obesity induces aging-related processes and could cause premature liver aging with hepatic growth restriction in the offspring. Created with BioRender.com.

Overall, in the present study hepatic growth restriction could result from the interplay of the discussed processes leading to the following proposed pathological sequence: perinatal obesity-related inflammation induces increased hepatic DNA damage in the offspring, thereby leading to increased cellular senescence. This is accompanied by a reduced activity of the pro-proliferative IGF1-AKT-FoxO1 axis, resulting in an inadequate compensatory proliferative response and ultimately insufficient regenerative growth (Figure 7).

### Limitations of the Study

Specific limitations have to be taken into account when interpreting the results of the present study. First, phenotyping, histological, and molecular analyses were only performed in male offspring at P21. To further elucidate sex-specific differences in hepatic metabolism of the offspring due to maternal obesity (e.g., mediated by sex hormones), future studies should compare both male and female offspring. However, since previously published studies examining the effects of maternal obesity on the offspring also used male offspring only, our study provides well comparable information. Second, molecular analysis was only performed at one single time point in early life, on postnatal day 21. In order to determine whether these early findings persist and result in long-term adverse sequelae for liver function and regeneration, additional time points need to be investigated in the future. This is central to test the hypothesis if the altered markers of cell cycle and proliferation in the offspring of obese dams indicate an insufficient compensation of early hepatic growth restriction due to impaired regenerative catch-up growth. Third, while the scope of the present study was liver size and aging-related processes, future studies should address the metabolic capacity and function of the liver after maternal obesity. Fourth, liver weight was recorded only at P21 in the present study. In future studies, liver weight and liver size should be additionally measured during the fetal period, at birth, and regularly during the early postnatal period (P1–P21) to gain detailed knowledge about the dynamic liver growth pattern of the offspring of obese dams during different windows of organ development. This should be complemented by recording the liver weight of the dams to further understand the metabo-hepatic interrelationship between dams and offspring. Finally, the present experimental design does not distinguish between the individual contribution of maternal obesity, maternal diet, and maternal metabolism. It remains uncertain, whether the described findings are an indirect effect (obesity and metabolism) or a direct effect of the HFD (diet composition, e.g., fatty acids). Although the translation of findings from rodent studies to humans is difficult and should be discussed critically, our results indicate that maternal obesity might adversely interfere with offspring liver development and emphasize the need for time- and cost-intensive long-term mother–child studies in men. Future translational approaches should include data on infants with overweight/obesity as well as data on maternal body mass index and metabolic health.

## 4. Materials and Methods

### 4.1. Animal Procedures

The present study was carried out by the Department of Pediatric and Adolescent Medicine of the University Hospital of Cologne. All animal procedures were performed in accordance with the German regulations and legal requirements and were approved by the appropriate governmental authority (Landesamt für Natur, Umwelt und Verbraucherschutz (LANUV), North Rhine-Westphalia, Germany (Protocol number of the animal welfare application: 2012.A424; 2018.A230).

#### Animal Studies

C57BL/6N mice were kept and bred at the animal facility of the Department of Pharmacology of the University Hospital of Cologne (Cologne, Germany), in humidity-controlled (50% to 60%) and temperature-controlled (22 ± 2 °C) rooms with a 12-h dark/light cycle and had ad libitum access to water and their respective chow. Maternal obesity was induced in young virgin female mice by feeding an obesogenic high-fat diet (HFD (n = 14); modified, catalog no. C1057; Altromin, Lage, Germany; containing a total metabolizable energy (MetE) of 5237 kcal/kg, 60% of MetE from fat, 16% of MetE from protein, 24% of MetE from carbohydrates of which 7% were sugar) seven weeks prior to conception. In parallel, a second group of female mice received a standard diet and served as the lean control group (SD (n = 9); catalog no. R/M-H V1534-0; Ssniff, Soest, Germany; containing a total MetE of 3225 kcal/kg, 9% of MetE from fat, 24% of MetE from protein, 67% of MetE from carbohydrates of which 8.8% were sugar). An overview of the specific diet composition is provided in Supplementary Table S1. HFD and SD dams were time-mated with SD-fed male mice and continued on their respective diets throughout the gestation and lactation period. Following the HFD feeding period, dams exhibited significantly increased body weight and impaired glucose tolerance, as previously demonstrated by our research group [19,31]. This indicates that the offspring were exposed to a disturbed intrauterine metabolic environment during fetal development. At birth, the litter size of all dams was randomly normalized to six for each litter. While litters with reduced litter size could contain both male and female offspring, depending on the litter composition, only male offspring were further studied in the present study. Postpartum, male offspring of both groups (SD vs. HFD) were named after the maternal conditions. After weaning at P21, the offspring were sacrificed for organ harvest. To exclude sex influences, all studies were performed using male offspring in accordance with previous study settings [19,54]. The exact numbers of animals are listed in the figure legends. The animal model of perinatal metabolic programming was performed as previously described [19,55], and the experimental protocol is shown schematically in Figure 1A.

### 4.2. Tissue Preparation

The animals were sacrificed at P21 in a non-fasted state and always at the same time of day (morning). The liver and epigonadal white adipose tissue (egWAT) were excised, weighed, immediately snap-frozen in liquid nitrogen, and stored at −80 °C for protein analysis and assessment of gene expression. Liver tissue was additionally fixed in 4% paraformaldehyde (PFA) for histological and immunohistochemical analyses.

### 4.3. Histological Analysis of the Liver Tissue

The histological analysis of liver samples was performed as previously described [16,56]. In brief, upon sacrificing the mice at P21, the liver tissue was excised and immediately fixed in 4% PFA. Afterwards, liver samples were embedded in paraffin and sectioned at 3 µm. Slices were stained with hematoxylin and eosin (H&E) for microscopic examination; the degree of steatosis, type of steatosis, lobular inflammation, and hepatocellular ballooning were evaluated as previously described [57]. The liver sections were analyzed by an expert liver pathologist, who was blinded to dietary conditions.

### 4.4. RNA Extraction and Real-Time Reverse Transcription Polymerase Chain Reaction (RT-PCR)

Total RNA was isolated from frozen liver tissue using Tri-Reagent^®^ (Sigma-Aldrich, Steinheim, Germany). Afterwards, extracted RNA was transcribed into cDNA and quantitative real time reverse transcription polymerase chain reaction (qRT-PCR) was performed using the 7500 real-time PCR system (Applied Biosystems, Foster City, CA, USA) as described previously [19,54]. Gene expression was normalized to β-Actin. Primer pairs and TaqMan probes used in this study are listed in Supplementary Table S2.

### 4.5. Protein Isolation and Immunoblotting

Protein isolation and immunoblotting were performed as described previously [19,56]. Briefly, for protein extraction, frozen liver samples were completely homogenized and mixed with a specific protein extraction buffer (1% sodium dodecyl sulfate [SDS], 6.65 mol/L urea, 10% glycerol, 0.5 mmol/L phenylmethanesulfonylfluoride, and 10 mmol/L Tris-HCl pH 6.8, 5 mmol/L dithiothreitol) as previously described [19,56]. Protein concentration was determined using a bicichoninic acid (BCA)-protein assay kit (Thermo Scientific, Waltham, MA, USA). Subsequently, immunoblotting of liver samples was performed as previously described [16].

Immunoblots were probed with the antibodies listed in Supplementary Table S3. For quantitative immunoblot analysis, densitometry was performed using Bio-Rad ImageLab software (Bio-Rad, Munich, Germany). Protein samples were normalized using β-Actin as the loading control from the same samples. Original blots are provided in the Supplementary Materials.

### 4.6. Proteomic Analysis

Proteomic analysis was performed as previously reported [56,58]. First, protein isolation was done as described above for immunoblot analysis. Subsequently, protein acetone precipitation was performed, and proteins were digested into peptides with lysyl-endopeptidase (Lys-C) and trypsin. Peptides were purified using specific styrene divinyl benzene- reversed-phase (SDB-RP) stage tips and stored at 4 °C prior to analysis. All liver samples were analyzed on an Orbitrap Exploris 480 (Thermo Scientific, Waltham, MA, USA) mass spectrometer, that was equipped with a FAIMSpro differential ion mobility device coupled to an Easy 1200 nano LC (Thermo Scientific, Waltham, MA, USA), as previously described [56,58]. Peptides were chromatographically separated at a constant flow rate of 300 nL/min and the Orbitrap was operated in DIA mode. For proteomic data processing, generated thermo raw files were demultiplexed and transformed to mzML files using the ms-convert module in Proteowizard. For generation of a spectral library, a mouse canonical Swissprot FASTA file was converted to a specific upload file (Prosit) with the convert tool in an encyclopedia using default settings. Subsequently, a csv file was generated and uploaded to the Prosit webserver and converted to a spectrum library in generic text format. Based on the resulting library, which was searched in DIA-NN 1.7.18 [59], a project-specific library was then created. False discovery rates (DFR) on protein and peptide spectrum match (PSM) level were estimated by the target-decoy approach to 1% (Protein FDR) and 1% (PSM FDR), respectively. Protein quantification was conducted using data-independent acquisition. Data analysis was performed using Perseus software (version 1.6.15.0) and data filtering was performed using the *q*-value option.

### 4.7. Immunofluorescent Staining

Immunofluorescent staining was performed as described previously [60]. Briefly, PFA-fixed liver sections (3 µm) were first deparaffinized in Neo-Clear^®^ (Sigma–Aldrich, St. Louis, MO, USA), rehydrated in a graded ethanol series (100%, 96%, 80% and 70% for one minute each) to PBS and then treated with MaxBlock™ reagent A autofluorescence reducing kit (MaxVision Biosciences, #MB-L, DC, Kenmore, WA, USA). Afterwards, slides were washed with 60% ethanol, aqua dest, and PBS, and antigen retrieval was performed by boiling with 10 mM citrate buffer (DAKO, catalog no. S2369, pH6, Santa Clara, CA, USA) at 90–120 °C or rodent Decloaker (BioCare Medical, Pacheco, CA, USA) for 25 min. Subsequently, tissue sections were treated with 3% peroxidase block and SeaBlock blocking solution (Sea Block, Thermo Fisher Scientific, catalog no. 37527, Waltham, MA, USA) at room temperature for 1 h. Afterwards, sections were incubated with the primary antibodies γH2AX (Cell Signaling, Danvers, MA, USA, rabbit anti-mouse γH2AX (phospho, S139), catalog no. 2577; 1:400) or p21 (Invitrogen, Waltham, MA, USA, mouse anti-mouse p21, catalog no. MA5-31479; 1:200) diluted in antibody dilent (DAKO, catalog no. S0809) in the dark at 4 °C overnight. Subsequently, slides were washed with PBS and liver sections stained for p21 or γH2AX and were then incubated with mouse anti-CY3 (Sigma-Aldrich, #C6198, St. Louis, MO, USA, 1:200) or goat secondary antibody, conjugated with an F488 fluorochrome (1:500) for 1 h, respectively. Next, liver sections were treated with solution B from the MaxBlock autofluorescence reducing kit (MaxVision Biosciences, cataol no. MB-L, Washington, DC, USA) for 5 min at room temperature to enhance the fluorescent signal and slides were mounted with glass coverslips using Fluoromount Aqueous mounting medium (Sigma Aldrich, catalog no. F4680, St. Louis, MO, USA). DAPI dye (Sigma-Aldrich, cataol no. D9542, Taufkirchen, Germany, 1:5000) was used to counterstain the nucleus. Images of liver sections were taken using a fluorescence microscope (Olympus, Hamburg, Germany) at 40X and 100X magnification (CellSens Dimension software; Olympus, Tokyo, Japan). Quantification of γH2AX was performed by counting γH2AX -positive (+) cells relative to all DAPI (+) cells (%). Quantification of immunostained p21 was performed by measuring the p21 integrated density of the positive immunofluorescence signal by using the ImageJ analysis software (version 1.51). Ten fields of view per liver section and five animals per group were analyzed, and the mean value was calculated.

### 4.8. Statistical Analysis

All values are expressed as mean ± standard error of mean (SEM). The real-time qRT-PCR results were calculated based on the 2^−∆∆Ct^ method and were expressed as fold induction of mRNA expression compared to the corresponding control group (1.0-fold induction) as previously described [19,54]. For the comparison of measurements between the two analyzed study groups (SD vs. HFD), we performed either an unpaired *t*-test for parametric distribution or performed a Mann–Whitney U-test for non-parametric distribution. Statistical significance was defined as *p* < 0.05 and indicated with * *p* < 0.05, ** *p* < 0.01, and *** *p* < 0.001 within the respective graphs. The statistical analysis was performed using the Graph Pad Prism software (GraphPad version 8.0, San Diego, CA, USA). Proteomic data analysis was performed with Perseus software (version 1.6.15). Briefly, the generic matrix was uploaded, data were lg2(x) transformed, and only proteins with five valid values were included in the further analysis. Missing values were replaced by using Impute LCMD and q (MinDet) = 0.01, and data from this matrix are shown in a principal component analysis (PCA) with components 2 (13.5%) and 5 (5.9%) to show clustering dependent on less pronounced components. Subsequently, a two-way ANOVA was performed, and results were Bonferroni corrected. All proteins with significantly different expressions (*q*-value < 0.05) were filtered and analyzed with the gene set enrichment analysis web tool EnrichR (https://maayanlab.cloud/Enrichr/, accessed on 15 January 2022). 

## 5. Conclusions

In conclusion, the present study demonstrates that HFD-induced maternal obesity causes an obese body composition and hepatic growth restriction in the male offspring in early life. As depicted in our working model (Figure 7) this impaired liver growth pattern in male offspring of obese dams might be mechanistically linked to (1) an increased DDR, (2) hepatocellular senescence, and (3) inhibition of insulin/IGF-1-mediated AKT-pFoxO1 signaling, resulting in signs of a possibly inadequate compensatory pro-proliferative growth response. (4) An inflammatory egWAT-liver axis with a hepatic activation of the IL-6/STAT3 signaling might be central in the inflamm-aging of the liver after perinatal obesity in rodents. Based on these findings we speculate that perinatal obesity in mice might induce an early onset of aging-related processes, resulting in premature liver aging and hepatic growth restriction in male offspring that may ultimately increase the susceptibility to chronic liver disease later in life.

## Data Availability

The data presented in this study are available on request from the corresponding author.

## Supplementary Materials

The following supporting information can be downloaded at: https://www.mdpi.com/article/10.3390/ijms23105609/s1.

## References

[1] Abarca-Gómez L., Abdeen Z., Hamid Z., Abu-Rmeileh N.M., Acosta-Cazares B., Acuin C., Adams R.J., Aekplakorn W., Afsana K., Aguilar-Salinas C.A. (2017). Worldwide trends in body-mass index, underweight, overweight, and obesity from 1975 to 2016: A pooled analysis of 2416 population-based measurement studies in 128·9 million children, adolescents, and adults. Lancet.

[2] Blüher M. (2019). Obesity: Global epidemiology and pathogenesis. Nat. Rev. Endocrinol..

[3] Poston L., Caleyachetty R., Cnattingius S., Corvalán C., Uauy R., Herring S., Gillman M.W. (2016). Preconceptional and maternal obesity: Epidemiology and health consequences. Lancet Diabetes Endocrinol..

[4] Heslehurst N., Rankin J., Wilkinson J.R., Summerbell C.D. (2010). A nationally representative study of maternal obesity in England, UK: Trends in incidence and demographic inequalities in 619 323 births 1989. Int. J. Obes..

[5] Catalano P.M., Shankar K. (2017). Obesity and pregnancy: Mechanisms of short term and long term adverse consequences for mother and child. BMJ.

[6] Butler E.A., Cohen E., Berger H., Ray J.G. (2021). Change in Pre-Pregnancy Body Mass Index in Relation to COVID-19 Pandemic. J. Obs. Gynaecol. Can..

[7] Godfrey K., Reynolds R., Prescott S., Nyirenda M., Jaddoe V.W., Eriksson J.G., Broekman B.F. (2017). Influence of maternal obesity on the long-term health of offspring. Lancet Diabetes Endocrinol..

[8] Shrestha N., Ezechukwu H.C., Holland O.J., Hryciw D.H. (2020). Developmental programming of peripheral diseases in offspring exposed to maternal obesity during pregnancy. Am. J. Physiol. Regul. Integr. Comp. Physiol..

[9] Schoonejans J.M., Ozanne S.E. (2021). Developmental programming by maternal obesity: Lessons from animal models. Diabet. Med..

[10] Barker D.J.P. (2007). The origins of the developmental origins theory. J. Intern. Med..

[11] Kuiper-Makris C., Selle J., Nüsken E., Dötsch J., Alejandre Alcazar M.A. (2021). Perinatal Nutritional and Metabolic Pathways: Early Origins of Chronic Lung Diseases. Front. Med..

[12] Lumbers E.R., Kandasamy Y., Delforce S.J., Boyce A.C., Gibson K.J., Pringle K.G. (2020). Programming of Renal Development and Chronic Disease in Adult Life. Front. Physiol..

[13] Wesolowski S., El Kasmi K., Jonscher K., Friedman J. (2017). Developmental origins of NAFLD: A womb with a clue. Nat. Rev. Gastroenterol. Hepatol..

[14] Scheja L., Heeren J. (2019). The endocrine function of adipose tissues in health and cardiometabolic disease. Nat. Rev. Endocrinol..

[15] Mau T., Yung R. (2018). Adipose tissue inflammation in aging. Exp. Gerontol..

[16] Kasper P., Breuer S., Hoffmann T., Vohlen C., Janoschek R., Schmitz L., Appel S., Fink G., Hünseler C., Quaas A. (2021). Maternal Exercise Mediates Hepatic Metabolic Programming via Activation of AMPK-PGC1α Axis in the Offspring of Obese Mothers. Cells.

[17] Yakar S., Adamo M.L. (2012). Insulin-like growth factor 1 physiology: Lessons from mouse models. Endocrinol. Metab. Clin. North. Am..

[18] Adamek A., Kasprzak A. (2018). Insulin-Like Growth Factor (IGF) System in Liver Diseases. Int. J. Mol. Sci..

[19] Kasper P., Vohlen C., Dinger K., Mohr J., Hucklenbruch-Rother E., Janoschek R., Köth J., Matthes J., Appel S., Dötsch J. (2017). Renal metabolic programming is linked to the dynamic regulation of a Leptin-Klf15 axis and Akt/AMPKα signaling in male offspring of obese dams. Endocrinology.

[20] Aguirre G.A., De Ita J.R., de la Garza R.G., Castilla-Cortazar I. (2016). Insulin-like growth factor-1 deficiency and metabolic syndrome. J. Transl. Med..

[21] Schmidt-Arras D., Rose-John S. (2016). IL-6 pathway in the liver: From physiopathology to therapy. J. Hepatol..

[22] Varela-Rey M., Beraza N., Lu S.C., Mato J.M., Martínez-Chantar M.L. (2011). Role of AMP-activated protein kinase in the control of hepatocyte priming and proliferation during liver regeneration. Exp. Biol. Med..

[23] Kim I.H., Kisseleva T., Brenner D.A. (2015). Aging and liver disease. Curr. Opin. Gastroenterol..

[24] Sharma A., Singh K., Almasan A. (2012). Histone H2AX phosphorylation: A marker for DNA damage. Methods Mol. Biol..

[25] Wang C., Jurk D., Maddick M., Nelson G., Martin-Ruiz C., von Zglinicki T. (2009). DNA damage response and cellular senescence in tissues of aging mice. Aging Cell.

[26] Pestell R.G. (2013). New roles of cyclin D1. Am. J. Pathol..

[27] Sun B., Karin M. (2008). NF-kappaB signaling, liver disease and hepatoprotective agents. Oncogene.

[28] Chen Y., Liu X., Wang H., Liu S., Hu N., Li X. (2019). Akt Regulated Phosphorylation of GSK-3β/Cyclin D1, p21 and p27 Contributes to Cell Proliferation Through Cell Cycle Progression From G1 to S/G2M Phase in Low-Dose Arsenite Exposed HaCat Cells. Front. Pharmacol..

[29] Böhm F., Köhler U.A., Speicher T., Werner S. (2010). Regulation of liver regeneration by growth factors and cytokines. EMBO Mol. Med..

[30] Moh A., Iwamoto Y., Chai G.-X., Zhang S.S.M., Kano A., Yang D.D., Zhang W., Wang J., Jacoby J.J., Gao B. (2007). Role of STAT3 in liver regeneration: Survival, DNA synthesis, inflammatory reaction and liver mass recovery. Lab. Investig..

[31] Dinger K., Koningsbruggen-Rietschel S.V., Dötsch J., Alejandre Alcazar M.A. (2020). Identification of Critical Windows of Metabolic Programming of Metabolism and Lung Function in Male Offspring of Obese Dams. Clin. Transl. Sci..

[32] Zhao H.-M., Zhang X.-Y., Lu X.-Y., Yu S.R., Wang X., Zou Y., Zuo Z.Y., Liu D.Y., Zhou B.G. (2018). Erzhi Pill(^®^) Protected Experimental Liver Injury Against Apoptosis via the PI3K/Akt/Raptor/Rictor Pathway. Front. Pharmacol..

[33] Mortensen O.H., Olsen H.L., Frandsen L., Nielsen P.E., Nielsen F.C., Grunnet N., Quistorff B. (2010). Gestational protein restriction in mice has pronounced effects on gene expression in newborn offspring’s liver and skeletal muscle; protective effect of taurine. Pediatr. Res..

[34] Howell K.R., Powell T.L. (2017). Effects of maternal obesity on placental function and fetal development. Reproduction.

[35] Lewandowska M. (2021). Maternal Obesity and Risk of Low Birth Weight, Fetal Growth Restriction, and Macrosomia: Multiple Analyses. Nutrients.

[36] Guzzardi M.A., Liistro T., Gargani L., Ait Ali L., D’Angelo G., Rocchiccioli S., La Rosa F., Kemeny A., Sanguinetti E., Ucciferri N. (2018). Maternal Obesity and Cardiac Development in the Offspring: Study in Human Neonates and Minipigs. JACC Cardiovasc. Imaging.

[37] Moritz K.M., Wintour E.M., Black M.J., Bertram J.F., Caruana G. (2008). Factors influencing mammalian kidney development: Implications for health in adult life. Adv. Anat. Embryol. Cell Biol..

[38] Lessard-Beaudoin M., Laroche M., Demers M.-J., Grenier G., Graham R.K. (2015). Characterization of age-associated changes in peripheral organ and brain region weights in C57BL/6 mice. Exp. Gerontol..

[39] Dollé M.E.T., Kuiper R.V., Roodbergen M., Robinson J., de Vlugt S., Wijnhoven S.W., Beems R.B., de la Fonteyne L., de With P., van der Pluijm I. (2011). Broad segmental progeroid changes in short-lived Ercc1(-/Δ7) mice. Pathobiol. Aging Age Relat. Dis..

[40] White R.R., Milholland B., de Bruin A., Curran S., Laberge R.M., Van Steeg H., Campisi J., Maslov A.Y., Vijg J. (2015). Controlled induction of DNA double-strand breaks in the mouse liver induces features of tissue ageing. Nat. Commun..

[41] Włodarczyk M., Nowicka G. (2019). Obesity, DNA Damage, and Development of Obesity-Related Diseases. Int. J. Mol. Sci..

[42] Bankoglu E.E., Tschopp O., Schmitt J., Burkard P., Jahn D., Geier A., Stopper H. (2016). Role of PTEN in Oxidative Stress and DNA Damage in the Liver of Whole-Body Pten Haplodeficient Mice. PLoS ONE.

[43] Di Micco R., Krizhanovsky V., Baker D., d’Adda di Fagagna F. (2021). Cellular senescence in ageing: From mechanisms to therapeutic opportunities. Nat. Rev. Mol. Cell Biol..

[44] Ferreira-Gonzalez S., Rodrigo-Torres D., Gadd V.L., Forbes S.J. (2021). Cellular Senescence in Liver Disease and Regeneration. Semin. Liver Dis..

[45] Engelmann C., Tacke F. (2022). The Potential Role of Cellular Senescence in Non-Alcoholic Fatty Liver Disease. Int. J. Mol. Sci..

[46] Ogrodnik M., Miwa S., Tchkonia T., Tiniakos D., Wilson C.L., Lahat A., Day C.P., Burt A., Palmer A., Anstee Q.M. (2017). Cellular senescence drives age-dependent hepatic steatosis. Nat. Commun..

[47] Sell C. (2015). Minireview: The Complexities of IGF/Insulin Signaling in Aging: Why Flies and Worms Are Not Humans. Mol. Endocrinol..

[48] Beyer T.A., Xu W., Teupser D., Auf dem Keller U., Bugnon P., Hildt E., Thiery J., Kan Y.W., Werner S. (2008). Impaired liver regeneration in Nrf2 knockout mice: Role of ROS-mediated insulin/IGF-1 resistance. EMBO J..

[49] Desbois-Mouthon C., Wendum D., Cadoret A., Rey C., Leneuve P., Blaise A., Housset C., Tronche F., Le Bouc Y., Holzenberger M. (2006). Hepatocyte proliferation during liver regeneration is impaired in mice with liver-specific IGF-1R knockout. FASEB J..

[50] Morales-Garza L.A., Puche J.E., Aguirre G.A., Muñoz Ú., García-Magariño M., De la Garza R.G., Castilla-Cortazar I. (2017). Experimental approach to IGF-1 therapy in CCl(4)-induced acute liver damage in healthy controls and mice with partial IGF-1 deficiency. J. Transl. Med..

[51] Tosh D.N., Fu Q., Callaway C.W., McKnight R.A., McMillen I.C., Ross M.G., Lane R.H., Desai M. (2010). Epigenetics of programmed obesity: Alteration in IUGR rat hepatic IGF1 mRNA expression and histone structure in rapid vs. delayed postnatal catch-up growth. Am. J. Physiol. Gastrointest. Liver Physiol..

[52] O’Connor J.C., McCusker R.H., Strle K., Johnson R.W., Dantzer R., Kelley K.W. (2008). Regulation of IGF-I function by proinflammatory cytokines: At the interface of immunology and endocrinology. Cell Immunol..

[53] Al-Mansoori L., Al-Jaber H., Prince M.S., Elrayess M.A. (2022). Role of Inflammatory Cytokines, Growth Factors and Adipokines in Adipogenesis and Insulin Resistance. Inflammation.

[54] Dinger K., Kasper P., Hucklenbruch-Rother E., Vohlen C., Jobst E., Janoschek R., Bae-Gartz I., van Koningsbruggen-Rietschel S., Plank C., Dötsch J. (2016). Early-onset obesity dysregulates pulmonary adipocytokine/insulin signaling and induces asthma-like disease in mice. Sci. Rep..

[55] Litzenburger T., Huber E.-K., Dinger K., Wilke R., Vohlen C., Selle J., Kadah M., Persigehl T., Heneweer C., Dötsch J. (2020). Maternal high-fat diet induces long-term obesity with sex-dependent metabolic programming of adipocyte differentiation, hypertrophy and dysfunction in the offspring. Clin. Sci..

[56] Bae-Gartz I., Kasper P., Großmann N., Breuer S., Janoschek R., Kretschmer T., Appel S., Schmitz L., Vohlen C., Quaas A. (2020). Maternal exercise conveys protection against NAFLD in the offspring via hepatic metabolic programming. Sci. Rep..

[57] Bruce K.D., Cagampang F.R., Argenton M., Zhang J., Ethirajan P.L., Burdge G.C., Bateman A.C., Clough G.F., Poston L., Hanson M.A. (2009). Maternal high-fat feeding primes steatohepatitis in adult mice offspring, involving mitochondrial dysfunction and altered lipogenesis gene expression. Hepatology.

[58] Breuer S., Kasper P., Vohlen C., Janoschek R., Hoffmann T., Appel S., Müller-Limberger E., Mesaros A., Rose-John S., Garbers C. (2021). Brain-Restricted Inhibition of IL-6 Trans-Signaling Mildly Affects Metabolic Consequences of Maternal Obesity in Male Offspring. Nutrients.

[59] Demichev V., Messner C.B., Vernardis S.I., Lilley K.S., Ralser M. (2020). DIA-NN: Neural networks and interference correction enable deep proteome coverage in high throughput. Nat. Methods.

[60] Hirani D., Alvira C.M., Danopoulos S., Milla C., Donato M., Tian L., Mohr J., Dinger K., Vohlen C., Selle J. (2021). Macrophage-derived IL-6 trans-signaling as a novel target in the pathogenesis of bronchopulmonary dysplasia. Eur. Respir. J..

