# Supplementary material for: Perinatal Obesity Induces Hepatic Growth Restriction with Increased DNA Damage Response, Senescence, and Dysregulated Igf-1-Akt-Foxo1 Signaling in Male Offspring of Obese Mice
Source: Int J Mol Sci. 2022 May 17;23(10):5609. doi: 10.3390/ijms23105609 (PMC9144113; doi:10.3390/ijms23105609)
Supplement: Supplementary file 1 [file ijms-23-05609-s001.zip › ijms-1706913-supplementary.pdf]

Supplementary Material

Title of manuscript: Perinatal obesity induces hepatic growth restriction with increased DNA damage response, senescence and dysregulated IGF-1-AKT-FoxO1 signaling in male offspring of obese mice

Authors: Philipp Kasper, Jaco Selle, Christina Vohlen, Rebecca Wilke, Celien Kuiper-Makris, Oleksiy Klymenko, Inga-Bae-Gartz, Charlotte Schömig, Alexander Quaas, Björn Schumacher, Münevver Demir, Martin Bürger, Sonja Lang, Anna Martin, Hans-Michael Steffen, Tobias Goeser, Jörg Doetsch and Miguel A. Alejandre Alcazar

List of contents:

1. Supplementary Table S1 (Experimental diets)
2. Supplementary Table S2 (Primer list, real time RT-PCR)
3. Supplementary Table S3 (Antibody list, immunoblots)
4. Supplementary Table S4 (Proteomic analysis; Overview of all identified proteins)
5. Supplementary Table S5 (Proteomic analysis; Overview of all significantly altered pathways in the offspring by maternal diet)

1. Supplementary Table S1

Information on crude nutrients and energy density of the experimental diets:

			SD	HFD
Name			**R/M-Maintenance**	**C1057, modified**
Company			Ssniff	Altromin
Order number			V1534 -000	10005791
Additional information			complete feed for rats & mice	high fat diet
Metab. Energy		kcal/kg	3225	5237
Metab. Energy		MJ/kg	13,5	21,9
Fat		kJ%	9	60
Protein		kJ%	24	16
Carbohydrates		kJ%	67	24
Sugar		kJ%	8,8	7,0
Crude Fat		g/kg	19,00	20,80
Crude Protein		g/kg	3,30	35,10
Crude Fibre		g/kg	5,00	0,39
Crude Ash		g/kg	6,40	6,10
N free extracts		g/kg	41,3	41,7
**Sugar**				
Sucrose		g/kg	190,0	208,0
Monosaccharides		g/kg	33,0	351,0
Dissacharides		g/kg	413,0	417,0
Sugar (total)		g/kg	54,0	121,0

2. Supplementary Table S2

List of primers used for real-time RT-PCR:

Gene	Primer	Primer sequence 5‘-3‘
Bax<br>(Bcl-2-associated X protein)	for<br>rev<br>Probe (FAM, TAMRA)	TGCTGATGGCAACTTCAACTG<br>TTTAGTGCACAGGGCCTTGAG<br>CCGCGTGGTTGCCCTCTTCTACTTTG
Bcl2<br>(B-cell lymphoma 2)	for<br>rev<br>Probe (FAM, TAMRA)	GCATCACTCTGGGTGCATACC<br>GCTTTAGTGAACCTTTTGCATATTTG<br>CCACAAGTGAGGTCGACAAACCTGCC
Col1a1<br>(Collagen type I alpha 1)	for<br>rev	GCAGTGCTGTTGCGATCTTG<br>CAGAGGGACAGAGCACAGCTT
Ccnd1<br>(Cyclin D1)	for<br>rev<br>Probe (FAM, TAMRA)	CGCCCTCCGTATCTTACTTCAA<br>CTCACAGACCTCCAGCATCCA<br>CCATGCGGAAAATCGTGGCCAC
Ccnd2<br>(Cyclin D2)	for<br>rev<br>Probe (FAM, TAMRA)	CAGCAGGATGATGAAGTGAACAC<br>GGCTTTGAGACAATCCACATCAG<br>CTCACGTGTGATGCCCTGACAGAGC
Gapdh	for<br>rev<br>Probe (FAM, TAMRA)	ATGTGTCCGTCGTGGATCTGA<br>TGCCTGCTTCACCACCTTCT<br>CCGCCTGGAGAAACCTGCCAAGTATG
Il6<br>(Interleukin 6)	for<br>rev<br>Probe (FAM, TAMRA)	ACAAGTCGGAGGCTTAATTACACAT<br>AATCAGAATTGCCATTGCCCAA<br>TCTTTTCTCATTTCCACGATTTCCCAGAGAA
Igf-1[1]<br>(Insulin-like growth factor 1)	for<br>rev	GCTGGTGGATGCTCTTCAGTT<br>GGTGCCCTCCGAATGCT

Igfr[1]<br><br>(Insulin-like growth factor 1 receptor)	for<br><br>rev	GCTTCCTGTGAAAGTGATGTTCTC<br><br>CGTTTTTAAATGGTGCCTCC
Igfbp1[1]<br><br>(Insulin-like growth factor-binding protein 1)	for<br><br>rev	CAGGGAGCCTGTGTACCAGAA<br><br>GCAGCCTTTGCCTCTTCATG
Igfbp2[1]<br><br>(Insulin-like growth factor-binding protein 2)	for<br><br>rev	GCCCCCTGGAACATCTCTACT<br><br>AGAGACATCTTGCACTGCTTAAGGT
Igfbp3[1]<br><br>(Insulin-like growth factor-binding protein 3)	for<br><br>rev	CCAGAACTTCTCCTCCGAGTCTAA<br><br>GATGATTCAGTGTGTCCTCCATTT
Il1b[1]<br><br>(Interleukin 1 beta)	for<br><br>rev	TGACAGTGATGAGAATGACCTGTTC<br><br>GGACAGCCCAGGTCAAAGG
Insr[1]<br><br>(Insulin receptor)	for<br><br>rev	GCAAACAGATGCCACTAATCCTT<br><br>GGGCTTCCACTTTAAGATAATCTGA
Irs-1[1]<br><br>(Insulin Receptor Substrate 1)	for<br><br>rev	CCAGAGTCAAGCCTCACACA<br><br>CCCAACTCAACTCCACCACT
Ki67	for<br><br>rev<br><br>Probe (FAM, TAMRA)	AAATCATGGGCTGATGTTGTAAAA<br><br>TTGCTTGTGGGTTTCTTTGGA<br><br>AAACATGTCCCTCAAAAGCAGACGAGCAA
Leptin	for<br><br>rev<br><br>Probe (FAM, TAMRA)	TCACCAGGATCAATGACATTTCAC<br><br>AGCCCAGGAATGAAGTCCAA<br><br>ACGCAGTCGGTATCCGCCAAGC
Mcp-1<br><br>(Monocyte chemoattractant protein 1)	for<br><br>rev<br><br>Probe (FAM, TAMRA)	GGCTCAGCCAGATGCAGTTAAC<br><br>CTTGGTGACAAAAACTACAGCTTCTT<br><br>CCCCACTCACCTGCTGCTACTCATTCA
Nfkb1[1]<br><br>(Nuclear Factor Kappa B Subunit 1)	for<br><br>rev	CATCCCGGAGTCACGAAATC<br><br>GCACAATCTTTAGGGCCATTTT
Nfkb2[1]<br><br>(Nuclear Factor Kappa B Subunit 2)	for<br><br>rev	TCTAGCCACAGAGATGGAGGAGTT<br><br>AGGTCCGGGCATTCACATTA

p16[1]<br><br>(Cyclin-dependent kinase inhibitor 2A)	for<br><br>rev	CAAGACATCGTGCGATATTTGC<br><br>GGGCGTGCTTGAGCTGAA
p27<br><br>(Cyclin-dependent kinase inhibitor 1B)	for<br><br>rev<br><br>Probe (FAM, TAMRA)	CAAAAGGGCCAACAGAACAGA<br><br>CGAAGGCCGGGCTTCTT<br><br>AAAATGTTTCAGACGGTTCCCCGAACG
P53	for<br><br>rev<br><br>Probe (FAM, TAMRA)	CAGCGTGGTGGTACCTTATGAG<br><br>GGTTCCCACTGGAGTCTTCCA<br><br>AACCGCCGACCTATCCTTACCATCATCA
Pcna<br><br>(Proliferating cell nuclear antigen)	for<br><br>rev<br><br>Probe (FAM, TAMRA)	GGAGGCGGTAACCATAGAGATG<br><br>ACAGTGGAGTGGCTTTTGTGAA<br><br>ATGAGCCTGTTCACCTAACGTTTGCTCTGA
Ppargc1a<br><br>(Peroxisome proliferator-activated receptor gamma coactivator 1-alpha,PGC-1α)	for<br><br>rev<br><br>Probe (FAM, TAMRA)	TCGAAAAAGAAGTCCCATACACAA<br><br>TTCCACACTTAAGGTTCGCTCAATA<br><br>CACCAAATGACCCCAAGGGTTCCC
Tfam<br><br>(Mitochondrial transcription factor A)	for<br><br>rev<br><br>Probe (FAM, TAMRA)	GGGAATGTGGAGCGTGCTAA<br><br>GGATAGCTACCCATGCTGGAAA<br><br>CGCATCCCCTCGTCTATCAGTCTTGTCTGTA
Timp1<br><br>(TIMP metallopeptidase inhibitor 1)	for<br><br>rev<br><br>Probe (FAM, TAMRA)	ACCTGGTCATAAGGGCTAAATTCA<br><br>CCGGATATCTGCGGCATT<br><br>TTCCCCAGAAATCAACGAGACCACCTT
Tnfa[1]<br><br>(Tumor necrosis factor-alpha)	for<br><br>rev	AGGGATGAGAAGTTCCCAAATG<br><br>GCTTGTCACTCGAATTTTGAGAAG

[1]Genes were measured by the SYBR-Green method. No probe was needed.

Abbreviations: FAM, 6-carboxyfluorescein; TAMRA, tetramethylrhodamine.

3. Supplementary Table S3

Blots were probed with the following antibodies:

**Primary antibody**	**Description**	**Purchased from**
phospho-AMPK-α (Thr172)	Monoclonal rabbit anti–phosphorylated AMPKα, 1:<br><br>1000	Cell Signaling, Danvers, MA, USA; catalog no. 2535
AMPK-α<br><br>(AMP-activated protein kinase alpha subunit)	Monoclonal rabbit<br><br>anti-total AMPKα, 1:2000	Cell Signaling, Danvers,MA, USA; catalog no. 2603
phospho-AKT (Ser473)	Monoclonal rabbit<br><br>anti-phosphorylated AKT; 1:1000	Cell Signaling, Danvers, MA, USA; catalog no. 4058
AKT	Monocloncal rabbit<br><br>anti-total AKT, 1:2000	Cell Signaling, Danvers, MA, USA; catalog no. 9272
Insulin receptor β	Monocloncal rabbit<br><br>anti-InsR, 1:1000	Cell Signaling, Danvers, MA, USA, catalog no. 3025
Insulin-like grwoth factor 1 (IGF-1) receptor type 1	Monocloncal rabbit<br><br>anti-InsR, 1:1000	Cell Signaling, Danvers, MA, USA, catalog no. 3027
Proliferating cell nuclear antigen (PCNA)	Monocloncal mouse<br><br>anti PCNA, 1:5000	Aligent Dako, Santa Clara, CA, USA, catalog no. M0879
phospho-STAT3 (Tyr705),	Monocloncal rabbit<br><br>anti-phospho-STAT3, 1:1000	Cell Signaling, Danvers, MA, catalog no. 9145
STAT-3	Monoclonal mouse<br><br>anti- total STAT3, 1:2000	Cell Signaling, Danvers, MA, catalog no. 9139
p21<br><br>(Cyclin Dependent Kinase Inhibitor 1A)	Monoclonal mouse<br><br>anti-total p21, 1:200	Invitrogen, Waltham, MA, USA, catalog no. MA5-31479
p gamma H2A.X (γH2A.X Ser139)	Monoclonal rabbit<br><br>Anti- gamma H2A.X, 1:400	Abcam, Cambridge, Great Britain, catalog no. ab11174
Phospho p38 (Thr180/Tyr182)	Monoclonal rabbit	Cell Signaling, Danvers, MA, USA, catalog no. 4511

	anti-phospho p38, 1:1000	
p38	Monoclonal rabbit<br><br>anti-total p38, 1:2000	Cell Signaling, Danvers, MA, USA, catalog no. 9212
p53	Monoconal rabbit<br><br>anti- p53, 1:1000	Thermo Fisher Scientific, Waltham, MA, catalog no. RB-9006-PO
phospho-NF-κB p65<br><br>(Ser536)	Monoconal rabbit<br><br>anti phospho-NF-κB p65, 1:1000	Cell Signaling, Danvers, MA, USA, catalog no. 3033
NF-κB p65	Monoconal rabbit<br><br>anti NF-κB p65, 1:1000	Cell Signaling, Danvers, MA, USA, catalog no. 8242
β-Actin<br><br>(Loadin	Monoclonal mouse anti ß-actin 1:5000) served as a loading control.	Cell Signaling, Danvers, MA, USA, catalog no. 3700
Secondary antibody:		
Anti-rabbit IgG (horseradish peroxidase–linked;<br><br>Cell Signaling Technology,Danvers, MA, USA catatolg no. 7074) was used as secondary antibody.		

4. Supplemental Table S4

List of all identified proteins in the proteomic screen at P21

No.	Protein	Gene	Description	-Log Student's T-test p-value	Difference_Log2(fold-change)
1	AMY1_MOUSE	Amy1	Alpha-amylase 1	4,60922	-2,62907
2	IGF1_MOUSE	Igf1	Insulin-like growth factor I	4,71989	3,22718
3	TRY2_MOUSE	Prss2	Anionic trypsin-2	3,21568	-5,00773
4	PRG2_MOUSE	Prg2	Bone marrow proteoglycan	4,76846	2,69596
5	CP7A1_MOUSE	Cyp7a1	Cytochrome P450 7A1	2,70063	-2,70129
6	UPP2_MOUSE	Upp2	Uridine phosphorylase 2	7,75122	-5,49209
7	ATTY_MOUSE	Tat	Tyrosine aminotransferase	3,58592	-2,13026
8	CSAD_MOUSE	Csad	Cysteine sulfinic acid decarboxylase	5,00377	1,9675
9	LPP60_MOUSE	Aspg	60 kDa lysophospholipase	0,285613	-0,283841
10	DHX8_MOUSE	Dhx8	ATP-dependent RNA helicase DHX8	0,595396	-0,720249
11	BIG2_MOUSE	Arfgef2	Brefeldin A-inhibited guanine nucleotide-exchange protein 2	0,287579	-0,301965
12	SMUF2_MOUSE	Smurf2	E3 ubiquitin-protein ligase SMURF2	0,354756	-0,176146
13	K1522_MOUSE	Kiaa1522	Uncharacterized protein KIAA1522	0,361409	0,345546
14	PTPRF_MOUSE	Ptprf	Receptor-type tyrosine-protein phosphatase F	0,0663575	-0,0588051
15	OSBL9_MOUSE	Osbpl9	Oxysterol-binding protein-related protein 9	0,29872	-0,187315
16	DDI2_MOUSE	Ddi2	Protein DDI1 homolog 2	0,614041	0,295199
17	CKAP5_MOUSE	Ckap5	Cytoskeleton-associated protein 5	0,641486	0,381702
18	MA1B1_MOUSE	Man1b1	Endoplasmic reticulum mannosyl-oligosaccharide 1,2-alpha-mannosidase	0,468532	0,319627
19	MA7D1_MOUSE	Map7d1	MAP7 domain-containing protein 1	0,532241	0,501106
20	FGGY_MOUSE	Fggy	FGGY carbohydrate kinase domain-containing protein	0,428773	0,355222
21	ACNT1_MOUSE	Acnat1	Acyl-coenzyme A amino acid N-acyltransferase 1	0,572816	0,415279
22	SHRM2_MOUSE	Shroom2	Protein Shroom2	1,07217	0,93689
23	UBR4_MOUSE	Ubr4	E3 ubiquitin-protein ligase UBR4	0,0527886	-0,0338684
24	SKT_MOUSE	Skt	Sickle tail protein	0,590844	0,304812
25	SPEB_MOUSE	Agmat	Agmatinase, mitochondrial	0,413942	0,145953
26	AGRIN_MOUSE	Agrn	Agrin	1,02882	-0,738444
27	SCMC2_MOUSE	Slc25a25	Calcium-binding mitochondrial carrier protein SCaMC-2	0,558562	0,160556
28	DHTK1_MOUSE	Dhtkd1	Probable 2-oxoglutarate dehydrogenase E1	0,610939	-0,408257
29	RBGP1_MOUSE	Rabgap1	Rab GTPase-activating protein 1	0,112617	-0,101908
30	ARGAL_MOUSE	Arhgef10l	Rho guanine nucleotide exchange factor 10-like protein	0,34321	0,400511
31	MOCS3_MOUSE	Mocs3	Adenylyltransferase and sulfurtransferase MOCS3	0,856445	0,614841
32	ARI1A_MOUSE	Arid1a	AT-rich interactive domain-containing protein 1A	1,62218	0,482361
33	UH1BL_MOUSE	Uhrf1bp1l	UHRF1-binding protein 1-like	0,451001	0,29528
34	DEN5B_MOUSE	Dennd5b	DENN domain-containing protein 5B	0,140328	-0,0727348
35	TRM1L_MOUSE	Trmt1l	TRMT1-like protein	0,915706	0,253371
36	RGPA2_MOUSE	Ralgapa2	Ral GTPase-activating protein subunit alpha-2	0,802658	0,504702
37	TTC38_MOUSE	Ttc38	Tetratricopeptide repeat protein 38	0,0166576	-0,00790367
38	METH_MOUSE	Mtr	Methionine synthase	0,448913	0,298833
39	MIPEP_MOUSE	Mipep	Mitochondrial intermediate peptidase	0,177195	0,0934769
40	DEN4C_MOUSE	Dennd4c	DENN domain-containing protein 4C	0,452429	0,290934
41	D19L1_MOUSE	Dpy19l1	Probable C-mannosyltransferase DPY19L1	0,24776	-0,440895
42	ITIH4_MOUSE	Itih4	Inter alpha-trypsin inhibitor, heavy chain 4	0,673345	0,254615
43	EKI2_MOUSE	Etnk2	Ethanolamine kinase 2	0,34736	-0,462049
44	CISD3_MOUSE	Cisd3	CDGSH iron-sulfur domain-containing protein 3, mitochondrial	0,332825	-0,151203
45	UBP24_MOUSE	Usp24	Ubiquitin carboxyl-terminal hydrolase 24	0,008969	0,00802765
46	THOC2_MOUSE	Thoc2	THO complex subunit 2	0,236507	0,248735
47	PYR1_MOUSE	Cad	CAD protein	0,948332	0,477317

48	YTDC2_MOUSE	Ythdc2	3'-5' RNA helicase YTHDC2	0,622057	0,501745
49	OTUD4_MOUSE	Otud4	OTU domain-containing protein 4	1,00013	0,386871
50	OTU7B_MOUSE	Otud7b	OTU domain-containing protein 7B	0,314819	0,264807
51	TPC11_MOUSE	Trappc11	Trafficking protein particle complex subunit 11	0,922459	-0,576794
52	PLXB2_MOUSE	Plxnb2	Plexin-B2	1,41314	0,420562
53	RBM25_MOUSE	Rbm25	RNA-binding protein 25	0,152457	0,0614315
54	XPP3_MOUSE	Xpnpep3	Xaa-Pro aminopeptidase 3	1,2638	0,562651
55	ITA9_MOUSE	Itga9	Integrin alpha-9	0,235551	-0,12433
56	DAAF5_MOUSE	Dnaaf5	Dynein assembly factor 5, axonemal	0,873623	0,470064
57	MFA1A_MOUSE;MF	Mfap1a;Mfap1b	Microfibrillar-associated protein 1A	0,140742	-0,11334
58	H2A1B_MOUSE;H2/	H2afj;Hist1h2ab;I	Histone H2A type 1-B	0,203148	-0,153691
59	RNT2A_MOUSE;RN	Rnaset2a;Rnaset	Ribonuclease T2-A	0,545506	-0,228217
60	SAFB1_MOUSE	Safb	Scaffold attachment factor B1	0,338537	0,178955
61	CCDC6_MOUSE	Ccdc6	Coiled-coil domain-containing protein 6	0,231941	0,212493
62	BIN2_MOUSE	Bin2	Bridging integrator 2	0,804084	0,676822
63	FIBA_MOUSE	Fga	Fibrinogen alpha chain	0,844199	0,57374
64	GCN1_MOUSE	Gcn1	eIF-2-alpha kinase activator GCN1	0,0868579	0,0532825
65	SET1A_MOUSE	Setd1a	Histone-lysine N-methyltransferase SETD1A	0,13402	-0,15428
66	PARP4_MOUSE	Parp4	Protein mono-ADP-ribosyltransferase PARP4	1,25288	0,64562
67	ASCC3_MOUSE	Ascc3	Activating signal cointegrator 1 complex subunit 3	0,0657224	-0,0371323
68	CHD2_MOUSE	Chd2	Chromodomain-helicase-DNA-binding protein 2	0,931649	-0,340173
69	AKP13_MOUSE	Akap13	A-kinase anchor protein 13	0,812926	0,217504
70	C2CD2_MOUSE	C2cd2	C2 domain-containing protein 2	0,550768	-0,238201
71	APOB_MOUSE	Apob	Apolipoprotein B-100	0,671131	0,345029
72	ARI1B_MOUSE	Arid1b	AT-rich interactive domain-containing protein 1B	0,601797	0,363103
73	RN213_MOUSE	Rnf213	E3 ubiquitin-protein ligase RNF213	0,609496	0,355637
74	DESP_MOUSE	Dsp	Desmoplakin	1,41863	0,580641
75	NOLC1_MOUSE	Nolc1	Nucleolar and coiled-body phosphoprotein 1	0,694026	0,424923
76	MYO1E_MOUSE	Myo1e	Unconventional myosin-Ie	1,1889	0,451371
77	BD1L1_MOUSE	Bod1l	Biorientation of chromosomes in cell division protein 1-like 1	0,0250165	0,0453171
78	NUMA1_MOUSE	Numa1	Nuclear mitotic apparatus protein 1	0,444495	0,166167
79	DJC21_MOUSE	Dnajc21	DnaJ homolog subfamily C member 21	0,222688	0,287266
80	HELZ2_MOUSE	Helz2	Helicase with zinc finger domain 2	0,611103	0,61557
81	SC16A_MOUSE	Sec16a	Protein transport protein Sec16A	1,32155	0,491949
82	TPR_MOUSE	Tpr	Nucleoprotein TPR	0,604038	0,113947
83	FARP1_MOUSE	Farp1	FERM, ARHGEF and pleckstrin domain-containing protein 1	0,0610052	-0,0392525
84	AOXC_MOUSE	Aox3	Aldehyde oxidase 3	0,221333	0,235994
85	AT2B1_MOUSE	Atp2b1	Plasma membrane calcium-transporting ATPase 1	0,21828	0,140683
86	TRIPC_MOUSE	Trip12	E3 ubiquitin-protein ligase TRIP12	0,184381	0,0965187
87	ANK3_MOUSE	Ank3	Ankyrin-3	0,0657122	-0,169185
88	AGRF5_MOUSE	Adgrf5	Adhesion G protein-coupled receptor F5	0,00280246	0,00340843
89	CAN2_MOUSE	Capn2	Calpain-2 catalytic subunit	0,83452	0,227911
90	BIN1_MOUSE	Bin1	Myc box-dependent-interacting protein 1	0,38815	0,56845
91	SC22B_MOUSE	Sec22b	Vesicle-trafficking protein SEC22b	0,521456	0,343167
92	DPYL2_MOUSE	Dpysl2	Dihydropyrimidinase-related protein 2	0,306012	0,253717
93	LEG9_MOUSE	Lgals9	Galectin-9	0,0578642	0,033865
94	EMD_MOUSE	Emd	Emerin	0,62912	0,176661
95	GTPB1_MOUSE	Gtpbp1	GTP-binding protein 1	0,946933	-0,308851
96	THOC4_MOUSE	Alyref	THO complex subunit 4	0,728815	0,240137
97	CLCA_MOUSE	Clta	Clathrin light chain A	1,05496	-0,441913
98	PTEN_MOUSE	Pten	Phosphatidylinositol 3,4,5-trisphosphate 3-phosphatase	1,77741	1,34702
99	STXB1_MOUSE	Stxbp1	Syntaxin-binding protein 1	0,851513	0,535283

100	NUCG_MOUSE	Endog	Endonuclease G, mitochondrial	0,0847508	-0,0427937
101	MTP_MOUSE	Mttp	Microsomal triglyceride transfer protein large subunit	0,0297534	0,0395218
102	MYH11_MOUSE	Myh11	Myosin-11	0,230917	-0,416191
103	MAP2_MOUSE	Metap2	Methionine aminopeptidase 2	0,5402	0,233154
104	KNG1_MOUSE	Kng1	Kininogen-1	0,419282	0,277584
105	NGP_MOUSE	Ngp	Neutrophilic granule protein	2,29134	2,24039
106	PRDX6_MOUSE	Prdx6	Peroxiredoxin-6	0,118853	-0,0853111
107	AKAP1_MOUSE	Akap1	A-kinase anchor protein 1, mitochondrial	0,739109	0,729539
108	CASP6_MOUSE	Casp6	Caspase-6	0,171774	-0,0876549
109	DLDH_MOUSE	Dld	Dihydrolipoyl dehydrogenase, mitochondrial	0,622681	0,266712
110	HNF6_MOUSE	Onecut1	Hepatocyte nuclear factor 6	0,0776316	0,105769
111	HCD2_MOUSE	Hsd17b10	3-hydroxyacyl-CoA dehydrogenase type-2	0,996915	-0,300369
112	UBE3A_MOUSE	Ube3a	Ubiquitin-protein ligase E3A	0,161176	0,0837391
113	TCOF_MOUSE	Tcof1	Treacle protein	0,179453	0,400183
114	DCTN1_MOUSE	Dctn1	Dynactin subunit 1	0,346939	0,166074
115	GLU2B_MOUSE	Prkcsh	Glucosidase 2 subunit beta	0,927503	0,348358
116	PRDX4_MOUSE	Prdx4	Peroxiredoxin-4	1,99483	0,498704
117	DIAP1_MOUSE	Diaph1	Protein diaphanous homolog 1	1,92654	0,33991
118	U5S1_MOUSE	Eftud2	116 kDa U5 small nuclear ribonucleoprotein component	0,104412	0,122163
119	ELL_MOUSE	Ell	RNA polymerase II elongation factor ELL	0,000490143	-0,000828934
120	FAAH1_MOUSE	Faah	Fatty-acid amide hydrolase 1	1,10297	0,664925
121	AIP_MOUSE	Aip	AH receptor-interacting protein	0,99556	0,408405
122	FLOT1_MOUSE	Flot1	Flotillin-1	1,36763	0,579185
123	ATOX1_MOUSE	Atox1	Copper transport protein ATOX1	0,44546	-1,18076
124	PEX5_MOUSE	Pex5	Peroxisomal targeting signal 1 receptor	1,09626	0,423758
125	SNP23_MOUSE	Snap23	Synaptosomal-associated protein 23	0,390484	0,195586
126	PSB1_MOUSE	Psmb1	Proteasome subunit beta type-1	0,485544	0,229786
127	HDAC1_MOUSE	Hdac1	Histone deacetylase 1	0,703647	0,346618
128	MP2K3_MOUSE	Map2k3	Dual specificity mitogen-activated protein kinase kinase 3	0,065951	-0,0333408
129	GSTO1_MOUSE	Gsto1	Glutathione S-transferase omega-1	0,271592	0,203801
130	CP3AP_MOUSE	Cyp3a25	Cytochrome P450 3A25	1,47109	-1,09434
131	MA2B1_MOUSE	Man2b1	Lysosomal alpha-mannosidase	0,136756	0,0789223
132	SODE_MOUSE	Sod3	Extracellular superoxide dismutase [Cu-Zn]	0,292067	0,304996
133	RL21_MOUSE	Rpl21	60S ribosomal protein L21	0,622288	0,257167
134	GSH0_MOUSE	Gclm	Glutamate--cysteine ligase regulatory subunit	0,0510829	-0,0265686
135	HGD_MOUSE	Hgd	Homogentisate 1,2-dioxygenase	0,465434	-0,285865
136	AMACR_MOUSE	Amacr	Alpha-methylacyl-CoA racemase	0,733268	0,208795
137	KIF1C_MOUSE	Kif1c	Kinesin-like protein KIF1C	1,417	0,57209
138	VP26C_MOUSE	Vps26c	Vacuolar protein sorting-associated protein 26C	0,193682	0,0708817
139	SCRB2_MOUSE	Scarb2	Lysosome membrane protein 2	1,64833	0,664616
140	ATN1_MOUSE	Atn1	Atrophin-1	1,71796	2,35218
141	C10_MOUSE	Grcc10	Protein C10	2,15525	0,794867
142	PHB2_MOUSE	Phb2	Prohibitin-2	0,47442	-0,117195
143	ATIF1_MOUSE	ATP5IF1	ATPase inhibitor, mitochondrial	0,539956	-0,522665
144	COFA1_MOUSE	Col15a1	Collagen alpha-1(XV) chain	0,130525	-0,222908
145	DOPD_MOUSE	Ddt	D-dopachrome decarboxylase	0,0620254	0,0861275
146	CPSF2_MOUSE	Cpsf2	Cleavage and polyadenylation specificity factor subunit 2	0,0940448	-0,0281258
147	PSMD4_MOUSE	Psmd4	26S proteasome non-ATPase regulatory subunit 4	1,57914	0,549744
148	EXOC7_MOUSE	Exoc7	Exocyst complex component 7	1,34048	0,500913
149	DHX15_MOUSE	Dhx15	Pre-mRNA-splicing factor ATP-dependent RNA helicase DHX15	0,492614	0,118484
150	PURB_MOUSE	Purb	Transcriptional activator protein Pur-beta	0,0538433	0,025993
151	NMI_MOUSE	Nmi	N-myc-interactor	0,270107	0,0830948

152	SRSF5_MOUSE	Srsf5	Serine/arginine-rich splicing factor 5	0,645563	-0,284154
153	IMA7_MOUSE	Kpna6	Importin subunit alpha-7	0,229396	0,110966
154	CAN1_MOUSE	Capn1	Calpain-1 catalytic subunit	0,726588	0,49407
155	AN32A_MOUSE	Anp32a	Acidic leucine-rich nuclear phosphoprotein 32 family member A	1,81961	-0,600511
156	EXOC4_MOUSE	Exoc4	Exocyst complex component 4	0,975999	0,175736
157	PAHX_MOUSE	Phyh	Phytanoyl-CoA dioxygenase, peroxisomal	0,0189555	-0,0174824
158	HAX1_MOUSE	Hax1	HCLS1-associated protein X-1	1,25739	0,605725
159	PLD3_MOUSE	Pld3	Phospholipase D3	0,148402	0,0706394
160	SPYA_MOUSE	Agxt	Serine--pyruvate aminotransferase, mitochondrial	1,067	-0,609003
161	PYRD_MOUSE	Dhodh	Dihydroorotate dehydrogenase (quinone), mitochondrial	0,807668	0,339437
162	ECH1_MOUSE	Ech1	Delta(3,5)-Delta(2,4)-dienoyl-CoA isomerase, mitochondrial	2,47266	0,730622
163	FKBP8_MOUSE	Fkbp8	Peptidyl-prolyl cis-trans isomerase FKBP8	0,507549	0,26951
164	S27A2_MOUSE	Slc27a2	Very long-chain acyl-CoA synthetase	1,14052	0,583918
165	BHMT1_MOUSE	Bhmt	Betaine--homocysteine S-methyltransferase 1	1,43163	-0,592636
166	NOTC2_MOUSE	Notch2	Neurogenic locus notch homolog protein 2	0,554076	0,232595
167	RABE1_MOUSE	Rabep1	Rab GTPase-binding effector protein 1	0,595665	0,300748
168	PSDE_MOUSE	Psmd14	26S proteasome non-ATPase regulatory subunit 14	0,165146	0,132202
169	SCAM3_MOUSE	Scamp3	Secretory carrier-associated membrane protein 3	0,80635	0,396841
170	HYAL2_MOUSE	Hyal2	Hyaluronidase-2	0,520008	0,368804
171	STAG2_MOUSE	Stag2	Cohesin subunit SA-2	1,32548	-0,782625
172	ANXA3_MOUSE	Anxa3	Annexin A3	0,163383	-0,0991489
173	AP1B1_MOUSE	Ap1b1	AP-1 complex subunit beta-1	0,633548	0,153428
174	NEUR1_MOUSE	Neu1	Sialidase-1	0,894771	1,16505
175	GSTM6_MOUSE	Gstm6	Glutathione S-transferase Mu 6	0,0473786	-0,0602032
176	MGLL_MOUSE	Mgll	Monoglyceride lipase	1,44289	0,776797
177	MYADM_MOUSE	Myadm	Myeloid-associated differentiation marker	0,609703	-0,325126
178	NDUA1_MOUSE	Ndufa1	NADH dehydrogenase [ubiquinone] 1 alpha subcomplex subunit 1	0,34003	0,258551
179	NUDC_MOUSE	Nudc	Nuclear migration protein nudC	0,215857	0,131532
180	PININ_MOUSE	Pnn	Pinin	0,0211206	-0,0107788
181	SPTC1_MOUSE	Sptlc1	Serine palmitoyltransferase 1	0,0589543	0,0727692
182	LIPB2_MOUSE	Ppfibp2	Liprin-beta-2	0,0334622	-0,0343353
183	CP4AE_MOUSE	Cyp4a14	Cytochrome P450 4A14	1,17128	1,25349
184	HNRH1_MOUSE	Hnrnph1	Heterogeneous nuclear ribonucleoprotein H	0,2877	0,345877
185	API5_MOUSE	Api5	Apoptosis inhibitor 5	0,0285975	-0,0170265
186	TIM44_MOUSE	Timm44	Mitochondrial import inner membrane translocase subunit TIM44	0,921262	-0,245686
187	CSN5_MOUSE	Cops5	COP9 signalosome complex subunit 5	0,14605	-0,239699
188	CALU_MOUSE	Calu	Calumenin	0,898731	0,189204
189	SP100_MOUSE	Sp100	Nuclear autoantigen Sp-100	1,12438	1,09208
190	LSM2_MOUSE	Lsm2	U6 snRNA-associated Sm-like protein LSm2	0,00658273	0,00799789
191	FRDA_MOUSE	Fxn	Frataxin, mitochondrial	1,64134	-1,61768
192	AL1A7_MOUSE	Aldh1a7	Aldehyde dehydrogenase, cytosolic 1	0,348079	0,366859
193	PSB10_MOUSE	Psmb10	Proteasome subunit beta type-10	0,231796	0,210806
194	GAMT_MOUSE	Gamt	Guanidinoacetate N-methyltransferase	0,275755	0,195629
195	RM23_MOUSE	Mrpl23	39S ribosomal protein L23, mitochondrial	0,0922597	0,0430111
196	ZW10_MOUSE	Zw10	Centromere/kinetochore protein zw10 homolog	0,251885	0,274789
197	CAVN1_MOUSE	Cavin1	Caveolae-associated protein 1	0,0808717	-0,118099
198	OST48_MOUSE	Ddost	Dolichyl-diphosphooligosaccharide--protein glycosyltransferase	0,482857	0,143827
199	CP2J5_MOUSE	Cyp2j5	Cytochrome P450 2J5	0,601324	0,347379
200	CP2J6_MOUSE	Cyp2j6	Cytochrome P450 2J6	0,79532	-0,524625
201	AOXA_MOUSE	Aox1	Aldehyde oxidase 1	0,426847	0,563046
202	AP3D1_MOUSE	Ap3d1	AP-3 complex subunit delta-1	0,762975	0,380339
203	MA2B2_MOUSE	Man2b2	Epididymis-specific alpha-mannosidase	1,06104	0,26106

204	CHKA_MOUSE	Chka	Choline kinase alpha	1,60001	1,2239
205	IL16_MOUSE	Il16	Pro-interleukin-16	0,207406	0,272547
206	BYST_MOUSE	Bysl	Bystin	1,92336	0,526053
207	CSK22_MOUSE	Csnk2a2	Casein kinase II subunit alpha'	1,40221	0,390972
208	ITB3_MOUSE	Itgb3	Integrin beta-3	0,41571	-0,752239
209	RDH16_MOUSE	Rdh16	Retinol dehydrogenase 16	0,476729	-0,414701
210	REPS1_MOUSE	Reps1	RalBP1-associated Eps domain-containing protein 1	1,73913	0,521913
211	AKAP2_MOUSE	Akap2	A-kinase anchor protein 2	0,56734	0,416685
212	SMCE1_MOUSE	Smarce1	SWI/SNF-related matrix-associated actin-dependent regulator	1,77135	0,618299
213	DNJB6_MOUSE	Dnajb6	DnaJ homolog subfamily B member 6	0,0376434	-0,0253307
214	BAF_MOUSE	Banf1	Barrier-to-autointegration factor	0,420434	-0,27768
215	RNF13_MOUSE	Rnf13	E3 ubiquitin-protein ligase RNF13	0,262308	0,281155
216	ASNA_MOUSE	Asna1	ATPase Asna1	0,589848	0,147019
217	SLK_MOUSE	Slk	STE20-like serine/threonine-protein kinase	0,133597	-0,119568
218	BNIP3_MOUSE	Bnip3	BCL2/adenovirus E1B 19 kDa protein-interacting protein 3	2,68239	-1,93775
219	TPPC3_MOUSE	Trappc3	Trafficking protein particle complex subunit 3	0,233344	0,193909
220	PGRC1_MOUSE	Pgrmc1	Membrane-associated progesterone receptor component 1	0,901061	0,383833
221	IMPA1_MOUSE	Impa1	Inositol monophosphatase 1	1,60424	0,406408
222	BCKD_MOUSE	Bckdk	[3-methyl-2-oxobutanoate dehydrogenase	0,484407	-0,421981
223	COPB2_MOUSE	Copb2	Coatomer subunit beta'	0,666915	0,166441
224	SYUA_MOUSE	Snca	Alpha-synuclein	0,420099	-0,355709
225	TPMT_MOUSE	Tpmt	Thiopurine S-methyltransferase	0,999615	-0,573256
226	BL1S1_MOUSE	Bloc1s1	Biogenesis of lysosome-related organelles complex 1 subunit 1	0,255068	0,233672
227	STRN_MOUSE	Strn	Striatin	0,7488	0,568821
228	DSG2_MOUSE	Dsg2	Desmoglein-2	1,79118	0,489574
229	NIPS1_MOUSE	Nipsnap1	Protein NipSnap homolog 1	0,232449	0,175307
230	NIPS2_MOUSE	Nipsnap2	Protein NipSnap homolog 2	0,792702	-0,591191
231	SAP18_MOUSE	Sap18	Histone deacetylase complex subunit SAP18	0,316397	0,157799
232	SEPT7_MOUSE	Septin7	Septin-7	0,623842	0,314635
233	IF6_MOUSE	Eif6	Eukaryotic translation initiation factor 6	1,13165	0,473828
234	RL35A_MOUSE	Rpl35a	60S ribosomal protein L35a	0,80804	0,453584
235	AT2A2_MOUSE	Atp2a2	Sarcoplasmic/endoplasmic reticulum calcium ATPase 2	0,325177	-0,264781
236	SPT5H_MOUSE	Supt5h	Transcription elongation factor SPT5	1,57849	0,589685
237	ILK_MOUSE	Ilk	Integrin-linked protein kinase	0,294439	-0,297627
238	CHKB_MOUSE	Chkb	Choline/ethanolamine kinase	1,16598	0,37384
239	PSB5_MOUSE	Psmb5	Proteasome subunit beta type-5	0,34515	0,157587
240	DHX9_MOUSE	Dhx9	ATP-dependent RNA helicase A	0,179607	0,0928577
241	DPM1_MOUSE	Dpm1	Dolichol-phosphate mannosyltransferase subunit 1	0,039703	-0,0455898
242	PI42A_MOUSE	Pip4k2a	Phosphatidylinositol 5-phosphate 4-kinase type-2 alpha	0,422964	0,388544
243	EIF3D_MOUSE	Eif3d	Eukaryotic translation initiation factor 3 subunit D	0,535524	0,163395
244	AIF1_MOUSE	Aif1	Allograft inflammatory factor 1	0,0996384	-0,146147
245	EF1B_MOUSE	Eef1b	Elongation factor 1-beta	1,56498	0,279131
246	HMOX2_MOUSE	Hmox2	Heme oxygenase 2	0,173613	-0,0777317
247	ATX2_MOUSE	Atxn2	Ataxin-2	1,74438	0,44291
248	NMT1_MOUSE	Nmt1	Glycylpeptide N-tetradecanoyltransferase 1	0,591871	-0,410671
249	NMT2_MOUSE	Nmt2	Glycylpeptide N-tetradecanoyltransferase 2	0,144325	-0,12873
250	E41L2_MOUSE	Epb41l2	Band 4.1-like protein 2	1,12911	0,618185
251	GPX4_MOUSE	Gpx4	Phospholipid hydroperoxide glutathione peroxidase	0,284521	0,414174
252	PHLD_MOUSE	Gpld1	Phosphatidylinositol-glycan-specific phospholipase D	0,00481734	0,00360641
253	EMC8_MOUSE	Emc8	ER membrane protein complex subunit 8	1,31529	0,45994
254	PDLI1_MOUSE	Pdlim1	PDZ and LIM domain protein 1	0,355886	-0,530625
255	VAMP8_MOUSE	Vamp8	Vesicle-associated membrane protein 8	0,896311	-0,326622

256	PSA3_MOUSE	Psma3	Proteasome subunit alpha type-3	0,118296	-0,137832
257	UGDH_MOUSE	Ugdh	UDP-glucose 6-dehydrogenase	0,373346	0,319511
258	VAMP4_MOUSE	Vamp4	Vesicle-associated membrane protein 4	0,374898	-0,515882
259	SNX3_MOUSE	Snx3	Sorting nexin-3	0,797801	0,287064
260	SNX12_MOUSE	Snx12	Sorting nexin-12	0,291345	0,234904
261	DHB12_MOUSE	Hsd17b12	Very-long-chain 3-oxoacyl-CoA reductase	0,27571	0,270237
262	SRPK1_MOUSE	Srpk1	SRSF protein kinase 1	0,186433	0,118496
263	PIGR_MOUSE	Pigr	Polymeric immunoglobulin receptor	0,0369606	0,0669176
264	DTNB_MOUSE	Dtnb	Dystrobrevin beta	0,0920602	-0,121644
265	CSKP_MOUSE	Cask	Peripheral plasma membrane protein CASK	0,234645	0,0822594
266	PFD2_MOUSE	Pfdn2	Prefoldin subunit 2	0,54745	0,369526
267	CFDP1_MOUSE	Cfdp1	Craniofacial development protein 1	0,105505	-0,176973
268	ZN326_MOUSE	Znf326	DBIRD complex subunit ZNF326	0,0291628	0,0233345
269	NID2_MOUSE	Nid2	Nidogen-2	0,116445	0,132233
270	WDR1_MOUSE	Wdr1	WD repeat-containing protein 1	1,31013	0,314067
271	IKKB_MOUSE	Ikbkb	Inhibitor of nuclear factor kappa-B kinase subunit beta	2,4728	0,406231
272	VTI1B_MOUSE	Vti1b	Vesicle transport through interaction with t-SNAREs homolog 1B	1,98611	0,552102
273	PAPS2_MOUSE	Papss2	Bifunctional 3'-phosphoadenosine 5'-phosphosulfate synthase 2	0,325289	-0,226628
274	KLC1_MOUSE	Klc1	Kinesin light chain 1	0,509257	0,398492
275	RDH7_MOUSE	Rdh7	Retinol dehydrogenase 7	0,245568	0,107156
276	CPNS1_MOUSE	Capns1	Calpain small subunit 1	0,300015	-0,249646
277	DC1I2_MOUSE	Dync1i2	Cytoplasmic dynein 1 intermediate chain 2	0,678128	0,460245
278	PPT1_MOUSE	Ppt1	Palmitoyl-protein thioesterase 1	0,186044	0,136077
279	ZFR_MOUSE	Zfr	Zinc finger RNA-binding protein	0,594322	0,236528
280	DDC_MOUSE	Ddc	Aromatic-L-amino-acid decarboxylase	0,730191	0,344307
281	CSN3_MOUSE	Cops3	COP9 signalosome complex subunit 3	0,749668	-0,178307
282	CSN4_MOUSE	Cops4	COP9 signalosome complex subunit 4	0,511797	0,192035
283	CSN6_MOUSE	Cops6	COP9 signalosome complex subunit 6	0,349896	-0,262082
284	ROA2_MOUSE	Hnrnpa2b1	Heterogeneous nuclear ribonucleoproteins A2/B1	1,3231	0,549076
285	COMT_MOUSE	Comt	Catechol O-methyltransferase	0,0123681	0,0185928
286	BECN1_MOUSE	Becn1	Beclin-1	0,922562	-0,591751
287	CREG1_MOUSE	Creg1	Protein CREG1	0,297818	0,233323
288	PRS6A_MOUSE	Psmc3	26S proteasome regulatory subunit 6A	1,69661	0,323216
289	CLPP_MOUSE	Clpp	ATP-dependent Clp protease proteolytic subunit, mitochondrial	0,859392	0,37013
290	CTBP1_MOUSE	Ctbp1	C-terminal-binding protein 1	0,544221	0,14812
291	MAP7_MOUSE	Map7	Ensconsin	1,28186	0,333576
292	BIRC6_MOUSE	Birc6	Baculoviral IAP repeat-containing protein 6	1,9307	0,570615
293	TOM1_MOUSE	Tom1	Target of Myb protein 1	0,704129	0,453687
294	FA5_MOUSE	F5	Coagulation factor V	0,160899	0,145419
295	STAM2_MOUSE	Stam2	Signal transducing adapter molecule 2	0,870311	-1,06877
296	CP4AA_MOUSE	Cyp4a10	Cytochrome P450 4A10	0,0305973	0,0482555
297	IDHC_MOUSE	Idh1	Isocitrate dehydrogenase [NADP] cytoplasmic	1,51754	1,41566
298	AKA10_MOUSE	Akap10	A-kinase anchor protein 10, mitochondrial	0,75502	0,738417
299	DHRS3_MOUSE	Dhrs3	Short-chain dehydrogenase/reductase 3	0,333034	0,258724
300	SOAT2_MOUSE	Soat2	Sterol O-acyltransferase 2	0,068544	-0,0522312
301	FA10_MOUSE	F10	Coagulation factor X	0,0122332	-0,00927505
302	LIN7C_MOUSE	Lin7c	Protein lin-7 homolog C	1,15428	0,411602
303	GNPI1_MOUSE	Gnpda1	Glucosamine-6-phosphate isomerase 1	0,144861	-0,170126
304	CP8B1_MOUSE	Cyp8b1	7-alpha-hydroxycholest-4-en-3-one 12-alpha-hydroxylase	0,761419	0,426362
305	YMEL1_MOUSE	Yme1l1	ATP-dependent zinc metalloprotease YME1L1	0,0512781	-0,0129868
306	KBL_MOUSE	Gcat	2-amino-3-ketobutyrate coenzyme A ligase, mitochondrial	0,234272	-0,0861458
307	CBPD_MOUSE	Cpd	Carboxypeptidase D	0,860177	0,315047

308	LGMN_MOUSE	Lgmn	Legumain	0,00635797	0,00311203
309	AFAM_MOUSE	Afm	Afamin	0,520211	0,44669
310	COR1A_MOUSE	Coro1a	Coronin-1A	1,07286	0,568177
311	COPE_MOUSE	Cope	Coatomer subunit epsilon	1,76981	0,584388
312	RBM3_MOUSE	Rbm3	RNA-binding protein 3	0,0168756	0,0100666
313	SP1_MOUSE	Sp1	Transcription factor Sp1	1,16813	0,777503
314	FHIT_MOUSE	Fhit	Bis(5'-adenosyl)-triphosphatase	1,12442	-0,387112
315	CASP8_MOUSE	Casp8	Caspase-8	2,14758	0,71021
316	CP1A2_MOUSE	Cyp1a2	Cytochrome P450 1A2	0,730604	-0,977103
317	ADH1_MOUSE	Adh1	Alcohol dehydrogenase 1	0,791585	-0,343983
318	DYR_MOUSE	Dhfr	Dihydrofolate reductase	0,405738	0,455161
319	COX2_MOUSE	Mtco2	Cytochrome c oxidase subunit 2	1,18022	-0,512659
320	HPRT_MOUSE	Hprt1	Hypoxanthine-guanine phosphoribosyltransferase	1,37514	0,363833
321	CAH2_MOUSE	Ca2	Carbonic anhydrase 2	0,0535996	-0,0527161
322	CO3_MOUSE	C3	Complement C3	0,193978	0,204689
323	CO4B_MOUSE	C4b	Complement C4-B	1,15343	0,485162
324	B2MG_MOUSE	B2m	Beta-2-microglobulin	0,781282	0,831074
325	HA10_MOUSE	H2-Q10	H-2 class I histocompatibility antigen, Q10 alpha chain	0,237568	0,300004
326	HA11_MOUSE	H2-D1	H-2 class I histocompatibility antigen, D-B alpha chain	0,0611498	-0,0550507
327	HA1B_MOUSE	H2-K1	H-2 class I histocompatibility antigen, K-B alpha chain	0,669757	0,411671
328	HBA_MOUSE	Hba	Hemoglobin subunit alpha	0,605001	-0,334325
329	HBB1_MOUSE	Hbb-b1	Hemoglobin subunit beta-1	1,03589	-0,885406
330	CO4A1_MOUSE	Col4a1	Collagen alpha-1(IV) chain	0,285399	-0,24525
331	LAMC1_MOUSE	Lamc1	Laminin subunit gamma-1	0,314731	-0,186521
332	LAMB1_MOUSE	Lamb1	Laminin subunit beta-1	0,792183	-0,420607
333	ATP8_MOUSE	Mtatp8	ATP synthase protein 8	0,797817	-0,599809
334	ADA_MOUSE	Ada	Adenosine deaminase	0,517204	-0,316713
335	FABP4_MOUSE	Fabp4	Fatty acid-binding protein, adipocyte	0,575208	0,392391
336	CFAB_MOUSE	Cfb	Complement factor B	1,88702	0,715646
337	MYG_MOUSE	Mb	Myoglobin	0,206218	-0,601238
338	ARAF_MOUSE	Araf	Serine/threonine-protein kinase A-Raf	0,465792	0,163539
339	B3AT_MOUSE	Slc4a1	Band 3 anion transport protein	0,183211	0,158434
340	ALDOC_MOUSE	Aldoc	Fructose-bisphosphate aldolase C	0,082255	0,0956272
341	ALDOA_MOUSE	Aldoa	Fructose-bisphosphate aldolase A	0,0974963	0,104233
342	KAPCA_MOUSE	Prkaca	cAMP-dependent protein kinase catalytic subunit alpha	0,0447888	-0,0290707
343	AATC_MOUSE	Got1	Aspartate aminotransferase, cytoplasmic	1,06194	-0,416196
344	AATM_MOUSE	Got2	Aspartate aminotransferase, mitochondrial	0,292087	0,0975868
345	K1C18_MOUSE	Krt18	Keratin, type I cytoskeletal 18	0,364459	-0,584444
346	LDHA_MOUSE	Ldha	L-lactate dehydrogenase A chain	0,128175	0,129628
347	CO5_MOUSE	C5	Complement C5	0,0823178	-0,17929
348	APOA4_MOUSE	Apoa4	Apolipoprotein A-IV	1,79801	1,53501
349	G6PI_MOUSE	Gpi	Glucose-6-phosphate isomerase	0,514073	0,337784
350	CATL1_MOUSE	Ctsl	Cathepsin L1	0,473744	-0,403151
351	MAOX_MOUSE	Me1	NADP-dependent malic enzyme	1,50148	1,36618
352	ENPP1_MOUSE	Enpp1	Ectonucleotide pyrophosphatase/phosphodiesterase family member 1	0,487132	-0,473989
353	CFAH_MOUSE	Cfh	Complement factor H	0,991557	0,462323
354	SPRC_MOUSE	Sparc	SPARC	0,744061	-0,862325
355	TTHY_MOUSE	Ttr	Transthyretin	0,217941	0,191072
356	KCRM_MOUSE	Ckm	Creatine kinase M-type	0,343307	-0,899456
357	ANXA2_MOUSE	Anxa2	Annexin A2	0,366808	-0,243013
358	ALBU_MOUSE	Alb	Serum albumin	0,30532	0,277277
359	RIR1_MOUSE	Rrm1	Ribonucleoside-diphosphate reductase large subunit	0,740999	0,37032

360	A1AT1_MOUSE	Serpina1a	Alpha-1-antitrypsin 1-1	0,312975	0,223091
361	HS90A_MOUSE	Hsp90aa1	Heat shock protein HSP 90-alpha	0,804188	0,320671
362	PDIA4_MOUSE	Pdia4	Protein disulfide-isomerase A4	4,16185	0,688692
363	SPTA1_MOUSE	Spta1	Spectrin alpha chain, erythrocytic 1	0,0354505	0,0313622
364	ENPL_MOUSE	Hsp90b1	Endoplasmin	0,455929	0,243634
365	APOE_MOUSE	Apoe	Apolipoprotein E	1,35214	0,76574
366	SODC_MOUSE	Sod1	Superoxide dismutase [Cu-Zn]	0,489855	0,177618
367	MDHM_MOUSE	Mdh2	Malate dehydrogenase, mitochondrial	0,645442	-0,179473
368	C4BPA_MOUSE	C4bpa	C4b-binding protein	1,17321	0,486435
369	GNAI2_MOUSE	Gnai2	Guanine nucleotide-binding protein G(i) subunit alpha-2	0,264746	-0,146893
370	RPB1_MOUSE	Polr2a	DNA-directed RNA polymerase II subunit RPB1	0,442213	0,287287
371	ITB1_MOUSE	Itgb1	Integrin beta-1	0,615055	0,203053
372	PDIA1_MOUSE	P4hb	Protein disulfide-isomerase	1,23657	0,491418
373	NUCL_MOUSE	Ncl	Nucleolin	0,183209	-0,0824104
374	PGK1_MOUSE	Pgk1	Phosphoglycerate kinase 1	0,0485321	0,062534
375	FRIH_MOUSE	Fth1	Ferritin heavy chain	0,31342	0,5626
376	CSF1R_MOUSE	Csf1r	Macrophage colony-stimulating factor 1 receptor	1,4056	0,373219
377	SODM_MOUSE	Sod2	Superoxide dismutase [Mn], mitochondrial	0,031238	-0,0247768
378	CADH1_MOUSE	Cdh1	Cadherin-1	0,0912819	0,0783531
379	APOA2_MOUSE	Apoa2	Apolipoprotein A-II	0,128508	0,113832
380	SURF1_MOUSE	Surf1	Surfeit locus protein 1	0,233689	-0,194342
381	H2AV_MOUSE;H2AZ	H2afv;H2afz	Histone H2A.Z	0,0164103	0,00753899
382	KGP1_MOUSE	Prkg1	cGMP-dependent protein kinase 1	0,329169	0,154096
383	DERPC_MOUSE	Derpc	Decreased expression in renal and prostate cancer protein	1,28961	1,29632
384	NDUB1_MOUSE	Ndufb1	NADH dehydrogenase [ubiquinone] 1 beta subcomplex subunit 1	0,349192	0,130403
385	IFI4_MOUSE	Ifi204	Interferon-activable protein 204	0,499879	0,396895
386	CALM1_MOUSE;CA	Calm1;Calm2;Ca	Calmodulin-1	0,404625	-0,4207
387	ANXA1_MOUSE	Anxa1	Annexin A1	0,599063	0,34142
388	EF1A1_MOUSE	Eef1a1	Elongation factor 1-alpha 1	0,103157	0,145184
389	ENV1_MOUSE		MLV-related proviral Env polyprotein	1,81222	1,50723
390	NID1_MOUSE	Nid1	Nidogen-1	0,202503	0,171417
391	HEM2_MOUSE	Alad	Delta-aminolevulinic acid dehydratase	0,346017	0,424314
392	CATB_MOUSE	Ctsb	Cathepsin B	0,129506	-0,0548973
393	IF4A2_MOUSE	Eif4a2	Eukaryotic initiation factor 4A-II	0,936613	-1,28363
394	THIO_MOUSE	Txn	Thioredoxin	0,521592	0,161278
395	GSTA2_MOUSE	Gsta2	Glutathione S-transferase A2	0,765281	-0,998146
396	GSTM1_MOUSE	Gstm1	Glutathione S-transferase Mu 1	0,0667977	0,0876278
397	TCEA1_MOUSE	Tcea1	Transcription elongation factor A protein 1	0,835724	0,271922
398	4F2_MOUSE	Slc3a2	4F2 cell-surface antigen heavy chain	1,63348	0,643377
399	H2B1C_MOUSE;H2B	Hist1h2bc;Hist1h	Histone H2B type 1-M	0,194028	0,793594
400	H10_MOUSE	H1f0	Histone H1.0	0,831333	-0,178568
401	TCP4_MOUSE	Sub1	Activated RNA polymerase II transcriptional coactivator p15	0,429201	-0,328524
402	GRAA_MOUSE	Gzma	Granzyme A	1,52002	-0,858032
403	CO1A1_MOUSE	Col1a1	Collagen alpha-1(I) chain	0,339001	0,579707
404	PARP1_MOUSE	Parp1	Poly [ADP-ribose] polymerase 1	0,663798	-0,541944
405	LIPL_MOUSE	Lpl	Lipoprotein lipase	0,744061	0,678962
406	FINC_MOUSE	Fn1	Fibronectin	0,456311	0,320708
407	GPX1_MOUSE	Gpx1	Glutathione peroxidase 1	0,331326	-0,198779
408	FABPH_MOUSE	Fabp3	Fatty acid-binding protein, heart	0,335191	-1,7118
409	LAMP1_MOUSE	Lamp1	Lysosome-associated membrane glycoprotein 1	0,438359	0,22222
410	HS90B_MOUSE	Hsp90ab1	Heat shock protein HSP 90-beta	0,412448	0,185541
411	DMD_MOUSE	Dmd	Dystrophin	0,297751	0,071957

412	CD1D1_MOUSE	Cd1d1	Antigen-presenting glycoprotein CD1d1	0,18799	0,133424
413	K2C8_MOUSE	Krt8	Keratin, type II cytoskeletal 8	2,27559	-0,439433
414	PROP_MOUSE	Cfp	Properdin	1,13538	0,735957
415	ITA5_MOUSE	Itga5	Integrin alpha-5	1,98587	0,601036
416	CP2D9_MOUSE	Cyp2d9	Cytochrome P450 2D9	0,286314	-0,725552
417	OTC_MOUSE	Otc	Ornithine carbamoyltransferase, mitochondrial	0,0455344	-0,0269707
418	ITB2_MOUSE	Itgb2	Integrin beta-2	0,368947	0,262451
419	GAS2_MOUSE	Gas2	Growth arrest-specific protein 2	0,079741	0,144096
420	ITPR1_MOUSE	Itpr1	Inositol 1,4,5-trisphosphate receptor type 1	0,816051	0,548442
421	NUD19_MOUSE	Nudt19	Nucleoside diphosphate-linked moiety X motif 19	0,97686	0,586165
422	TCPA_MOUSE	Tcp1	T-complex protein 1 subunit alpha	0,93084	0,445995
423	SAMP_MOUSE	Apcs	Serum amyloid P-component	0,498102	-0,781419
424	BGLR_MOUSE	Gusb	Beta-glucuronidase	0,51694	-0,808931
425	KAP2_MOUSE	Prkar2a	cAMP-dependent protein kinase type II-alpha regulatory subunit	0,487053	0,420385
426	PFKAL_MOUSE	Pfkl	ATP-dependent 6-phosphofructokinase, liver type	1,12228	-0,336861
427	FABPL_MOUSE	Fabp1	Fatty acid-binding protein, liver	2,17476	0,631281
428	COX5A_MOUSE	Cox5a	Cytochrome c oxidase subunit 5A, mitochondrial	0,247224	0,172781
429	CP2B9_MOUSE	Cyp2b9	Cytochrome P450 2B9	0,898269	-0,646226
430	PDCD6_MOUSE	Pdcd6	Programmed cell death protein 6	0,870654	-1,08286
431	RL7A_MOUSE	Rpl7a	60S ribosomal protein L7a	0,859084	-0,715966
432	GELS_MOUSE	Gsn	Gelsolin	0,0451445	0,055798
433	UMPS_MOUSE	Umps	Uridine 5'-monophosphate synthase	0,129311	0,0920895
434	ICAM1_MOUSE	Icam1	Intercellular adhesion molecule 1	0,0221127	-0,025132
435	CAH1_MOUSE	Ca1	Carbonic anhydrase 1	0,303243	-0,307631
436	GPDA_MOUSE	Gpd1	Glycerol-3-phosphate dehydrogenase [NAD(+)], cytoplasmic	0,372603	-0,265236
437	AT1B1_MOUSE	Atp1b1	Sodium/potassium-transporting ATPase subunit beta-1	0,857671	-0,636382
438	RL27A_MOUSE	Rpl27a	60S ribosomal protein L27a	0,593873	0,136023
439	RS16_MOUSE	Rps16	40S ribosomal protein S16	0,796152	0,426965
440	RL7_MOUSE	Rpl7	60S ribosomal protein L7	0,482616	0,331941
441	MDHC_MOUSE	Mdh1	Malate dehydrogenase, cytoplasmic	0,27069	-0,275351
442	RSSA_MOUSE	Rpsa	40S ribosomal protein SA	0,0855767	0,0663097
443	CALR_MOUSE	Calr	Calreticulin	0,472721	0,186427
444	GTR2_MOUSE	Slc2a2	Solute carrier family 2, facilitated glucose transporter member 2	0,250491	-0,309864
445	SRP54_MOUSE	Srp54	Signal recognition particle 54 kDa protein	1,51258	0,460307
446	PSMD3_MOUSE	Psmd3	26S proteasome non-ATPase regulatory subunit 3	0,477202	-0,295277
447	LMNB1_MOUSE	Lmnb1	Lamin-B1	0,172894	-0,0878059
448	ANXA6_MOUSE	Anxa6	Annexin A6	0,360702	0,243259
449	RLA0_MOUSE	Rplp0	60S acidic ribosomal protein P0	0,143234	-0,0447327
450	HMOX1_MOUSE	Hmox1	Heme oxygenase 1	1,29359	0,71044
451	GLNA_MOUSE	Glul	Glutamine synthetase	0,293575	0,405193
452	CADH2_MOUSE	Cdh2	Cadherin-2	0,249613	-0,0984657
453	INSR_MOUSE	Insr	Insulin receptor	1,43883	-0,664775
454	REL_MOUSE	Rel	Proto-oncogene c-Rel	0,594978	0,357901
455	PMGE_MOUSE	Bpgm	Bisphosphoglycerate mutase	0,189214	0,195515
456	CD44_MOUSE	Cd44	CD44 antigen	2,08344	2,36966
457	CP2A4_MOUSE	Cyp2a4	Cytochrome P450 2A4	0,0344538	0,0397934
458	SPTB1_MOUSE	Sptb	Spectrin beta chain, erythrocytic	0,395018	0,398886
459	NDKA_MOUSE	Nme1	Nucleoside diphosphate kinase A	0,413094	-0,341826
460	GSTM2_MOUSE	Gstm2	Glutathione S-transferase Mu 2	0,391772	-0,611282
461	H12_MOUSE	Hist1h1c	Histone H1.2	0,228449	0,178421
462	CAH3_MOUSE	Ca3	Carbonic anhydrase 3	1,52666	0,855269
463	LEG1_MOUSE	Lgals1	Galectin-1	1,28523	-0,700853

464	MET_MOUSE	Met	Hepatocyte growth factor receptor	1,02599	-0,304887
465	LDHB_MOUSE	Ldhb	L-lactate dehydrogenase B chain	0,796636	-0,989117
466	SRP14_MOUSE	Srp14	Signal recognition particle 14 kDa protein	1,68508	0,32029
467	CN37_MOUSE	Cnp	2',3'-cyclic-nucleotide 3'-phosphodiesterase	0,314089	0,411969
468	PH4H_MOUSE	Pah	Phenylalanine-4-hydroxylase	0,0822138	-0,0764763
469	MUTA_MOUSE	Mmut	Methylmalonyl-CoA mutase, mitochondrial	2,0952	-0,497775
470	AMPE_MOUSE	Enpep	Glutamyl aminopeptidase	0,753343	-0,495048
471	ASSY_MOUSE	Ass1	Argininosuccinate synthase	0,762687	-0,801434
472	SPTN1_MOUSE	Sptan1	Spectrin alpha chain, non-erythrocytic 1	0,414277	0,244096
473	PPGB_MOUSE	Ctsa	Lysosomal protective protein	0,48871	0,35078
474	G3P_MOUSE	Gapdh	Glyceraldehyde-3-phosphate dehydrogenase	0,106863	0,199353
475	LAMP2_MOUSE	Lamp2	Lysosome-associated membrane glycoprotein 2	0,951957	-0,59334
476	ENOA_MOUSE	Eno1	Alpha-enolase	1,71215	1,12418
477	PTBP1_MOUSE	Ptbp1	Polypyrimidine tract-binding protein 1	0,299631	0,245593
478	AP2A1_MOUSE	Ap2a1	AP-2 complex subunit alpha-1	0,597408	0,454369
479	AP2A2_MOUSE	Ap2a2	AP-2 complex subunit alpha-2	2,38656	0,455393
480	SBP1_MOUSE	Selenbp1	Methanethiol oxidase	0,789007	-0,521093
481	COX7C_MOUSE	Cox7c	Cytochrome c oxidase subunit 7C, mitochondrial	1,3135	-0,693682
482	HXK1_MOUSE	Hk1	Hexokinase-1	0,22724	0,261738
483	UDB17_MOUSE	Ugt2b17	UDP-glucuronosyltransferase 2B17	0,644155	0,391264
484	PPIA_MOUSE	Ppia	Peptidyl-prolyl cis-trans isomerase A	0,638641	0,144875
485	TPIS_MOUSE	Tpi1	Triosephosphate isomerase	0,686805	-0,134504
486	HS71A_MOUSE;HS7	Hspa1a;Hspa1b	Heat shock 70 kDa protein 1B	2,54955	0,586753
487	PCNA_MOUSE	Pcna	Proliferating cell nuclear antigen	0,740786	0,447347
488	CATD_MOUSE	Ctsd	Cathepsin D	1,25336	0,493399
489	BASI_MOUSE	Bsg	Basigin	0,307283	0,23465
490	KS6A3_MOUSE	Rps6ka3	Ribosomal protein S6 kinase alpha-3	0,338644	0,111446
491	COF1_MOUSE	Cfl1	Cofilin-1	1,30018	0,550194
492	K1C19_MOUSE	Krt19	Keratin, type I cytoskeletal 19	0,342502	-0,231685
493	FAS_MOUSE	Fasn	Fatty acid synthase	0,00111809	0,00227318
494	GSTP1_MOUSE	Gstp1	Glutathione S-transferase P 1	0,659962	1,05631
495	THRB_MOUSE	F2	Prothrombin	0,71439	0,229208
496	RL13A_MOUSE	Rpl13a	60S ribosomal protein L13a	0,204342	0,109268
497	SERPH_MOUSE	Serpinh1	Serpin H1	0,294222	-0,252639
498	COX5B_MOUSE	Cox5b	Cytochrome c oxidase subunit 5B, mitochondrial	0,612838	-0,216396
499	GSTM3_MOUSE	Gstm3	Glutathione S-transferase Mu 3	0,349192	-0,483163
500	COX41_MOUSE	Cox4i1	Cytochrome c oxidase subunit 4 isoform 1, mitochondrial	1,66774	1,16518
501	LSP1_MOUSE	Lsp1	Lymphocyte-specific protein 1	0,856715	0,400934
502	BIP_MOUSE	Hspa5	Endoplasmic reticulum chaperone BiP	0,658072	0,283416
503	HEXB_MOUSE	Hexb	Beta-hexosaminidase subunit beta	0,230887	0,256398
504	TYB4_MOUSE	Tmsb4x	Thymosin beta-4	0,450846	-0,734558
505	PRDX3_MOUSE	Prdx3	Thioredoxin-dependent peroxide reductase, mitochondrial	0,0619792	-0,0431614
506	VIME_MOUSE	Vim	Vimentin	0,0571225	0,0741913
507	CP2A5_MOUSE	Cyp2a5	Cytochrome P450 2A5	0,958019	-0,977493
508	PLMN_MOUSE	Plg	Plasminogen	0,995804	0,250274
509	TPM3_MOUSE	Tpm3	Tropomyosin alpha-3 chain	1,23519	0,490975
510	UBL4A_MOUSE	Ubl4a	Ubiquitin-like protein 4A	0,818863	0,288529
511	MYO5B_MOUSE	Myo5b	Unconventional myosin-Vb	0,586512	-0,451186
512	GNA11_MOUSE	Gna11	Guanine nucleotide-binding protein subunit alpha-11	0,480032	-0,372295
513	GNAQ_MOUSE	Gnaq	Guanine nucleotide-binding protein G(q) subunit alpha	0,162967	0,166125
514	ALD1_MOUSE	Akr1b7	Aldo-keto reductase family 1 member B7	1,33888	-0,805934
515	MDR3_MOUSE	Abcb4	Phosphatidylcholine translocator ABCB4	0,541101	0,199294

516	ENOB_MOUSE	Eno3	Beta-enolase	0,23344	-0,590524
517	ANGI_MOUSE	Ang	Angiogenin	0,173994	0,301687
518	VTDB_MOUSE	Gc	Vitamin D-binding protein	0,983941	0,587824
519	LMNB2_MOUSE	Lmnb2	Lamin-B2	0,107962	-0,0796669
520	TGM2_MOUSE	Tgm2	Protein-glutamine gamma-glutamyltransferase 2	0,469978	0,295403
521	HEMH_MOUSE	Fech	Ferrochelatase, mitochondrial	0,935131	0,498508
522	MYD88_MOUSE	Myd88	Myeloid differentiation primary response protein MyD88	0,724168	0,698199
523	PGH1_MOUSE	Ptgs1	Prostaglandin G/H synthase 1	0,326805	0,153155
524	A1AT2_MOUSE	Serpina1b	Alpha-1-antitrypsin 1-2	0,57066	-0,601443
525	AP1G1_MOUSE	Ap1g1	AP-1 complex subunit gamma-1	0,426324	-0,432601
526	HEM3_MOUSE	Hmbs	Porphobilinogen deaminase	0,722536	0,553481
527	EIF3A_MOUSE	Eif3a	Eukaryotic translation initiation factor 3 subunit A	1,88251	0,294622
528	CBX3_MOUSE	Cbx3	Chromobox protein homolog 3	0,371172	0,121444
529	MOV10_MOUSE	Mov10	Putative helicase MOV-10	0,0236367	0,021796
530	XRCC6_MOUSE	Xrcc6	X-ray repair cross-complementing protein 6	0,0448325	-0,100252
531	PNPH_MOUSE	Pnp	Purine nucleoside phosphorylase	1,04722	-0,74254
532	PIMT_MOUSE	Pcmt1	Protein-L-isoaspartate(D-aspartate) O-methyltransferase	0,606323	0,337132
533	CAH5A_MOUSE	Ca5a	Carbonic anhydrase 5A, mitochondrial	0,145159	-0,0947678
534	FCL_MOUSE	Tsta3	GDP-L-fucose synthase	0,899905	0,626199
535	BGAL_MOUSE	Glb1	Beta-galactosidase	0,972293	-0,391734
536	TCEA3_MOUSE	Tcea3	Transcription elongation factor A protein 3	0,708712	0,70404
537	TISB_MOUSE	Zfp36l1	mRNA decay activator protein ZFP36L1	0,919025	-0,902974
538	EST1C_MOUSE	Ces1c	Carboxylesterase 1C	0,335395	-0,276857
539	CATA_MOUSE	Cat	Catalase	1,96554	0,184806
540	PPIB_MOUSE	Ppib	Peptidyl-prolyl cis-trans isomerase B	1,43089	0,368091
541	CAPG_MOUSE	Capg	Macrophage-capping protein	0,0343255	-0,0637749
542	CP2DA_MOUSE	Cyp2d10	Cytochrome P450 2D10	0,189718	-0,127562
543	GSTA4_MOUSE	Gsta4	Glutathione S-transferase A4	1,22292	-0,525446
544	LKHA4_MOUSE	Lta4h	Leukotriene A-4 hydrolase	0,148381	0,059581
545	IMDH2_MOUSE	Impdh2	Inosine-5'-monophosphate dehydrogenase 2	0,72214	0,24768
546	AL1A1_MOUSE	Aldh1a1	Retinal dehydrogenase 1	1,24652	1,00063
547	ASGR2_MOUSE	Asgr2	Asialoglycoprotein receptor 2	0,506536	-1,13302
548	CD11B_MOUSE	Cdk11b	Cyclin-dependent kinase 11B	0,19692	-0,10849
549	3BHS1_MOUSE	Hsd3b1	3 beta-hydroxysteroid dehydrogenase/Delta 5-->4-isomerase type 1	0,986788	-1,20163
550	MCM3_MOUSE	Mcm3	DNA replication licensing factor MCM3	0,569278	0,517851
551	CCND1_MOUSE	Ccnd1	G1/S-specific cyclin-D1	2,46133	0,555096
552	RS2_MOUSE	Rps2	40S ribosomal protein S2	0,917719	0,441904
553	URIC_MOUSE	Uox	Uricase	0,575304	-0,76055
554	NFKB1_MOUSE	Nfkb1	Nuclear factor NF-kappa-B p105 subunit	0,00262303	0,00119286
555	LYN_MOUSE	Lyn	Tyrosine-protein kinase Lyn	0,312073	0,285716
556	UBF1_MOUSE	Ubtf	Nucleolar transcription factor 1	0,74428	0,213834
557	TLN1_MOUSE	Tln1	Talin-1	1,04338	0,377521
558	EZRI_MOUSE	Ezr	Ezrin	0,405091	0,185784
559	MOES_MOUSE	Msn	Moesin	0,794247	0,499309
560	RADI_MOUSE	Rdx	Radixin	0,180965	-0,145327
561	3BHS3_MOUSE	Hsd3b3	3 beta-hydroxysteroid dehydrogenase/Delta 5-->4-isomerase type 3	0,253847	-0,143249
562	CTNA1_MOUSE	Ctnna1	Catenin alpha-1	0,0835405	0,0522667
563	KLKB1_MOUSE	Klkb1	Plasma kallikrein	1,07587	0,771715
564	PTMA_MOUSE	Ptma	Prothymosin alpha	0,142798	0,131416
565	U2AF2_MOUSE	U2af2	Splicing factor U2AF 65 kDa subunit	0,680817	-0,362292
566	DHE3_MOUSE	Glud1	Glutamate dehydrogenase 1, mitochondrial	0,620755	0,15096
567	P85A_MOUSE	Pik3r1	Phosphatidylinositol 3-kinase regulatory subunit alpha	0,267744	-0,469772

568	PSMD7_MOUSE	Psmd7	26S proteasome non-ATPase regulatory subunit 7	0,787251	0,190926
569	SYSC_MOUSE	Sars	Serine--tRNA ligase, cytoplasmic	2,91396	0,418105
570	S10A8_MOUSE	S100a8	Protein S100-A8	1,50137	1,68392
571	MA2A1_MOUSE	Man2a1	Alpha-mannosidase 2	0,745803	0,275576
572	RSMB_MOUSE;RSM	Snrpb;Snrpn	Small nuclear ribonucleoprotein-associated protein B	0,500252	0,177144
573	MAP4_MOUSE	Map4	Microtubule-associated protein 4	0,466337	0,243614
574	GNA13_MOUSE	Gna13	Guanine nucleotide-binding protein subunit alpha-13	0,826205	0,251391
575	PLAP_MOUSE	Plaa	Phospholipase A-2-activating protein	0,394638	0,158889
576	XRCC5_MOUSE	Xrcc5	X-ray repair cross-complementing protein 5	0,569314	-0,360449
577	RL3_MOUSE	Rpl3	60S ribosomal protein L3	0,407279	0,370742
578	H2AX_MOUSE	H2afx	Histone H2AX	1,33846	0,322171
579	PDIA3_MOUSE	Pdia3	Protein disulfide-isomerase A3	1,76303	0,895212
580	CP17A_MOUSE	Cyp17a1	Steroid 17-alpha-hydroxylase/17,20 lyase	0,583135	-0,500576
581	MGAT1_MOUSE	Mgat1	Alpha-1,3-mannosyl-glycoprotein 2-beta-N-glucosaminyltransferase	0,431583	0,240067
582	CEBPB_MOUSE	Cebpb	CCAAT/enhancer-binding protein beta	0,866563	-0,621975
583	PSB8_MOUSE	Psmb8	Proteasome subunit beta type-8	0,243876	-0,29677
584	CXB1_MOUSE	Gjb1	Gap junction beta-1 protein	1,78216	-0,700304
585	ACOC_MOUSE	Aco1	Cytoplasmic aconitate hydratase	0,395704	0,28431
586	APEX1_MOUSE	Apex1	DNA-(apurinic or apyrimidinic site) lyase	0,0683283	0,062524
587	ADHX_MOUSE	Adh5	Alcohol dehydrogenase class-3	0,137818	0,0625732
588	PURA1_MOUSE	Adssl1	Adenylosuccinate synthetase isozyme 1	0,0133174	-0,0132568
589	CAH8_MOUSE	Ca8	Carbonic anhydrase-related protein	0,240437	0,15464
590	PGS1_MOUSE	Bgn	Biglycan	0,0221247	0,0167316
591	PGS2_MOUSE	Dcn	Decorin	0,290195	-0,437787
592	NP1L1_MOUSE	Nap1l1	Nucleosome assembly protein 1-like 1	0,873198	0,259452
593	ATX10_MOUSE	Atxn10	Ataxin-10	1,00715	-0,414239
594	CELF1_MOUSE	Celf1	CUGBP Elav-like family member 1	0,237784	0,128151
595	NCKP1_MOUSE	Nckap1	Nck-associated protein 1	0,881393	-0,184591
596	SEPT4_MOUSE	Septin4	Septin-4	0,0104801	0,008741
597	MUG1_MOUSE	Mug1	Murinoglobulin-1	0,308597	0,308126
598	MUG2_MOUSE	Mug2	Murinoglobulin-2	2,84662	0,822369
599	RXRA_MOUSE	Rxra	Retinoic acid receptor RXR-alpha	1,69935	0,209333
600	GRN_MOUSE	Grn	Progranulin	0,154036	0,146847
601	DPP4_MOUSE	Dpp4	Dipeptidyl peptidase 4	0,123596	-0,124923
602	KPCD_MOUSE	Prkcd	Protein kinase C delta type	0,943302	0,637051
603	PABP1_MOUSE	Pabpc1	Polyadenylate-binding protein 1	0,471943	0,235259
604	PTN6_MOUSE	Ptpn6	Tyrosine-protein phosphatase non-receptor type 6	0,0464582	0,0351742
605	FRIL1_MOUSE	Ftl1	Ferritin light chain 1	0,479175	1,10465
606	VCAM1_MOUSE	Vcam1	Vascular cell adhesion protein 1	0,200577	-0,162471
607	NEDD8_MOUSE	Nedd8	NEDD8	0,218763	-0,0911751
608	FETUA_MOUSE	Ahsg	Alpha-2-HS-glycoprotein	0,663992	-0,473175
609	OAT_MOUSE	Oat	Ornithine aminotransferase, mitochondrial	0,496433	-0,542378
610	VTNC_MOUSE	Vtn	Vitronectin	1,61815	0,745412
611	GSTA3_MOUSE	Gsta3	Glutathione S-transferase A3	0,552992	-0,245564
612	PPIC_MOUSE	Ppic	Peptidyl-prolyl cis-trans isomerase C	0,437179	-0,502412
613	FKBP4_MOUSE	Fkbp4	Peptidyl-prolyl cis-trans isomerase FKBP4	0,962881	0,440101
614	HMGB2_MOUSE	Hmgb2	High mobility group protein B2	0,729382	-1,05892
615	CTND1_MOUSE	Ctnnd1	Catenin delta-1	0,803846	0,225111
616	DESM_MOUSE	Des	Desmin	0,200516	0,293048
617	AIMP1_MOUSE	Aimp1	Aminoacyl tRNA synthase complex-interacting protein 1	0,987327	0,499415
618	S10A9_MOUSE	S100a9	Protein S100-A9	2,09407	1,39158
619	ACBP_MOUSE	Dbi	Acyl-CoA-binding protein	0,40411	-0,219909

620	MP2K1_MOUSE	Map2k1	Dual specificity mitogen-activated protein kinase kinase 1	0,134567	0,110665
621	NLTP_MOUSE	Scp2	Non-specific lipid-transfer protein	2,22244	1,40545
622	LA_MOUSE	Ssb	Lupus La protein homolog	0,384811	0,194939
623	DRG1_MOUSE	Drg1	Developmentally-regulated GTP-binding protein 1	0,1161	0,0555004
624	ANT3_MOUSE	Serpinc1	Antithrombin-III	0,191382	0,148637
625	NECT2_MOUSE	Nectin2	Nectin-2	0,600924	0,300293
626	RASK_MOUSE	Kras	GTPase KRas	0,573661	0,25433
627	SYWC_MOUSE	Wars	Tryptophan--tRNA ligase, cytoplasmic	1,1661	0,199219
628	CP2F2_MOUSE	Cyp2f2	Cytochrome P450 2F2	0,172341	-0,168359
629	PROC_MOUSE	Proc	Vitamin K-dependent protein C	1,26212	0,584216
630	APOC3_MOUSE	Apoc3	Apolipoprotein C-III	0,151848	0,203619
631	RANG_MOUSE	Ranbp1	Ran-specific GTPase-activating protein	0,996131	-0,439364
632	FAK1_MOUSE	Ptk2	Focal adhesion kinase 1	0,0627694	0,0586716
633	HYES_MOUSE	Ephx2	Bifunctional epoxide hydrolase 2	3,77204	0,695476
634	ASGR1_MOUSE	Asgr1	Asialoglycoprotein receptor 1	0,551325	-0,242605
635	APOC1_MOUSE	Apoc1	Apolipoprotein C-I	0,628475	0,270815
636	UBP4_MOUSE	Usp4	Ubiquitin carboxyl-terminal hydrolase 4	0,091717	-0,0691235
637	PTN11_MOUSE	Ptpn11	Tyrosine-protein phosphatase non-receptor type 11	0,845542	0,358399
638	RAB5C_MOUSE	Rab5c	Ras-related protein Rab-5C	0,463857	-0,256536
639	RAB6A_MOUSE	Rab6a	Ras-related protein Rab-6A	0,151656	0,140311
640	RAB21_MOUSE	Rab21	Ras-related protein Rab-21	1,02934	0,681445
641	RAB18_MOUSE	Rab18	Ras-related protein Rab-18	2,27503	0,515598
642	TSP1_MOUSE	Thbs1	Thrombospondin-1	1,51064	0,632071
643	ODPA_MOUSE	Pdha1	Pyruvate dehydrogenase E1	0,185445	-0,0726902
644	HUTH_MOUSE	Hal	Histidine ammonia-lyase	0,638792	-0,332693
645	FAAA_MOUSE	Fah	Fumarylacetoacetase	0,551651	-0,977559
646	FBRL_MOUSE	Fbl	rRNA 2'-O-methyltransferase fibrillarin	0,193326	-0,0726055
647	CALX_MOUSE	Canx	Calnexin	0,247888	-0,0728432
648	IRS1_MOUSE	Irs1	Insulin receptor substrate 1	0,300196	0,220712
649	AP1M1_MOUSE	Ap1m1	AP-1 complex subunit mu-1	0,339811	0,282882
650	PRDX1_MOUSE	Prdx1	Peroxiredoxin-1	0,583032	-0,291983
651	PTN1_MOUSE	Ptpn1	Tyrosine-protein phosphatase non-receptor type 1	0,407007	0,22588
652	PTPRK_MOUSE	Ptprk	Receptor-type tyrosine-protein phosphatase kappa	1,02489	0,89446
653	PTN12_MOUSE	Ptpn12	Tyrosine-protein phosphatase non-receptor type 12	0,854802	-0,512933
654	FMR1_MOUSE	Fmr1	Synaptic functional regulator FMR1	0,474507	0,317738
655	LDLR_MOUSE	Ldlr	Low-density lipoprotein receptor	0,758623	0,474814
656	RL12_MOUSE	Rpl12	60S ribosomal protein L12	0,843321	0,745198
657	RL18_MOUSE	Rpl18	60S ribosomal protein L18	0,4165	0,363221
658	TAP2_MOUSE	Tap2	Antigen peptide transporter 2	0,196228	0,114317
659	HEM6_MOUSE	Cpox	Oxygen-dependent coproporphyrinogen-III oxidase	0,434151	0,233881
660	PPM1B_MOUSE	Ppm1b	Protein phosphatase 1B	0,119564	0,136725
661	NCPR_MOUSE	Por	NADPH--cytochrome P450 reductase	0,0849437	-0,0480217
662	TAGL_MOUSE	Tagln	Transgelin	0,718022	-0,957339
663	HMGCL_MOUSE	Hmgcl	Hydroxymethylglutaryl-CoA lyase, mitochondrial	0,00838672	-0,00393715
664	GRP75_MOUSE	Hspa9	Stress-70 protein, mitochondrial	0,917488	0,599097
665	MBL1_MOUSE	Mbl1	Mannose-binding protein A	2,36235	0,895544
666	DYN2_MOUSE	Dnm2	Dynamin-2	0,943283	0,335656
667	COIA1_MOUSE	Col18a1	Collagen alpha-1(XVIII) chain	0,585297	0,340623
668	ZO1_MOUSE	Tjp1	Tight junction protein ZO-1	0,34373	-0,142363
669	FEN1_MOUSE	Fen1	Flap endonuclease 1	0,886657	0,47333
670	CAP1_MOUSE	Cap1	Adenylyl cyclase-associated protein 1	0,451152	0,313747
671	TKT_MOUSE	Tkt	Transketolase	0,764726	0,513929

672	VP26A_MOUSE	Vps26a	Vacuolar protein sorting-associated protein 26A	0,575798	-0,337981
673	VHL_MOUSE	Vhl	von Hippel-Lindau disease tumor suppressor	0,914902	0,589205
674	TFAM_MOUSE	Tfam	Transcription factor A, mitochondrial	0,86551	0,182943
675	INMT_MOUSE	Inmt	Indolethylamine N-methyltransferase	0,609034	-0,33261
676	RL28_MOUSE	Rpl28	60S ribosomal protein L28	0,100932	-0,0496159
677	ACSL1_MOUSE	Acsl1	Long-chain-fatty-acid--CoA ligase 1	0,0961624	-0,0735252
678	ABCA1_MOUSE	Abca1	Phospholipid-transporting ATPase ABCA1	0,676734	0,366715
679	CSK_MOUSE	Csk	Tyrosine-protein kinase CSK	0,0595363	-0,0215797
680	MBL2_MOUSE	Mbl2	Mannose-binding protein C	1,90785	0,607495
681	ECI1_MOUSE	Eci1	Enoyl-CoA delta isomerase 1, mitochondrial	0,281724	-0,147007
682	FOXK1_MOUSE	Foxk1	Forkhead box protein K1	0,0117323	-0,00999527
683	SEPT2_MOUSE	Septin2	Septin-2	0,517407	0,202602
684	STAT1_MOUSE	Stat1	Signal transducer and activator of transcription 1	0,924074	-0,406846
685	STAT3_MOUSE	Stat3	Signal transducer and activator of transcription 3	0,535821	0,172846
686	STA5A_MOUSE	Stat5a	Signal transducer and activator of transcription 5A	0,103921	-0,116742
687	STA5B_MOUSE	Stat5b	Signal transducer and activator of transcription 5B	0,120184	-0,101437
688	PK3CA_MOUSE	Pik3ca	Phosphatidylinositol 4,5-bisphosphate 3-kinase	0,0726465	-0,0383381
689	EPS15_MOUSE	Eps15	Epidermal growth factor receptor substrate 15	0,725628	0,1697
690	PURA_MOUSE	Pura	Transcriptional activator protein Pur-alpha	0,898208	0,336817
691	LIFR_MOUSE	Lifr	Leukemia inhibitory factor receptor	0,00929241	0,0114697
692	TCPQ_MOUSE	Cct8	T-complex protein 1 subunit theta	1,54573	0,543346
693	TETN_MOUSE	Clec3b	Tetranectin	0,273446	0,102824
694	MSH2_MOUSE	Msh2	DNA mismatch repair protein Msh2	0,170966	-0,179272
695	H14_MOUSE	Hist1h1e	Histone H1.4	0,514125	1,42293
696	H11_MOUSE	Hist1h1a	Histone H1.1	0,111242	-0,0980419
697	H15_MOUSE	Hist1h1b	Histone H1.5	0,177035	-0,0792328
698	ITAV_MOUSE	Itgav	Integrin alpha-V	0,952488	0,390231
699	PLIN2_MOUSE	Plin2	Perilipin-2	1,28847	1,69409
700	ALDR_MOUSE	Akr1b1	Aldo-keto reductase family 1 member B1	0,254012	-0,0856003
701	ALD2_MOUSE	Akr1b8	Aldose reductase-related protein 2	0,021318	0,0153309
702	CBP_MOUSE	Crebbp	CREB-binding protein	0,0364879	0,0814487
703	COF2_MOUSE	Cfl2	Cofilin-2	0,693706	0,404613
704	MA1A1_MOUSE	Man1a1	Mannosyl-oligosaccharide 1,2-alpha-mannosidase IA	0,113403	0,102908
705	FKBP2_MOUSE	Fkbp2	Peptidyl-prolyl cis-trans isomerase FKBP2	0,459708	0,402418
706	ACADM_MOUSE	Acadm	Medium-chain specific acyl-CoA dehydrogenase, mitochondrial	0,391706	-0,235801
707	RAGP1_MOUSE	Rangap1	Ran GTPase-activating protein 1	0,297441	-0,11931
708	SIPA1_MOUSE	Sipa1	Signal-induced proliferation-associated protein 1	0,0224482	-0,0410172
709	GPX3_MOUSE	Gpx3	Glutathione peroxidase 3	0,620644	-0,298275
710	CDN1B_MOUSE	Cdkn1b	Cyclin-dependent kinase inhibitor 1B	1,26253	0,266585
711	NSF_MOUSE	Nsf	Vesicle-fusing ATPase	0,881093	0,260408
712	VPS4B_MOUSE	Vps4b	Vacuolar protein sorting-associated protein 4B	0,412226	0,363902
713	PRS7_MOUSE	Psmc2	26S proteasome regulatory subunit 7	0,810876	0,164662
714	RB11B_MOUSE	Rab11b	Ras-related protein Rab-11B	0,370838	-0,155098
715	ADX_MOUSE	Fdx1	Adrenodoxin, mitochondrial	0,342207	0,297111
716	PURA2_MOUSE	Adss	Adenylosuccinate synthetase isozyme 2	1,04341	0,516118
717	PDCD2_MOUSE	Pdcd2	Programmed cell death protein 2	1,12352	0,592668
718	MYO1B_MOUSE	Myo1b	Unconventional myosin-Ib	0,193052	0,0385262
719	BRCC3_MOUSE	Brcc3	Lys-63-specific deubiquitinase BRCC36	0,719706	-0,946062
720	NEDD4_MOUSE	Nedd4	E3 ubiquitin-protein ligase NEDD4	0,749011	0,208867
721	YAP1_MOUSE	Yap1	Transcriptional coactivator YAP1	1,3011	0,445317
722	STT3A_MOUSE	Stt3a	Dolichyl-diphosphooligosaccharide--protein glycosyltransferase	0,248734	-0,252344
723	QOR_MOUSE	Cryz	Quinone oxidoreductase	0,178721	-0,0927654

724	ALDH2_MOUSE	Aldh2	Aldehyde dehydrogenase, mitochondrial	0,171202	0,146772
725	AL3A2_MOUSE	Aldh3a2	Aldehyde dehydrogenase family 3 member A2	0,29979	0,149913
726	CAZA1_MOUSE	Capza1	F-actin-capping protein subunit alpha-1	0,568578	0,320848
727	CAZA2_MOUSE	Capza2	F-actin-capping protein subunit alpha-2	0,350047	-0,907615
728	CAPZB_MOUSE	Capzb	F-actin-capping protein subunit beta	0,0752077	-0,0131855
729	SRPRB_MOUSE	Srprb	Signal recognition particle receptor subunit beta	0,869702	0,24093
730	GSHR_MOUSE	Gsr	Glutathione reductase, mitochondrial	1,54469	0,60327
731	MTX1_MOUSE	Mtx1	Metaxin-1	0,31062	-0,26578
732	MP2K4_MOUSE	Map2k4	Dual specificity mitogen-activated protein kinase kinase 4	0,988317	0,447959
733	MK14_MOUSE	Mapk14	Mitogen-activated protein kinase 14	0,747592	-1,02294
734	GFPT1_MOUSE	Gfpt1	Glutamine--fructose-6-phosphate aminotransferase [isomerizing] 1	0,382066	0,191678
735	PFKAM_MOUSE	Pfkm	ATP-dependent 6-phosphofructokinase, muscle type	0,132372	0,181871
736	IBP1_MOUSE	Igfbp1	Insulin-like growth factor-binding protein 1	0,130068	-0,237882
737	IBP3_MOUSE	Igfbp3	Insulin-like growth factor-binding protein 3	1,08746	1,41666
738	IBP4_MOUSE	Igfbp4	Insulin-like growth factor-binding protein 4	1,19632	0,835209
739	RL6_MOUSE	Rpl6	60S ribosomal protein L6	0,698418	0,336848
740	RL29_MOUSE	Rpl29	60S ribosomal protein L29	0,0294022	-0,0143879
741	CACP_MOUSE	Crat	Carnitine O-acetyltransferase	0,428568	0,313346
742	CRKL_MOUSE	Crkl	Crk-like protein	0,630837	0,309932
743	RL5_MOUSE	Rpl5	60S ribosomal protein L5	0,147226	0,0860973
744	RL13_MOUSE	Rpl13	60S ribosomal protein L13	0,0544254	-0,0250561
745	RL36_MOUSE	Rpl36	60S ribosomal protein L36	0,0099274	0,00434875
746	EIF1_MOUSE	Eif1	Eukaryotic translation initiation factor 1	0,663284	-0,338169
747	KSYK_MOUSE	Syk	Tyrosine-protein kinase SYK	1,06352	1,02045
748	ANXA5_MOUSE	Anxa5	Annexin A5	0,433626	-0,30325
749	41_MOUSE	Epb41	Protein 4.1	0,21625	0,152479
750	TBCA_MOUSE	Tbca	Tubulin-specific chaperone A	0,867635	0,461982
751	LMNA_MOUSE	Lmna	Prelamin-A/C	0,200313	0,123799
752	HS74L_MOUSE	Hspa4l	Heat shock 70 kDa protein 4L	1,44978	0,629794
753	CBR1_MOUSE	Cbr1	Carbonyl reductase [NADPH] 1	0,884082	0,591402
754	GSTM5_MOUSE	Gstm5	Glutathione S-transferase Mu 5	0,338253	-0,243084
755	T23O_MOUSE	Tdo2	Tryptophan 2,3-dioxygenase	2,36877	-1,51069
756	ADT1_MOUSE	Slc25a4	ADP/ATP translocase 1	1,22179	-1,09897
757	MAPK2_MOUSE	Mapkapk2	MAP kinase-activated protein kinase 2	2,02569	0,747411
758	HEP2_MOUSE	Serpind1	Heparin cofactor 2	0,00949619	0,0121799
759	EPB42_MOUSE	Epb42	Erythrocyte membrane protein band 4.2	1,83979	1,00016
760	PERE_MOUSE	Epx	Eosinophil peroxidase	1,98472	1,54596
761	ROA1_MOUSE	Hnrnpa1	Heterogeneous nuclear ribonucleoprotein A1	0,658106	0,401308
762	HPPD_MOUSE	Hpd	4-hydroxyphenylpyruvate dioxygenase	2,09036	-1,08078
763	INPP_MOUSE	Inpp1	Inositol polyphosphate 1-phosphatase	0,836191	0,949996
764	PPM1A_MOUSE	Ppm1a	Protein phosphatase 1A	0,545054	0,496687
765	PCY1A_MOUSE	Pcyt1a	Choline-phosphate cytidylyltransferase A	0,247588	0,300076
766	CDK5_MOUSE	Cdk5	Cyclin-dependent-like kinase 5	0,0539529	-0,0466946
767	HNF4A_MOUSE	Hnf4a	Hepatocyte nuclear factor 4-alpha	0,0825733	-0,0399578
768	HCLS1_MOUSE	Hcls1	Hematopoietic lineage cell-specific protein	1,47342	0,556715
769	MCM4_MOUSE	Mcm4	DNA replication licensing factor MCM4	0,992409	0,504875
770	MCM5_MOUSE	Mcm5	DNA replication licensing factor MCM5	0,568314	0,506454
771	PSA2_MOUSE	Psma2	Proteasome subunit alpha type-2	0,857223	0,36588
772	CATH_MOUSE	Ctsh	Pro-cathepsin H	0,731712	-0,624318
773	SRP09_MOUSE	Srp9	Signal recognition particle 9 kDa protein	1,43728	0,460791
774	ODBA_MOUSE	Bckdha	2-oxoisovalerate dehydrogenase subunit alpha, mitochondrial	0,278565	0,149389
775	DHB8_MOUSE	Hsd17b8	Estradiol 17-beta-dehydrogenase 8	0,00569887	-0,00644417

776	DHI1_MOUSE	Hsd11b1	Corticosteroid 11-beta-dehydrogenase isozyme 1	0,177685	0,091927
777	ST2A2_MOUSE	Sult2a2	Bile salt sulfotransferase 2	1,58923	-2,00991
778	SAHH_MOUSE	Ahcy	Adenosylhomocysteinase	1,20913	1,03002
779	FMO1_MOUSE	Fmo1	Dimethylaniline monooxygenase [N-oxide-forming] 1	0,920318	0,609616
780	ARY2_MOUSE	Nat2	Arylamine N-acetyltransferase 2	0,93655	0,548981
781	GDIA_MOUSE	Gdi1	Rab GDP dissociation inhibitor alpha	0,0830815	-0,0658836
782	STS_MOUSE	Sts	Steryl-sulfatase	0,84191	0,462734
783	ARSB_MOUSE	Arsb	Arylsulfatase B	0,362149	-0,348869
784	GLYC_MOUSE	Shmt1	Serine hydroxymethyltransferase, cytosolic	0,294306	0,116143
785	CSRP3_MOUSE	Csrp3	Cysteine and glycine-rich protein 3	0,493954	0,382003
786	VATA_MOUSE	Atp6v1a	V-type proton ATPase catalytic subunit A	0,492364	0,406726
787	VATE1_MOUSE	Atp6v1e1	V-type proton ATPase subunit E 1	0,7009	0,256028
788	S10AB_MOUSE	S100a11	Protein S100-A11	0,339302	-0,90506
789	ACADV_MOUSE	Acadvl	Very long-chain specific acyl-CoA dehydrogenase, mitochondrial	1,85231	0,497198
790	PA2G4_MOUSE	Pa2g4	Proliferation-associated protein 2G4	0,698933	0,258555
791	FMOD_MOUSE	Fmod	Fibromodulin	0,256852	0,71948
792	ICAL_MOUSE	Cast	Calpastatin	0,111665	0,125669
793	RAB7A_MOUSE	Rab7a	Ras-related protein Rab-7a	0,469618	-0,206083
794	ACADL_MOUSE	Acadl	Long-chain specific acyl-CoA dehydrogenase, mitochondrial	0,922397	0,79673
795	PPOX_MOUSE	Ppox	Protoporphyrinogen oxidase	0,670762	0,46838
796	RL9_MOUSE	Rpl9	60S ribosomal protein L9	1,07614	0,595771
797	PLCB3_MOUSE	Plcb3	1-phosphatidylinositol 4,5-bisphosphate phosphodiesterase beta-3	0,517728	0,249361
798	GPC4_MOUSE	Gpc4	Glypican-4	1,18057	0,732
799	DHB2_MOUSE	Hsd17b2	Estradiol 17-beta-dehydrogenase 2	0,0722261	0,0877129
800	DHB4_MOUSE	Hsd17b4	Peroxisomal multifunctional enzyme type 2	1,93409	0,84901
801	GSHB_MOUSE	Gss	Glutathione synthetase	1,12667	0,828722
802	HDGF_MOUSE	Hdgf	Hepatoma-derived growth factor	0,283146	0,124926
803	VA0D1_MOUSE	Atp6v0d1	V-type proton ATPase subunit d 1	0,107272	-0,140431
804	ADT2_MOUSE	Slc25a5	ADP/ATP translocase 2	0,278786	-0,253061
805	LUM_MOUSE	Lum	Lumican	0,0182047	-0,0254864
806	THTR_MOUSE	Tst	Thiosulfate sulfurtransferase	0,410352	0,179684
807	IMA1_MOUSE	Kpna2	Importin subunit alpha-1	0,339881	0,32834
808	JAK1_MOUSE	Jak1	Tyrosine-protein kinase JAK1	0,0508851	0,0140144
809	PON1_MOUSE	Pon1	Serum paraoxonase/arylesterase 1	0,209069	-0,216276
810	DPOD1_MOUSE	Pold1	DNA polymerase delta catalytic subunit	0,665337	0,396667
811	RPAC1_MOUSE	Polr1c	DNA-directed RNA polymerases I and III subunit RPAC1	1,06583	0,35201
812	UBP10_MOUSE	Usp10	Ubiquitin carboxyl-terminal hydrolase 10	0,236328	-0,115229
813	KPYM_MOUSE	Pkm	Pyruvate kinase PKM	0,28205	0,185635
814	NDUS6_MOUSE	Ndufs6	NADH dehydrogenase [ubiquinone] iron-sulfur protein 6, mitochondrial	0,0218266	-0,012532
815	UCK1_MOUSE	Uck1	Uridine-cytidine kinase 1	3,19081	0,532621
816	STAT6_MOUSE	Stat6	Signal transducer and transcription activator 6	0,0718715	-0,0715439
817	RIDA_MOUSE	Rida	2-iminobutanoate/2-iminopropanoate deaminase	0,468979	0,222734
818	HXK4_MOUSE	Gck	Hexokinase-4	0,321385	-0,296624
819	CPT2_MOUSE	Cpt2	Carnitine O-palmitoyltransferase 2, mitochondrial	0,634964	0,301624
820	ST1A1_MOUSE	Sult1a1	Sulfotransferase 1A1	0,41933	-0,0975246
821	ST2A1_MOUSE	Sult2a1	Bile salt sulfotransferase 1	1,50268	-1,80769
822	RL10A_MOUSE	Rpl10a	60S ribosomal protein L10a	0,659023	-0,247018
823	ODB2_MOUSE	Dbt	Lipoamide acyltransferase	0,358194	-0,134444
824	KPYR_MOUSE	Pklr	Pyruvate kinase PKLR	1,09296	0,502551
825	CCHL_MOUSE	Hccs	Cytochrome c-type heme lyase	0,415722	0,534011
826	FDFT_MOUSE	Fdft1	Squalene synthase	0,940004	-1,00433
827	PIPNA_MOUSE	Pitpna	Phosphatidylinositol transfer protein alpha isoform	0,117759	-0,164637

828	PIPNB_MOUSE	Pitpnb	Phosphatidylinositol transfer protein beta isoform	0,488504	0,280813
829	RAB2A_MOUSE	Rab2a	Ras-related protein Rab-2A	0,0238077	0,0204998
830	APC1_MOUSE	Anapc1	Anaphase-promoting complex subunit 1	0,568659	0,291097
831	CNBP_MOUSE	Cnbp	Cellular nucleic acid-binding protein	0,292279	-0,146348
832	IDHP_MOUSE	Idh2	Isocitrate dehydrogenase [NADP], mitochondrial	0,0943124	0,0702194
833	DNJC2_MOUSE	Dnajc2	DnaJ homolog subfamily C member 2	0,682414	0,255461
834	STOM_MOUSE	Stom	Erythrocyte band 7 integral membrane protein	0,216312	0,0656464
835	STMN1_MOUSE	Stmn1	Stathmin	0,0239068	-0,0157696
836	ATX1_MOUSE	Atxn1	Ataxin-1	0,33773	0,274272
837	MSH6_MOUSE	Msh6	DNA mismatch repair protein Msh6	0,551987	0,319207
838	RD23A_MOUSE	Rad23a	UV excision repair protein RAD23 homolog A	0,619667	0,728009
839	RD23B_MOUSE	Rad23b	UV excision repair protein RAD23 homolog B	1,18746	0,526881
840	NUB1_MOUSE	Nub1	NEDD8 ultimate buster 1	0,152105	0,120167
841	FAF1_MOUSE	Faf1	FAS-associated factor 1	0,19996	-0,206559
842	SIA4A_MOUSE	St3gal1	CMP-N-acetylneuraminate-galactosamide-alpha-2,3-transferase 1	0,714789	0,245959
843	EPHB4_MOUSE	Ephb4	Ephrin type-B receptor 4	0,299676	0,374615
844	PRS6B_MOUSE	Psmc4	26S proteasome regulatory subunit 6B	0,827306	0,286354
845	TNG2_MOUSE	Tango2	Transport and Golgi organization 2 homolog	0,673723	-0,19446
846	PUR8_MOUSE	Adsl	Adenylosuccinate lyase	0,217488	0,0937206
847	HMCS2_MOUSE	Hmgcs2	Hydroxymethylglutaryl-CoA synthase, mitochondrial	1,57683	0,63071
848	FABPI_MOUSE	Fabp2	Fatty acid-binding protein, intestinal	2,01673	1,08577
849	ABCD3_MOUSE	Abcd3	ATP-binding cassette sub-family D member 3	1,32677	0,343606
850	CATK_MOUSE	Ctsk	Cathepsin K	0,377428	0,491379
851	RAB8A_MOUSE	Rab8a	Ras-related protein Rab-8A	0,320217	-0,209919
852	ADK_MOUSE	Adk	Adenosine kinase	0,136822	0,147684
853	DSC2_MOUSE	Dsc2	Desmocollin-2	0,774891	0,337595
854	AMRP_MOUSE	Lrpap1	Alpha-2-macroglobulin receptor-associated protein	0,245909	0,09361
855	GOGA3_MOUSE	Golga3	Golgin subfamily A member 3	0,381444	0,273275
856	ATPK_MOUSE	Atp5mf	ATP synthase subunit f, mitochondrial	0,177895	-0,253405
857	ALR_MOUSE	Gfer	FAD-linked sulfhydryl oxidase ALR	1,24765	0,691879
858	AP4A_MOUSE	Nudt2	Bis(5'-nucleosyl)-tetraphosphatase [asymmetrical]	0,138532	0,0731487
859	ATP5E_MOUSE	Atp5f1e	ATP synthase subunit epsilon, mitochondrial	0,566336	0,379455
860	CDD_MOUSE	Cda	Cytidine deaminase	0,124427	-0,1126
861	CX6B1_MOUSE	Cox6b1	Cytochrome c oxidase subunit 6B1	0,657432	0,462669
862	CYB5_MOUSE	Cyb5a	Cytochrome b5	1,34404	-0,555935
863	UBP5_MOUSE	Usp5	Ubiquitin carboxyl-terminal hydrolase 5	2,2577	0,334105
864	ATPB_MOUSE	Atp5f1b	ATP synthase subunit beta, mitochondrial	0,307941	-0,323479
865	CD38_MOUSE	Cd38	ADP-ribosyl cyclase/cyclic ADP-ribose hydrolase 1	1,13933	0,592888
866	CTBP2_MOUSE	Ctbp2	C-terminal-binding protein 2	0,0697556	-0,109276
867	CP2AC_MOUSE	Cyp2a12	Cytochrome P450 2A12	0,0104025	-0,00577812
868	CP237_MOUSE	Cyp2c37	Cytochrome P450 2C37	0,341104	-0,377966
869	CP240_MOUSE	Cyp2c40	Cytochrome P450 2C40	0,854531	0,589268
870	PDCD5_MOUSE	Pdcd5	Programmed cell death protein 5	0,198452	0,131818
871	ZNRD2_MOUSE	Znrd2	Protein ZNRD2	1,24563	0,543735
872	FUS_MOUSE	Fus	RNA-binding protein FUS	0,672092	-0,713767
873	NICA_MOUSE	Ncstn	Nicastrin	0,124567	0,0355549
874	VATD_MOUSE	Atp6v1d	V-type proton ATPase subunit D	0,2644	0,0779591
875	ERP29_MOUSE	Erp29	Endoplasmic reticulum resident protein 29	2,68414	0,610559
876	EF1D_MOUSE	Eef1d	Elongation factor 1-delta	1,29794	0,482358
877	ACTN4_MOUSE	Actn4	Alpha-actinin-4	0,496992	-0,109239
878	RU2A_MOUSE	Snrpa1	U2 small nuclear ribonucleoprotein A'	0,87646	0,366454
879	IDI1_MOUSE	Idi1	Isopentenyl-diphosphate Delta-isomerase 1	0,79739	-0,899167

880	NADK_MOUSE	Nadk	NAD kinase	1,68754	0,340064
881	RT21_MOUSE	Mrps21	28S ribosomal protein S21, mitochondrial	0,629101	0,595452
882	RT06_MOUSE	Mrps6	28S ribosomal protein S6, mitochondrial	0,225827	0,234001
883	ACOT8_MOUSE	Acot8	Acyl-coenzyme A thioesterase 8	1,95304	1,09436
884	EF2_MOUSE	Eef2	Elongation factor 2	0,131434	0,0762619
885	OPA1_MOUSE	Opa1	Dynamin-like 120 kDa protein, mitochondrial	0,731263	0,150128
886	PTPA_MOUSE	Ptpa	Serine/threonine-protein phosphatase 2A activator	0,103292	-0,0805191
887	STRN4_MOUSE	Strn4	Striatin-4	0,0742333	0,0346859
888	GGLO_MOUSE	Gulo	L-gulonolactone oxidase	0,320919	-0,154597
889	S26A1_MOUSE	Slc26a1	Sulfate anion transporter 1	0,639279	-0,324111
890	TPM1_MOUSE	Tpm1	Tropomyosin alpha-1 chain	0,0108084	-0,0120617
891	TPM2_MOUSE	Tpm2	Tropomyosin beta chain	0,0868049	-0,29166
892	TB182_MOUSE	Tnks1bp1	182 kDa tankyrase-1-binding protein	0,831947	0,290382
893	GMEB2_MOUSE	Gmeb2	Glucocorticoid modulatory element-binding protein 2	0,935515	1,13823
894	B2L13_MOUSE	Bcl2l13	Bcl-2-like protein 13	0,545668	-0,162357
895	CING_MOUSE	Cgn	Cingulin	1,19779	0,585118
896	IF5_MOUSE	Eif5	Eukaryotic translation initiation factor 5	0,962469	0,564505
897	SF3B6_MOUSE	Sf3b6	Splicing factor 3B subunit 6	0,953207	0,575989
898	MRTFB_MOUSE	Mrtfb	Myocardin-related transcription factor B	0,331133	0,358149
899	DOCK4_MOUSE	Dock4	Dedicator of cytokinesis protein 4	0,69914	-0,791391
900	ARPC4_MOUSE	Arpc4	Actin-related protein 2/3 complex subunit 4	0,800624	-0,613457
901	SC61G_MOUSE	Sec61g	Protein transport protein Sec61 subunit gamma	0,302838	0,357185
902	RUVB1_MOUSE	Ruvbl1	RuvB-like 1	0,044811	0,0177326
903	EIF3E_MOUSE	Eif3e	Eukaryotic translation initiation factor 3 subunit E	0,333004	0,193718
904	CDO1_MOUSE	Cdo1	Cysteine dioxygenase type 1	0,301	-0,74988
905	PCBP1_MOUSE	Pcbp1	Poly(rC)-binding protein 1	1,28327	0,441629
906	GBRL2_MOUSE	Gabarapl2	Gamma-aminobutyric acid receptor-associated protein-like 2	1,45627	1,38034
907	UB2G2_MOUSE	Ube2g2	Ubiquitin-conjugating enzyme E2 G2	0,163864	-0,378014
908	NPL4_MOUSE	Nploc4	Nuclear protein localization protein 4 homolog	1,35414	0,536668
909	ACTB_MOUSE;ACT	Actb;Actg1	Actin, cytoplasmic 1	0,805744	1,39028
910	CDC42_MOUSE	Cdc42	Cell division control protein 42 homolog	0,232905	0,0804237
911	IF4A1_MOUSE	Eif4a1	Eukaryotic initiation factor 4A-I	0,284641	0,964907
912	RS20_MOUSE	Rps20	40S ribosomal protein S20	1,29066	0,453395
913	CHP1_MOUSE	Chp1	Calcineurin B homologous protein 1	0,633778	0,563835
914	RAB10_MOUSE	Rab10	Ras-related protein Rab-10	1,18394	-0,407579
915	UB2D3_MOUSE	Ube2d3	Ubiquitin-conjugating enzyme E2 D3	0,923255	0,441639
916	UBC12_MOUSE	Ube2m	NEDD8-conjugating enzyme Ubc12	0,849342	0,471022
917	UBE2N_MOUSE	Ube2n	Ubiquitin-conjugating enzyme E2 N	0,143975	-0,0411861
918	ARP2_MOUSE	Actr2	Actin-related protein 2	0,927773	-0,16312
919	ACTZ_MOUSE	Actr1a	Alpha-centractin	0,167559	0,0827705
920	CSN2_MOUSE	Cops2	COP9 signalosome complex subunit 2	0,257468	0,222063
921	ARF1_MOUSE;ARF	Arf1;Arf3	ADP-ribosylation factor 3	0,0966887	0,0915882
922	ABCE1_MOUSE	Abce1	ATP-binding cassette sub-family E member 1	0,628191	0,22043
923	RL26_MOUSE	Rpl26	60S ribosomal protein L26	0,0631544	0,0381027
924	PSME3_MOUSE	Psme3	Proteasome activator complex subunit 3	0,0511018	-0,047644
925	MGN2_MOUSE;MGN	Magoh;Magohb	Protein mago nashi homolog	0,876242	0,2733
926	RL27_MOUSE	Rpl27	60S ribosomal protein L27	0,33024	-0,377678
927	PHS_MOUSE	Pcbd1	Pterin-4-alpha-carbinolamine dehydratase	0,0981851	-0,0576351
928	RL37A_MOUSE	Rpl37a	60S ribosomal protein L37a	0,824995	0,385809
929	PFD3_MOUSE	Vbp1	Prefoldin subunit 3	0,968407	0,340568
930	GABT_MOUSE	Abat	4-aminobutyrate aminotransferase, mitochondrial	2,23367	-0,508974
931	COPZ1_MOUSE	Copz1	Coatomer subunit zeta-1	0,833389	-0,548349

932	DCAF7_MOUSE	Dcaf7	DDB1- and CUL4-associated factor 7	0,678043	0,606327
933	WDR5_MOUSE	Wdr5	WD repeat-containing protein 5	0,575052	0,354172
934	AP1S1_MOUSE	Ap1s1	AP-1 complex subunit sigma-1A	0,0748228	-0,0771732
935	HNRPK_MOUSE	Hnrnpk	Heterogeneous nuclear ribonucleoprotein K	0,676102	0,210752
936	1433G_MOUSE	Ywhag	14-3-3 protein gamma	0,676523	0,202129
937	RRAS2_MOUSE	Rras2	Ras-related protein R-Ras2	0,0303916	-0,0208385
938	TIM10_MOUSE	Timm10	Mitochondrial import inner membrane translocase subunit Tim10	0,291327	0,397927
939	TIM13_MOUSE	Timm13	Mitochondrial import inner membrane translocase subunit Tim13	0,866875	0,221608
940	TIM8B_MOUSE	Timm8b	Mitochondrial import inner membrane translocase subunit Tim8 B	0,689218	0,665804
941	RS7_MOUSE	Rps7	40S ribosomal protein S7	0,565121	0,769315
942	PP1A_MOUSE	Ppp1ca	Serine/threonine-protein phosphatase PP1-alpha catalytic subunit	1,07762	0,158593
943	PRS4_MOUSE	Psmc1	26S proteasome regulatory subunit 4	1,2125	0,362318
944	PRS8_MOUSE	Psmc5	26S proteasome regulatory subunit 8	0,0509748	0,0357399
945	RS8_MOUSE	Rps8	40S ribosomal protein S8	0,773798	0,367237
946	RS15A_MOUSE	Rps15a	40S ribosomal protein S15a	0,574447	0,327444
947	UBE2H_MOUSE	Ube2h	Ubiquitin-conjugating enzyme E2 H	0,135264	-0,147052
948	1433E_MOUSE	Ywhae	14-3-3 protein epsilon	0,42347	-0,310006
949	RS14_MOUSE	Rps14	40S ribosomal protein S14	0,0912769	0,0493095
950	RS23_MOUSE	Rps23	40S ribosomal protein S23	0,573927	0,259471
951	RS18_MOUSE	Rps18	40S ribosomal protein S18	0,437476	0,27763
952	RS29_MOUSE	Rps29	40S ribosomal protein S29	1,44797	0,438507
953	RS11_MOUSE	Rps11	40S ribosomal protein S11	1,27759	0,516235
954	RS13_MOUSE	Rps13	40S ribosomal protein S13	1,25943	0,412474
955	RUXG_MOUSE	Snrpg	Small nuclear ribonucleoprotein G	0,742623	-0,477732
956	LSM6_MOUSE	Lsm6	U6 snRNA-associated Sm-like protein LSm6	0,379844	-0,170747
957	SMD1_MOUSE	Snrpd1	Small nuclear ribonucleoprotein Sm D1	0,382393	-0,379785
958	SMD2_MOUSE	Snrpd2	Small nuclear ribonucleoprotein Sm D2	0,132197	-0,119039
959	SMD3_MOUSE	Snrpd3	Small nuclear ribonucleoprotein Sm D3	2,15404	0,8863
960	ARF6_MOUSE	Arf6	ADP-ribosylation factor 6	0,402198	-0,356991
961	PRS10_MOUSE	Psmc6	26S proteasome regulatory subunit 10B	0,818853	0,37049
962	RS4X_MOUSE	Rps4x	40S ribosomal protein S4, X isoform	1,12715	0,239322
963	PP2AB_MOUSE	Ppp2cb	Serine/threonine-protein phosphatase 2A	0,619731	-0,143289
964	RL18A_MOUSE	Rpl18a	60S ribosomal protein L18a	0,368224	0,261719
965	ACTA_MOUSE;ACTI	Acta2;Actg2	Actin, aortic smooth muscle	0,347065	-0,57949
966	AP2S1_MOUSE	Ap2s1	AP-2 complex subunit sigma	0,538832	1,43843
967	HPCL1_MOUSE	Hpcal1	Hippocalcin-like protein 1	1,03545	-0,684055
968	RL23A_MOUSE	Rpl23a	60S ribosomal protein L23a	0,551517	-0,232844
969	RS6_MOUSE	Rps6	40S ribosomal protein S6	1,06829	0,318949
970	MTPN_MOUSE	Mtpn	Myotrophin	0,588423	-0,438375
971	H4_MOUSE	Hist1h4a	Histone H4	0,952146	1,13535
972	VATB2_MOUSE	Atp6v1b2	V-type proton ATPase subunit B, brain isoform	0,51545	0,228283
973	RAB1A_MOUSE	Rab1A	Ras-related protein Rab-1A	0,353636	0,298883
974	RAN_MOUSE	Ran	GTP-binding nuclear protein Ran	0,125139	-0,0474598
975	RL23_MOUSE	Rpl23	60S ribosomal protein L23	0,335616	0,109594
976	RS15_MOUSE	Rps15	40S ribosomal protein S15	0,398121	0,23345
977	RS24_MOUSE	Rps24	40S ribosomal protein S24	0,186372	-0,0480915
978	RS25_MOUSE	Rps25	40S ribosomal protein S25	0,65025	0,298276
979	RS26_MOUSE	Rps26	40S ribosomal protein S26	0,239277	0,781213
980	RS28_MOUSE	Rps28	40S ribosomal protein S28	0,378216	0,224116
981	RS30_MOUSE	Fau	40S ribosomal protein S30	0,849624	0,130791
982	ELOB_MOUSE	Elob	Elongin-B	0,509598	-0,225853
983	GBB1_MOUSE	Gnb1	Guanine nucleotide-binding protein G(I)/G(S)/G(T) subunit beta-1	0,071411	-0,131123

984	GBB2_MOUSE	Gnb2	Guanine nucleotide-binding protein G(I)/G(S)/G(T) subunit beta-2	0,371465	0,0643772
985	RL30_MOUSE	Rpl30	60S ribosomal protein L30	1,11259	0,415338
986	CYC_MOUSE	Cycs	Cytochrome c, somatic	0,191745	0,0691452
987	RL31_MOUSE	Rpl31	60S ribosomal protein L31	1,21809	0,344746
988	RS3_MOUSE	Rps3	40S ribosomal protein S3	0,199254	-0,0615921
989	RL32_MOUSE	Rpl32	60S ribosomal protein L32	0,0200724	-0,0161999
990	TF2B_MOUSE	Gtf2b	Transcription initiation factor IIB	2,11147	0,561566
991	RL8_MOUSE	Rpl8	60S ribosomal protein L8	0,81311	0,237477
992	YBOX1_MOUSE	Ybx1	Nuclease-sensitive element-binding protein 1	0,102958	0,0665775
993	PROF1_MOUSE	Pfn1	Profilin-1	0,082583	0,09342
994	RS27A_MOUSE	Rps27a	Ubiquitin-40S ribosomal protein S27a	0,984494	-0,712703
995	RL40_MOUSE	Uba52	Ubiquitin-60S ribosomal protein L40	0,228231	0,123164
996	TRA2B_MOUSE	Tra2b	Transformer-2 protein homolog beta	0,30008	0,145908
997	RAC1_MOUSE	Rac1	Ras-related C3 botulinum toxin substrate 1	1,85913	0,418819
998	LIS1_MOUSE	Pafah1b1	Platelet-activating factor acetylhydrolase IB subunit alpha	0,289719	0,198756
999	HSP7C_MOUSE	Hspa8	Heat shock cognate 71 kDa protein	0,0601368	0,049065
1000	TCTP_MOUSE	Tpt1	Translationally-controlled tumor protein	0,445394	0,36352
1001	MPC1_MOUSE	Mpc1	Mitochondrial pyruvate carrier 1	0,0915414	0,0850403
1002	DNJA1_MOUSE	Dnaja1	DnaJ homolog subfamily A member 1	0,120886	-0,118525
1003	CH60_MOUSE	Hspd1	60 kDa heat shock protein, mitochondrial	0,826393	0,697746
1004	IF4E_MOUSE	Eif4e	Eukaryotic translation initiation factor 4E	0,464319	0,429221
1005	MK01_MOUSE	Mapk1	Mitogen-activated protein kinase 1	0,145965	0,0834633
1006	GNAS1_MOUSE;GN	Gnas	Guanine nucleotide-binding protein G(s)	0,266283	-0,140232
1007	1433Z_MOUSE	Ywhaz	14-3-3 protein zeta/delta	0,361558	0,497781
1008	CRNL1_MOUSE	Crnkl1	Crooked neck-like protein 1	1,174	0,330572
1009	HMGB1_MOUSE	Hmgb1	High mobility group protein B1	1,06877	-0,338321
1010	SUMO1_MOUSE	Sumo1	Small ubiquitin-related modifier 1	0,553198	0,323542
1011	IF5A1_MOUSE	Eif5a	Eukaryotic translation initiation factor 5A-1	0,748049	-0,607359
1012	CRIP1_MOUSE	Crip1	Cysteine-rich protein 1	0,0447422	0,0392677
1013	RS17_MOUSE	Rps17	40S ribosomal protein S17	0,188732	-0,149403
1014	UBC9_MOUSE	Ube2i	SUMO-conjugating enzyme UBC9	0,226909	-0,266729
1015	RS12_MOUSE	Rps12	40S ribosomal protein S12	0,386313	0,733858
1016	RS10_MOUSE	Rps10	40S ribosomal protein S10	0,0966937	-0,0705441
1017	PP2BA_MOUSE	Ppp3ca	Serine/threonine-protein phosphatase 2B	0,086299	0,12648
1018	PHB_MOUSE	Phb	Prohibitin	0,0279277	-0,0173332
1019	RL22_MOUSE	Rpl22	60S ribosomal protein L22	0,44644	0,434195
1020	UB2L3_MOUSE	Ube2l3	Ubiquitin-conjugating enzyme E2 L3	0,802828	-0,23306
1021	RACK1_MOUSE	Rack1	Receptor of activated protein C kinase 1	0,907166	0,287641
1022	ACTS_MOUSE	Acta1	Actin, alpha skeletal muscle	0,148196	0,0656902
1023	1433T_MOUSE	Ywhaq	14-3-3 protein theta	0,0419244	-0,029459
1024	TBA4A_MOUSE	Tuba4a	Tubulin alpha-4A chain	0,225195	-0,159658
1025	TBB4B_MOUSE	Tubb4b	Tubulin beta-4B chain	0,799378	0,485301
1026	TBA1C_MOUSE	Tuba1c	Tubulin alpha-1C chain	0,155773	0,0617966
1027	H31_MOUSE;H32_M	Hist1h3a;Hist1h3	Histone H3.1	0,495712	1,20259
1028	1433F_MOUSE	Ywhah	14-3-3 protein eta	0,463849	-0,243127
1029	SBDS_MOUSE	Sbds	Ribosome maturation protein SBDS	1,29528	0,190792
1030	ASM3A_MOUSE	Smpdl3a	Acid sphingomyelinase-like phosphodiesterase 3a	0,526885	-0,393094
1031	IMB1_MOUSE	Kpnb1	Importin subunit beta-1	0,439546	0,351617
1032	CLC4F_MOUSE	Clec4f	C-type lectin domain family 4 member F	1,45086	0,996268
1033	PSB7_MOUSE	Psmb7	Proteasome subunit beta type-7	0,536075	0,106015
1034	PALD_MOUSE	Pald1	Paladin	0,940545	0,291098
1035	F261_MOUSE	Pfkfb1	6-phosphofructo-2-kinase/fructose-2,6-bisphosphatase 1	0,181674	-0,178069

1036	PKN1_MOUSE	Pkn1	Serine/threonine-protein kinase N1	1,16123	0,497885
1037	SEPP1_MOUSE	Selenop	Selenoprotein P	0,724415	-0,51796
1038	SURF6_MOUSE	Surf6	Surfeit locus protein 6	0,723319	0,853632
1039	VAMP7_MOUSE	Vamp7	Vesicle-associated membrane protein 7	0,055936	-0,0925522
1040	EM55_MOUSE	Mpp1	55 kDa erythrocyte membrane protein	0,247198	0,136482
1041	AUP1_MOUSE	Aup1	Ancient ubiquitous protein 1	0,836582	0,196867
1042	PEBP1_MOUSE	Pebp1	Phosphatidylethanolamine-binding protein 1	0,463985	0,116182
1043	STIM1_MOUSE	Stim1	Stromal interaction molecule 1	0,283001	-0,232172
1044	PYRG2_MOUSE	Ctps2	CTP synthase 2	0,575955	0,261981
1045	TIAR_MOUSE	Tial1	Nucleolysin TIAR	0,734758	0,389198
1046	HNRH2_MOUSE	Hnrnph2	Heterogeneous nuclear ribonucleoprotein H2	0,686669	0,722044
1047	ROCK1_MOUSE	Rock1	Rho-associated protein kinase 1	0,0357556	0,0365665
1048	ROCK2_MOUSE	Rock2	Rho-associated protein kinase 2	0,442419	0,332943
1049	HINT1_MOUSE	Hint1	Histidine triad nucleotide-binding protein 1	0,0731062	0,116631
1050	NFYC_MOUSE	Nfyc	Nuclear transcription factor Y subunit gamma	0,310056	0,131784
1051	UFD1_MOUSE	Ufd1	Ubiquitin recognition factor in ER-associated degradation protein 1	0,467284	0,160627
1052	ELAV1_MOUSE	Elavl1	ELAV-like protein 1	0,945536	0,553499
1053	RAD50_MOUSE	Rad50	DNA repair protein RAD50	0,903523	0,404807
1054	ALS_MOUSE	Igfals	Insulin-like growth factor-binding protein complex acid labile subunit	0,186549	0,235966
1055	USP9X_MOUSE	Usp9x	Probable ubiquitin carboxyl-terminal hydrolase FAF-X	0,0692802	-0,045068
1056	IDHG1_MOUSE	Idh3g	Isocitrate dehydrogenase [NAD] subunit gamma 1, mitochondrial	0,0913709	0,152875
1057	NHRF1_MOUSE	Slc9a3r1	Na(+)/H(+) exchange regulatory cofactor NHE-RF1	0,39335	0,356051
1058	BID_MOUSE	Bid	BH3-interacting domain death agonist	0,350267	0,275871
1059	STX4_MOUSE	Stx4	Syntaxin-4	0,391091	0,36964
1060	VASP_MOUSE	Vasp	Vasodilator-stimulated phosphoprotein	0,637461	0,733619
1061	SIAE_MOUSE	Siae	Sialate O-acetylesterase	0,766805	0,401754
1062	NACAM_MOUSE	Naca	Nascent polypeptide-associated complex subunit alpha	0,489817	0,656484
1063	IRF3_MOUSE	Irf3	Interferon regulatory factor 3	0,203465	-0,229988
1064	CASP3_MOUSE	Casp3	Caspase-3	0,299307	0,138949
1065	DHB5_MOUSE	Akr1c6	Estradiol 17 beta-dehydrogenase 5	0,0755161	-0,0919426
1066	DCUP_MOUSE	Urod	Uroporphyrinogen decarboxylase	0,585837	0,314928
1067	PYRG1_MOUSE	Ctps1	CTP synthase 1	0,335203	0,296947
1068	LYAG_MOUSE	Gaa	Lysosomal alpha-glucosidase	0,265861	-0,129582
1069	TCPH_MOUSE	Cct7	T-complex protein 1 subunit eta	1,09223	0,276238
1070	TCPB_MOUSE	Cct2	T-complex protein 1 subunit beta	0,382396	0,228069
1071	TCPD_MOUSE	Cct4	T-complex protein 1 subunit delta	0,893581	0,300241
1072	TCPE_MOUSE	Cct5	T-complex protein 1 subunit epsilon	1,61163	0,511111
1073	TCPZ_MOUSE	Cct6a	T-complex protein 1 subunit zeta	0,995818	0,367902
1074	TCPG_MOUSE	Cct3	T-complex protein 1 subunit gamma	1,55866	0,805191
1075	NUCB2_MOUSE	Nucb2	Nucleobindin-2	0,258437	0,398416
1076	ATF1_MOUSE;CREI	Atf1;Creb1	Cyclic AMP-dependent transcription factor ATF-1	0,489037	-0,389467
1077	BGH3_MOUSE	Tgfbi	Transforming growth factor-beta-induced protein ig-h3	0,211497	0,273572
1078	STIM2_MOUSE	Stim2	Stromal interaction molecule 2	0,759199	0,721299
1079	WNK1_MOUSE	Wnk1	Serine/threonine-protein kinase WNK1	1,32958	0,667242
1080	PHF5A_MOUSE	Phf5a	PHD finger-like domain-containing protein 5A	0,259047	-0,172996
1081	RL36A_MOUSE	Rpl36a	60S ribosomal protein L36a	0,605911	-0,496797
1082	TBG1_MOUSE;TBG:	Tubg1;Tubg2	Tubulin gamma-1 chain	0,705546	0,402715
1083	CBX1_MOUSE	Cbx1	Chromobox protein homolog 1	0,538573	-0,286384
1084	ELOC_MOUSE	Eloc	Elongin-C	0,813543	-0,259258
1085	ARF5_MOUSE	Arf5	ADP-ribosylation factor 5	0,277885	-0,202694
1086	ERH_MOUSE	Erh	Enhancer of rudimentary homolog	0,0843782	-0,118852
1087	AP2M1_MOUSE	Ap2m1	AP-2 complex subunit mu	0,436631	0,30892

1088	RHOG_MOUSE	Rhog	Rho-related GTP-binding protein RhoG	1,75651	0,617971
1089	RL19_MOUSE	Rpl19	60S ribosomal protein L19	0,622684	0,360911
1090	SRSF3_MOUSE	Srsf3	Serine/arginine-rich splicing factor 3	1,45995	0,490135
1091	ISC2A_MOUSE	Isoc2a	Isochorismatase domain-containing protein 2A	0,565091	-0,41957
1092	IC1_MOUSE	Serping1	Plasma protease C1 inhibitor	0,465845	-0,227561
1093	PEDF_MOUSE	Serpinf1	Pigment epithelium-derived factor	1,33223	0,624557
1094	NPTN_MOUSE	Nptn	Neuroplastin	0,546344	0,332067
1095	MCM2_MOUSE	Mcm2	DNA replication licensing factor MCM2	0,974398	0,53157
1096	MCM6_MOUSE	Mcm6	DNA replication licensing factor MCM6	1,08117	0,8807
1097	PRKDC_MOUSE	Prkdc	DNA-dependent protein kinase catalytic subunit	0,361535	0,289256
1098	CSRP2_MOUSE	Csrp2	Cysteine and glycine-rich protein 2	0,336915	0,204052
1099	CSRP1_MOUSE	Csrp1	Cysteine and glycine-rich protein 1	0,560418	-0,499004
1100	KHK_MOUSE	Khk	Ketohexokinase	0,371831	-0,149136
1101	NRP1_MOUSE	Nrp1	Neuropilin-1	1,85797	1,46364
1102	RS3A_MOUSE	Rps3a	40S ribosomal protein S3a	0,859484	0,196778
1103	S10AD_MOUSE	S100a13	Protein S100-A13	1,20084	-0,220601
1104	SPS2_MOUSE	Sephs2	Selenide, water dikinase 2	0,0903354	-0,0885006
1105	EVI5_MOUSE	Evi5	Ecotropic viral integration site 5 protein	0,789205	0,347976
1106	AT1B3_MOUSE	Atp1b3	Sodium/potassium-transporting ATPase subunit beta-3	0,458821	-0,506544
1107	PSME1_MOUSE	Psme1	Proteasome activator complex subunit 1	0,0408598	-0,0195976
1108	PSME2_MOUSE	Psme2	Proteasome activator complex subunit 2	0,0701529	-0,0425251
1109	FRG1_MOUSE	Frg1	Protein FRG1	0,0430287	-0,0643356
1110	G3BP2_MOUSE	G3bp2	Ras GTPase-activating protein-binding protein 2	0,336561	0,238861
1111	ANX11_MOUSE	Anxa11	Annexin A11	0,227491	-0,0798107
1112	DNLI3_MOUSE	Lig3	DNA ligase 3	1,05028	0,274915
1113	VPS45_MOUSE	Vps45	Vacuolar protein sorting-associated protein 45	0,00846605	0,00720558
1114	ANXA4_MOUSE	Anxa4	Annexin A4	0,786495	0,206748
1115	MPRIP_MOUSE	Mprip	Myosin phosphatase Rho-interacting protein	0,908767	0,453164
1116	AMPN_MOUSE	Anpep	Aminopeptidase N	0,527982	0,318377
1117	ATP5J_MOUSE	Atp5pf	ATP synthase-coupling factor 6, mitochondrial	0,263626	-0,169563
1118	BOP1_MOUSE	Bop1	Ribosome biogenesis protein BOP1	1,26566	0,351085
1119	RS5_MOUSE	Rps5	40S ribosomal protein S5	0,152957	-0,0800312
1120	COQ7_MOUSE	Coq7	5-demethoxyubiquinone hydroxylase, mitochondrial	0,119074	0,0968204
1121	EYA3_MOUSE	Eya3	Eyes absent homolog 3	0,0772602	-0,103836
1122	GSH1_MOUSE	Gclc	Glutamate--cysteine ligase catalytic subunit	1,53964	0,735316
1123	SMRC1_MOUSE	Smarcc1	SWI/SNF complex subunit SMARCC1	0,667756	-0,949657
1124	FMO3_MOUSE	Fmo3	Dimethylaniline monooxygenase [N-oxide-forming] 3	0,952057	-0,521083
1125	CPT1A_MOUSE	Cpt1a	Carnitine O-palmitoyltransferase 1, liver isoform	0,45667	-0,594932
1126	RPB3_MOUSE	Polr2c	DNA-directed RNA polymerase II subunit RPB3	0,230977	0,266998
1127	WBP2_MOUSE	Wbp2	WW domain-binding protein 2	0,388119	0,29697
1128	THUM3_MOUSE	Thumpd3	THUMP domain-containing protein 3	1,53495	0,537134
1129	CRY1_MOUSE	Cry1	Cryptochrome-1	0,129492	0,107927
1130	XRN1_MOUSE	Xrn1	5'-3' exoribonuclease 1	0,084519	0,10253
1131	CXAR_MOUSE	Cxadr	Coxsackievirus and adenovirus receptor homolog	0,414544	0,109221
1132	FUMH_MOUSE	Fh	Fumarate hydratase, mitochondrial	0,656427	-0,411333
1133	PLPL9_MOUSE	Pla2g6	85/88 kDa calcium-independent phospholipase A2	0,702924	0,77163
1134	CATC_MOUSE	Ctsc	Dipeptidyl peptidase 1	0,752126	-0,370078
1135	AN32E_MOUSE	Anp32e	Acidic leucine-rich nuclear phosphoprotein 32 family member E	0,619466	0,460549
1136	LYPA1_MOUSE	Lypla1	Acyl-protein thioesterase 1	0,16245	-0,0664707
1137	G3BP1_MOUSE	G3bp1	Ras GTPase-activating protein-binding protein 1	0,0440298	-0,0129841
1138	CASP7_MOUSE	Casp7	Caspase-7	1,13018	-0,614421
1139	FMO5_MOUSE	Fmo5	Dimethylaniline monooxygenase [N-oxide-forming] 5	0,0571319	-0,0397041

1140	LAMA4_MOUSE	Lama4	Laminin subunit alpha-4	0,123244	-0,111898
1141	KTHY_MOUSE	Dtymk	Thymidylate kinase	1,02899	-0,223291
1142	DAB2_MOUSE	Dab2	Disabled homolog 2	0,176448	-0,109771
1143	SHC1_MOUSE	Shc1	SHC-transforming protein 1	0,41495	0,320743
1144	ARVC_MOUSE	Arvcf	Armadillo repeat protein	0,952548	0,549466
1145	TBB5_MOUSE	Tubb5	Tubulin beta-5 chain	0,620391	0,221047
1146	GFRP_MOUSE	Gchfr	GTP cyclohydrolase 1 feedback regulatory protein	0,159515	-0,0947067
1147	PSB4_MOUSE	Psmb4	Proteasome subunit beta type-4	0,169809	0,136753
1148	RLA2_MOUSE	Rplp2	60S acidic ribosomal protein P2	0,547803	-0,34024
1149	QCR6_MOUSE	Uqcrh	Cytochrome b-c1 complex subunit 6, mitochondrial	0,604251	0,402778
1150	PRDX5_MOUSE	Prdx5	Peroxiredoxin-5, mitochondrial	1,59725	0,345524
1151	XDH_MOUSE	Xdh	Xanthine dehydrogenase/oxidase	0,624322	0,366488
1152	F8I2_MOUSE	F8a1	Factor VIII intron 22 protein	0,162965	0,142015
1153	IL6RB_MOUSE	Il6st	Interleukin-6 receptor subunit beta	1,4576	0,563505
1154	G6PD1_MOUSE	G6pdx	Glucose-6-phosphate 1-dehydrogenase X	0,816994	0,45404
1155	APOA1_MOUSE	Apoa1	Apolipoprotein A-I	0,189982	0,232864
1156	RET4_MOUSE	Rbp4	Retinol-binding protein 4	0,884551	0,618248
1157	A1AT4_MOUSE	Serpina1d	Alpha-1-antitrypsin 1-4	0,237954	-0,101341
1158	A1AT5_MOUSE	Serpina1e	Alpha-1-antitrypsin 1-5	1,02456	1,8324
1159	TYY1_MOUSE	Yy1	Transcriptional repressor protein YY1	0,184106	0,2281
1160	RET1_MOUSE	Rbp1	Retinol-binding protein 1	0,501533	0,350504
1161	HNRL2_MOUSE	Hnrnpul2	Heterogeneous nuclear ribonucleoprotein U-like protein 2	0,709613	0,279716
1162	CO1A2_MOUSE	Col1a2	Collagen alpha-2(I) chain	0,00236105	-0,00585289
1163	HMDH_MOUSE	Hmgcr	3-hydroxy-3-methylglutaryl-coenzyme A reductase	0,319715	-0,645862
1164	EGFR_MOUSE	Egfr	Epidermal growth factor receptor	0,103655	-0,101654
1165	APOH_MOUSE	Apoh	Beta-2-glycoprotein 1	0,669361	0,294164
1166	SC23A_MOUSE	Sec23a	Protein transport protein Sec23A	0,632073	-0,23138
1167	RSU1_MOUSE	Rsu1	Ras suppressor protein 1	0,149837	0,0575222
1168	NDKB_MOUSE	Nme2	Nucleoside diphosphate kinase B	0,492713	0,292206
1169	TERA_MOUSE	Vcp	Transitional endoplasmic reticulum ATPase	0,68336	0,381658
1170	UBA1_MOUSE	Uba1	Ubiquitin-like modifier-activating enzyme 1	0,535454	-0,147116
1171	CTNB1_MOUSE	Ctnnb1	Catenin beta-1	0,465919	0,254321
1172	PLAK_MOUSE	Jup	Junction plakoglobin	0,00755789	-0,00922432
1173	ANK1_MOUSE	Ank1	Ankyrin-1	0,407021	0,454671
1174	NFIA_MOUSE	Nfia	Nuclear factor 1 A-type	0,697715	-0,572563
1175	CO6A2_MOUSE	Col6a2	Collagen alpha-2(VI) chain	0,00101957	-0,00117188
1176	NUCB1_MOUSE	Nucb1	Nucleobindin-1	0,0475837	0,0859768
1177	MARK3_MOUSE	Mark3	MAP/microtubule affinity-regulating kinase 3	0,130338	-0,0924965
1178	GRB7_MOUSE	Grb7	Growth factor receptor-bound protein 7	0,780184	0,521473
1179	GALT_MOUSE	Galt	Galactose-1-phosphate uridylyltransferase	0,179613	-0,0964237
1180	ATPA_MOUSE	Atp5f1a	ATP synthase subunit alpha, mitochondrial	0,0324449	-0,0244289
1181	CHLE_MOUSE	Bche	Cholinesterase	0,109109	0,050177
1182	SPA3M_MOUSE	Serpina3m	Serine protease inhibitor A3M	0,142492	0,211989
1183	PFD6_MOUSE	Pfdn6	Prefoldin subunit 6	0,500581	0,264215
1184	E2AK2_MOUSE	Eif2ak2	Interferon-induced, double-stranded RNA-activated protein kinase	2,37351	0,893211
1185	TF65_MOUSE	Rela	Transcription factor p65	0,474178	0,697023
1186	KCRB_MOUSE	Ckb	Creatine kinase B-type	0,34339	0,209767
1187	ASM_MOUSE	Smpd1	Sphingomyelin phosphodiesterase	1,05603	-0,543592
1188	YES_MOUSE	Yes1	Tyrosine-protein kinase Yes	0,767819	0,343647
1189	TOP1_MOUSE	Top1	DNA topoisomerase 1	1,85	0,228048
1190	CO6A1_MOUSE	Col6a1	Collagen alpha-1(VI) chain	0,083443	0,0980663
1191	APOC2_MOUSE	Apoc2	Apolipoprotein C-II	0,370876	0,289103

1192	PPA5_MOUSE	Acp5	Tartrate-resistant acid phosphatase type 5	2,16702	0,766495
1193	RCN1_MOUSE	Rcn1	Reticulocalbin-1	0,619207	-0,516655
1194	CP2E1_MOUSE	Cyp2e1	Cytochrome P450 2E1	0,503709	-0,456622
1195	MARK2_MOUSE	Mark2	Serine/threonine-protein kinase MARK2	0,908969	0,229584
1196	PGBM_MOUSE	Hspg2	Basement membrane-specific heparan sulfate	0,31316	0,13669
1197	FABP5_MOUSE	Fabp5	Fatty acid-binding protein 5	0,2637	-0,297083
1198	GCH1_MOUSE	Gch1	GTP cyclohydrolase 1	0,23829	-0,122496
1199	PYC_MOUSE	Pc	Pyruvate carboxylase, mitochondrial	0,469268	0,24603
1200	LARP7_MOUSE	Larp7	La-related protein 7	0,693896	0,144197
1201	IF2P_MOUSE	Eif5b	Eukaryotic translation initiation factor 5B	0,623421	-0,419026
1202	CAB39_MOUSE	Cab39	Calcium-binding protein 39	0,598496	-0,27179
1203	PTN2_MOUSE	Ptpn2	Tyrosine-protein phosphatase non-receptor type 2	1,65136	1,78642
1204	ATP5I_MOUSE	Atp5me	ATP synthase subunit e, mitochondrial	0,478748	0,129946
1205	CLUS_MOUSE	Clu	Clusterin	0,87946	0,730743
1206	ANXA7_MOUSE	Anxa7	Annexin A7	1,43946	0,366464
1207	MPRI_MOUSE	Igf2r	Cation-independent mannose-6-phosphate receptor	0,263046	0,218198
1208	ACADS_MOUSE	Acads	Short-chain specific acyl-CoA dehydrogenase, mitochondrial	0,957331	0,243199
1209	AMBP_MOUSE	Ambp	Protein AMBP	0,687545	0,264369
1210	LG3BP_MOUSE	Lgals3bp	Galectin-3-binding protein	1,21183	0,846291
1211	F13B_MOUSE	F13b	Coagulation factor XIII B chain	0,660334	0,465073
1212	CNN2_MOUSE	Cnn2	Calponin-2	0,613331	0,306371
1213	DMWD_MOUSE	Dmwd	Dystrophia myotonica WD repeat-containing protein	1,91655	0,999999
1214	LYAR_MOUSE	Lyar	Cell growth-regulating nucleolar protein	0,524818	-0,431332
1215	CD36_MOUSE	Cd36	Platelet glycoprotein 4	0,882212	0,420734
1216	SSRP1_MOUSE	Ssrp1	FACT complex subunit SSRP1	0,155255	0,150812
1217	B4GN1_MOUSE	B4galnt1	Beta-1,4 N-acetylgalactosaminyltransferase 1	0,612233	-0,292588
1218	INF2_MOUSE	Inf2	Inverted formin-2	0,285669	-0,168927
1219	ZCH18_MOUSE	Zc3h18	Zinc finger CCCH domain-containing protein 18	0,0440094	0,0620926
1220	RBM15_MOUSE	Rbm15	RNA-binding protein 15	0,197146	0,202017
1221	PP4R2_MOUSE	Ppp4r2	Serine/threonine-protein phosphatase 4 regulatory subunit 2	0,995843	0,488546
1222	PSA_MOUSE	Npepps	Puromycin-sensitive aminopeptidase	0,766179	0,484448
1223	PEPD_MOUSE	Pepd	Xaa-Pro dipeptidase	0,0263474	-0,0126221
1224	RELCH_MOUSE	Relch	RAB11-binding protein RELCH	0,787197	0,624789
1225	FA83H_MOUSE	Fam83h	Protein FAM83H	0,0542656	0,0511635
1226	RUSD2_MOUSE	Rpusd2	RNA pseudouridylate synthase domain-containing protein 2	0,0304065	0,0405983
1227	RUSD3_MOUSE	Rpusd3	Mitochondrial mRNA pseudouridine synthase Rpusd3	0,33948	0,385613
1228	PTCD3_MOUSE	Ptcd3	Pentatricopeptide repeat domain-containing protein 3	0,0826111	0,0542076
1229	MOCOS_MOUSE	Mocos	Molybdenum cofactor sulfurase	0,364508	0,32368
1230	SYAM_MOUSE	Aars2	Alanine--tRNA ligase, mitochondrial	0,215257	0,166898
1231	ACSS3_MOUSE	Acss3	Acyl-CoA synthetase short-chain family member 3, mitochondrial	0,285537	0,19015
1232	NSUN2_MOUSE	Nsun2	tRNA (cytosine(34)-C(5))-methyltransferase	0,434323	0,0975731
1233	URAD_MOUSE	Urad	2-oxo-4-hydroxy-4-carboxy-5-ureidoimidazoline decarboxylase	0,138384	0,120243
1234	PAR14_MOUSE	Parp14	Protein mono-ADP-ribosyltransferase PARP14	0,494694	0,31038
1235	PSD3_MOUSE	Psd3	PH and SEC7 domain-containing protein 3	0,395998	0,385712
1236	P4K2A_MOUSE	Pi4k2a	Phosphatidylinositol 4-kinase type 2-alpha	0,16523	-0,146598
1237	HSDL2_MOUSE	Hsdl2	Hydroxysteroid dehydrogenase-like protein 2	2,10233	1,04152
1238	C163A_MOUSE	Cd163	Scavenger receptor cysteine-rich type 1 protein M130	0,408815	-0,611503
1239	CNNM3_MOUSE	Cnnm3	Metal transporter CNNM3	0,06794	-0,0989683
1240	OSBP1_MOUSE	Osbp	Oxysterol-binding protein 1	0,279269	0,0932957
1241	COL11_MOUSE	Colec11	Collectin-11	0,870047	0,601713
1242	HMHA1_MOUSE	Arhgap45	Rho GTPase-activating protein 45	1,06138	-0,639313
1243	RM10_MOUSE	Mrpl10	39S ribosomal protein L10, mitochondrial	0,532585	0,185496

1244	PATL1_MOUSE	Patl1	Protein PAT1 homolog 1	0,21726	0,206663
1245	FAHD2_MOUSE	Fahd2	Fumarylacetoacetate hydrolase domain-containing protein 2A	0,185566	0,175338
1246	CUL4A_MOUSE	Cul4a	Cullin-4A	0,918651	-0,17667
1247	ABRX2_MOUSE	Abraxas2	BRISC complex subunit Abraxas 2	0,339354	0,166691
1248	KHDC4_MOUSE	Khdc4	KH homology domain-containing protein 4	1,2389	0,630685
1249	PPR21_MOUSE	Ppp1r21	Protein phosphatase 1 regulatory subunit 21	0,0211914	-0,0259899
1250	STT3B_MOUSE	Stt3b	Dolichyl-diphosphooligosaccharide--protein glycosyltransferase	0,155304	0,256131
1251	HP1B3_MOUSE	Hp1bp3	Heterochromatin protein 1-binding protein 3	0,0626201	-0,051107
1252	F107B_MOUSE	Fam107b	Protein FAM107B	0,536681	0,157964
1253	EEPD1_MOUSE	Eepd1	Endonuclease/exonuclease/phosphatase	0,120335	0,103101
1254	ML12B_MOUSE	Myl12b	Myosin regulatory light chain 12B	0,110991	0,174443
1255	AASD1_MOUSE	Aarsd1	Alanyl-tRNA editing protein Aarsd1	0,865295	0,372844
1256	GUAA_MOUSE	Gmps	GMP synthase [glutamine-hydrolyzing]	0,164502	0,136295
1257	METK2_MOUSE	Mat2a	S-adenosylmethionine synthase isoform type-2	1,04845	0,296633
1258	TPC13_MOUSE	Trappc13	Trafficking protein particle complex subunit 13	1,22374	0,557424
1259	PDE12_MOUSE	Pde12	2',5'-phosphodiesterase 12	0,434295	0,595287
1260	ZC3HF_MOUSE	Zc3h15	Zinc finger CCCH domain-containing protein 15	0,348031	0,158199
1261	SNUT2_MOUSE	Usp39	U4/U6.U5 tri-snRNP-associated protein 2	0,649171	0,968504
1262	L2GL2_MOUSE	Llgl2	Lethal(2) giant larvae protein homolog 2	0,651129	0,473741
1263	FA98A_MOUSE	Fam98a	Protein FAM98A	0,30923	-0,400238
1264	SMCA4_MOUSE	Smarca4	Transcription activator BRG1	0,28238	0,169258
1265	NLRX1_MOUSE	Nlrx1	NLR family member X1	0,236019	0,0810276
1266	PRC2C_MOUSE	Prrc2c	Protein PRRC2C	0,712637	0,349232
1267	ECHD2_MOUSE	Echdc2	Enoyl-CoA hydratase domain-containing protein 2, mitochondrial	0,0934369	0,079974
1268	SCRN3_MOUSE	Scrn3	Secernin-3	0,702039	0,338531
1269	XYLB_MOUSE	Xylb	Xylulose kinase	0,600475	0,479066
1270	EXOC5_MOUSE	Exoc5	Exocyst complex component 5	0,121269	0,107022
1271	FXRD1_MOUSE	Foxred1	FAD-dependent oxidoreductase domain-containing protein 1	0,217651	-0,242158
1272	HXK3_MOUSE	Hk3	Hexokinase-3	0,158932	0,155536
1273	TAM41_MOUSE	Tamm41	Phosphatidate cytidylyltransferase, mitochondrial	0,182305	-0,0877056
1274	UAP1L_MOUSE	Uap1l1	UDP-N-acetylhexosamine pyrophosphorylase-like protein 1	1,13553	0,88073
1275	SRSF6_MOUSE	Srsf6	Serine/arginine-rich splicing factor 6	0,849479	0,303596
1276	TRM1_MOUSE	Trmt1	tRNA (guanine(26)-N(2))-dimethyltransferase	0,715349	0,568663
1277	PSMD1_MOUSE	Psmd1	26S proteasome non-ATPase regulatory subunit 1	1,21252	0,370178
1278	DHYS_MOUSE	Dhps	Deoxyhypusine synthase	0,140487	-0,111684
1279	SMYD5_MOUSE	Smyd5	SET and MYND domain-containing protein 5	0,2258	0,444133
1280	ESYT2_MOUSE	Esyt2	Extended synaptotagmin-2	1,57653	-0,852337
1281	DHR11_MOUSE	Dhrs11	Dehydrogenase/reductase SDR family member 11	0,385641	-0,109931
1282	TBD2B_MOUSE	Tbc1d2b	TBC1 domain family member 2B	1,06623	1,02212
1283	RAIN_MOUSE	Rasip1	Ras-interacting protein 1	0,124257	0,120311
1284	FUBP2_MOUSE	Khsrp	Far upstream element-binding protein 2	1,73086	0,380115
1285	TRADD_MOUSE	Tradd	Tumor necrosis factor receptor type 1	1,35951	0,597411
1286	SYRM_MOUSE	Rars2	Probable arginine--tRNA ligase, mitochondrial	0,110968	0,13664
1287	DDB1_MOUSE	Ddb1	DNA damage-binding protein 1	0,8232	0,225137
1288	SYVM_MOUSE	Vars2	Valine--tRNA ligase, mitochondrial	0,0331981	0,0204647
1289	SC24A_MOUSE	Sec24a	Protein transport protein Sec24A	0,1464	-0,0827675
1290	CTU2_MOUSE	Ctu2	Cytoplasmic tRNA 2-thiolation protein 2	1,61035	0,429623
1291	BRE1B_MOUSE	Rnf40	E3 ubiquitin-protein ligase BRE1B	0,0354901	-0,0274719
1292	HECD3_MOUSE	Hectd3	E3 ubiquitin-protein ligase HECTD3	0,173514	0,186105
1293	CMPK2_MOUSE	Cmpk2	UMP-CMP kinase 2, mitochondrial	1,36656	0,470609
1294	ESYT1_MOUSE	Esyt1	Extended synaptotagmin-1	0,248977	-0,0927032
1295	LBR_MOUSE	Lbr	Delta(14)-sterol reductase LBR	0,077366	0,0809948

1296	PCLI1_MOUSE	Pid1	PTB-containing, cubilin and LRP1-interacting protein	1,12221	-1,47058
1297	OTUL_MOUSE	Otulin	Ubiquitin thioesterase otulin	0,252634	0,364414
1298	TTL12_MOUSE	Ttll12	Tubulin--tyrosine ligase-like protein 12	0,559571	0,441391
1299	TRAD1_MOUSE	Trafd1	TRAF-type zinc finger domain-containing protein 1	0,571338	0,638845
1300	YIPF3_MOUSE	Yipf3	Protein YIPF3	0,634565	0,401754
1301	UBE2Z_MOUSE	Ube2z	Ubiquitin-conjugating enzyme E2 Z	1,09483	0,411574
1302	PUF60_MOUSE	Puf60	Poly(U)-binding-splicing factor PUF60	0,385571	0,129522
1303	AGT2_MOUSE	Agxt2	Alanine--glyoxylate aminotransferase 2, mitochondrial	0,188655	0,105821
1304	TM10C_MOUSE	Trmt10c	tRNA methyltransferase 10 homolog C	0,310764	-0,178244
1305	EI3JA_MOUSE;EI3JI	Eif3j1;Eif3j2	Eukaryotic translation initiation factor 3 subunit J-A	0,411276	-0,235991
1306	HDHD2_MOUSE	Hdhd2	Haloacid dehalogenase-like hydrolase	0,32329	-0,306723
1307	MCRI1_MOUSE	Mcrip1	Mapk-regulated corepressor-interacting protein 1	1,37047	-0,460471
1308	DIP2B_MOUSE	Dip2b	Disco-interacting protein 2 homolog B	0,988215	0,404195
1309	SPTCS_MOUSE	Spg11	Spatacsin	0,781032	0,434568
1310	SNX27_MOUSE	Snx27	Sorting nexin-27	0,276801	0,241193
1311	NDST1_MOUSE	Ndst1	Bifunctional heparan sulfate N-deacetylase/N-sulfotransferase 1	0,583319	0,382758
1312	HAP28_MOUSE	Pdap1	28 kDa heat- and acid-stable phosphoprotein	1,13202	0,687075
1313	BABA1_MOUSE	Babam1	BRISC and BRCA1-A complex member 1	0,761938	0,264867
1314	RHG17_MOUSE	Arhgap17	Rho GTPase-activating protein 17	1,01588	2,29145
1315	PKHA7_MOUSE	Plekha7	Pleckstrin homology domain-containing family A member 7	0,220172	-0,174586
1316	DTX3L_MOUSE	Dtx3l	E3 ubiquitin-protein ligase DTX3L	1,68277	0,718784
1317	NDUB6_MOUSE	Ndufb6	NADH dehydrogenase [ubiquinone] 1 beta subcomplex subunit 6	1,40442	-0,651532
1318	EDC4_MOUSE	Edc4	Enhancer of mRNA-decapping protein 4	0,450821	0,161314
1319	UBP19_MOUSE	Usp19	Ubiquitin carboxyl-terminal hydrolase 19	1,09933	0,291514
1320	RMD3_MOUSE	Rmdn3	Regulator of microtubule dynamics protein 3	0,189106	-0,097644
1321	SMU1_MOUSE	Smu1	WD40 repeat-containing protein SMU1	0,106164	0,0524639
1322	ARGL1_MOUSE	Arglu1	Arginine and glutamate-rich protein 1	0,181096	-0,247371
1323	MCCB_MOUSE	Mccc2	Methylcrotonoyl-CoA carboxylase beta chain, mitochondrial	0,360739	0,444063
1324	SPG7_MOUSE	Spg7	Paraplegin	0,26835	0,190906
1325	GPD1L_MOUSE	Gpd1l	Glycerol-3-phosphate dehydrogenase 1-like protein	0,712172	-0,399483
1326	PARP3_MOUSE	Parp3	Protein mono-ADP-ribosyltransferase PARP3	0,718808	0,536603
1327	COG7_MOUSE	Cog7	Conserved oligomeric Golgi complex subunit 7	0,889188	-0,615666
1328	PP1R7_MOUSE	Ppp1r7	Protein phosphatase 1 regulatory subunit 7	0,233964	0,140891
1329	WASC4_MOUSE	Washc4	WASH complex subunit 4	0,0695338	0,0411556
1330	AFG2H_MOUSE	Spata5	ATPase family protein 2 homolog	0,545249	-0,824794
1331	COBL1_MOUSE	Cobll1	Cordon-bleu protein-like 1	0,187716	0,158698
1332	PP12C_MOUSE	Ppp1r12c	Protein phosphatase 1 regulatory subunit 12C	0,238942	0,124968
1333	HDGR2_MOUSE	Hdgfl2	Hepatoma-derived growth factor-related protein 2	0,0971306	0,0595612
1334	EMAL4_MOUSE	Eml4	Echinoderm microtubule-associated protein-like 4	0,475451	0,200934
1335	UBP30_MOUSE	Usp30	Ubiquitin carboxyl-terminal hydrolase 30	0,0848293	0,12725
1336	ACSM3_MOUSE	Acsm3	Acyl-coenzyme A synthetase ACSM3, mitochondrial	0,299335	0,0707195
1337	QORL2_MOUSE	Cryzl2	Quinone oxidoreductase-like protein 2	0,488865	-0,402311
1338	ZCCHV_MOUSE	Zc3hav1	Zinc finger CCCH-type antiviral protein 1	1,56015	0,37578
1339	PRRC1_MOUSE	Prrc1	Protein PRRC1	0,072414	0,0156414
1340	SC31A_MOUSE	Sec31a	Protein transport protein Sec31A	1,57296	0,49744
1341	PXDN_MOUSE	Pxdn	Peroxidasin homolog	0,169995	0,205529
1342	IQGA2_MOUSE	Iqgap2	Ras GTPase-activating-like protein IQGAP2	0,165624	-0,137827
1343	SYTM_MOUSE	Tars2	Threonine--tRNA ligase, mitochondrial	0,797133	0,42735
1344	TGFA1_MOUSE	Tgfbrap1	Transforming growth factor-beta receptor-associated protein 1	0,424757	0,185596
1345	SLMAP_MOUSE	Slmap	Sarcolemmal membrane-associated protein	0,556825	0,262462
1346	ACSF3_MOUSE	Acsf3	Malonate--CoA ligase ACSF3, mitochondrial	0,950426	0,25889
1347	CCD51_MOUSE	Ccdc51	Coiled-coil domain-containing protein 51	0,00371164	-0,00498581

1348	DIK2A_MOUSE	Dipk2a	Divergent protein kinase domain 2A	1,61489	0,425837
1349	SRBS2_MOUSE	Sorbs2	Sorbin and SH3 domain-containing protein 2	0,0824585	0,036673
1350	THEM4_MOUSE	Them4	Acyl-coenzyme A thioesterase THEM4	1,19024	0,249303
1351	PDP1_MOUSE	Pdp1	[Pyruvate dehydrogenase [acetyl-transferring]]-phosphatase 1	0,541373	0,397614
1352	F91A1_MOUSE	Fam91a1	Protein FAM91A1	0,338778	0,205081
1353	VPS51_MOUSE	Vps51	Vacuolar protein sorting-associated protein 51 homolog	0,29244	0,105132
1354	RGL3_MOUSE	Rgl3	Ral guanine nucleotide dissociation stimulator-like 3	0,895155	0,37708
1355	ST1D1_MOUSE	Sult1d1	Sulfotransferase 1 family member D1	1,34265	-1,00191
1356	PLSI_MOUSE	Pls1	Plastin-1	0,358064	-0,501567
1357	5NTC_MOUSE	Nt5c2	Cytosolic purine 5'-nucleotidase	0,345935	0,119567
1358	P3H1_MOUSE	P3h1	Prolyl 3-hydroxylase 1	1,34611	0,597153
1359	N42L1_MOUSE	N4bp2l1	NEDD4-binding protein 2-like 1	0,0097293	0,0122753
1360	AFG1L_MOUSE	Afg1l	AFG1-like ATPase	1,24999	0,314607
1361	PACS2_MOUSE	Pacs2	Phosphofurin acidic cluster sorting protein 2	1,02043	-0,643868
1362	C1TM_MOUSE	Mthfd1l	Monofunctional C1-tetrahydrofolate synthase, mitochondrial	0,0306198	0,0263893
1363	ITA1_MOUSE	Itga1	Integrin alpha-1	1,99394	0,243674
1364	SRBD1_MOUSE	Srbd1	S1 RNA-binding domain-containing protein 1	0,213879	0,0698277
1365	SHAN3_MOUSE	Shank3	SH3 and multiple ankyrin repeat domains protein 3	1,42118	0,670228
1366	MAGIX_MOUSE	Magix	PDZ domain-containing protein MAGIX	1,07075	0,663602
1367	ABRAL_MOUSE	Abracl	Costars family protein ABRACL	0,389359	0,767012
1368	ARAP1_MOUSE	Arap1	Arf-GAP with Rho-GAP domain, ANK repeat PH domain protein 1	0,177814	-0,114691
1369	S27A5_MOUSE	Slc27a5	Bile acyl-CoA synthetase	0,422434	-0,177873
1370	ODR4_MOUSE	Odr4	Protein odr-4 homolog	2,10116	0,575142
1371	ECE1_MOUSE	Ece1	Endothelin-converting enzyme 1	0,272976	-0,161016
1372	PDS5B_MOUSE	Pds5b	Sister chromatid cohesion protein PDS5 homolog B	0,630761	0,21118
1373	CDV3_MOUSE	Cdv3	Protein CDV3	2,06004	0,524036
1374	DDX17_MOUSE	Ddx17	Probable ATP-dependent RNA helicase DDX17	1,00993	0,266133
1375	ARCH_MOUSE	Zbtb8os	Protein archease	1,55567	0,529103
1376	ANR28_MOUSE	Ankrd28	Serine/threonine-protein phosphatase 6	0,299674	0,202369
1377	OPA3_MOUSE	Opa3	Optic atrophy 3 protein homolog	0,1406	0,112526
1378	LRC47_MOUSE	Lrrc47	Leucine-rich repeat-containing protein 47	1,01514	0,476664
1379	COQ8B_MOUSE	Coq8b	Atypical kinase COQ8B, mitochondrial	0,396435	0,244963
1380	DDX46_MOUSE	Ddx46	Probable ATP-dependent RNA helicase DDX46	0,042078	-0,0186821
1381	TR150_MOUSE	Thrap3	Thyroid hormone receptor-associated protein 3	0,672364	0,35694
1382	DPTOR_MOUSE	Deptor	DEP domain-containing mTOR-interacting protein	0,228382	-0,248802
1383	GALNS_MOUSE	Galns	N-acetylgalactosamine-6-sulfatase	0,168461	0,138951
1384	GLSL_MOUSE	Gls2	Glutaminase liver isoform, mitochondrial	0,7781	0,511504
1385	A16A1_MOUSE	Aldh16a1	Aldehyde dehydrogenase family 16 member A1	0,337992	-0,153871
1386	JIP4_MOUSE	Spag9	C-Jun-amino-terminal kinase-interacting protein 4	1,3904	0,482749
1387	NDUF2_MOUSE	Ndufaf2	NADH dehydrogenase [ubiquinone] 1 alpha	0,139789	0,134005
1388	PEX1_MOUSE	Pex1	Peroxisome biogenesis factor 1	2,73721	0,610834
1389	TUT7_MOUSE	Tut7	Terminal uridylyltransferase 7	0,673335	0,197498
1390	AF1L2_MOUSE	Afap1l2	Actin filament-associated protein 1-like 2	0,0153536	-0,0228073
1391	EHMT1_MOUSE	Ehmt1	Histone-lysine N-methyltransferase EHMT1	1,57997	1,24608
1392	AAPK1_MOUSE	Prkaa1	5'-AMP-activated protein kinase catalytic subunit alpha-1	0,443665	0,197579
1393	NUFP2_MOUSE	Nufip2	Nuclear fragile X mental retardation-interacting protein 2	0,544559	0,334954
1394	TAOK1_MOUSE	Taok1	Serine/threonine-protein kinase TAO1	1,3174	0,771301
1395	ABH15_MOUSE	Abhd15	Protein ABHD15	0,0384016	0,0379238
1396	GLYAL_MOUSE	Gm4952	Glycine N-acyltransferase-like protein	0,572072	0,168914
1397	ARHGF_MOUSE	Arhgef15	Rho guanine nucleotide exchange factor 15	0,141989	0,124475
1398	CEIP2_MOUSE	Cemip2	Cell surface hyaluronidase	0,0116943	0,0122463
1399	RHG01_MOUSE	Arhgap1	Rho GTPase-activating protein 1	0,00760392	-0,00292435

1400	MTUS1_MOUSE	Mtus1	Microtubule-associated tumor suppressor 1 homolog	0,56121	0,449273
1401	VPS41_MOUSE	Vps41	Vacuolar protein sorting-associated protein 41 homolog	0,399022	0,27571
1402	D39U1_MOUSE	Sdr39u1	Epimerase family protein SDR39U1	0,645323	0,48068
1403	COBL_MOUSE	Cobl	Protein cordon-bleu	0,605864	0,26089
1404	TPPC1_MOUSE	Trappc1	Trafficking protein particle complex subunit 1	1,16401	2,12512
1405	RILP_MOUSE	Rilp	Rab-interacting lysosomal protein	0,438542	0,326979
1406	WDR81_MOUSE	Wdr81	WD repeat-containing protein 81	0,346095	0,268232
1407	MRM3_MOUSE	Mrm3	rRNA methyltransferase 3, mitochondrial	1,35863	1,04292
1408	MOCS1_MOUSE	Mocs1	Molybdenum cofactor biosynthesis protein 1	0,313732	0,171193
1409	DHR13_MOUSE	Dhrs13	Dehydrogenase/reductase SDR family member 13	0,398358	0,0797974
1410	ZZEF1_MOUSE	Zzef1	Zinc finger ZZ-type and EF-hand domain-containing protein 1	0,0195792	0,0172768
1411	UTP18_MOUSE	Utp18	U3 small nucleolar RNA-associated protein 18 homolog	0,52677	0,421203
1412	TEFM_MOUSE	Tefm	Transcription elongation factor, mitochondrial	0,125262	-0,0607506
1413	TENS3_MOUSE	Tns3	Tensin-3	0,211595	0,138482
1414	LC7L3_MOUSE	Luc7l3	Luc7-like protein 3	0,523509	0,20957
1415	PUR4_MOUSE	Pfas	Phosphoribosylformylglycinamidine synthase	0,295191	0,176665
1416	TBC9B_MOUSE	Tbc1d9b	TBC1 domain family member 9B	1,05292	-0,505047
1417	CLU_MOUSE	Cluh	Clustered mitochondria protein homolog	0,462019	0,290452
1418	ACACA_MOUSE	Acaca	Acetyl-CoA carboxylase 1	0,130497	0,165536
1419	MYH1_MOUSE	Myh1	Myosin-1	0,189189	0,76111
1420	SHRM1_MOUSE	Shroom1	Protein Shroom1	0,0238618	-0,0302906
1421	MYO1D_MOUSE	Myo1d	Unconventional myosin-Id	0,347755	0,273713
1422	TM199_MOUSE	Tmem199	Transmembrane protein 199	1,26563	1,40623
1423	DJC11_MOUSE	Dnajc11	DnaJ homolog subfamily C member 11	1,41228	0,260859
1424	THOC6_MOUSE	Thoc6	THO complex subunit 6 homolog	1,07173	0,255113
1425	REPI1_MOUSE	Repin1	Replication initiator 1	1,45667	1,12015
1426	BL1S3_MOUSE	Bloc1s3	Biogenesis of lysosome-related organelles complex 1 subunit 3	1,10772	0,890333
1427	HYKK_MOUSE	Hykk	Hydroxylysine kinase	0,185656	0,0874092
1428	ACBD5_MOUSE	Acbd5	Acyl-CoA-binding domain-containing protein 5	0,896178	0,535962
1429	LEO1_MOUSE	Leo1	RNA polymerase-associated protein LEO1	0,823733	0,387057
1430	COPD_MOUSE	Arcn1	Coatomer subunit delta	2,16992	0,370177
1431	RN123_MOUSE	Rnf123	E3 ubiquitin-protein ligase RNF123	1,89641	2,29346
1432	A1CF_MOUSE	A1cf	APOBEC1 complementation factor	0,539176	0,275822
1433	SIN3A_MOUSE	Sin3a	Paired amphipathic helix protein Sin3a	0,149259	0,0855881
1434	KIF1B_MOUSE	Kif1b	Kinesin-like protein KIF1B	0,95737	0,449337
1435	ODO1_MOUSE	Ogdh	2-oxoglutarate dehydrogenase, mitochondrial	0,0850637	0,0843475
1436	SRC8_MOUSE	Cttn	Src substrate cortactin	0,0542541	0,0256618
1437	MYL6_MOUSE	Myl6	Myosin light polypeptide 6	0,60197	-0,48313
1438	GRB2_MOUSE	Grb2	Growth factor receptor-bound protein 2	0,630269	0,415618
1439	FLOT2_MOUSE	Flot2	Flotillin-2	0,832828	-0,656495
1440	CLPB_MOUSE	Clpb	Caseinolytic peptidase B protein homolog	1,04578	0,543115
1441	HNRPD_MOUSE	Hnrnpd	Heterogeneous nuclear ribonucleoprotein D0	1,13935	-0,411162
1442	PPP5_MOUSE	Ppp5c	Serine/threonine-protein phosphatase 5	0,620969	0,169256
1443	IKKA_MOUSE	Chuk	Inhibitor of nuclear factor kappa-B kinase subunit alpha	0,383428	0,301955
1444	PSB6_MOUSE	Psmb6	Proteasome subunit beta type-6	0,0551242	-0,118883
1445	SAMH1_MOUSE	Samhd1	Deoxynucleoside triphosphate triphosphohydrolase SAMHD1	1,12362	0,451586
1446	P4HA1_MOUSE	P4ha1	Prolyl 4-hydroxylase subunit alpha-1	0,833303	0,356029
1447	P4HA2_MOUSE	P4ha2	Prolyl 4-hydroxylase subunit alpha-2	0,173321	0,182391
1448	CSK21_MOUSE	Csnk2a1	Casein kinase II subunit alpha	0,200199	-0,0672451
1449	ZNT1_MOUSE	Slc30a1	Zinc transporter 1	0,0223003	-0,0350685
1450	BAG1_MOUSE	Bag1	BAG family molecular chaperone regulator 1	0,890323	0,527499
1451	KHDR1_MOUSE	Khdrbs1	KH domain-containing, RNA-binding protein 1	0,184289	0,248684

1452	GCDH_MOUSE	Gcdh	Glutaryl-CoA dehydrogenase, mitochondrial	0,180336	0,175732
1453	GRB10_MOUSE	Grb10	Growth factor receptor-bound protein 10	0,170481	0,10004
1454	IRGM1_MOUSE	Irgm1	Immunity-related GTPase family M protein 1	1,02293	-0,888817
1455	STXB3_MOUSE	Stxbp3	Syntaxin-binding protein 3	0,788536	0,31938
1456	CDN2C_MOUSE	Cdkn2c	Cyclin-dependent kinase 4 inhibitor C	1,19062	0,574913
1457	AKT2_MOUSE	Akt2	RAC-beta serine/threonine-protein kinase	0,220632	0,287346
1458	SPB6_MOUSE	Serpinb6	Serpin B6	0,348704	-0,16496
1459	RIPK1_MOUSE	Ripk1	Receptor-interacting serine/threonine-protein kinase 1	0,612213	0,213264
1460	STIP1_MOUSE	Stip1	Stress-induced-phosphoprotein 1	0,70188	0,341416
1461	CAPR1_MOUSE	Caprin1	Caprin-1	0,430059	0,112635
1462	PTER_MOUSE	Pter	Phosphotriesterase-related protein	0,454805	0,16596
1463	IF1A_MOUSE	Eif1a	Eukaryotic translation initiation factor 1A	0,937621	0,778129
1464	EP15R_MOUSE	Eps15l1	Epidermal growth factor receptor substrate 15-like 1	1,06483	0,348477
1465	VDAC2_MOUSE	Vdac2	Voltage-dependent anion-selective channel protein 2	0,33408	0,171128
1466	VDAC3_MOUSE	Vdac3	Voltage-dependent anion-selective channel protein 3	1,87489	-0,714952
1467	VDAC1_MOUSE	Vdac1	Voltage-dependent anion-selective channel protein 1	0,0259101	0,0165493
1468	COQ8A_MOUSE	Coq8a	Atypical kinase COQ8A, mitochondrial	0,522051	-0,356282
1469	PML_MOUSE	Pml	Protein PML	0,557488	-0,463934
1470	IMA5_MOUSE	Kpna1	Importin subunit alpha-5	1,3435	-1,33989
1471	PAPS1_MOUSE	Papss1	Bifunctional 3'-phosphoadenosine 5'-phosphosulfate synthase 1	1,43033	0,565094
1472	RBBP4_MOUSE	Rbbp4	Histone-binding protein RBBP4	0,129832	0,0858654
1473	RBBP7_MOUSE	Rbbp7	Histone-binding protein RBBP7	1,44021	0,132182
1474	NCOR1_MOUSE	Ncor1	Nuclear receptor corepressor 1	0,64623	0,425904
1475	2A5G_MOUSE	Ppp2r5c	Serine/threonine-protein phosphatase 2A	0,223566	0,293069
1476	LAMA5_MOUSE	Lama5	Laminin subunit alpha-5	0,0114692	-0,0110832
1477	LAP2B_MOUSE	Tmpo	Lamina-associated polypeptide 2, isoforms beta/delta/epsilon/gamma	0,121241	0,197432
1478	LAP2A_MOUSE	Tmpo	Lamina-associated polypeptide 2, isoforms alpha/zeta	0,304722	0,419582
1479	SYHC_MOUSE	Hars	Histidine--tRNA ligase, cytoplasmic	0,520139	0,240575
1480	TSC2_MOUSE	Tsc2	Tuberin	1,043	0,55142
1481	EI24_MOUSE	Ei24	Etoposide-induced protein 2.4	0,248133	-0,232632
1482	PPM1G_MOUSE	Ppm1g	Protein phosphatase 1G	1,22755	0,463165
1483	CDC37_MOUSE	Cdc37	Hsp90 co-chaperone Cdc37	1,16577	0,211465
1484	ABCB7_MOUSE	Abcb7	ATP-binding cassette sub-family B member 7, mitochondrial	0,43279	-0,311999
1485	NAB2_MOUSE	Nab2	NGFI-A-binding protein 2	0,252045	-0,386379
1486	CFAI_MOUSE	Cfi	Complement factor I	0,76018	0,636826
1487	GSTT2_MOUSE	Gstt2	Glutathione S-transferase theta-2	0,0786158	0,120135
1488	PRP4B_MOUSE	Prpf4b	Serine/threonine-protein kinase PRP4 homolog	0,491631	0,287672
1489	CERU_MOUSE	Cp	Ceruloplasmin	0,941695	0,358766
1490	CTCF_MOUSE	Ctcf	Transcriptional repressor CTCF	0,609334	0,221843
1491	MARE1_MOUSE	Mapre1	Microtubule-associated protein RP/EB family member 1	0,130657	-0,0554195
1492	PRDX2_MOUSE	Prdx2	Peroxiredoxin-2	0,951169	0,262162
1493	ARGI1_MOUSE	Arg1	Arginase-1	0,699642	0,384647
1494	TS101_MOUSE	Tsg101	Tumor susceptibility gene 101 protein	0,0991319	-0,124144
1495	HCFC1_MOUSE	Hcfc1	Host cell factor 1	1,88924	0,355487
1496	PA1B3_MOUSE	Pafah1b3	Platelet-activating factor acetylhydrolase IB subunit gamma	0,651926	0,368375
1497	PA1B2_MOUSE	Pafah1b2	Platelet-activating factor acetylhydrolase IB subunit beta	0,0413069	0,0329327
1498	SAP_MOUSE	Psap	Prosaposin	0,661982	0,389727
1499	ARHG1_MOUSE	Arhgef1	Rho guanine nucleotide exchange factor 1	1,77144	0,555663
1500	EIF2D_MOUSE	Eif2d	Eukaryotic translation initiation factor 2D	0,624068	0,175212
1501	MRE11_MOUSE	Mre11	Double-strand break repair protein MRE11	0,463602	0,537407
1502	PLSL_MOUSE	Lcp1	Plastin-2	0,0126942	-0,00693893
1503	SNTA1_MOUSE	Snta1	Alpha-1-syntrophin	0,880151	0,485438

1504	SNTB2_MOUSE	Sntb2	Beta-2-syntrophin	0,491761	0,252185
1505	FNTA_MOUSE	Fnta	Protein farnesyltransferase/geranylgeranyltransferase type-1	0,51057	-0,366569
1506	A2AP_MOUSE	Serpinf2	Alpha-2-antiplasmin	1,35039	-1,14208
1507	IGBP1_MOUSE	Igbp1	Immunoglobulin-binding protein 1	0,793712	0,185065
1508	APOC4_MOUSE	Apoc4	Apolipoprotein C-IV	1,11702	0,547595
1509	ABCD2_MOUSE	Abcd2	ATP-binding cassette sub-family D member 2	1,30012	0,609438
1510	LAMB2_MOUSE	Lamb2	Laminin subunit beta-2	0,231887	0,143796
1511	HSP74_MOUSE	Hspa4	Heat shock 70 kDa protein 4	0,620149	0,196326
1512	BAP31_MOUSE	Bcap31	B-cell receptor-associated protein 31	0,178238	-0,13297
1513	BAD_MOUSE	Bad	Bcl2-associated agonist of cell death	0,670842	0,529192
1514	COX7R_MOUSE	Cox7a2l	Cytochrome c oxidase subunit 7A-related protein, mitochondrial	0,440336	-0,196758
1515	PDE3B_MOUSE	Pde3b	cGMP-inhibited 3',5'-cyclic phosphodiesterase B	0,87784	0,32581
1516	RASH_MOUSE	Hras	GTPase HRas	0,405025	0,121445
1517	CMAH_MOUSE	Cmah	Cytidine monophosphate-N-acetylneuraminic acid hydroxylase	0,115088	-0,119319
1518	HCDH_MOUSE	Hadh	Hydroxyacyl-coenzyme A dehydrogenase, mitochondrial	0,213323	0,211703
1519	ZN638_MOUSE	Znf638	Zinc finger protein 638	1,50736	0,553067
1520	CD166_MOUSE	Alcam	CD166 antigen	0,402868	-0,236855
1521	5NTD_MOUSE	Nt5e	5'-nucleotidase	1,18962	0,553146
1522	ECM1_MOUSE	Ecm1	Extracellular matrix protein 1	1,1766	0,602824
1523	TRI25_MOUSE	Trim25	E3 ubiquitin/ISG15 ligase TRIM25	0,270316	-0,089991
1524	GSLG1_MOUSE	Glg1	Golgi apparatus protein 1	1,40962	0,760297
1525	RAD21_MOUSE	Rad21	Double-strand-break repair protein rad21 homolog	0,209487	0,12859
1526	FSCN1_MOUSE	Fscn1	Fascin	0,0317622	-0,0291595
1527	FBN1_MOUSE	Fbn1	Fibrillin-1	0,0214338	-0,0441181
1528	FCGRN_MOUSE	Fcgrt	IgG receptor FcRn large subunit p51	0,143301	0,0907219
1529	FKB10_MOUSE	Fkbp10	Peptidyl-prolyl cis-trans isomerase FKBP10	0,228989	0,129622
1530	ADRO_MOUSE	Fdxr	NADPH:adrenodoxin oxidoreductase, mitochondrial	0,444701	0,160235
1531	FXR1_MOUSE	Fxr1	Fragile X mental retardation syndrome-related protein 1	0,377011	0,186717
1532	GPAT1_MOUSE	Gpam	Glycerol-3-phosphate acyltransferase 1, mitochondrial	0,113131	0,118523
1533	KTN1_MOUSE	Ktn1	Kinectin	0,361959	-0,432707
1534	GDIB_MOUSE	Gdi2	Rab GDP dissociation inhibitor beta	0,531218	0,336394
1535	GDIR2_MOUSE	Arhgdib	Rho GDP-dissociation inhibitor 2	0,662645	0,316114
1536	HPT_MOUSE	Hp	Haptoglobin	0,343065	0,226225
1537	DD19A_MOUSE	Ddx19a	ATP-dependent RNA helicase DDX19A	0,689468	0,358253
1538	DDX5_MOUSE	Ddx5	Probable ATP-dependent RNA helicase DDX5	0,622046	0,273423
1539	ATRX_MOUSE	Atrx	Transcriptional regulator ATRX	0,472348	0,215774
1540	HS105_MOUSE	Hsph1	Heat shock protein 105 kDa	1,6167	0,766339
1541	ITIH1_MOUSE	Itih1	Inter-alpha-trypsin inhibitor heavy chain H1	1,41348	0,421021
1542	ITIH2_MOUSE	Itih2	Inter-alpha-trypsin inhibitor heavy chain H2	0,968087	0,778904
1543	ITIH3_MOUSE	Itih3	Inter-alpha-trypsin inhibitor heavy chain H3	0,802269	0,393192
1544	DNJC1_MOUSE	Dnajc1	DnaJ homolog subfamily C member 1	0,132761	-0,0959866
1545	IL1AP_MOUSE	Il1rap	Interleukin-1 receptor accessory protein	0,39742	0,449246
1546	RT31_MOUSE	Mrps31	28S ribosomal protein S31, mitochondrial	0,309924	0,208527
1547	ITA6_MOUSE	Itga6	Integrin alpha-6	1,0384	0,89238
1548	EI2BD_MOUSE	Eif2b4	Translation initiation factor eIF-2B subunit delta	0,259564	0,201348
1549	SERA_MOUSE	Phgdh	D-3-phosphoglycerate dehydrogenase	1,55089	1,33879
1550	KINH_MOUSE	Kif5b	Kinesin-1 heavy chain	0,62986	0,277066
1551	LASP1_MOUSE	Lasp1	LIM and SH3 domain protein 1	1,11186	0,353893
1552	PDCD4_MOUSE	Pdcd4	Programmed cell death protein 4	0,108256	-0,0591465
1553	MRC1_MOUSE	Mrc1	Macrophage mannose receptor 1	0,774827	-0,441852
1554	PZP_MOUSE	Pzp	Pregnancy zone protein	1,07939	0,442014
1555	MYH10_MOUSE	Myh10	Myosin-10	0,173171	-0,293366

1556	MCM7_MOUSE	Mcm7	DNA replication licensing factor MCM7	0,76565	0,606584
1557	NPM_MOUSE	Npm1	Nucleophosmin	1,13067	0,606306
1558	NNTM_MOUSE	Nnt	NAD(P) transhydrogenase, mitochondrial	0,507886	0,212551
1559	PCBP2_MOUSE	Pcbp2	Poly(rC)-binding protein 2	0,12891	-0,0763092
1560	POSTN_MOUSE	Postn	Periostin	0,6697	1,40073
1561	CTR9_MOUSE	Ctr9	RNA polymerase-associated protein CTR9 homolog	0,138999	0,0862995
1562	CP131_MOUSE	Cep131	Centrosomal protein of 131 kDa	0,0907783	0,175159
1563	PEA15_MOUSE	Pea15	Astrocytic phosphoprotein PEA-15	0,143735	-0,148088
1564	PLCG1_MOUSE	Plcg1	1-phosphatidylinositol 4,5-bisphosphate phosphodiesterase gamma-1	0,577646	-0,46412
1565	PP14B_MOUSE	Ppp1r14b	Protein phosphatase 1 regulatory subunit 14B	0,0915605	0,136216
1566	PON3_MOUSE	Pon3	Serum paraoxonase/lactonase 3	0,101657	-0,124463
1567	SRSF2_MOUSE	Srsf2	Serine/arginine-rich splicing factor 2	0,12778	0,10838
1568	DDX3Y_MOUSE	Ddx3y	ATP-dependent RNA helicase DDX3Y	0,333155	-0,13054
1569	DDX3X_MOUSE	Ddx3x	ATP-dependent RNA helicase DDX3X	1,71063	0,446273
1570	SSRD_MOUSE	Ssr4	Translocon-associated protein subunit delta	0,568649	0,278002
1571	DPYL3_MOUSE	Dpysl3	Dihydropyrimidinase-related protein 3	0,027365	0,0265049
1572	SNRPA_MOUSE	Snrpa	U1 small nuclear ribonucleoprotein A	0,22821	-0,191053
1573	RFA2_MOUSE	Rpa2	Replication protein A 32 kDa subunit	0,519294	0,175042
1574	SF3A2_MOUSE	Sf3a2	Splicing factor 3A subunit 2	0,67509	0,287067
1575	TGFI1_MOUSE	Tgfb1i1	Transforming growth factor beta-1-induced transcript 1 protein	0,0696416	0,0569305
1576	SN_MOUSE	Siglec1	Sialoadhesin	1,34883	0,789771
1577	SPTB2_MOUSE	Sptbn1	Spectrin beta chain, non-erythrocytic 1	0,135105	0,137697
1578	THRSP_MOUSE	Thrsp	Thyroid hormone-inducible hepatic protein	0,787715	-1,9427
1579	MED22_MOUSE	Med22	Mediator of RNA polymerase II transcription subunit 22	0,398681	0,484307
1580	TIF1B_MOUSE	Trim28	Transcription intermediary factor 1-beta	1,15988	0,351453
1581	TSN_MOUSE	Tsn	Translin	0,170491	-0,207894
1582	TFR1_MOUSE	Tfrc	Transferrin receptor protein 1	0,732957	-0,653273
1583	RU17_MOUSE	Snrnp70	U1 small nuclear ribonucleoprotein 70 kDa	1,12234	0,426707
1584	SPT6H_MOUSE	Supt6h	Transcription elongation factor SPT6	0,73061	0,422361
1585	ZPR1_MOUSE	Zpr1	Zinc finger protein ZPR1	0,443015	0,243925
1586	PHLA1_MOUSE	Phlda1	Pleckstrin homology-like domain family A member 1	0,439669	0,406136
1587	TPD52_MOUSE	Tpd52	Tumor protein D52	0,0790257	0,0635826
1588	SRBS1_MOUSE	Sorbs1	Sorbin and SH3 domain-containing protein 1	0,678701	0,424861
1589	DBNL_MOUSE	Dbnl	Drebrin-like protein	1,26653	0,329153
1590	SH3G1_MOUSE	Sh3gl1	Endophilin-A2	0,232269	0,110841
1591	NDUA4_MOUSE	Ndufa4	Cytochrome c oxidase subunit NDUFA4	0,734544	-0,294509
1592	CYTB_MOUSE	Cstb	Cystatin-B	0,811832	0,248965
1593	SMAD2_MOUSE	Smad2	Mothers against decapentaplegic homolog 2	0,3551	0,156813
1594	FKBP3_MOUSE	Fkbp3	Peptidyl-prolyl cis-trans isomerase FKBP3	0,0965489	0,0616245
1595	IF4G2_MOUSE	Eif4g2	Eukaryotic translation initiation factor 4 gamma 2	0,203142	0,155867
1596	VAT1_MOUSE	Vat1	Synaptic vesicle membrane protein VAT-1 homolog	0,544191	-0,318651
1597	MINT_MOUSE	Spen	Msx2-interacting protein	0,160456	-0,169386
1598	ZYX_MOUSE	Zyx	Zyxin	0,263165	0,255012
1599	TP4A1_MOUSE	Ptp4a1	Protein tyrosine phosphatase type IVA 1	0,340316	-0,295094
1600	COMD3_MOUSE	Commd3	COMM domain-containing protein 3	0,966103	0,422615
1601	SBP2_MOUSE	Selenbp2	Selenium-binding protein 2	0,483493	-0,202768
1602	MK03_MOUSE	Mapk3	Mitogen-activated protein kinase 3	0,11183	-0,0682838
1603	NUP62_MOUSE	Nup62	Nuclear pore glycoprotein p62	0,261607	-0,446533
1604	EST3A_MOUSE	Ces3a	Carboxylesterase 3A	1,25379	0,883447
1605	UD11_MOUSE	Ugt1a1	UDP-glucuronosyltransferase 1-1	0,003443	0,00261993
1606	CAVN2_MOUSE	Cavin2	Caveolae-associated protein 2	1,25362	0,303484
1607	MP2K2_MOUSE	Map2k2	Dual specificity mitogen-activated protein kinase kinase 2	1,2862	0,470441

1608	EGLN_MOUSE	Eng	Endoglin	0,459125	0,803655
1609	CRK_MOUSE	Crk	Adapter molecule crk	0,537986	0,221581
1610	RALY_MOUSE	Raly	RNA-binding protein Raly	0,952303	0,479921
1611	SPRE_MOUSE	Spr	Sepiapterin reductase	0,173334	0,155256
1612	AOFA_MOUSE	Maoa	Amine oxidase [flavin-containing] A	0,932642	-0,404788
1613	BTF3_MOUSE	Btf3	Transcription factor BTF3	0,835087	0,335331
1614	EST1E_MOUSE	Ces1e	Carboxylesterase 1E	1,44419	1,49167
1615	ASPG_MOUSE	Aga	N(4)-(beta-N-acetylglucosaminyl)-L-asparaginase	1,06687	0,442719
1616	SF01_MOUSE	Sf1	Splicing factor 1	0,5183	0,338736
1617	CDK6_MOUSE	Cdk6	Cyclin-dependent kinase 6	0,0399644	0,0171871
1618	IFIT1_MOUSE	Ifit1	Interferon-induced protein with tetratricopeptide repeats 1	0,701017	0,428621
1619	STXB2_MOUSE	Stxbp2	Syntaxin-binding protein 2	0,672276	0,43569
1620	MYO6_MOUSE	Myo6	Unconventional myosin-VI	0,158015	0,117093
1621	SQSTM_MOUSE	Sqstm1	Sequestosome-1	2,21977	1,29635
1622	IFIT3_MOUSE	Ifit3	Interferon-induced protein with tetratricopeptide repeats 3	0,788414	-0,614974
1623	RGN_MOUSE	Rgn	Regucalcin	0,106431	-0,137727
1624	FKBP5_MOUSE	Fkbp5	Peptidyl-prolyl cis-trans isomerase FKBP5	0,825218	-0,311908
1625	CH10_MOUSE	Hspe1	10 kDa heat shock protein, mitochondrial	0,396749	0,217022
1626	DHSO_MOUSE	Sord	Sorbitol dehydrogenase	0,424199	0,178268
1627	ATP7B_MOUSE	Atp7b	Copper-transporting ATPase 2	1,19535	-0,564849
1628	CP2CT_MOUSE	Cyp2c29	Cytochrome P450 2C29	0,375539	-0,329755
1629	CP3AB_MOUSE	Cyp3a11	Cytochrome P450 3A11	0,376437	-0,464805
1630	CP4B1_MOUSE	Cyp4b1	Cytochrome P450 4B1	0,394402	-0,211481
1631	CP3AD_MOUSE	Cyp3a13	Cytochrome P450 3A13	0,340641	-0,247824
1632	GSTT1_MOUSE	Gstt1	Glutathione S-transferase theta-1	0,438661	0,281251
1633	PTPRD_MOUSE	Ptprd	Receptor-type tyrosine-protein phosphatase delta	0,0255722	0,0259819
1634	TOP2B_MOUSE	Top2b	DNA topoisomerase 2-beta	0,0906695	-0,050037
1635	TPP2_MOUSE	Tpp2	Tripeptidyl-peptidase 2	0,561425	0,267134
1636	GLPK_MOUSE	Gk	Glycerol kinase	0,674931	0,322736
1637	GPDM_MOUSE	Gpd2	Glycerol-3-phosphate dehydrogenase, mitochondrial	1,18908	0,496434
1638	H2A2A_MOUSE;H2A	Hist2h2aa1;Hist2	Histone H2A type 2-C	0,362003	-0,440004
1639	NQO1_MOUSE	Nqo1	NAD(P)H dehydrogenase [quinone] 1	1,06845	-0,715784
1640	SPEE_MOUSE	Srm	Spermidine synthase	0,483267	0,386166
1641	USF2_MOUSE	Usf2	Upstream stimulatory factor 2	0,870685	0,557278
1642	ZA2G_MOUSE	Azgp1	Zinc-alpha-2-glycoprotein	0,056003	0,0636753
1643	VINC_MOUSE	Vcl	Vinculin	0,541097	0,341022
1644	PUR2_MOUSE	Gart	Trifunctional purine biosynthetic protein adenosine-3	0,44424	0,327725
1645	RETST_MOUSE	Retsat	All-trans-retinol 13,14-reductase	1,60626	2,18195
1646	WAPL_MOUSE	Wapl	Wings apart-like protein homolog	0,262533	-0,0785839
1647	PTPM1_MOUSE	Ptpmt1	Phosphatidylglycerophosphatase and protein-tyrosine phosphatase 1	0,0343974	-0,0257702
1648	CLH1_MOUSE	Cltc	Clathrin heavy chain 1	0,66724	0,221452
1649	RIPR1_MOUSE	Ripor1	Rho family-interacting cell polarization regulator 1	1,11068	1,12389
1650	GIT1_MOUSE	Git1	ARF GTPase-activating protein GIT1	0,683241	0,381244
1651	GALK2_MOUSE	Galk2	N-acetylgalactosamine kinase	0,483099	0,479254
1652	SYMC_MOUSE	Mars	Methionine--tRNA ligase, cytoplasmic	0,709082	0,282841
1653	SAM9L_MOUSE	Samd9l	Sterile alpha motif domain-containing protein 9-like	0,311643	-0,221233
1654	PNKD_MOUSE	Pnkd	Probable hydrolase PNKD	2,27036	0,670633
1655	HECD1_MOUSE	Hectd1	E3 ubiquitin-protein ligase HECTD1	1,49378	0,380782
1656	HBS1L_MOUSE	Hbs1l	HBS1-like protein	0,258882	0,12263
1657	TMCC1_MOUSE	Tmcc1	Transmembrane and coiled-coil domains protein 1	1,74551	0,585851
1658	LTN1_MOUSE	Ltn1	E3 ubiquitin-protein ligase listerin	1,17173	0,522173
1659	PDS5A_MOUSE	Pds5a	Sister chromatid cohesion protein PDS5 homolog A	0,901802	0,207599

1660	SWP70_MOUSE	Swap70	Switch-associated protein 70	0,181639	0,119037
1661	CDC5L_MOUSE	Cdc5l	Cell division cycle 5-like protein	0,611701	0,473983
1662	LAR4B_MOUSE	Larp4b	La-related protein 4B	0,781151	0,40789
1663	F120A_MOUSE	FAM120A	Constitutive coactivator of PPAR-gamma-like protein 1	0,369964	0,200587
1664	UBP7_MOUSE	Usp7	Ubiquitin carboxyl-terminal hydrolase 7	0,367004	0,107891
1665	CGNL1_MOUSE	Cgnl1	Cingulin-like protein 1	0,21697	-0,0954044
1666	RHG21_MOUSE	Arhgap21	Rho GTPase-activating protein 21	1,1356	1,33161
1667	NOP58_MOUSE	Nop58	Nucleolar protein 58	0,469966	0,231935
1668	SMCA2_MOUSE	Smarca2	Probable global transcription activator SNF2L2	0,133569	-0,179516
1669	SCMC3_MOUSE	Slc25a23	Calcium-binding mitochondrial carrier protein SCaMC-3	0,264516	0,159913
1670	NOMO1_MOUSE	Nomo1	Nodal modulator 1	0,297962	0,248168
1671	ZN219_MOUSE	Znf219	Zinc finger protein 219	0,993919	0,564916
1672	TPM4_MOUSE	Tpm4	Tropomyosin alpha-4 chain	0,0295541	0,020821
1673	CLCB_MOUSE	Cltb	Clathrin light chain B	0,00758774	-0,00186081
1674	EXOC3_MOUSE	Exoc3	Exocyst complex component 3	0,0745853	-0,0774345
1675	RRP5_MOUSE	Pdcd11	Protein RRP5 homolog	0,148636	0,131934
1676	PEPL1_MOUSE	Npepl1	Probable aminopeptidase NPEPL1	1,89506	0,497499
1677	DUS23_MOUSE	Dusp23	Dual specificity protein phosphatase 23	0,939424	0,259954
1678	SR140_MOUSE	U2surp	U2 snRNP-associated SURP motif-containing protein	0,292251	-0,218036
1679	WDR44_MOUSE	Wdr44	WD repeat-containing protein 44	0,525665	0,196257
1680	HELB_MOUSE	Helb	DNA helicase B	0,860935	0,740532
1681	CPSF6_MOUSE	Cpsf6	Cleavage and polyadenylation specificity factor subunit 6	0,0264339	-0,0160267
1682	FND3B_MOUSE	Fndc3b	Fibronectin type III domain-containing protein 3B	0,880136	0,196147
1683	DNJC8_MOUSE	Dnajc8	DnaJ homolog subfamily C member 8	0,424975	-0,396875
1684	S23IP_MOUSE	Sec23ip	SEC23-interacting protein	0,0803224	-0,0728973
1685	ZC11A_MOUSE	Zc3h11a	Zinc finger CCCH domain-containing protein 11A	1,15109	0,278302
1686	IF4G1_MOUSE	Eif4g1	Eukaryotic translation initiation factor 4 gamma 1	0,0661921	0,0509289
1687	RBM26_MOUSE	Rbm26	RNA-binding protein 26	0,125119	0,075008
1688	ZN740_MOUSE	Znf740	Zinc finger protein 740	0,47605	0,576265
1689	SORCN_MOUSE	Sri	Sorcin	0,24585	0,09599
1690	XPP1_MOUSE	Xpnpep1	Xaa-Pro aminopeptidase 1	0,213257	0,0671516
1691	2ABA_MOUSE	Ppp2r2a	Serine/threonine-protein phosphatase 2A	0,326885	0,162527
1692	P4R3A_MOUSE	Ppp4r3a	Serine/threonine-protein phosphatase 4 regulatory subunit 3A	0,937719	0,661671
1693	ODBB_MOUSE	Bckdhb	2-oxoisovalerate dehydrogenase subunit beta, mitochondrial	0,144513	-0,110746
1694	NUD16_MOUSE	Nudt16	U8 snoRNA-decapping enzyme	0,220626	-0,123271
1695	SIK3_MOUSE	Sik3	Serine/threonine-protein kinase SIK3	0,359499	0,142397
1696	U520_MOUSE	Snrnp200	U5 small nuclear ribonucleoprotein 200 kDa helicase	0,95922	0,22333
1697	ABCF1_MOUSE	Abcf1	ATP-binding cassette sub-family F member 1	0,229177	-0,193494
1698	SMHD1_MOUSE	Smchd1	Structural maintenance of chromosomes flexible protein 1	0,328571	-0,189314
1699	UGGG1_MOUSE	Uggt1	UDP-glucose:glycoprotein glucosyltransferase 1	0,104939	0,0969715
1700	XPO1_MOUSE	Xpo1	Exportin-1	0,422248	-0,267768
1701	NEST_MOUSE	Nes	Nestin	0,877143	0,403304
1702	TXD15_MOUSE	Txndc15	Thioredoxin domain-containing protein 15	2,43684	0,473545
1703	PX11C_MOUSE	Pex11g	Peroxisomal membrane protein 11C	0,132914	0,15708
1704	SPCS_MOUSE	Sepsecs	O-phosphoseryl-tRNA(Sec) selenium transferase	0,58454	0,450788
1705	PCNP_MOUSE	Pcnp	PEST proteolytic signal-containing nuclear protein	0,71215	0,497595
1706	SAT2_MOUSE	Sat2	Diamine acetyltransferase 2	0,0986889	0,081411
1707	TATD1_MOUSE	Tatdn1	Putative deoxyribonuclease TATDN1	0,231997	-0,132577
1708	SNX6_MOUSE	Snx6	Sorting nexin-6	1,18691	-0,291977
1709	FHOD1_MOUSE	Fhod1	FH1/FH2 domain-containing protein 1	1,13324	0,438796
1710	FKB15_MOUSE	Fkbp15	FK506-binding protein 15	0,6714	-0,456097
1711	DDX51_MOUSE	Ddx51	ATP-dependent RNA helicase DDX51	0,2626	0,360073

1712	OXSR1_MOUSE	Oxsr1	Serine/threonine-protein kinase OSR1	1,73716	0,724573
1713	ATLA2_MOUSE	Atl2	Atlastin-2	0,139242	0,0850964
1714	DCA13_MOUSE	Dcaf13	DDB1- and CUL4-associated factor 13	0,112101	-0,140247
1715	BCR_MOUSE	Bcr	Breakpoint cluster region protein	1,33308	1,22658
1716	TXLNA_MOUSE	Txlna	Alpha-taxilin	1,62614	0,344396
1717	GAPD1_MOUSE	Gapvd1	GTPase-activating protein and VPS9 domain-containing protein 1	1,13522	0,478305
1718	HERC4_MOUSE	Herc4	Probable E3 ubiquitin-protein ligase HERC4	0,100819	0,0506569
1719	PTN23_MOUSE	Ptpn23	Tyrosine-protein phosphatase non-receptor type 23	1,16442	0,461856
1720	LPPRC_MOUSE	Lrpprc	Leucine-rich PPR motif-containing protein, mitochondrial	0,0689269	-0,0449421
1721	GALT2_MOUSE	Galnt2	Polypeptide N-acetylgalactosaminyltransferase 2	0,509946	0,292477
1722	2A5A_MOUSE	Ppp2r5a	Serine/threonine-protein phosphatase 2A	0,841169	0,234318
1723	SMRC2_MOUSE	Smarcc2	SWI/SNF complex subunit SMARCC2	0,0253071	0,0175732
1724	ECM29_MOUSE	Ecpas	Proteasome adapter and scaffold protein ECM29	0,393814	0,24871
1725	DC1L2_MOUSE	Dync1li2	Cytoplasmic dynein 1 light intermediate chain 2	0,00898447	-0,00600471
1726	SRSF1_MOUSE	Srsf1	Serine/arginine-rich splicing factor 1	0,346197	0,258918
1727	MYLK_MOUSE	Mylk	Myosin light chain kinase, smooth muscle	0,248996	0,124164
1728	CHD4_MOUSE	Chd4	Chromodomain-helicase-DNA-binding protein 4	0,755764	0,474402
1729	AEDO_MOUSE	Ado	2-aminoethanethiol dioxygenase	0,772456	0,223992
1730	SNR40_MOUSE	Snrnp40	U5 small nuclear ribonucleoprotein 40 kDa protein	0,712211	-0,628814
1731	ABHDA_MOUSE	Abhd10	Mycophenolic acid acyl-glucuronide esterase, mitochondrial	0,564743	-0,359793
1732	CTIF_MOUSE	Ctif	CBP80/20-dependent translation initiation factor	0,350112	0,466383
1733	MARE3_MOUSE	Mapre3	Microtubule-associated protein RP/EB family member 3	0,301674	0,160926
1734	NUP98_MOUSE	Nup98	Nuclear pore complex protein Nup98-Nup96	0,164478	0,0969223
1735	NAA50_MOUSE	Naa50	N-alpha-acetyltransferase 50	0,351294	-0,241535
1736	DHX29_MOUSE	Dhx29	ATP-dependent RNA helicase DHX29	0,19876	0,101499
1737	EXOC8_MOUSE	Exoc8	Exocyst complex component 8	0,601123	0,354694
1738	GNL3L_MOUSE	Gnl3l	Guanine nucleotide-binding protein-like 3-like protein	2,12023	1,37671
1739	BUD31_MOUSE	Bud31	Protein BUD31 homolog	1,17149	0,673394
1740	JUPI2_MOUSE	Jpt2	Jupiter microtubule associated homolog 2	0,0616579	0,0835876
1741	NAA35_MOUSE	Naa35	N-alpha-acetyltransferase 35, NatC auxiliary subunit	0,479701	-0,652466
1742	KCC2D_MOUSE	Camk2d	Calcium/calmodulin-dependent protein kinase type II subunit delta	1,55235	1,07557
1743	NUDC1_MOUSE	Nudcd1	NudC domain-containing protein 1	1,00437	0,481173
1744	AT2B4_MOUSE	Atp2b4	Plasma membrane calcium-transporting ATPase 4	0,693705	0,284989
1745	DDX58_MOUSE	Ddx58	Probable ATP-dependent RNA helicase DDX58	0,231354	0,166999
1746	SEC20_MOUSE	Bnip1	Vesicle transport protein SEC20	0,734686	0,283136
1747	PAQR9_MOUSE	Paqr9	Membrane progesterone receptor epsilon	1,64772	1,65403
1748	MYH14_MOUSE	Myh14	Myosin-14	0,0663547	-0,0486988
1749	RBP10_MOUSE	Ranbp10	Ran-binding protein 10	1,31783	1,0146
1750	WDFY3_MOUSE	Wdfy3	WD repeat and FYVE domain-containing protein 3	0,356371	0,394968
1751	NOSTN_MOUSE	Nostrin	Nostrin	0,0295622	-0,0187565
1752	UBR2_MOUSE	Ubr2	E3 ubiquitin-protein ligase UBR2	0,104729	0,0746738
1753	KCD12_MOUSE	Kctd12	BTB/POZ domain-containing protein KCTD12	0,877341	0,438725
1754	GGYF2_MOUSE	Gigyf2	GRB10-interacting GYF protein 2	0,93353	0,726856
1755	MTMR5_MOUSE	Sbf1	Myotubularin-related protein 5	0,862112	0,461792
1756	TEX2_MOUSE	Tex2	Testis-expressed protein 2	0,83182	0,456517
1757	UBE2O_MOUSE	Ube2o	(E3-independent) E2 ubiquitin-conjugating enzyme UBE2O	0,614298	0,354352
1758	ZC3H4_MOUSE	Zc3h4	Zinc finger CCCH domain-containing protein 4	0,448487	0,31247
1759	CNOT1_MOUSE	Cnot1	CCR4-NOT transcription complex subunit 1	1,47615	0,370184
1760	CAND1_MOUSE	Cand1	Cullin-associated NEDD8-dissociated protein 1	0,208763	-0,176123
1761	LARP1_MOUSE	Larp1	La-related protein 1	2,35382	0,471046
1762	KDM1A_MOUSE	Kdm1a	Lysine-specific histone demethylase 1A	0,212221	0,14636
1763	VIP2_MOUSE	Ppip5k2	Inositol hexakisphosphate and diphosphoinositol-pentakisphosphate kinase :	0,360269	-0,268956

1764	MLEC_MOUSE	Mlec	Malectin	0,924096	0,552832
1765	ACAP2_MOUSE	Acap2	Arf-GAP with coiled-coil, ANK repeat and PH domain-containing protein 2	0,51217	0,207696
1766	WDR43_MOUSE	Wdr43	WD repeat-containing protein 43	2,04961	0,333632
1767	LSM8_MOUSE	Lsm8	U6 snRNA-associated Sm-like protein LSm8	0,836297	0,33039
1768	RS9_MOUSE	Rps9	40S ribosomal protein S9	0,0594305	-0,0233971
1769	SYNE2_MOUSE	Syne2	Nesprin-2	0,487542	0,273841
1770	SPCS3_MOUSE	Spcs3	Signal peptidase complex subunit 3	0,895276	-0,507188
1771	SYNE1_MOUSE	Syne1	Nesprin-1	0,115485	-0,321661
1772	RS27_MOUSE	Rps27	40S ribosomal protein S27	1,62391	0,49806
1773	RL10_MOUSE	Rpl10	60S ribosomal protein L10	0,546216	0,465676
1774	RL35_MOUSE	Rpl35	60S ribosomal protein L35	1,25234	0,443335
1775	IF2A_MOUSE	Eif2s1	Eukaryotic translation initiation factor 2 subunit 1	0,0719045	0,05308
1776	UB2R2_MOUSE	Ube2r2	Ubiquitin-conjugating enzyme E2 R2	1,08234	0,306757
1777	AKAP9_MOUSE	Akap9	A-kinase anchor protein 9	0,100903	0,0513496
1778	NADE_MOUSE	Nadsyn1	Glutamine-dependent NAD(+) synthetase	0,341615	0,190952
1779	TLN2_MOUSE	Tln2	Talin-2	0,0770345	0,0858463
1780	KAT3_MOUSE	Kyat3	Kynurenine--oxoglutarate transaminase 3	0,807168	-0,279788
1781	GIMA8_MOUSE	Gimap8	GTPase IMAP family member 8	1,26548	0,974254
1782	MINY1_MOUSE	Mindy1	Ubiquitin carboxyl-terminal hydrolase MINDY-1	0,851694	0,588807
1783	S22AR_MOUSE	Slc22a27	Solute carrier family 22 member 27	0,00382359	-0,00627899
1784	2AAA_MOUSE	Ppp2r1a	Serine/threonine-protein phosphatase 2A 65 kDa regulatory subunit A alpha	0,84841	0,31913
1785	MB12A_MOUSE	Mvb12a	Multivesicular body subunit 12A	1,12765	0,557366
1786	MIC27_MOUSE	Apool	MICOS complex subunit Mic27	0,438434	-0,278577
1787	S39A4_MOUSE	Slc39a4	Zinc transporter ZIP4	1,38974	1,05446
1788	MSRB2_MOUSE	Msrb2	Methionine-R-sulfoxide reductase B2, mitochondrial	0,123671	0,0601013
1789	FBX22_MOUSE	Fbxo22	F-box only protein 22	0,0069264	0,00688858
1790	3HAO_MOUSE	Haao	3-hydroxyanthranilate 3,4-dioxygenase	0,455259	-0,146377
1791	CCD25_MOUSE	Ccdc25	Coiled-coil domain-containing protein 25	1,15906	-0,897297
1792	SND1_MOUSE	Snd1	Staphylococcal nuclease domain-containing protein 1	0,699249	0,382482
1793	NP1L4_MOUSE	Nap1l4	Nucleosome assembly protein 1-like 4	1,22817	0,443001
1794	MTCH2_MOUSE	Mtch2	Mitochondrial carrier homolog 2	0,0219119	0,017276
1795	PICAL_MOUSE	Picalm	Phosphatidylinositol-binding clathrin assembly protein	0,389231	-0,428638
1796	CYFP1_MOUSE	Cyfip1	Cytoplasmic FMR1-interacting protein 1	0,569291	0,118738
1797	FCSK_MOUSE	Fcsk	L-fucose kinase	0,624503	0,330167
1798	NDUAC_MOUSE	Ndufa12	NADH dehydrogenase [ubiquinone] 1 alpha subcomplex subunit 12	0,214638	0,331468
1799	HNRPQ_MOUSE	Syncrip	Heterogeneous nuclear ribonucleoprotein Q	0,284111	0,12373
1800	TBB2A_MOUSE	Tubb2a	Tubulin beta-2A chain	0,775595	0,516737
1801	WDR91_MOUSE	Wdr91	WD repeat-containing protein 91	0,243775	0,237056
1802	PCP_MOUSE	Prcp	Lysosomal Pro-X carboxypeptidase	0,159138	-0,130236
1803	ABCG2_MOUSE	Abcg2	ATP-binding cassette sub-family G member 2	0,323693	-0,250526
1804	CIAO3_MOUSE	Ciao3	Cytosolic iron-sulfur assembly component 3	1,79241	0,842078
1805	THOC7_MOUSE	Thoc7	THO complex subunit 7 homolog	1,06088	-0,557104
1806	HUWE1_MOUSE	Huwe1	E3 ubiquitin-protein ligase HUWE1	0,156803	0,0963535
1807	SMAP2_MOUSE	Smap2	Stromal membrane-associated protein 2	0,65766	0,38489
1808	LC7L2_MOUSE	Luc7l2	Putative RNA-binding protein Luc7-like 2	1,85062	0,371852
1809	RPF1_MOUSE	Rpf1	Ribosome production factor 1	0,942436	0,921781
1810	SUCHY_MOUSE	Sugct	Succinate--hydroxymethylglutarate CoA-transferase	0,132619	-0,109908
1811	LDHD_MOUSE	Ldhd	Probable D-lactate dehydrogenase, mitochondrial	0,370831	0,242169
1812	2AAB_MOUSE	Ppp2r1b	Serine/threonine-protein phosphatase 2A 65 kDa regulatory subunit A beta i:	1,178	0,950091
1813	DEK_MOUSE	Dek	Protein DEK	0,336524	-0,218715
1814	INT3_MOUSE	Ints3	Integrator complex subunit 3	0,111054	0,0841228
1815	ROBO2_MOUSE	Robo2	Roundabout homolog 2	0,905521	0,617259

1816	S7A6O_MOUSE	Slc7a6os	Probable RNA polymerase II nuclear localization protein SLC7A6OS	1,56001	1,43971
1817	MTMRA_MOUSE	Mtmr10	Myotubularin-related protein 10	1,53691	0,605836
1818	ACTN1_MOUSE	Actn1	Alpha-actinin-1	0,00474798	-0,00189476
1819	MBB1A_MOUSE	Mybbp1a	Myb-binding protein 1A	0,647249	0,25304
1820	TPPP_MOUSE	Tppp	Tubulin polymerization-promoting protein	2,20658	0,744778
1821	PKHA6_MOUSE	Plekha6	Pleckstrin homology domain-containing family A member 6	0,662643	0,220217
1822	ATX2L_MOUSE	Atxn2l	Ataxin-2-like protein	0,294086	0,123689
1823	OTUB1_MOUSE	Otub1	Ubiquitin thioesterase OTUB1	0,635923	-0,377173
1824	INT7_MOUSE	Ints7	Integrator complex subunit 7	1,18489	0,47408
1825	CCD93_MOUSE	Ccdc93	Coiled-coil domain-containing protein 93	0,335476	-0,19377
1826	PRC2A_MOUSE	Prrc2a	Protein PRRC2A	0,909576	0,379085
1827	CTDP1_MOUSE	Ctdp1	RNA polymerase II subunit A C-terminal domain phosphatase	0,661944	0,495677
1828	SCAF4_MOUSE	Scaf4	SR-related and CTD-associated factor 4	0,283468	0,164795
1829	PP6R1_MOUSE	Ppp6r1	Serine/threonine-protein phosphatase 6 regulatory subunit 1	0,0102007	0,0135807
1830	ELP1_MOUSE	Elp1	Elongator complex protein 1	0,160926	0,0872398
1831	MRCKB_MOUSE	Cdc42bpb	Serine/threonine-protein kinase MRCK beta	0,591013	0,267612
1832	SAHH2_MOUSE	Ahcyl1	S-adenosylhomocysteine hydrolase-like protein 1	0,262098	-0,156942
1833	MIB1_MOUSE	Mib1	E3 ubiquitin-protein ligase MIB1	0,671179	0,891556
1834	GBG5_MOUSE	Gng5	Guanine nucleotide-binding protein G(I)/G(S)/G(O) subunit gamma-5	0,712406	0,207954
1835	RPAP1_MOUSE	Rpap1	RNA polymerase II-associated protein 1	0,932035	0,839187
1836	ERBIN_MOUSE	Erbin	Erbin	0,537952	0,32849
1837	MON2_MOUSE	Mon2	Protein MON2 homolog	0,300747	-0,280301
1838	NISCH_MOUSE	Nisch	Nischarin	2,46483	0,456982
1839	UBR5_MOUSE	Ubr5	E3 ubiquitin-protein ligase UBR5	0,890909	1,41485
1840	DCAF1_MOUSE	Dcaf1	DDB1- and CUL4-associated factor 1	0,093812	0,113127
1841	C170B_MOUSE	Cep170b	Centrosomal protein of 170 kDa protein B	0,749892	0,290427
1842	PUM2_MOUSE	Pum2	Pumilio homolog 2	0,702758	0,540134
1843	MFN2_MOUSE	Mfn2	Mitofusin-2	0,362551	-0,179205
1844	SCRIB_MOUSE	Scrib	Protein scribble homolog	0,153698	-0,0840366
1845	PUM1_MOUSE	Pum1	Pumilio homolog 1	1,46034	0,355636
1846	UBP8_MOUSE	Usp8	Ubiquitin carboxyl-terminal hydrolase 8	0,0484182	0,0755959
1847	NU214_MOUSE	Nup214	Nuclear pore complex protein Nup214	1,2654	0,85339
1848	UBE3C_MOUSE	Ube3c	Ubiquitin-protein ligase E3C	0,214585	0,259184
1849	FADS6_MOUSE	Fads6	Fatty acid desaturase 6	0,932584	-1,04593
1850	SEPT9_MOUSE	Septin9	Septin-9	0,705262	0,236113
1851	RB3GP_MOUSE	Rab3gap1	Rab3 GTPase-activating protein catalytic subunit	0,0372272	0,0167908
1852	NAA15_MOUSE	Naa15	N-alpha-acetyltransferase 15, NatA auxiliary subunit	0,979818	0,545708
1853	MOGS_MOUSE	Mogs	Mannosyl-oligosaccharide glucosidase	0,384042	0,0643856
1854	DGKZ_MOUSE	Dgkz	Diacylglycerol kinase zeta	1,90743	1,12584
1855	ANKZ1_MOUSE	Ankzf1	Ankyrin repeat and zinc finger domain-containing protein 1	0,576001	0,461341
1856	PGRC2_MOUSE	Pgrmc2	Membrane-associated progesterone receptor component 2	1,04109	0,217173
1857	RPAB1_MOUSE	Polr2e	DNA-directed RNA polymerases I, II, and III subunit RPABC1	0,0386001	-0,0291653
1858	KCMF1_MOUSE	Kcmf1	E3 ubiquitin-protein ligase KCMF1	0,202192	0,199498
1859	IMPA3_MOUSE	Impad1	Inositol monophosphatase 3	0,668441	1,21773
1860	CAMP3_MOUSE	Camsap3	Calmodulin-regulated spectrin-associated protein 3	2,36243	0,676435
1861	FA98B_MOUSE	Fam98b	Protein FAM98B	0,652283	0,569437
1862	TRI56_MOUSE	Trim56	E3 ubiquitin-protein ligase TRIM56	1,22402	0,472551
1863	SRA1_MOUSE	Sra1	Steroid receptor RNA activator 1	1,8165	0,770991
1864	TDRKH_MOUSE	Tdrkh	Tudor and KH domain-containing protein	0,847089	0,731341
1865	EPN1_MOUSE	Epn1	Epsin-1	0,0358645	-0,0372055
1866	PP1RA_MOUSE	Ppp1r10	Serine/threonine-protein phosphatase 1 regulatory subunit 10	0,363146	0,139738
1867	GSTM7_MOUSE	Gstm7	Glutathione S-transferase Mu 7	1,61644	-0,628403

1868	THNS2_MOUSE	Thnsl2	Threonine synthase-like 2	0,624626	0,34105
1869	SPN1_MOUSE	Snupn	Snurportin-1	0,106063	0,0458458
1870	FACE1_MOUSE	Zmpste24	CAAX prenyl protease 1 homolog	0,611472	-0,412611
1871	AGFG2_MOUSE	Agfg2	Arf-GAP domain and FG repeat-containing protein 2	0,0848762	0,0688534
1872	LYRIC_MOUSE	Mtdh	Protein LYRIC	1,4451	0,256958
1873	VAC14_MOUSE	Vac14	Protein VAC14 homolog	0,236774	0,331014
1874	CA174_MOUSE		UPF0688 protein C1orf174 homolog	1,04211	0,35594
1875	DDRGK_MOUSE	Ddrgk1	DDRGK domain-containing protein 1	2,03595	1,86493
1876	COEA1_MOUSE	Col14a1	Collagen alpha-1(XIV) chain	0,609592	-0,430096
1877	UBP2L_MOUSE	Ubap2l	Ubiquitin-associated protein 2-like	0,374296	0,129609
1878	PELO_MOUSE	Pelo	Protein pelota homolog	0,329585	0,196249
1879	C2C2L_MOUSE	C2cd2l	Phospholipid transfer protein C2CD2L	1,26599	0,456392
1880	RT07_MOUSE	Mrps7	28S ribosomal protein S7, mitochondrial	0,261113	0,170663
1881	FLNB_MOUSE	Flnb	Filamin-B	1,1702	0,378184
1882	RRAGA_MOUSE	Rraga	Ras-related GTP-binding protein A	1,5612	0,241077
1883	IF4G3_MOUSE	Eif4g3	Eukaryotic translation initiation factor 4 gamma 3	0,533789	0,205493
1884	ACD11_MOUSE	Acad11	Acyl-CoA dehydrogenase family member 11	1,99903	0,91016
1885	BDH_MOUSE	Bdh1	D-beta-hydroxybutyrate dehydrogenase, mitochondrial	1,70719	0,627647
1886	GLRX5_MOUSE	Glrx5	Glutaredoxin-related protein 5, mitochondrial	0,370885	0,162933
1887	BSDC1_MOUSE	Bsdc1	BSD domain-containing protein 1	1,95104	1,44483
1888	RNZ2_MOUSE	Elac2	Zinc phosphodiesterase ELAC protein 2	0,127928	0,0550945
1889	FA12_MOUSE	F12	Coagulation factor XII	0,0190996	0,0377106
1890	SUV3_MOUSE	Supv3l1	ATP-dependent RNA helicase SUPV3L1, mitochondrial	0,479463	-0,306247
1891	DAPK1_MOUSE	Dapk1	Death-associated protein kinase 1	1,14587	0,467315
1892	ZN598_MOUSE	Znf598	E3 ubiquitin-protein ligase ZNF598	0,0937083	-0,208786
1893	SAFB2_MOUSE	Safb2	Scaffold attachment factor B2	1,01321	-0,479982
1894	NIPA_MOUSE	Zc3hc1	Nuclear-interacting partner of ALK	0,735206	0,609483
1895	TENA_MOUSE	Tnc	Tenascin	0,0578076	-0,0783772
1896	RT10_MOUSE	Mrps10	28S ribosomal protein S10, mitochondrial	0,42807	-0,212319
1897	ABITM_MOUSE	Abitram	Protein Abitram	1,01899	0,739747
1898	RT26_MOUSE	Mrps26	28S ribosomal protein S26, mitochondrial	0,0443329	-0,0218884
1899	DDX42_MOUSE	Ddx42	ATP-dependent RNA helicase DDX42	0,600391	0,239278
1900	ANFY1_MOUSE	Ankfy1	Rabankyrin-5	0,227528	-0,0991077
1901	ZFYV1_MOUSE	Zfyve1	Zinc finger FYVE domain-containing protein 1	0,201035	0,164263
1902	CCD50_MOUSE	Ccdc50	Coiled-coil domain-containing protein 50	1,41239	0,972843
1903	DLG1_MOUSE	Dlg1	Disks large homolog 1	0,493371	0,119051
1904	MFN1_MOUSE	Mfn1	Mitofusin-1	0,0630869	-0,0267216
1905	CPEB2_MOUSE	Cpeb2	Cytoplasmic polyadenylation element-binding protein 2	0,0312988	0,0575455
1906	PDK1_MOUSE	Pdk1	[Pyruvate dehydrogenase (acetyl-transferring)] kinase isozyme 1, mitochonc	0,774069	-0,676941
1907	WDR82_MOUSE	Wdr82	WD repeat-containing protein 82	0,580764	0,457205
1908	GALD1_MOUSE	Gatd1	Glutamine amidotransferase-like class 1 domain-containing protein 1	0,27235	0,110698
1909	GNS_MOUSE	Gns	N-acetylglucosamine-6-sulfatase	0,105161	0,129334
1910	EFTU_MOUSE	Tufm	Elongation factor Tu, mitochondrial	0,481101	0,274041
1911	ZFAN1_MOUSE	Zfand1	AN1-type zinc finger protein 1	0,482618	0,556404
1912	CPPED_MOUSE	Cpped1	Serine/threonine-protein phosphatase CPPED1	0,475985	-0,501226
1913	RN214_MOUSE	Rnf214	RING finger protein 214	1,03975	1,17102
1914	LPP_MOUSE	Lpp	Lipoma-preferred partner homolog	1,25597	0,311158
1915	PEF1_MOUSE	Pef1	Peflin	0,111275	-0,0965874
1916	TNPO1_MOUSE	Tnpo1	Transportin-1	0,25783	-0,170502
1917	ACTBL_MOUSE	Actbl2	Beta-actin-like protein 2	0,473468	-0,220579
1918	ERLN2_MOUSE	Erlin2	Erlin-2	0,82935	0,550473
1919	ROA3_MOUSE	Hnrnpa3	Heterogeneous nuclear ribonucleoprotein A3	0,306988	-0,246338

1920	PLD4_MOUSE	Pld4	Phospholipase D4	0,199607	-0,199932
1921	PSD11_MOUSE	Psmd11	26S proteasome non-ATPase regulatory subunit 11	1,04884	0,461818
1922	MIRO1_MOUSE	Rhot1	Mitochondrial Rho GTPase 1	0,484468	0,37942
1923	EFR3A_MOUSE	Efr3a	Protein EFR3 homolog A	0,322565	0,130927
1924	PDIP3_MOUSE	Poldip3	Polymerase delta-interacting protein 3	0,530555	0,453499
1925	COMD7_MOUSE	Commd7	COMM domain-containing protein 7	0,41197	-0,38155
1926	ACSM5_MOUSE	Acsm5	Acyl-coenzyme A synthetase ACSM5, mitochondrial	0,0894483	-0,0295593
1927	OXA1L_MOUSE	Oxa1l	Mitochondrial inner membrane protein OXA1L	0,00182565	0,00296364
1928	ENOPH_MOUSE	Enoph1	Enolase-phosphatase E1	0,308222	0,25746
1929	HTSF1_MOUSE	Htatsf1	HIV Tat-specific factor 1 homolog	2,25386	1,27979
1930	PTGR3_MOUSE	Zadh2	Prostaglandin reductase-3	0,966029	0,388615
1931	IF4B_MOUSE	Eif4b	Eukaryotic translation initiation factor 4B	0,902158	0,547239
1932	PAN2_MOUSE	Pan2	PAN2-PAN3 deadenylation complex catalytic subunit Pan2	0,239118	0,402027
1933	SAM50_MOUSE	Samm50	Sorting and assembly machinery component 50 homolog	0,393662	-0,186278
1934	SYAC_MOUSE	Aars	Alanine--tRNA ligase, cytoplasmic	0,570271	0,226088
1935	LRC8D_MOUSE	Lrrc8d	Volume-regulated anion channel subunit LRRC8D	0,853405	0,396204
1936	UBCP1_MOUSE	Ublcp1	Ubiquitin-like domain-containing CTD phosphatase 1	0,524418	0,25769
1937	E41L5_MOUSE	Epb41l5	Band 4.1-like protein 5	0,833351	0,457466
1938	BOLA2_MOUSE	Bola2	BolA-like protein 2	1,52523	0,524736
1939	CEPT1_MOUSE	Cept1	Choline/ethanolaminephosphotransferase 1	0,01708	-0,0157558
1940	ALAT2_MOUSE	Gpt2	Alanine aminotransferase 2	0,0565863	-0,0609837
1941	SYNM_MOUSE	Nars2	Probable asparagine--tRNA ligase, mitochondrial	0,12779	0,110661
1942	MID51_MOUSE	Mief1	Mitochondrial dynamics protein MID51	0,293883	0,285461
1943	FTO_MOUSE	Fto	Alpha-ketoglutarate-dependent dioxygenase FTO	0,7133	0,978613
1944	TIM29_MOUSE	Timm29	Mitochondrial import inner membrane translocase subunit Tim29	1,4487	0,6491
1945	CDC23_MOUSE	Cdc23	Cell division cycle protein 23 homolog	0,00915276	-0,0104225
1946	AL8A1_MOUSE	Aldh8a1	2-aminomuconic semialdehyde dehydrogenase	0,894251	0,134177
1947	PCKGM_MOUSE	Pck2	Phosphoenolpyruvate carboxykinase [GTP], mitochondrial	0,632575	0,310028
1948	TM9S4_MOUSE	Tm9sf4	Transmembrane 9 superfamily member 4	0,69099	-0,32507
1949	CO8B_MOUSE	C8b	Complement component C8 beta chain	0,151273	-0,301268
1950	WASF2_MOUSE	Wasf2	Wiskott-Aldrich syndrome protein family member 2	0,616834	0,237748
1951	TIPRL_MOUSE	Tiprl	TIP41-like protein	0,158673	0,0692131
1952	CMC1_MOUSE	Slc25a12	Calcium-binding mitochondrial carrier protein Aralar1	0,840535	-0,479828
1953	GOPC_MOUSE	Gopc	Golgi-associated PDZ and coiled-coil motif-containing protein	0,647002	-0,287512
1954	EHD2_MOUSE	Ehd2	EH domain-containing protein 2	0,0760022	-0,104786
1955	SPS1_MOUSE	Sephs1	Selenide, water dikinase 1	0,666196	-0,260571
1956	GLUCM_MOUSE	Dglucy	D-glutamate cyclase, mitochondrial	0,0481421	-0,0164169
1957	ECHM_MOUSE	Echs1	Enoyl-CoA hydratase, mitochondrial	0,028439	-0,0131943
1958	RCN3_MOUSE	Rcn3	Reticulocalbin-3	0,779659	-0,36624
1959	DTD2_MOUSE	Dtd2	D-aminoacyl-tRNA deacylase 2	0,0331648	-0,0319927
1960	LYVE1_MOUSE	Lyve1	Lymphatic vessel endothelial hyaluronic acid receptor 1	0,15616	0,0801857
1961	DCAKD_MOUSE	Dcakd	Dephospho-CoA kinase domain-containing protein	0,433866	0,300973
1962	FUT11_MOUSE	Fut11	Alpha-(1,3)-fucosyltransferase 11	0,199293	0,145209
1963	PTBP3_MOUSE	Ptbp3	Polypyrimidine tract-binding protein 3	0,0711624	-0,0347477
1964	NRDC_MOUSE	Nrdc	Nardilysin	0,635062	-0,257489
1965	CZIB_MOUSE	Czib	CXXC motif containing zinc binding protein	0,40163	-0,528772
1966	CGBP1_MOUSE	Cggbp1	CGG triplet repeat-binding protein 1	0,635179	-1,11218
1967	ELMO2_MOUSE	Elmo2	Engulfment and cell motility protein 2	0,446209	-0,409751
1968	PSMF1_MOUSE	Psmf1	Proteasome inhibitor PI31 subunit	0,188335	0,0960537
1969	PPM1L_MOUSE	Ppm1l	Protein phosphatase 1L	1,23888	0,433543
1970	TXLNG_MOUSE	Txlng	Gamma-taxilin	0,444963	-0,441807
1971	GANAB_MOUSE	Ganab	Neutral alpha-glucosidase AB	1,01587	0,449488

1972	RBM22_MOUSE	Rbm22	Pre-mRNA-splicing factor RBM22	0,284546	0,148745
1973	CARF_MOUSE	Cdkn2aip	CDKN2A-interacting protein	0,499105	0,214613
1974	TGO1_MOUSE	Mia3	Transport and Golgi organization protein 1 homolog	0,268035	0,152037
1975	SP130_MOUSE	Sap130	Histone deacetylase complex subunit SAP130	0,90511	0,410973
1976	SYIM_MOUSE	Iars2	Isoleucine--tRNA ligase, mitochondrial	0,106455	0,0493736
1977	RUFY1_MOUSE	Rufy1	RUN and FYVE domain-containing protein 1	0,716688	0,346671
1978	HOOK1_MOUSE	Hook1	Protein Hook homolog 1	1,05204	0,398801
1979	SYDM_MOUSE	Dars2	Aspartate--tRNA ligase, mitochondrial	0,794745	0,36309
1980	CSTF2_MOUSE	Cstf2	Cleavage stimulation factor subunit 2	0,748225	0,469099
1981	PRUN1_MOUSE	Prune1	Exopolyphosphatase PRUNE1	0,682167	0,284843
1982	ZC3HE_MOUSE	Zc3h14	Zinc finger CCCH domain-containing protein 14	0,350222	0,191445
1983	NAGPA_MOUSE	Nagpa	N-acetylglucosamine-1-phosphodiester alpha-N-acetylglucosaminidase	1,37287	0,484982
1984	CHDH_MOUSE	Chdh	Choline dehydrogenase, mitochondrial	0,00598927	0,00287933
1985	NUP93_MOUSE	Nup93	Nuclear pore complex protein Nup93	0,919757	0,180553
1986	ZN771_MOUSE	Znf771	Zinc finger protein 771	0,0254334	-0,0312817
1987	CHM2B_MOUSE	Chmp2b	Charged multivesicular body protein 2b	0,368403	0,216391
1988	SUN2_MOUSE	Sun2	SUN domain-containing protein 2	0,096177	-0,0934654
1989	SGTA_MOUSE	Sgta	Small glutamine-rich tetratricopeptide repeat-containing protein alpha	0,951927	0,635886
1990	RF1ML_MOUSE	Mtrf1l	Peptide chain release factor 1-like, mitochondrial	0,650623	-0,666568
1991	EIF2A_MOUSE	Eif2a	Eukaryotic translation initiation factor 2A	0,0244813	0,0212086
1992	PSMD5_MOUSE	Psmd5	26S proteasome non-ATPase regulatory subunit 5	0,331785	0,162709
1993	RT35_MOUSE	Mrps35	28S ribosomal protein S35, mitochondrial	0,0707552	-0,0696228
1994	TMM11_MOUSE	Tmem11	Transmembrane protein 11, mitochondrial	0,542064	-0,812857
1995	EST2E_MOUSE	Ces2e	Pyrethroid hydrolase Ces2e	0,0338409	-0,0156818
1996	KC1A_MOUSE	Csnk1a1	Casein kinase I isoform alpha	0,696259	0,193187
1997	AHSA1_MOUSE	Ahsa1	Activator of 90 kDa heat shock protein ATPase homolog 1	0,634598	0,318943
1998	RCC2_MOUSE	Rcc2	Protein RCC2	0,199299	0,099678
1999	RT27_MOUSE	Mrps27	28S ribosomal protein S27, mitochondrial	0,674512	0,220486
2000	IPO5_MOUSE	Ipo5	Importin-5	0,572318	-0,60918
2001	PTK7_MOUSE	Ptk7	Inactive tyrosine-protein kinase 7	0,782173	1,13159
2002	TNR6B_MOUSE	Tnrc6b	Trinucleotide repeat-containing gene 6B protein	0,190046	0,322943
2003	PUM3_MOUSE	Pum3	Pumilio homolog 3	0,674892	0,317653
2004	THOC5_MOUSE	Thoc5	THO complex subunit 5 homolog	0,914433	1,23292
2005	BAIP2_MOUSE	Baiap2	Brain-specific angiogenesis inhibitor 1-associated protein 2	0,285677	0,192719
2006	ODPX_MOUSE	Pdhx	Pyruvate dehydrogenase protein X component, mitochondrial	0,695408	0,366899
2007	EEA1_MOUSE	Eea1	Early endosome antigen 1	0,0603286	-0,090588
2008	SRSF7_MOUSE	Srsf7	Serine/arginine-rich splicing factor 7	1,06927	0,299463
2009	ERG7_MOUSE	Lss	Lanosterol synthase	0,260936	-0,224196
2010	SMOC1_MOUSE	Smoc1	SPARC-related modular calcium-binding protein 1	0,159939	0,267732
2011	SYTC2_MOUSE	Tarsl2	Threonine--tRNA ligase 2, cytoplasmic	0,269465	0,239146
2012	TM214_MOUSE	Tmem214	Transmembrane protein 214	0,792637	0,202372
2013	HSP13_MOUSE	Hspa13	Heat shock 70 kDa protein 13	0,147194	0,137937
2014	SRP68_MOUSE	Srp68	Signal recognition particle subunit SRP68	0,93639	0,348974
2015	IF4E2_MOUSE	Eif4e2	Eukaryotic translation initiation factor 4E type 2	0,850857	0,429987
2016	ODP2_MOUSE	Dlat	Dihydrolipoyllysine-residue acetyltransferase component of pyruvate dehydr	0,178468	0,129704
2017	RBGPR_MOUSE	Rab3gap2	Rab3 GTPase-activating protein non-catalytic subunit	0,31152	0,235061
2018	SYLC_MOUSE	Lars	Leucine--tRNA ligase, cytoplasmic	0,988461	0,419984
2019	CKAP4_MOUSE	Ckap4	Cytoskeleton-associated protein 4	0,0375689	0,017099
2020	SYQ_MOUSE	Qars	Glutamine--tRNA ligase	0,854435	0,403695
2021	GCP60_MOUSE	Acbd3	Golgi resident protein GCP60	0,206676	-0,177502
2022	ECHA_MOUSE	Hadha	Trifunctional enzyme subunit alpha, mitochondrial	0,710322	0,295385
2023	SEN34_MOUSE	Tsen34	tRNA-splicing endonuclease subunit Sen34	0,792793	0,471888

2024	QSOX1_MOUSE	Qsox1	Sulfhydryl oxidase 1	0,172286	0,142533
2025	ARMC6_MOUSE	Armc6	Armadillo repeat-containing protein 6	1,29303	0,601952
2026	SFR1_MOUSE	Sfr1	Swi5-dependent recombination DNA repair protein 1 homolog	0,0314563	0,0355473
2027	PPA6_MOUSE	Acp6	Lysophosphatidic acid phosphatase type 6	0,0302135	-0,0292572
2028	SYNC_MOUSE	Nars	Asparagine--tRNA ligase, cytoplasmic	0,0279276	0,0167908
2029	MAP11_MOUSE	Metap1	Methionine aminopeptidase 1	0,00490413	-0,00285187
2030	PGGHG_MOUSE	Pgghg	Protein-glucosylgalactosylhydroxylysine glucosidase	0,163058	0,145412
2031	RL24_MOUSE	Rpl24	60S ribosomal protein L24	0,868871	0,393829
2032	RFOX1_MOUSE;RF	Rbfox1;Rbfox2	RNA binding protein fox-1 homolog 2	0,0947072	-0,140358
2033	RCN2_MOUSE	Rcn2	Reticulocalbin-2	0,109456	0,181149
2034	TM177_MOUSE	Tmem177	Transmembrane protein 177	0,0867179	-0,0982277
2035	DAAM1_MOUSE	Daam1	Disheveled-associated activator of morphogenesis 1	1,20026	0,455787
2036	PPR18_MOUSE	Ppp1r18	Phostensin	2,17497	0,877172
2037	CNPY4_MOUSE	Cnpy4	Protein canopy homolog 4	0,851514	0,428481
2038	HEAT3_MOUSE	Heatr3	HEAT repeat-containing protein 3	0,427831	0,255053
2039	SCFD1_MOUSE	Scfd1	Sec1 family domain-containing protein 1	1,50357	0,546238
2040	TMTC3_MOUSE	Tmtc3	Protein O-mannosyl-transferase TMTC3	0,95368	0,503251
2041	AAPK2_MOUSE	Prkaa2	5'-AMP-activated protein kinase catalytic subunit alpha-2	0,181342	0,109032
2042	C2D1B_MOUSE	Cc2d1b	Coiled-coil and C2 domain-containing protein 1B	1,25229	0,787185
2043	CLAP2_MOUSE	Clasp2	CLIP-associating protein 2	0,437897	-0,207156
2044	RMD2_MOUSE	Rmdn2	Regulator of microtubule dynamics protein 2	0,0942397	0,0930664
2045	PISD_MOUSE	Pisd	Phosphatidylserine decarboxylase proenzyme, mitochondrial	0,139286	-0,117455
2046	KS6B1_MOUSE	Rps6kb1	Ribosomal protein S6 kinase beta-1	0,168642	-0,255527
2047	PB1_MOUSE	Pbrm1	Protein polybromo-1	0,0438557	-0,0255455
2048	ASPH_MOUSE	Asph	Aspartyl/asparaginyl beta-hydroxylase	1,02296	-0,284893
2049	CPNE3_MOUSE	Cpne3	Copine-3	0,6842	-1,30588
2050	SRRM2_MOUSE	Srrm2	Serine/arginine repetitive matrix protein 2	0,774592	0,378376
2051	FLNA_MOUSE	Flna	Filamin-A	0,47744	-0,364258
2052	DUS28_MOUSE	Dusp28	Dual specificity phosphatase 28	0,613361	0,563402
2053	NUP54_MOUSE	Nup54	Nuclear pore complex protein Nup54	0,899072	-0,560321
2054	CPSF7_MOUSE	Cpsf7	Cleavage and polyadenylation specificity factor subunit 7	0,296026	-0,122895
2055	EXOS6_MOUSE	Exosc6	Exosome complex component MTR3	0,01994	0,00933838
2056	PAK4_MOUSE	Pak4	Serine/threonine-protein kinase PAK 4	0,422738	0,434448
2057	KAT1_MOUSE	Kyat1	Kynurenine--oxoglutarate transaminase 1	0,715442	-0,68559
2058	SCFD2_MOUSE	Scfd2	Sec1 family domain-containing protein 2	0,142627	0,809373
2059	GMPPB_MOUSE	Gmppb	Mannose-1-phosphate guanyltransferase beta	0,0407147	0,117131
2060	SEC62_MOUSE	Sec62	Translocation protein SEC62	2,64758	0,661737
2061	SYIC_MOUSE	Iars	Isoleucine--tRNA ligase, cytoplasmic	0,649951	0,115409
2062	RAP2C_MOUSE	Rap2c	Ras-related protein Rap-2c	0,210238	-0,192009
2063	ILVBL_MOUSE	Ilvbl	Acetolactate synthase-like protein	1,12617	0,783635
2064	RM22_MOUSE	Mrpl22	39S ribosomal protein L22, mitochondrial	0,0732229	0,123106
2065	AIFM2_MOUSE	Aifm2	Apoptosis-inducing factor 2	0,839662	0,979709
2066	HOOK3_MOUSE	Hook3	Protein Hook homolog 3	1,51422	0,776489
2067	DOCK1_MOUSE	Dock1	Dedicator of cytokinesis protein 1	0,344047	0,134271
2068	GEPH_MOUSE	Gphn	Gephyrin	0,6652	0,363933
2069	LDAH_MOUSE	Ldah	Lipid droplet-associated hydrolase	0,0149107	0,021035
2070	VATH_MOUSE	Atp6v1h	V-type proton ATPase subunit H	1,14648	0,388742
2071	DPP9_MOUSE	Dpp9	Dipeptidyl peptidase 9	2,44622	0,855169
2072	DHPR_MOUSE	Qdpr	Dihydropteridine reductase	1,0259	-0,308097
2073	STX16_MOUSE	Stx16	Syntaxin-16	1,38572	0,439839
2074	PPME1_MOUSE	Ppme1	Protein phosphatase methylesterase 1	0,352457	0,122422
2075	NUDT9_MOUSE	Nudt9	ADP-ribose pyrophosphatase, mitochondrial	1,47183	0,579341

2076	TRI14_MOUSE	Trim14	Tripartite motif-containing protein 14	1,02656	-0,494736
2077	RL1D1_MOUSE	Rsl1d1	Ribosomal L1 domain-containing protein 1	0,550963	0,101857
2078	AOFB_MOUSE	Maob	Amine oxidase [flavin-containing] B	0,122111	-0,122665
2079	SSDH_MOUSE	Aldh5a1	Succinate-semialdehyde dehydrogenase, mitochondrial	0,115821	0,0412621
2080	GIMA5_MOUSE	Gimap5	GTPase IMAP family member 5	0,773337	1,03533
2081	PGES2_MOUSE	Ptges2	Prostaglandin E synthase 2	0,119488	0,095343
2082	ACOT4_MOUSE	Acot4	Peroxisomal succinyl-coenzyme A thioesterase	0,100093	0,160025
2083	TTPA_MOUSE	Ttpa	Alpha-tocopherol transfer protein	0,157079	0,0698692
2084	UD2A3_MOUSE	Ugt2a3	UDP-glucuronosyltransferase 2A3	0,0939922	-0,0955601
2085	VP35L_MOUSE	Vps35l	VPS35 endosomal protein sorting factor-like	0,082641	0,141569
2086	RHPN2_MOUSE	Rhpn2	Rhophilin-2	0,0294227	0,0475925
2087	GRIN3_MOUSE	Gprin3	G protein-regulated inducer of neurite outgrowth 3	1,09129	0,501376
2088	THIM_MOUSE	Acaa2	3-ketoacyl-CoA thiolase, mitochondrial	0,228132	0,084037
2089	OSGEP_MOUSE	Osgep	Probable tRNA N6-adenosine threonylcarbamoyltransferase	0,826081	1,16699
2090	AT2L1_MOUSE	Etnppl	Ethanolamine-phosphate phospho-lyase	0,0255438	-0,0224091
2091	LARP4_MOUSE	Larp4	La-related protein 4	0,772016	0,255219
2092	PKN2_MOUSE	Pkn2	Serine/threonine-protein kinase N2	0,831111	0,381527
2093	ERF1_MOUSE	Etf1	Eukaryotic peptide chain release factor subunit 1	0,455167	-0,211883
2094	NAA25_MOUSE	Naa25	N-alpha-acetyltransferase 25, NatB auxiliary subunit	0,189997	-0,110383
2095	KANK2_MOUSE	Kank2	KN motif and ankyrin repeat domain-containing protein 2	0,187419	-0,0972355
2096	RBBP5_MOUSE	Rbbp5	Retinoblastoma-binding protein 5	0,315055	-0,151977
2097	PGAM5_MOUSE	Pgam5	Serine/threonine-protein phosphatase PGAM5, mitochondrial	1,03234	0,375226
2098	GEMI5_MOUSE	Gemin5	Gem-associated protein 5	1,58907	0,2596
2099	VP13C_MOUSE	Vps13c	Vacuolar protein sorting-associated protein 13C	0,0478591	0,0315044
2100	FND3A_MOUSE	Fndc3a	Fibronectin type-III domain-containing protein 3A	0,368935	0,249622
2101	OSBL2_MOUSE	Osbpl2	Oxysterol-binding protein-related protein 2	1,49676	1,56892
2102	COMD2_MOUSE	Commd2	COMM domain-containing protein 2	0,633803	0,3845
2103	TMX3_MOUSE	Tmx3	Protein disulfide-isomerase TMX3	0,200099	0,338495
2104	HAT1_MOUSE	Hat1	Histone acetyltransferase type B catalytic subunit	0,749333	0,998552
2105	UBP47_MOUSE	Usp47	Ubiquitin carboxyl-terminal hydrolase 47	0,143473	0,107295
2106	TAOK3_MOUSE	Taok3	Serine/threonine-protein kinase TAO3	0,694374	0,356584
2107	YTHD3_MOUSE	Ythdf3	YTH domain-containing family protein 3	0,122295	0,048275
2108	SYYM_MOUSE	Yars2	Tyrosine--tRNA ligase, mitochondrial	0,80932	-0,392201
2109	TOIP2_MOUSE	Tor1aip2	Torsin-1A-interacting protein 2	1,03539	0,583437
2110	RHG25_MOUSE	Arhgap25	Rho GTPase-activating protein 25	0,924078	0,666846
2111	ABI3_MOUSE	Abi3	ABI gene family member 3	1,98874	0,695882
2112	ELMO3_MOUSE	Elmo3	Engulfment and cell motility protein 3	0,0696808	-0,0498158
2113	ODC_MOUSE	Slc25a21	Mitochondrial 2-oxodicarboxylate carrier	0,419213	-0,264148
2114	PAR12_MOUSE	Parp12	Protein mono-ADP-ribosyltransferase PARP12	0,67341	0,237835
2115	DYN3_MOUSE	Dnm3	Dynamin-3	0,356898	0,666938
2116	COAC_MOUSE	Ppcdc	Phosphopantothenoylcysteine decarboxylase	0,836788	0,858025
2117	NCBP3_MOUSE	Ncbp3	Nuclear cap-binding protein subunit 3	0,268072	0,160109
2118	NHLC2_MOUSE	Nhlrc2	NHL repeat-containing protein 2	0,611331	-0,203595
2119	MAP1S_MOUSE	Map1s	Microtubule-associated protein 1S	1,24515	0,797463
2120	ZHX2_MOUSE	Zhx2	Zinc fingers and homeoboxes protein 2	0,989682	0,637095
2121	SYFA_MOUSE	Farsa	Phenylalanine--tRNA ligase alpha subunit	0,123595	-0,114316
2122	EFL1_MOUSE	Efl1	Elongation factor-like GTPase 1	0,718042	0,442875
2123	VP26B_MOUSE	Vps26b	Vacuolar protein sorting-associated protein 26B	0,800819	0,263201
2124	A16L1_MOUSE	Atg16l1	Autophagy-related protein 16-1	0,611869	0,375342
2125	GDC_MOUSE	Slc25a16	Graves disease carrier protein homolog	0,302498	0,117944
2126	PAOX_MOUSE	Paox	Peroxisomal N(1)-acetyl-spermine/spermidine oxidase	0,210612	0,178462
2127	COG5_MOUSE	Cog5	Conserved oligomeric Golgi complex subunit 5	0,0729225	0,038855

2128	WDR59_MOUSE	Wdr59	GATOR complex protein WDR59	1,12895	0,964104
2129	DI3L1_MOUSE	Dis3l	DIS3-like exonuclease 1	0,276012	-0,33274
2130	F234A_MOUSE	Fam234a	Protein FAM234A	0,283707	-0,182275
2131	LCAP_MOUSE	Lnpep	Leucyl-cystinyl aminopeptidase	0,599803	-0,298388
2132	P20D1_MOUSE	Pm20d1	N-fatty-acyl-amino acid synthase/hydrolase PM20D1	0,54941	-0,263658
2133	CPNE1_MOUSE	Cpne1	Copine-1	1,1024	0,367587
2134	CPSM_MOUSE	Cps1	Carbamoyl-phosphate synthase [ammonia], mitochondrial	0,10831	0,0660789
2135	THOP1_MOUSE	Thop1	Thimet oligopeptidase	1,38785	0,356605
2136	SEP11_MOUSE	Septin11	Septin-11	0,458448	0,208546
2137	IWS1_MOUSE	Iws1	Protein IWS1 homolog	0,707291	0,808299
2138	PTCD1_MOUSE	Ptcd1	Pentatricopeptide repeat-containing protein 1, mitochondrial	0,32892	0,245743
2139	RBM14_MOUSE	Rbm14	RNA-binding protein 14	0,769374	0,237338
2140	CASP9_MOUSE	Casp9	Caspase-9	1,29048	1,66672
2141	GUF1_MOUSE	Guf1	Translation factor Guf1, mitochondrial	2,52649	-0,477719
2142	HTR5B_MOUSE	Heatr5b	HEAT repeat-containing protein 5B	0,223503	-0,220031
2143	RAE1L_MOUSE	Rae1	mRNA export factor	1,76731	0,487053
2144	NAKD2_MOUSE	Nadk2	NAD kinase 2, mitochondrial	0,117915	-0,0771355
2145	CWC22_MOUSE	Cwc22	Pre-mRNA-splicing factor CWC22 homolog	0,339997	-0,156182
2146	NT5D1_MOUSE	Nt5dc1	5'-nucleotidase domain-containing protein 1	1,70395	2,36977
2147	PRSR2_MOUSE	Proser2	Proline and serine-rich protein 2	0,187598	-0,215893
2148	CLMN_MOUSE	Clmn	Calmin	0,113505	0,077663
2149	TBCEL_MOUSE	Tbcel	Tubulin-specific chaperone cofactor E-like protein	0,044198	-0,031609
2150	SEP10_MOUSE	Septin10	Septin-10	0,49361	0,219878
2151	WDR26_MOUSE	Wdr26	WD repeat-containing protein 26	0,599737	-0,359936
2152	CO6A6_MOUSE	Col6a6	Collagen alpha-6(VI) chain	0,281914	-0,518025
2153	STBD1_MOUSE	Stbd1	Starch-binding domain-containing protein 1	0,483748	0,289977
2154	CSTFT_MOUSE	Cstf2t	Cleavage stimulation factor subunit 2 tau variant	0,874772	0,495427
2155	APOA5_MOUSE	Apoa5	Apolipoprotein A-V	1,18566	0,651405
2156	MMAA_MOUSE	Mmaa	Methylmalonic aciduria type A homolog, mitochondrial	0,211	0,0943569
2157	RBM4_MOUSE	Rbm4	RNA-binding protein 4	0,321983	0,258806
2158	UBA6_MOUSE	Uba6	Ubiquitin-like modifier-activating enzyme 6	0,53041	0,222373
2159	UTP15_MOUSE	Utp15	U3 small nucleolar RNA-associated protein 15 homolog	0,422192	-0,310003
2160	EMC1_MOUSE	Emc1	ER membrane protein complex subunit 1	0,317311	0,100379
2161	MYEF2_MOUSE	Myef2	Myelin expression factor 2	0,46645	0,18427
2162	LIPB1_MOUSE	Ppfibp1	Liprin-beta-1	0,0947393	-0,0385601
2163	DIDO1_MOUSE	Dido1	Death-inducer obliterator 1	0,0825265	0,0863323
2164	PGM2L_MOUSE	Pgm2l1	Glucose 1,6-bisphosphate synthase	0,719631	0,509325
2165	CAF17_MOUSE	Iba57	Putative transferase CAF17 homolog, mitochondrial	0,728017	0,896618
2166	RYDEN_MOUSE	Ryden	Repressor of yield of DENV protein homolog	1,19211	0,446339
2167	MIC60_MOUSE	Immt	MICOS complex subunit Mic60	0,073894	0,0358398
2168	PARP9_MOUSE	Parp9	Protein mono-ADP-ribosyltransferase PARP9	0,997827	0,491103
2169	THIC_MOUSE	Acat2	Acetyl-CoA acetyltransferase, cytosolic	0,547978	0,415954
2170	ELOA1_MOUSE	Eloa	Elongin-A	0,479353	0,682163
2171	ABI1_MOUSE	Abi1	Abl interactor 1	0,232095	0,274041
2172	GATC_MOUSE	Gatc	Glutamyl-tRNA(Gln) amidotransferase subunit C, mitochondrial	0,189992	-0,30984
2173	PNCB_MOUSE	Naprt	Nicotinate phosphoribosyltransferase	0,309022	0,0647335
2174	VWA8_MOUSE	Vwa8	von Willebrand factor A domain-containing protein 8	0,172448	-0,0591244
2175	VPS53_MOUSE	Vps53	Vacuolar protein sorting-associated protein 53 homolog	0,00653348	0,0060791
2176	PRP31_MOUSE	Prpf31	U4/U6 small nuclear ribonucleoprotein Prp31	1,13435	0,288769
2177	UFL1_MOUSE	Ufl1	E3 UFM1-protein ligase 1	0,172883	0,109467
2178	BCAS3_MOUSE	Bcas3	Breast carcinoma-amplified sequence 3 homolog	0,532157	0,32564
2179	NEMF_MOUSE	Nemf	Nuclear export mediator factor Nemf	2,09221	0,579564

2180	PABP2_MOUSE	Pabpn1	Polyadenylate-binding protein 2	0,0512941	0,0325848
2181	TTC27_MOUSE	Ttc27	Tetratricopeptide repeat protein 27	0,686995	0,799102
2182	SAC2_MOUSE	Inpp5f	Phosphatidylinositide phosphatase SAC2	0,638959	-0,315097
2183	VCIP1_MOUSE	Vcpip1	Deubiquitinating protein VCIP135	1,3978	0,234094
2184	STON1_MOUSE	Ston1	Stonin-1	0,359381	0,188245
2185	TXNL1_MOUSE	Txnl1	Thioredoxin-like protein 1	1,17444	0,398537
2186	TRM6_MOUSE	Trmt6	tRNA (adenine(58)-N(1))-methyltransferase non-catalytic subunit TRM6	1,16303	0,633166
2187	RDH13_MOUSE	Rdh13	Retinol dehydrogenase 13	0,0895396	-0,0413929
2188	NAA30_MOUSE	Naa30	N-alpha-acetyltransferase 30	0,17912	-0,124557
2189	TAB1_MOUSE	Tab1	TGF-beta-activated kinase 1 and MAP3K7-binding protein 1	0,5628	0,556819
2190	GCST_MOUSE	Amt	Aminomethyltransferase, mitochondrial	0,30009	0,155629
2191	SNX8_MOUSE	Snx8	Sorting nexin-8	0,634503	0,378072
2192	HPF1_MOUSE	Hpf1	Histone PARylation factor 1	1,15827	-1,07117
2193	SYPM_MOUSE	Pars2	Probable proline--tRNA ligase, mitochondrial	0,230426	-0,271564
2194	RPB2_MOUSE	Polr2b	DNA-directed RNA polymerase II subunit RPB2	0,177433	-0,0850872
2195	S2545_MOUSE	Slc25a45	Solute carrier family 25 member 45	1,40118	0,512387
2196	AQR_MOUSE	Aqr	RNA helicase aquarius	0,361814	0,152972
2197	RIFK_MOUSE	Rfk	Riboflavin kinase	0,00836053	-0,00899696
2198	G6PE_MOUSE	H6pd	GDH/6PGL endoplasmic bifunctional protein	1,09282	0,385744
2199	CS1A_MOUSE	C1sa	Complement C1s-A subcomponent	0,633411	0,478553
2200	C1RA_MOUSE	C1ra	Complement C1r-A subcomponent	0,964029	0,403
2201	SMC2_MOUSE	Smc2	Structural maintenance of chromosomes protein 2	0,538641	0,26392
2202	RAB43_MOUSE	Rab43	Ras-related protein Rab-43	1,30672	0,370105
2203	ARHL2_MOUSE	Adprhl2	ADP-ribose glycohydrolase ARH3	0,846809	0,240579
2204	ARK72_MOUSE	Akr7a2	Aflatoxin B1 aldehyde reductase member 2	1,55539	0,285168
2205	ASPP2_MOUSE	Tp53bp2	Apoptosis-stimulating of p53 protein 2	0,822924	0,338322
2206	PPM1F_MOUSE	Ppm1f	Protein phosphatase 1F	0,891146	0,485361
2207	TNS2_MOUSE	Tns2	Tensin-2	0,136643	0,113025
2208	RBM28_MOUSE	Rbm28	RNA-binding protein 28	0,121941	0,0734276
2209	SYEP_MOUSE	Eprs	Bifunctional glutamate/proline--tRNA ligase	0,705359	0,480014
2210	TCRG1_MOUSE	Tcerg1	Transcription elongation regulator 1	0,541146	0,389513
2211	LONM_MOUSE	Lonp1	Lon protease homolog, mitochondrial	0,361653	0,29537
2212	H2B3A_MOUSE;H2E	Hist3h2ba;Hist3h	Histone H2B type 3-B	0,223233	-0,839099
2213	OGT1_MOUSE	Ogt	UDP-N-acetylglucosamine--peptide N-acetylglucosaminyltransferase 110 kD	0,227792	0,0879608
2214	CHERP_MOUSE	Cherp	Calcium homeostasis endoplasmic reticulum protein	0,28991	0,122749
2215	CCAR1_MOUSE	Ccar1	Cell division cycle and apoptosis regulator protein 1	0,152398	0,112252
2216	SLTM_MOUSE	Sltm	SAFB-like transcription modulator	0,575638	0,579611
2217	NUDT6_MOUSE	Nudt6	Nucleoside diphosphate-linked moiety X motif 6	0,288453	0,432916
2218	GCC2_MOUSE	Gcc2	GRIP and coiled-coil domain-containing protein 2	1,18533	0,454697
2219	SEPT8_MOUSE	Septin8	Septin-8	1,03143	0,474028
2220	PYM1_MOUSE	Pym1	Partner of Y14 and mago	1,19212	0,488522
2221	PHC3_MOUSE	Phc3	Polyhomeotic-like protein 3	1,73613	0,903966
2222	PGP_MOUSE	Pgp	Glycerol-3-phosphate phosphatase	1,43696	0,575294
2223	FBX4_MOUSE	Fbxo4	F-box only protein 4	0,63934	0,551349
2224	NT8F2_MOUSE	Nat8f2	N-acetyltransferase family 8 member 2	0,116926	-0,165529
2225	DPYD_MOUSE	Dpyd	Dihydropyrimidine dehydrogenase [NADP(+)]	0,716241	-0,35391
2226	AL4A1_MOUSE	Aldh4a1	Delta-1-pyrroline-5-carboxylate dehydrogenase, mitochondrial	0,0284787	-0,0170834
2227	NGEF_MOUSE	Ngef	Ephexin-1	0,955561	-0,675282
2228	EI2BE_MOUSE	Eif2b5	Translation initiation factor eIF-2B subunit epsilon	0,311954	0,31771
2229	P66A_MOUSE	Gatad2a	Transcriptional repressor p66 alpha	1,01935	1,0104
2230	COG3_MOUSE	Cog3	Conserved oligomeric Golgi complex subunit 3	0,647268	0,842055
2231	SLAI2_MOUSE	Slain2	SLAIN motif-containing protein 2	0,970677	0,538036

2232	GNL3_MOUSE	Gnl3	Guanine nucleotide-binding protein-like 3	0,197031	0,111776
2233	C19L1_MOUSE	Cwf19l1	CWF19-like protein 1	0,191052	-0,297008
2234	PDLI5_MOUSE	Pdlim5	PDZ and LIM domain protein 5	0,581661	0,380249
2235	STEA3_MOUSE	Steap3	Metalloreductase STEAP3	0,987321	1,95378
2236	DI3L2_MOUSE	Dis3l2	DIS3-like exonuclease 2	1,06313	0,218158
2237	RMND1_MOUSE	Rmnd1	Required for meiotic nuclear division protein 1 homolog	0,0815116	-0,11627
2238	PYGB_MOUSE	Pygb	Glycogen phosphorylase, brain form	0,398694	-0,120979
2239	OSB11_MOUSE	Osbpl11	Oxysterol-binding protein-related protein 11	0,276144	0,181796
2240	FERM2_MOUSE	Fermt2	Fermitin family homolog 2	0,110911	0,0455406
2241	FA20A_MOUSE	Fam20a	Pseudokinase FAM20A	0,123024	0,0686684
2242	PAR10_MOUSE	Parp10	Protein mono-ADP-ribosyltransferase PARP10	0,848509	0,627068
2243	COPA_MOUSE	Copa	Coatomer subunit alpha	1,0728	0,505145
2244	BTD_MOUSE	Btd	Biotinidase	1,44253	0,932742
2245	ANM5_MOUSE	Prmt5	Protein arginine N-methyltransferase 5	0,750973	0,402912
2246	PLCG2_MOUSE	Plcg2	1-phosphatidylinositol 4,5-bisphosphate phosphodiesterase gamma-2	0,313453	0,409399
2247	CP2DQ_MOUSE	Cyp2d26	Cytochrome P450 2D26	0,122907	-0,151976
2248	INT4_MOUSE	Ints4	Integrator complex subunit 4	0,4946	0,45706
2249	PAK2_MOUSE	Pak2	Serine/threonine-protein kinase PAK 2	0,208748	-0,090881
2250	TBCE_MOUSE	Tbce	Tubulin-specific chaperone E	0,737631	-0,281686
2251	VWF_MOUSE	Vwf	von Willebrand factor	0,449698	0,383487
2252	CIP4_MOUSE	Trip10	Cdc42-interacting protein 4	0,2696	0,664502
2253	ELYS_MOUSE	Ahctf1	Protein ELYS	2,44696	1,0053
2254	AGO3_MOUSE	Ago3	Protein argonaute-3	0,579135	0,296531
2255	AGO2_MOUSE	Ago2	Protein argonaute-2	0,232057	-0,0951038
2256	HMCS1_MOUSE	Hmgcs1	Hydroxymethylglutaryl-CoA synthase, cytoplasmic	0,946318	-0,60917
2257	THTPA_MOUSE	Thtpa	Thiamine-triphosphatase	0,377988	0,658632
2258	TFB1M_MOUSE	Tfb1m	Dimethyladenosine transferase 1, mitochondrial	0,134075	0,0844078
2259	CDC73_MOUSE	Cdc73	Parafibromin	1,05908	0,61189
2260	ACAD9_MOUSE	Acad9	Acyl-CoA dehydrogenase family member 9, mitochondrial	0,129888	-0,0788826
2261	MIRO2_MOUSE	Rhot2	Mitochondrial Rho GTPase 2	0,712353	1,27879
2262	AFG32_MOUSE	Afg3l2	AFG3-like protein 2	0,0639219	0,0400997
2263	EIF3B_MOUSE	Eif3b	Eukaryotic translation initiation factor 3 subunit B	0,604413	-0,231456
2264	ACSL5_MOUSE	Acsl5	Long-chain-fatty-acid--CoA ligase 5	1,3399	0,608212
2265	LIN7A_MOUSE	Lin7a	Protein lin-7 homolog A	0,864643	0,404958
2266	RM48_MOUSE	Mrpl48	39S ribosomal protein L48, mitochondrial	0,142766	-0,100618
2267	TXTP_MOUSE	Slc25a1	Tricarboxylate transport protein, mitochondrial	0,27406	0,171725
2268	NAGA_MOUSE	Amdhd2	N-acetylglucosamine-6-phosphate deacetylase	0,308512	0,45312
2269	SPF45_MOUSE	Rbm17	Splicing factor 45	0,173843	0,139099
2270	MRPP3_MOUSE	Prorp	Mitochondrial ribonuclease P catalytic subunit	0,185811	-0,178133
2271	UD3A2_MOUSE	Ugt3a2	UDP-glucuronosyltransferase 3A2	0,0332673	0,0315403
2272	AGRL2_MOUSE	Adgrl2	Adhesion G protein-coupled receptor L2	1,43548	1,58738
2273	OPLA_MOUSE	Oplah	5-oxoprolinase	1,4809	0,574147
2274	FBP1L_MOUSE	Fnbp1l	Formin-binding protein 1-like	2,6401	1,11129
2275	BCLF1_MOUSE	Bclaf1	Bcl-2-associated transcription factor 1	0,531803	-0,197923
2276	AKC1H_MOUSE	Akr1c18	Aldo-keto reductase family 1 member C18	0,355322	-0,665307
2277	CP51A_MOUSE	Cyp51a1	Lanosterol 14-alpha demethylase	0,363837	-0,397197
2278	EFGM_MOUSE	Gfm1	Elongation factor G, mitochondrial	0,10488	-0,066457
2279	FIBB_MOUSE	Fgb	Fibrinogen beta chain	0,035296	-0,0287567
2280	ESRP2_MOUSE	Esrp2	Epithelial splicing regulatory protein 2	0,118695	0,0553192
2281	ZBT20_MOUSE	Zbtb20	Zinc finger and BTB domain-containing protein 20	0,216985	0,158898
2282	RHG18_MOUSE	Arhgap18	Rho GTPase-activating protein 18	0,260282	0,487415
2283	CNOT3_MOUSE	Cnot3	CCR4-NOT transcription complex subunit 3	0,527818	0,348623

2284	TACO1_MOUSE	Taco1	Translational activator of cytochrome c oxidase 1	0,659198	0,669915
2285	PKHO2_MOUSE	Plekho2	Pleckstrin homology domain-containing family O member 2	0,300551	-0,12428
2286	GALM_MOUSE	Galm	Aldose 1-epimerase	1,13894	1,06511
2287	CO8A_MOUSE	C8a	Complement component C8 alpha chain	2,55146	0,821595
2288	PDXK_MOUSE	Pdxk	Pyridoxal kinase	0,593908	0,645486
2289	COXM2_MOUSE	Cmc2	COX assembly mitochondrial protein 2 homolog	0,547106	0,627691
2290	C2D1A_MOUSE	Cc2d1a	Coiled-coil and C2 domain-containing protein 1A	1,44824	1,26676
2291	STX5_MOUSE	Stx5	Syntaxin-5	0,234892	0,0730785
2292	ALKB3_MOUSE	Alkbh3	Alpha-ketoglutarate-dependent dioxygenase alkB homolog 3	0,293947	0,259301
2293	TDRD7_MOUSE	Tdrd7	Tudor domain-containing protein 7	0,294848	0,249368
2294	SPP24_MOUSE	Spp2	Secreted phosphoprotein 24	0,335957	0,327024
2295	WIPF1_MOUSE	Wipf1	WAS/WASL-interacting protein family member 1	0,482894	0,180857
2296	TRNT1_MOUSE	Trnt1	CCA tRNA nucleotidyltransferase 1, mitochondrial	0,136866	-0,160666
2297	DNM1L_MOUSE	Dnm1l	Dynamin-1-like protein	1,0143	-0,352351
2298	PLPL8_MOUSE	Pnpla8	Calcium-independent phospholipase A2-gamma	1,28306	-0,585342
2299	PHLB2_MOUSE	Phldb2	Pleckstrin homology-like domain family B member 2	0,474764	0,226596
2300	SPAS2_MOUSE	Spats2	Spermatogenesis-associated serine-rich protein 2	0,620541	0,58271
2301	PNPT1_MOUSE	Pnpt1	Polyribonucleotide nucleotidyltransferase 1, mitochondrial	1,15085	0,34413
2302	NEK9_MOUSE	Nek9	Serine/threonine-protein kinase Nek9	0,641028	-0,462592
2303	WDR11_MOUSE	Wdr11	WD repeat-containing protein 11	0,234254	0,165665
2304	KPCD3_MOUSE	Prkd3	Serine/threonine-protein kinase D3	0,697886	-0,786536
2305	COQ9_MOUSE	Coq9	Ubiquinone biosynthesis protein COQ9, mitochondrial	0,869734	-0,552087
2306	LYRM4_MOUSE	Lyrm4	LYR motif-containing protein 4	0,512954	0,331906
2307	NAT10_MOUSE	Nat10	RNA cytidine acetyltransferase	1,6648	0,262538
2308	UVRAG_MOUSE	Uvrag	UV radiation resistance-associated protein	1,05872	0,462172
2309	ABCF3_MOUSE	Abcf3	ATP-binding cassette sub-family F member 3	0,00979336	-0,0100945
2310	KT3K_MOUSE	Fn3krp	Ketosamine-3-kinase	0,204692	-0,337716
2311	TF3C1_MOUSE	Gtf3c1	General transcription factor 3C polypeptide 1	0,083487	0,0309643
2312	GT251_MOUSE	Colgalt1	Procollagen galactosyltransferase 1	0,674479	0,51206
2313	SDHA_MOUSE	Sdha	Succinate dehydrogenase [ubiquinone] flavoprotein subunit, mitochondrial	0,0673601	-0,0349113
2314	SIR5_MOUSE	Sirt5	NAD-dependent protein deacylase sirtuin-5, mitochondrial	0,431587	0,284167
2315	OS9_MOUSE	Os9	Protein OS-9	0,339115	0,338394
2316	GPAT4_MOUSE	Gpat4	Glycerol-3-phosphate acyltransferase 4	1,17815	0,544313
2317	HACD3_MOUSE	Hacd3	Very-long-chain (3R)-3-hydroxyacyl-CoA dehydratase 3	0,196115	-0,276266
2318	EDC3_MOUSE	Edc3	Enhancer of mRNA-decapping protein 3	0,197004	0,0605549
2319	LS14A_MOUSE	Lsm14a	Protein LSM14 homolog A	0,517765	-0,733085
2320	FMO2_MOUSE	Fmo2	Dimethylaniline monooxygenase [N-oxide-forming] 2	0,0848371	-0,0368492
2321	MANBA_MOUSE	Manba	Beta-mannosidase	0,26697	0,355304
2322	AGFG1_MOUSE	Agfg1	Arf-GAP domain and FG repeat-containing protein 1	0,0621576	-0,138726
2323	RM38_MOUSE	Mrpl38	39S ribosomal protein L38, mitochondrial	0,0936402	0,0634644
2324	COMD9_MOUSE	Commd9	COMM domain-containing protein 9	0,337726	0,121848
2325	SHOT1_MOUSE	Shtn1	Shootin-1	0,400142	0,353435
2326	NMRL1_MOUSE	Nmral1	NmrA-like family domain-containing protein 1	0,335944	0,171175
2327	UQCC3_MOUSE	Uqcc3	Ubiquinol-cytochrome-c reductase complex assembly factor 3	0,232152	-0,200957
2328	PAF1_MOUSE	Paf1	RNA polymerase II-associated factor 1 homolog	0,154652	0,0834667
2329	PP4R1_MOUSE	Ppp4r1	Serine/threonine-protein phosphatase 4 regulatory subunit 1	0,441257	0,289208
2330	RM47_MOUSE	Mrpl47	39S ribosomal protein L47, mitochondrial	0,161462	0,0776749
2331	MATR3_MOUSE	Matr3	Matrin-3	0,107615	0,082238
2332	CHAP1_MOUSE	Champ1	Chromosome alignment-maintaining phosphoprotein 1	0,0215712	-0,0321049
2333	SSH3_MOUSE	Ssh3	Protein phosphatase Slingshot homolog 3	0,248545	-0,14777
2334	I5P2_MOUSE	Inpp5b	Type II inositol 1,4,5-trisphosphate 5-phosphatase	1,11971	0,846643
2335	CBR3_MOUSE	Cbr3	Carbonyl reductase [NADPH] 3	0,492001	-1,3394

2336	ACD10_MOUSE	Acad10	Acyl-CoA dehydrogenase family member 10	0,356766	-0,23266
2337	DEN1A_MOUSE	Dennd1a	DENN domain-containing protein 1A	0,133449	-0,225493
2338	FRRS1_MOUSE	FRRS1	Ferric-chelate reductase 1	0,850984	-0,424688
2339	MEPCE_MOUSE	Mepce	7SK snRNA methylphosphate capping enzyme	0,543127	0,204292
2340	LZIC_MOUSE	Lzic	Protein LZIC	0,998495	0,482136
2341	VRK3_MOUSE	Vrk3	Inactive serine/threonine-protein kinase VRK3	0,583484	0,320437
2342	DP13B_MOUSE	Appl2	DCC-interacting protein 13-beta	0,177724	-0,301505
2343	DP13A_MOUSE	Appl1	DCC-interacting protein 13-alpha	0,630843	0,472668
2344	NDUS8_MOUSE	Ndufs8	NADH dehydrogenase [ubiquinone] iron-sulfur protein 8, mitochondrial	1,01475	0,415523
2345	OPTN_MOUSE	Optn	Optineurin	0,390897	0,52137
2346	CASC3_MOUSE	Casc3	Protein CASC3	0,526128	0,46553
2347	DPOLB_MOUSE	Polb	DNA polymerase beta	0,803856	0,565337
2348	PREP_MOUSE	Pitrm1	Presequence protease, mitochondrial	0,400341	0,275226
2349	ABC8B_MOUSE	Abca8b	ATP-binding cassette sub-family A member 8-B	1,51892	0,417524
2350	ABCA6_MOUSE	Abca6	ATP-binding cassette sub-family A member 6	0,766289	-0,208939
2351	MTA1_MOUSE	Mta1	Metastasis-associated protein MTA1	0,19125	0,10495
2352	ABHDB_MOUSE	Abhd11	Protein ABHD11	0,962561	0,448441
2353	LTBP4_MOUSE	Ltbp4	Latent-transforming growth factor beta-binding protein 4	0,0388302	0,0586136
2354	ABLM1_MOUSE	Ablim1	Actin-binding LIM protein 1	0,0513736	0,0477718
2355	KFA_MOUSE	Afmid	Kynurenine formamidase	0,326863	-0,160326
2356	PANK1_MOUSE	Pank1	Pantothenate kinase 1	0,978665	-0,351203
2357	DDX54_MOUSE	Ddx54	ATP-dependent RNA helicase DDX54	0,366027	0,370721
2358	SVIL_MOUSE	Svil	Supervillin	0,27875	0,198848
2359	COMD1_MOUSE	Commd1	COMM domain-containing protein 1	0,155486	0,082613
2360	WDR33_MOUSE	Wdr33	pre-mRNA 3' end processing protein WDR33	0,199187	-0,0811394
2361	RPTOR_MOUSE	Rptor	Regulatory-associated protein of mTOR	0,184043	-0,0721539
2362	COL12_MOUSE	Colec12	Collectin-12	0,79286	0,601409
2363	PITC1_MOUSE	Pitpnc1	Cytoplasmic phosphatidylinositol transfer protein 1	0,117311	-0,0602493
2364	NNRE_MOUSE	Naxe	NAD(P)H-hydrate epimerase	0,685884	-0,315753
2365	SF3A1_MOUSE	Sf3a1	Splicing factor 3A subunit 1	0,0634267	0,0566418
2366	DCAF8_MOUSE	Dcaf8	DDB1- and CUL4-associated factor 8	0,579702	0,49362
2367	EST2A_MOUSE	Ces2a	Pyrethroid hydrolase Ces2a	0,0483933	0,0446983
2368	ALAT1_MOUSE	Gpt	Alanine aminotransferase 1	0,229239	-0,179319
2369	HIBCH_MOUSE	Hibch	3-hydroxyisobutyryl-CoA hydrolase, mitochondrial	1,30809	0,367067
2370	THIL_MOUSE	Acat1	Acetyl-CoA acetyltransferase, mitochondrial	0,668648	-0,524521
2371	INT13_MOUSE	IntS13	Integrator complex subunit 13	0,920691	0,489683
2372	EIF3L_MOUSE	Eif3l	Eukaryotic translation initiation factor 3 subunit L	0,820155	-0,520021
2373	GLCTK_MOUSE	Glyctk	Glycerate kinase	0,907747	0,181079
2374	AIMP2_MOUSE	Aimp2	Aminoacyl tRNA synthase complex-interacting multifunctional protein 2	0,299241	0,117226
2375	BL1S5_MOUSE	Bloc1s5	Biogenesis of lysosome-related organelles complex 1 subunit 5	0,218184	0,159364
2376	BLMH_MOUSE	Blmh	Bleomycin hydrolase	1,02616	-0,612421
2377	ICT1_MOUSE	Mrpl58	Peptidyl-tRNA hydrolase ICT1, mitochondrial	0,791802	0,270863
2378	ERF3A_MOUSE	Gspt1	Eukaryotic peptide chain release factor GTP-binding subunit ERF3A	1,01827	0,481992
2379	GALE_MOUSE	Gale	UDP-glucose 4-epimerase	1,74382	0,551118
2380	HNRPL_MOUSE	Hnrnpl	Heterogeneous nuclear ribonucleoprotein L	0,776143	0,195216
2381	SUOX_MOUSE	Suox	Sulfite oxidase, mitochondrial	0,257199	-0,110085
2382	ILKAP_MOUSE	Ilkap	Integrin-linked kinase-associated serine/threonine phosphatase 2C	1,51039	0,518017
2383	FAHD1_MOUSE	Fahd1	Acylpyruvase FAHD1, mitochondrial	0,00806459	-0,00367355
2384	S14L4_MOUSE	Sec14l4	SEC14-like protein 4	0,00395677	0,00192528
2385	GGA1_MOUSE	Gga1	ADP-ribosylation factor-binding protein GGA1	1,27419	0,319613
2386	TM134_MOUSE	Tmem134	Transmembrane protein 134	0,554176	0,381869
2387	GNTK_MOUSE	Idnk	Probable gluconokinase	0,255023	0,108394

2388	HOT_MOUSE	Adhfe1	Hydroxyacid-oxoacid transhydrogenase, mitochondrial	0,0228307	-0,0220482
2389	AAMDC_MOUSE	Aamdc	Mth938 domain-containing protein	1,10635	-1,00142
2390	IQEC1_MOUSE	Iqsec1	IQ motif and SEC7 domain-containing protein 1	0,457113	0,309486
2391	I23O2_MOUSE	Ido2	Indoleamine 2,3-dioxygenase 2	0,71712	-0,456596
2392	EPIPL_MOUSE	Eppk1	Epiplakin	0,0594456	-0,0648064
2393	SGPL1_MOUSE	Sgpl1	Sphingosine-1-phosphate lyase 1	0,171112	-0,103637
2394	AL1L1_MOUSE	Aldh1l1	Cytosolic 10-formyltetrahydrofolate dehydrogenase	1,03181	-0,482765
2395	S2542_MOUSE	Slc25a42	Mitochondrial coenzyme A transporter SLC25A42	0,77439	-0,836637
2396	SIR3_MOUSE	Sirt3	NAD-dependent protein deacetylase sirtuin-3	0,56477	-0,416286
2397	VP37C_MOUSE	Vps37c	Vacuolar protein sorting-associated protein 37C	1,55406	1,30255
2398	ZPI_MOUSE	Serpina10	Protein Z-dependent protease inhibitor	0,974177	0,64309
2399	FAD1_MOUSE	Flad1	FAD synthase	0,19597	0,147488
2400	APEH_MOUSE	Apeh	Acylamino-acid-releasing enzyme	1,26705	0,321299
2401	ZNFX1_MOUSE	Znfx1	NFX1-type zinc finger-containing protein 1	0,528684	0,465396
2402	BPHL_MOUSE	Bphl	Valacyclovir hydrolase	0,425576	-0,209149
2403	ERO1A_MOUSE	Ero1a	ERO1-like protein alpha	1,23236	0,374232
2404	EIF3C_MOUSE	Eif3c	Eukaryotic translation initiation factor 3 subunit C	0,879977	0,262938
2405	NIBA2_MOUSE	Niban2	Protein Niban 2	0,130746	0,05975
2406	CMBL_MOUSE	Cmbl	Carboxymethylenebutenolidase homolog	0,777908	-0,289364
2407	HOP_MOUSE	Hopx	Homeodomain-only protein	0,0588385	-0,0545067
2408	AT2L2_MOUSE	Phykpl	5-phosphohydroxy-L-lysine phospho-lyase	0,0702877	-0,0977142
2409	DC1L1_MOUSE	Dync1li1	Cytoplasmic dynein 1 light intermediate chain 1	0,580419	0,612464
2410	RBSK_MOUSE	Rbks	Ribokinase	0,565761	-0,962407
2411	STAR7_MOUSE	Stard7	StAR-related lipid transfer protein 7, mitochondrial	0,8079	0,646783
2412	COQ6_MOUSE	Coq6	Ubiquinone biosynthesis monooxygenase COQ6, mitochondrial	0,820015	0,319381
2413	MTSS1_MOUSE	Mtss1	Protein MTSS 1	0,124529	-0,0606293
2414	CHMP7_MOUSE	Chmp7	Charged multivesicular body protein 7	0,0222739	0,0402981
2415	COG4_MOUSE	Cog4	Conserved oligomeric Golgi complex subunit 4	0,0381951	-0,0199543
2416	SPART_MOUSE	Spart	Spartin	0,208366	0,116639
2417	SDSL_MOUSE	Sdsl	Serine dehydratase-like	1,3925	0,425613
2418	FUCM_MOUSE	Fuom	Fucose mutarotase	0,753318	0,309055
2419	TDIF2_MOUSE	Dnttip2	Deoxynucleotidyltransferase terminal-interacting protein 2	0,372714	0,961446
2420	UTP4_MOUSE	Utp4	U3 small nucleolar RNA-associated protein 4 homolog	0,32733	0,307082
2421	KLH25_MOUSE	Klhl25	Kelch-like protein 25	0,672999	0,691129
2422	RRF2M_MOUSE	Gfm2	Ribosome-releasing factor 2, mitochondrial	0,250973	0,094125
2423	BST2_MOUSE	Bst2	Bone marrow stromal antigen 2	1,65107	0,551996
2424	TF3C5_MOUSE	Gtf3c5	General transcription factor 3C polypeptide 5	0,706041	0,335872
2425	SEH1_MOUSE	Seh1l	Nucleoporin SEH1	1,23409	0,324043
2426	ADAP2_MOUSE	Adap2	Arf-GAP with dual PH domain-containing protein 2	1,19171	1,18306
2427	ABHD6_MOUSE	Abhd6	Monoacylglycerol lipase ABHD6	0,816487	0,402427
2428	PTH2_MOUSE	Ptrh2	Peptidyl-tRNA hydrolase 2, mitochondrial	0,59508	0,387357
2429	VPS18_MOUSE	Vps18	Vacuolar protein sorting-associated protein 18 homolog	0,186276	0,236515
2430	UBQL1_MOUSE	Ubqln1	Ubiquilin-1	1,50225	0,651954
2431	RFC3_MOUSE	Rfc3	Replication factor C subunit 3	0,691983	0,162698
2432	PSPC1_MOUSE	Pspc1	Paraspeckle component 1	0,427309	-0,310673
2433	IGSF8_MOUSE	Igsf8	Immunoglobulin superfamily member 8	2,13504	1,19603
2434	MCMBP_MOUSE	Mcmbp	Mini-chromosome maintenance complex-binding protein	1,05447	0,510612
2435	TBC13_MOUSE	Tbc1d13	TBC1 domain family member 13	0,420815	0,564876
2436	FABD_MOUSE	Mcat	Malonyl-CoA-acyl carrier protein transacylase, mitochondrial	0,367421	0,131073
2437	STPAP_MOUSE	Tut1	Speckle targeted PIP5K1A-regulated poly(A) polymerase	1,04742	0,582838
2438	TCF25_MOUSE	Tcf25	Transcription factor 25	1,0012	0,403698
2439	THOC1_MOUSE	Thoc1	THO complex subunit 1	0,265724	-0,231859

2440	SARAF_MOUSE	Saraf	Store-operated calcium entry-associated regulatory factor	1,28415	1,02309
2441	CCD58_MOUSE	Ccdc58	Coiled-coil domain-containing protein 58	0,818132	0,490457
2442	EXOC1_MOUSE	Exoc1	Exocyst complex component 1	0,0510443	0,021423
2443	SHLB2_MOUSE	Sh3glb2	Endophilin-B2	0,769855	0,306007
2444	MIC13_MOUSE	Micos13	MICOS complex subunit MIC13	1,18672	-0,800729
2445	DICER_MOUSE	Dicer1	Endoribonuclease Dicer	0,0624754	0,0744442
2446	SL9A8_MOUSE	Slc9a8	Sodium/hydrogen exchanger 8	0,170339	0,384343
2447	ARHGC_MOUSE	Arhgef12	Rho guanine nucleotide exchange factor 12	0,746985	0,217188
2448	NAGS_MOUSE	Nags	N-acetylglutamate synthase, mitochondrial	1,02268	-0,620471
2449	IRAK4_MOUSE	Irak4	Interleukin-1 receptor-associated kinase 4	1,01835	0,557961
2450	CLYBL_MOUSE	Clybl	Citramalyl-CoA lyase, mitochondrial	0,81047	0,245842
2451	STAB2_MOUSE	Stab2	Stabilin-2	0,938426	-0,344761
2452	LUZP1_MOUSE	Luzp1	Leucine zipper protein 1	0,383608	0,266946
2453	RBM12_MOUSE	Rbm12	RNA-binding protein 12	0,517824	0,23719
2454	STAB1_MOUSE	Stab1	Stabilin-1	1,33962	0,436449
2455	ACMSD_MOUSE	Acmsd	2-amino-3-carboxymuconate-6-semialdehyde decarboxylase	0,924231	-1,27917
2456	SNP47_MOUSE	Snap47	Synaptosomal-associated protein 47	0,891165	0,51649
2457	KPRB_MOUSE	Prpsap2	Phosphoribosyl pyrophosphate synthase-associated protein 2	0,714729	-0,947857
2458	TB22A_MOUSE	Tbc1d22a	TBC1 domain family member 22A	0,912085	0,543238
2459	IFIH1_MOUSE	Ifih1	Interferon-induced helicase C domain-containing protein 1	0,860085	0,380124
2460	ARAP3_MOUSE	Arap3	Arf-GAP with Rho-GAP domain, ANK repeat and PH domain-containing prot	0,0823026	0,0485687
2461	UBP15_MOUSE	Usp15	Ubiquitin carboxyl-terminal hydrolase 15	0,281143	0,128508
2462	VPS39_MOUSE	Vps39	Vam6/Vps39-like protein	0,153199	-0,103736
2463	SDHL_MOUSE	Sds	L-serine dehydratase/L-threonine deaminase	2,08876	-0,691093
2464	ASPC1_MOUSE	Aspscr1	Tether containing UBX domain for GLUT4	0,147028	0,0721699
2465	EXOS2_MOUSE	Exosc2	Exosome complex component RRP4	0,166047	0,0828339
2466	CSN8_MOUSE	Cops8	COP9 signalosome complex subunit 8	1,70871	0,369133
2467	ULA1_MOUSE	Nae1	NEDD8-activating enzyme E1 regulatory subunit	1,03788	0,23172
2468	TTC36_MOUSE	Ttc36	Tetratricopeptide repeat protein 36	0,684489	-0,297829
2469	MPDZ_MOUSE	Mpdz	Multiple PDZ domain protein	0,494386	-0,157659
2470	CLPT1_MOUSE	Clptm1	Cleft lip and palate transmembrane protein 1 homolog	0,765647	1,19712
2471	EMAL3_MOUSE	Eml3	Echinoderm microtubule-associated protein-like 3	1,08521	0,479685
2472	HUTU_MOUSE	Uroc1	Urocanate hydratase	1,01211	-0,434105
2473	HEM1_MOUSE	Alas1	5-aminolevulinate synthase, nonspecific, mitochondrial	0,126787	-0,218998
2474	AK1CD_MOUSE	Akr1c13	Aldo-keto reductase family 1 member C13	1,29395	0,93795
2475	TKFC_MOUSE	Tkfc	Triokinase/FMN cyclase	1,1622	0,49165
2476	RBPS2_MOUSE	Rbpms2	RNA-binding protein with multiple splicing 2	0,293595	0,184301
2477	LSM1_MOUSE	Lsm1	U6 snRNA-associated Sm-like protein LSm1	0,377793	0,238498
2478	BUP1_MOUSE	Upb1	Beta-ureidopropionase	0,420833	-0,30618
2479	SCRN2_MOUSE	Scrn2	Secernin-2	0,427597	-0,246593
2480	GYS2_MOUSE	Gys2	Glycogen [starch] synthase, liver	0,921689	0,564425
2481	PGDH_MOUSE	Hpgd	15-hydroxyprostaglandin dehydrogenase [NAD(+)]	0,483111	0,504661
2482	EST1_MOUSE	Ces1	Liver carboxylesterase 1	0,610848	0,669921
2483	MAVS_MOUSE	Mavs	Mitochondrial antiviral-signaling protein	1,35048	0,51767
2484	CO8G_MOUSE	C8g	Complement component C8 gamma chain	0,956701	0,521858
2485	THIKB_MOUSE	Acaa1b	3-ketoacyl-CoA thiolase B, peroxisomal	0,529039	0,43096
2486	DHC24_MOUSE	Dhcr24	Delta(24)-sterol reductase	0,310444	-0,275337
2487	RDH10_MOUSE	Rdh10	Retinol dehydrogenase 10	0,380613	0,431774
2488	UBXN4_MOUSE	Ubxn4	UBX domain-containing protein 4	1,62776	0,514595
2489	PLBL1_MOUSE	Plbd1	Phospholipase B-like 1	0,806651	1,80227
2490	PEX19_MOUSE	Pex19	Peroxisomal biogenesis factor 19	0,0609609	0,0430687
2491	SCO2_MOUSE	Sco2	Protein SCO2 homolog, mitochondrial	0,741406	0,424934

2492	ZFY21_MOUSE	Zfyve21	Zinc finger FYVE domain-containing protein 21	1,73567	0,576432
2493	FIBG_MOUSE	Fgg	Fibrinogen gamma chain	1,27339	0,468082
2494	NCLN_MOUSE	Ncln	Nicalin	0,0805635	0,0587383
2495	CGL_MOUSE	Cth	Cystathionine gamma-lyase	1,14251	-0,365092
2496	TBCC_MOUSE	Tbcc	Tubulin-specific chaperone C	0,795435	0,413832
2497	DHB13_MOUSE	Hsd17b13	17-beta-hydroxysteroid dehydrogenase 13	0,0277495	0,022411
2498	ABHEB_MOUSE	Abhd14b	Protein ABHD14B	0,525322	-0,366351
2499	AMPB_MOUSE	Rnpep	Aminopeptidase B	0,17606	0,116216
2500	CES1D_MOUSE	Ces1d	Carboxylesterase 1D	2,27391	0,636515
2501	EST3B_MOUSE	Ces3b	Carboxylesterase 3B	0,364491	0,339634
2502	ACSF2_MOUSE	Acsf2	Medium-chain acyl-CoA ligase ACSF2, mitochondrial	0,905998	0,398207
2503	AK1D1_MOUSE	Akr1d1	Aldo-keto reductase family 1 member D1	0,946541	0,518213
2504	MICU1_MOUSE	Micu1	Calcium uptake protein 1, mitochondrial	0,674603	-0,266479
2505	HYPDH_MOUSE	Prodh2	Hydroxyproline dehydrogenase	0,0484131	0,0352242
2506	GRAP1_MOUSE	Gripap1	GRIP1-associated protein 1	0,616599	0,632663
2507	TPSNR_MOUSE	Tapbpl	Tapasin-related protein	1,45924	0,896941
2508	PI3R4_MOUSE	Pik3r4	Phosphoinositide 3-kinase regulatory subunit 4	0,177782	-0,145318
2509	HIP1_MOUSE	Hip1	Huntingtin-interacting protein 1	0,380072	0,437148
2510	SYLM_MOUSE	Lars2	Probable leucine--tRNA ligase, mitochondrial	0,952566	0,310462
2511	FYCO1_MOUSE	Fyco1	FYVE and coiled-coil domain-containing protein 1	0,490472	0,244593
2512	MYH9_MOUSE	Myh9	Myosin-9	1,12054	0,246971
2513	WASH1_MOUSE	Washc1	WASH complex subunit 1	0,108992	0,228916
2514	PARN_MOUSE	Parn	Poly(A)-specific ribonuclease PARN	0,441088	0,339016
2515	PPCS_MOUSE	Ppcs	Phosphopantothenate--cysteine ligase	0,628703	0,203228
2516	PAFA2_MOUSE	Pafah2	Platelet-activating factor acetylhydrolase 2, cytoplasmic	0,101338	-0,108379
2517	UBAC1_MOUSE	Ubac1	Ubiquitin-associated domain-containing protein 1	0,57082	0,668
2518	VIGLN_MOUSE	Hdlbp	Vigilin	1,24347	0,367297
2519	NIT1_MOUSE	Nit1	Deaminated glutathione amidase	0,686064	0,348441
2520	ADPGK_MOUSE	Adpgk	ADP-dependent glucokinase	0,096241	-0,0627144
2521	PSMD2_MOUSE	Psmd2	26S proteasome non-ATPase regulatory subunit 2	0,754386	0,533445
2522	HNRL1_MOUSE	Hnrnpul1	Heterogeneous nuclear ribonucleoprotein U-like protein 1	0,674439	0,260379
2523	AT1A1_MOUSE	Atp1a1	Sodium/potassium-transporting ATPase subunit alpha-1	0,895865	0,501267
2524	CCAR2_MOUSE	Ccar2	Cell cycle and apoptosis regulator protein 2	0,0467103	-0,0150539
2525	PTGR2_MOUSE	Ptgr2	Prostaglandin reductase 2	0,177654	0,174299
2526	SIR2_MOUSE	Sirt2	NAD-dependent protein deacetylase sirtuin-2	0,875344	0,402098
2527	DX39A_MOUSE	Ddx39a	ATP-dependent RNA helicase DDX39A	0,129727	-0,0815323
2528	ZDHC5_MOUSE	Zdhhc5	Palmitoyltransferase ZDHHC5	0,741998	-0,533876
2529	TT39C_MOUSE	Ttc39c	Tetratricopeptide repeat protein 39C	1,0506	-0,706739
2530	MTMR6_MOUSE	Mtmr6	Myotubularin-related protein 6	0,526951	0,428562
2531	MIO_MOUSE	Mios	GATOR complex protein MIOS	0,776285	0,799179
2532	RT23_MOUSE	Mrps23	28S ribosomal protein S23, mitochondrial	0,472206	-0,157175
2533	OXND1_MOUSE	Oxnad1	Oxidoreductase NAD-binding domain-containing protein 1	0,482323	0,260027
2534	UBA5_MOUSE	Uba5	Ubiquitin-like modifier-activating enzyme 5	0,0496988	-0,0350403
2535	PDC10_MOUSE	Pdcd10	Programmed cell death protein 10	1,07827	0,365322
2536	THOC3_MOUSE	Thoc3	THO complex subunit 3	1,06578	-1,12698
2537	F1142_MOUSE	Fam114a2	Protein FAM114A2	0,308369	-0,25502
2538	CH082_MOUSE		UPF0598 protein C8orf82 homolog	1,01192	0,520398
2539	SRSF4_MOUSE	Srsf4	Serine/arginine-rich splicing factor 4	0,00023647	0,000141907
2540	CC115_MOUSE	Ccdc115	Coiled-coil domain-containing protein 115	1,40212	0,814216
2541	RPE_MOUSE	Rpe	Ribulose-phosphate 3-epimerase	1,29091	0,466713
2542	RFA1_MOUSE	Rpa1	Replication protein A 70 kDa DNA-binding subunit	0,263661	0,0773335
2543	EPMIP_MOUSE	Epm2aip1	EPM2A-interacting protein 1	0,933305	0,283509

2544	ERLEC_MOUSE	Erlec1	Endoplasmic reticulum lectin 1	0,904024	0,518328
2545	VPS4A_MOUSE	Vps4a	Vacuolar protein sorting-associated protein 4A	1,25976	1,5627
2546	CC50A_MOUSE	Tmem30a	Cell cycle control protein 50A	0,287553	0,405759
2547	HNRPU_MOUSE	Hnrnpu	Heterogeneous nuclear ribonucleoprotein U	0,126052	-0,0417854
2548	MPCP_MOUSE	Slc25a3	Phosphate carrier protein, mitochondrial	0,158218	-0,0722668
2549	RBM39_MOUSE	Rbm39	RNA-binding protein 39	0,293606	0,133378
2550	SEC63_MOUSE	Sec63	Translocation protein SEC63 homolog	0,219226	0,210364
2551	FMO4_MOUSE	Fmo4	Dimethylaniline monooxygenase [N-oxide-forming] 4	0,17436	-0,0780811
2552	CSKI2_MOUSE	Caskin2	Caskin-2	0,978538	0,437104
2553	DHX36_MOUSE	Dhx36	ATP-dependent DNA/RNA helicase DHX36	0,577367	0,234082
2554	P66B_MOUSE	Gatad2b	Transcriptional repressor p66-beta	0,622735	0,552022
2555	IMP4_MOUSE	Imp4	U3 small nucleolar ribonucleoprotein protein IMP4	0,84852	0,282764
2556	TAF9_MOUSE	Taf9	Transcription initiation factor TFIID subunit 9	0,543549	0,184852
2557	PAXI_MOUSE	Pxn	Paxillin	0,0529859	0,112864
2558	MRP2_MOUSE	Abcc2	Canalicular multispecific organic anion transporter 1	0,839976	-0,3065
2559	MOB2_MOUSE	Mob2	MOB kinase activator 2	0,854607	-0,553864
2560	IPO4_MOUSE	Ipo4	Importin-4	0,150086	-0,18523
2561	SFPQ_MOUSE	Sfpq	Splicing factor, proline- and glutamine-rich	0,262263	0,158797
2562	IRGQ_MOUSE	Irgq	Immunity-related GTPase family Q protein	1,32128	0,75084
2563	CS012_MOUSE		Protein C19orf12 homolog	1,60736	0,760803
2564	WDR13_MOUSE	Wdr13	WD repeat-containing protein 13	1,1339	0,289058
2565	BACH_MOUSE	Acot7	Cytosolic acyl coenzyme A thioester hydrolase	0,543562	0,218751
2566	ETFR1_MOUSE	Etfrf1	Electron transfer flavoprotein regulatory factor 1	0,0429006	0,0139027
2567	RAB14_MOUSE	Rab14	Ras-related protein Rab-14	0,330277	-0,262545
2568	ISOC1_MOUSE	Isoc1	Isochorismatase domain-containing protein 1	0,52178	-0,31817
2569	CK054_MOUSE		Ester hydrolase C11orf54 homolog	0,484528	0,226688
2570	ACLY_MOUSE	Acly	ATP-citrate synthase	0,26655	0,385088
2571	ACSM1_MOUSE	Acsm1	Acyl-coenzyme A synthetase ACSM1, mitochondrial	0,456292	0,335358
2572	PDIP2_MOUSE	Poldip2	Polymerase delta-interacting protein 2	1,21506	0,326056
2573	IF4A3_MOUSE	Eif4a3	Eukaryotic initiation factor 4A-III	0,137374	-0,148732
2574	PLVAP_MOUSE	Plvap	Plasmalemma vesicle-associated protein	0,857903	0,331691
2575	NDUS1_MOUSE	Ndufs1	NADH-ubiquinone oxidoreductase 75 kDa subunit, mitochondrial	0,407328	-0,175261
2576	S27A4_MOUSE	Slc27a4	Long-chain fatty acid transport protein 4	1,47565	0,464725
2577	HNMT_MOUSE	Hnmt	Histamine N-methyltransferase	0,844925	-0,333252
2578	SNX9_MOUSE	Snx9	Sorting nexin-9	0,0741329	0,0662716
2579	MEMO1_MOUSE	Memo1	Protein MEMO1	0,102492	0,0331066
2580	RINI_MOUSE	Rnh1	Ribonuclease inhibitor	0,659046	0,276203
2581	STK38_MOUSE	Stk38	Serine/threonine-protein kinase 38	0,373453	0,241252
2582	RMXL1_MOUSE	Rbmxl1	RNA binding motif protein, X-linked-like-1	0,741868	0,214381
2583	IPYR2_MOUSE	Ppa2	Inorganic pyrophosphatase 2, mitochondrial	0,135752	-0,0875854
2584	MIC25_MOUSE	Chchd6	MICOS complex subunit Mic25	1,97094	1,33476
2585	DDX41_MOUSE	Ddx41	Probable ATP-dependent RNA helicase DDX41	0,747176	0,317782
2586	ATPG_MOUSE	Atp5f1c	ATP synthase subunit gamma, mitochondrial	0,273575	-0,2804
2587	DDX1_MOUSE	Ddx1	ATP-dependent RNA helicase DDX1	0,688906	0,305557
2588	BRK1_MOUSE	Brk1	Protein BRICK1	0,456254	0,344436
2589	MGST1_MOUSE	Mgst1	Microsomal glutathione S-transferase 1	0,961835	-0,916946
2590	FARP2_MOUSE	Farp2	FERM, ARHGEF and pleckstrin domain-containing protein 2	0,45115	-0,271994
2591	CBR4_MOUSE	Cbr4	Carbonyl reductase family member 4	0,0558616	-0,0300091
2592	DCA11_MOUSE	Dcaf11	DDB1- and CUL4-associated factor 11	0,0580171	0,0449398
2593	PUS7_MOUSE	Pus7	Pseudouridylate synthase 7 homolog	0,43919	0,39599
2594	SH3L3_MOUSE	Sh3bgrl3	SH3 domain-binding glutamic acid-rich-like protein 3	1,62944	0,596616
2595	GOGA4_MOUSE	Golga4	Golgin subfamily A member 4	0,718681	0,627876

2596	UBAP2_MOUSE	Ubap2	Ubiquitin-associated protein 2	1,55966	0,682561
2597	NCOA5_MOUSE	Ncoa5	Nuclear receptor coactivator 5	0,200341	0,172465
2598	GCSP_MOUSE	Gldc	Glycine dehydrogenase (decarboxylating), mitochondrial	0,212467	0,110819
2599	CSDE1_MOUSE	Csde1	Cold shock domain-containing protein E1	0,788361	0,327848
2600	RBMS1_MOUSE	Rbms1	RNA-binding motif, single-stranded-interacting protein 1	0,318813	-0,175108
2601	FXL15_MOUSE	Fbxl15	F-box/LRR-repeat protein 15	1,78859	1,01294
2602	CP270_MOUSE	Cyp2c70	Cytochrome P450 2C70	0,548954	-0,516256
2603	UBL7_MOUSE	Ubl7	Ubiquitin-like protein 7	1,94743	1,40352
2604	VPS11_MOUSE	Vps11	Vacuolar protein sorting-associated protein 11 homolog	1,11655	0,431472
2605	MA2C1_MOUSE	Man2c1	Alpha-mannosidase 2C1	0,272137	-0,0880699
2606	TXND5_MOUSE	Txndc5	Thioredoxin domain-containing protein 5	0,379457	0,21848
2607	SETD3_MOUSE	Setd3	Actin-histidine N-methyltransferase	0,0357002	-0,0340481
2608	NDUS2_MOUSE	Ndufs2	NADH dehydrogenase [ubiquinone] iron-sulfur protein 2, mitochondrial	0,511105	-0,324656
2609	SNX15_MOUSE	Snx15	Sorting nexin-15	0,139654	0,0886837
2610	EST2C_MOUSE	Ces2c	Acylcarnitine hydrolase	0,981203	0,454869
2611	RABE2_MOUSE	Rabep2	Rab GTPase-binding effector protein 2	1,12216	0,518084
2612	ELP2_MOUSE	Elp2	Elongator complex protein 2	0,519628	0,323453
2613	GLCNE_MOUSE	Gne	Bifunctional UDP-N-acetylglucosamine 2-epimerase/N-acetylmannosamine	0,264594	-0,129296
2614	SPS2L_MOUSE	Spats2l	SPATS2-like protein	0,867443	0,517479
2615	FUBP1_MOUSE	Fubp1	Far upstream element-binding protein 1	1,28569	0,619849
2616	SPRY4_MOUSE	Spryd4	SPRY domain-containing protein 4	0,766403	-0,278342
2617	EIF3H_MOUSE	Eif3h	Eukaryotic translation initiation factor 3 subunit H	0,277761	-0,173653
2618	GCSH_MOUSE	Gcsh	Glycine cleavage system H protein, mitochondrial	0,526129	-1,30287
2619	WWOX_MOUSE	Wwox	WW domain-containing oxidoreductase	0,906075	1,3791
2620	STRBP_MOUSE	Strbp	Spermatid perinuclear RNA-binding protein	1,45295	1,88639
2621	HDHD5_MOUSE	Hdhd5	Haloacid dehalogenase-like hydrolase domain-containing 5	0,0606253	-0,0278984
2622	KMO_MOUSE	Kmo	Kynurenine 3-monooxygenase	0,0208302	-0,00903587
2623	MASP2_MOUSE	Masp2	Mannan-binding lectin serine protease 2	0,0809048	0,0841473
2624	SPA3N_MOUSE	Serpina3n	Serine protease inhibitor A3N	0,033859	-0,0252548
2625	SYYC_MOUSE	Yars	Tyrosine--tRNA ligase, cytoplasmic	1,21441	0,304842
2626	ASCC2_MOUSE	Ascc2	Activating signal cointegrator 1 complex subunit 2	0,384403	-0,485122
2627	CISD1_MOUSE	Cisd1	CDGSH iron-sulfur domain-containing protein 1	1,41649	-0,4321
2628	BHMT2_MOUSE	Bhmt2	S-methylmethionine--homocysteine S-methyltransferase BHMT2	0,969247	-1,90786
2629	RBM47_MOUSE	Rbm47	RNA-binding protein 47	0,4859	-0,314677
2630	CBS_MOUSE	Cbs	Cystathionine beta-synthase	0,851602	0,278531
2631	CES1F_MOUSE	Ces1f	Carboxylesterase 1F	1,50471	1,97619
2632	AS3MT_MOUSE	As3mt	Arsenite methyltransferase	0,19653	0,0727322
2633	NC2B_MOUSE	Dr1	Protein Dr1	0,646323	1,01274
2634	DTBP1_MOUSE	Dtnbp1	Dysbindin	2,28742	1,4447
2635	BAAT_MOUSE	Baat	Bile acid-CoA:amino acid N-acyltransferase	0,840781	0,443671
2636	GCKR_MOUSE	Gckr	Glucokinase regulatory protein	0,280469	-0,112549
2637	DCXR_MOUSE	Dcxr	L-xylulose reductase	0,0258191	0,0150993
2638	ZFN2B_MOUSE	Zfand2b	AN1-type zinc finger protein 2B	0,0714848	0,117928
2639	HEMO_MOUSE	Hpx	Hemopexin	0,0110715	0,0130058
2640	CY250_MOUSE	Cyp2c50	Cytochrome P450 2C50	0,589744	-1,09634
2641	ERLN1_MOUSE	Erlin1	Erlin-1	0,0708215	-0,0632065
2642	METK1_MOUSE	Mat1a	S-adenosylmethionine synthase isoform type-1	0,744329	-0,291088
2643	NADC_MOUSE	Qprt	Nicotinate-nucleotide pyrophosphorylase [carboxylating]	0,169835	-0,371723
2644	DAP1_MOUSE	Dap	Death-associated protein 1	0,0974345	-0,0424065
2645	PEX16_MOUSE	Pex16	Peroxisomal membrane protein PEX16	0,367369	0,19581
2646	FTCD_MOUSE	Ftcd	Formimidoyltransferase-cyclodeaminase	0,0664522	-0,0251957
2647	VPS36_MOUSE	Vps36	Vacuolar protein-sorting-associated protein 36	0,534074	0,325278

2648	CREL1_MOUSE	Creld1	Cysteine-rich with EGF-like domain protein 1	0,673148	0,279463
2649	GLYAT_MOUSE	Glyat	Glycine N-acyltransferase	1,06184	0,550508
2650	ACY3_MOUSE	Acy3	N-acyl-aromatic-L-amino acid amidohydrolase (carboxylate-forming)	0,112419	0,101366
2651	PNPO_MOUSE	Pnpo	Pyridoxine-5'-phosphate oxidase	0,763185	0,220422
2652	DUS3L_MOUSE	Dus3l	tRNA-dihydrouridine(47) synthase [NAD(P)(+)]-like	1,10045	1,43971
2653	OSBL1_MOUSE	Osbpl1a	Oxysterol-binding protein-related protein 1	0,181717	-0,145142
2654	SC16B_MOUSE	Sec16b	Protein transport protein Sec16B	0,0200761	0,0132717
2655	WRIP1_MOUSE	Wrnip1	ATPase WRNIP1	0,617117	0,464342
2656	FA11_MOUSE	F11	Coagulation factor XI	0,454817	0,325359
2657	MK08_MOUSE	Mapk8	Mitogen-activated protein kinase 8	0,185897	0,13838
2658	ALDOB_MOUSE	Aldob	Fructose-bisphosphate aldolase B	0,951841	-0,388822
2659	WASL_MOUSE	Wasl	Neural Wiskott-Aldrich syndrome protein	0,0463136	-0,0285915
2660	EGLN1_MOUSE	Egln1	Egl nine homolog 1	0,602943	0,571912
2661	IPO9_MOUSE	Ipo9	Importin-9	0,507038	-0,374035
2662	RBM5_MOUSE	Rbm5	RNA-binding protein 5	1,06697	0,796603
2663	ATLA3_MOUSE	Atl3	Atlastin-3	0,044932	-0,0337868
2664	ARLY_MOUSE	Asl	Argininosuccinate lyase	1,0633	-0,314177
2665	SNX4_MOUSE	Snx4	Sorting nexin-4	1,43261	0,343432
2666	THYN1_MOUSE	Thyn1	Thymocyte nuclear protein 1	0,0437815	0,0206627
2667	RHG35_MOUSE	Arhgap35	Rho GTPase-activating protein 35	0,923123	0,419786
2668	FAKD4_MOUSE	Tbrg4	FAST kinase domain-containing protein 4	0,467686	-0,153783
2669	UAP1_MOUSE	Uap1	UDP-N-acetylhexosamine pyrophosphorylase	0,300804	-0,120035
2670	L2HDH_MOUSE	L2hgdh	L-2-hydroxyglutarate dehydrogenase, mitochondrial	0,454014	0,286063
2671	NEUL_MOUSE	Nln	Neurolysin, mitochondrial	1,11563	0,347222
2672	DEOC_MOUSE	Dera	Deoxyribose-phosphate aldolase	0,276793	-0,118222
2673	RPN1_MOUSE	Rpn1	Dolichyl-diphosphooligosaccharide--protein glycosyltransferase subunit 1	0,533271	-0,298631
2674	TWF1_MOUSE	Twf1	Twinfilin-1	1,28945	0,427697
2675	EFNMT_MOUSE	EEF1AKNMT	eEF1A lysine and N-terminal methyltransferase	0,206001	0,132417
2676	PRP6_MOUSE	Prpf6	Pre-mRNA-processing factor 6	0,745807	0,746503
2677	PTGR1_MOUSE	Ptgr1	Prostaglandin reductase 1	0,66561	-0,327061
2678	NDUV1_MOUSE	Ndufv1	NADH dehydrogenase [ubiquinone] flavoprotein 1, mitochondrial	0,14412	0,11801
2679	SSF1_MOUSE	Ppan	Suppressor of SWI4 1 homolog	1,22048	0,614595
2680	DNJC3_MOUSE	Dnajc3	DnaJ homolog subfamily C member 3	1,31523	0,423448
2681	LGAT1_MOUSE	Lpgat1	Acyl-CoA:lysophosphatidylglycerol acyltransferase 1	0,426573	0,321289
2682	PTBP2_MOUSE	Ptbp2	Polypyrimidine tract-binding protein 2	0,752108	-0,547913
2683	TTC1_MOUSE	Ttc1	Tetratricopeptide repeat protein 1	0,931711	0,587255
2684	GRHPR_MOUSE	Grhpr	Glyoxylate reductase/hydroxypyruvate reductase	0,210627	0,133287
2685	CF132_MOUSE		Uncharacterized protein C6orf132 homolog	0,0334987	0,0386322
2686	SRGP2_MOUSE	Srgap2	SLIT-ROBO Rho GTPase-activating protein 2	0,00437576	-0,00261688
2687	B3GT6_MOUSE	B3galt6	Beta-1,3-galactosyltransferase 6	1,70767	0,963772
2688	PCCA_MOUSE	Pcca	Propionyl-CoA carboxylase alpha chain, mitochondrial	1,13429	-0,549999
2689	TMLH_MOUSE	Tmlhe	Trimethyllysine dioxygenase, mitochondrial	1,30501	-0,486898
2690	UGPA_MOUSE	Ugp2	UTP--glucose-1-phosphate uridylyltransferase	1,09388	-0,316174
2691	LPIN1_MOUSE	Lpin1	Phosphatidate phosphatase LPIN1	2,0214	-1,7367
2692	SNX18_MOUSE	Snx18	Sorting nexin-18	0,270766	0,212561
2693	FGD4_MOUSE	Fgd4	FYVE, RhoGEF and PH domain-containing protein 4	0,312747	0,264122
2694	MIA2_MOUSE	Mia2	Melanoma inhibitory activity protein 2	0,0344065	0,0221672
2695	SMCA5_MOUSE	Smarca5	SWI/SNF-related matrix-associated actin-dependent regulator of chromatin	0,65801	0,257757
2696	LRP1_MOUSE	Lrp1	Prolow-density lipoprotein receptor-related protein 1	0,304221	0,110423
2697	RISC_MOUSE	Scpep1	Retinoid-inducible serine carboxypeptidase	0,925911	-0,507405
2698	AFG31_MOUSE	Afg3l1	AFG3-like protein 1	0,192329	-0,132418
2699	SP16H_MOUSE	Supt16h	FACT complex subunit SPT16	0,24747	0,0566978

2700	FPPS_MOUSE	Fdps	Farnesyl pyrophosphate synthase	0,430086	-0,689444
2701	FADS1_MOUSE	Fads1	Acyl-CoA (8-3)-desaturase	1,53012	0,733422
2702	VPS16_MOUSE	Vps16	Vacuolar protein sorting-associated protein 16 homolog	0,376702	0,255754
2703	NS1BP_MOUSE	Ivns1abp	Influenza virus NS1A-binding protein homolog	0,0755923	-0,048999
2704	ALS2_MOUSE	Als2	Alsin	0,701031	0,496439
2705	RAB31_MOUSE	Rab31	Ras-related protein Rab-31	0,548745	-0,632719
2706	TADBP_MOUSE	Tardbp	TAR DNA-binding protein 43	0,527334	0,360643
2707	HNRLL_MOUSE	Hnrnpll	Heterogeneous nuclear ribonucleoprotein L-like	0,221164	0,0694553
2708	ETFD_MOUSE	Etfdh	Electron transfer flavoprotein-ubiquinone oxidoreductase, mitochondrial	0,162197	0,0665565
2709	THIKA_MOUSE	Acaa1a	3-ketoacyl-CoA thiolase A, peroxisomal	1,29374	0,975055
2710	COA7_MOUSE	Coa7	Cytochrome c oxidase assembly factor 7	0,162203	-0,130087
2711	TRFE_MOUSE	Tf	Serotransferrin	0,362954	0,268967
2712	EXOS4_MOUSE	Exosc4	Exosome complex component RRP41	0,797475	1,12846
2713	RHEB_MOUSE	Rheb	GTP-binding protein Rheb	1,82337	0,443243
2714	BCL7B_MOUSE	Bcl7b	B-cell CLL/lymphoma 7 protein family member B	0,947983	0,64496
2715	COG2_MOUSE	Cog2	Conserved oligomeric Golgi complex subunit 2	0,440944	0,150842
2716	SF3B3_MOUSE	Sf3b3	Splicing factor 3B subunit 3	0,45701	-0,184316
2717	GOGA2_MOUSE	Golga2	Golgin subfamily A member 2	2,02554	0,692187
2718	FA49B_MOUSE	Fam49b	Protein FAM49B	0,318982	0,119181
2719	DDX27_MOUSE	Ddx27	Probable ATP-dependent RNA helicase DDX27	0,759382	0,233138
2720	RM37_MOUSE	Mrpl37	39S ribosomal protein L37, mitochondrial	0,910299	-0,504908
2721	TOIP1_MOUSE	Tor1aip1	Torsin-1A-interacting protein 1	0,744043	0,856506
2722	SMTN_MOUSE	Smtn	Smoothelin	0,119627	-0,149842
2723	CHM1A_MOUSE	Chmp1a	Charged multivesicular body protein 1a	0,916368	0,402575
2724	QORL1_MOUSE	Cryzl1	Quinone oxidoreductase-like protein 1	0,717427	0,289792
2725	PDIA5_MOUSE	Pdia5	Protein disulfide-isomerase A5	0,435256	-0,216866
2726	MACD1_MOUSE	Macrod1	ADP-ribose glycohydrolase MACROD1	0,599495	-0,382129
2727	SYDC_MOUSE	Dars	Aspartate--tRNA ligase, cytoplasmic	1,82981	0,390141
2728	ITPI2_MOUSE	Itprid2	Protein ITPRID2	0,105447	0,0951748
2729	PP6R3_MOUSE	Ppp6r3	Serine/threonine-protein phosphatase 6 regulatory subunit 3	0,224183	-0,210066
2730	C1TC_MOUSE	Mthfd1	C-1-tetrahydrofolate synthase, cytoplasmic	0,0803873	0,0358135
2731	PCY2_MOUSE	Pcyt2	Ethanolamine-phosphate cytidylyltransferase	0,673134	0,422957
2732	TBB6_MOUSE	Tubb6	Tubulin beta-6 chain	0,0963306	-0,0306671
2733	GMPPA_MOUSE	Gmppa	Mannose-1-phosphate guanyltransferase alpha	0,482351	0,36182
2734	ZN330_MOUSE	Znf330	Zinc finger protein 330	0,0209427	0,0148163
2735	CLIP1_MOUSE	Clip1	CAP-Gly domain-containing linker protein 1	1,20874	0,293873
2736	NOP2_MOUSE	Nop2	Probable 28S rRNA (cytosine-C(5))-methyltransferase	0,656427	-0,214596
2737	GLYR1_MOUSE	Glyr1	Putative oxidoreductase GLYR1	0,133343	0,0780968
2738	MARC2_MOUSE	Marc2	Mitochondrial amidoxime reducing component 2	0,926176	0,294851
2739	LRC59_MOUSE	Lrrc59	Leucine-rich repeat-containing protein 59	0,002577	-0,000540161
2740	CHID1_MOUSE	Chid1	Chitinase domain-containing protein 1	0,896518	0,436899
2741	PDIA6_MOUSE	Pdia6	Protein disulfide-isomerase A6	1,66436	0,503585
2742	PDE2A_MOUSE	Pde2a	cGMP-dependent 3',5'-cyclic phosphodiesterase	0,7165	-0,546132
2743	PRPF3_MOUSE	Prpf3	U4/U6 small nuclear ribonucleoprotein Prp3	0,374388	0,190036
2744	PLRG1_MOUSE	Plrg1	Pleiotropic regulator 1	0,802145	0,203293
2745	UBXN1_MOUSE	Ubxn1	UBX domain-containing protein 1	0,544491	0,42606
2746	GGACT_MOUSE	Ggact	Gamma-glutamylaminecyclotransferase	1,23286	-0,662317
2747	DBR1_MOUSE	Dbr1	Lariat debranching enzyme	0,356106	-0,301723
2748	STEA4_MOUSE	Steap4	Metalloreductase STEAP4	0,771491	-0,486785
2749	BLVRB_MOUSE	Blvrb	Flavin reductase (NADPH)	0,535069	0,354353
2750	WBP11_MOUSE	Wbp11	WW domain-binding protein 11	0,0151022	0,0100079
2751	RPAB3_MOUSE	Polr2h	DNA-directed RNA polymerases I, II, and III subunit RPABC3	0,0472045	0,0255997

2752	STA13_MOUSE	Stard13	StAR-related lipid transfer protein 13	1,74023	0,224737
2753	TM1L1_MOUSE	Tom1l1	TOM1-like protein 1	0,455004	0,232324
2754	RT4I1_MOUSE	Rtn4ip1	Reticulon-4-interacting protein 1, mitochondrial	0,110569	-0,0498055
2755	LTMD1_MOUSE	Letmd1	LETM1 domain-containing protein 1	0,101498	-0,104314
2756	BODG_MOUSE	Bbox1	Gamma-butyrobetaine dioxygenase	0,314342	0,114527
2757	ESAM_MOUSE	Esam	Endothelial cell-selective adhesion molecule	0,154377	0,169147
2758	ATAD3_MOUSE	Atad3	ATPase family AAA domain-containing protein 3	0,164798	0,0619049
2759	SFXN5_MOUSE	Sfxn5	Sideroflexin-5	0,149896	0,0942974
2760	SFXN2_MOUSE	Sfxn2	Sideroflexin-2	0,222754	-0,116939
2761	TALDO_MOUSE	Taldo1	Transaldolase	0,0502639	0,0223114
2762	ROAA_MOUSE	Hnrnpab	Heterogeneous nuclear ribonucleoprotein A/B	0,304213	0,181231
2763	S14L2_MOUSE	Sec14l2	SEC14-like protein 2	0,571072	0,413261
2764	GHDC_MOUSE	Ghdc	GH3 domain-containing protein	1,22663	0,807487
2765	THUM1_MOUSE	Thumpd1	THUMP domain-containing protein 1	0,910326	0,297153
2766	DCMC_MOUSE	Mlycd	Malonyl-CoA decarboxylase, mitochondrial	0,191756	0,0936146
2767	DRS7B_MOUSE	Dhrs7b	Dehydrogenase/reductase SDR family member 7B	0,538761	0,410563
2768	RFC4_MOUSE	Rfc4	Replication factor C subunit 4	1,35907	0,450285
2769	SIAS_MOUSE	Nans	Sialic acid synthase	0,243221	-0,234763
2770	DHX58_MOUSE	Dhx58	Probable ATP-dependent RNA helicase DHX58	2,14388	0,71088
2771	CDK9_MOUSE	Cdk9	Cyclin-dependent kinase 9	1,6829	-0,636276
2772	THTM_MOUSE	Mpst	3-mercaptopyruvate sulfurtransferase	0,224922	0,173073
2773	STML2_MOUSE	Stoml2	Stomatin-like protein 2, mitochondrial	0,348792	0,344278
2774	PACN3_MOUSE	Pacsin3	Protein kinase C and casein kinase II substrate protein 3	0,474597	0,181736
2775	MVD1_MOUSE	Mvd	Diphosphomevalonate decarboxylase	0,181298	-0,453321
2776	PSIP1_MOUSE	Psip1	PC4 and SFRS1-interacting protein	1,15283	0,397185
2777	RP25L_MOUSE	Rpp25l	Ribonuclease P protein subunit p25-like protein	1,25725	0,55838
2778	PSMD6_MOUSE	Psmd6	26S proteasome non-ATPase regulatory subunit 6	0,868593	-1,0657
2779	RAP1B_MOUSE	Rap1b	Ras-related protein Rap-1b	1,56519	0,197852
2780	HOME3_MOUSE	Homer3	Homer protein homolog 3	0,815355	0,594337
2781	SFXN1_MOUSE	Sfxn1	Sideroflexin-1	0,138974	-0,112987
2782	TINAL_MOUSE	Tinagl1	Tubulointerstitial nephritis antigen-like	0,0906848	0,0820976
2783	NMNA3_MOUSE	Nmnat3	Nicotinamide/nicotinic acid mononucleotide adenylyltransferase 3	0,547672	-0,333942
2784	SMRD2_MOUSE	Smarcd2	SWI/SNF-related matrix-associated actin-dependent regulator of chromatin	0,687649	0,241053
2785	GATB_MOUSE	Gatb	Glutamyl-tRNA(Gln) amidotransferase subunit B, mitochondrial	0,762085	0,371675
2786	MTND_MOUSE	Adi1	1,2-dihydroxy-3-keto-5-methylthiopentene dioxygenase	2,86695	0,364408
2787	ACY1_MOUSE	Acy1	Aminoacylase-1	0,509815	-0,264122
2788	LIMS1_MOUSE	Lims1	LIM and senescent cell antigen-like-containing domain protein 1	0,163632	-0,247995
2789	GORS2_MOUSE	Gorasp2	Golgi reassembly-stacking protein 2	0,389922	0,191243
2790	EIF3M_MOUSE	Eif3m	Eukaryotic translation initiation factor 3 subunit M	0,407783	-0,250414
2791	NXF1_MOUSE	Nxf1	Nuclear RNA export factor 1	1,5104	0,515308
2792	ECHB_MOUSE	Hadhb	Trifunctional enzyme subunit beta, mitochondrial	0,442825	0,191341
2793	GIMA4_MOUSE	Gimap4	GTPase IMAP family member 4	0,0114083	0,00880814
2794	TRABD_MOUSE	Trabd	TraB domain-containing protein	1,58792	0,529511
2795	ARP3_MOUSE	Actr3	Actin-related protein 3	0,595359	-0,275338
2796	PDXD1_MOUSE	Pdxdc1	Pyridoxal-dependent decarboxylase domain-containing protein 1	0,692	0,187651
2797	UFSP2_MOUSE	Ufsp2	Ufm1-specific protease 2	0,0277204	-0,0136166
2798	ARFG2_MOUSE	Arfgap2	ADP-ribosylation factor GTPase-activating protein 2	1,23886	0,405437
2799	ES8L2_MOUSE	Eps8l2	Epidermal growth factor receptor kinase substrate 8-like protein 2	1,30043	0,776799
2800	EMIL1_MOUSE	Emilin1	EMILIN-1	0,518738	0,241713
2801	NONO_MOUSE	Nono	Non-POU domain-containing octamer-binding protein	0,410545	0,268606
2802	PLST_MOUSE	Pls3	Plastin-3	0,434757	0,314777
2803	AASS_MOUSE	Aass	Alpha-aminoadipic semialdehyde synthase, mitochondrial	2,1499	-0,565291

2804	RRAGC_MOUSE	Rragc	Ras-related GTP-binding protein C	0,308338	0,177429
2805	SERC_MOUSE	Psat1	Phosphoserine aminotransferase	1,70236	1,83904
2806	RTF2_MOUSE	Rtf2	Replication termination factor 2	2,07805	1,46
2807	GLO2_MOUSE	Hagh	Hydroxyacylglutathione hydrolase, mitochondrial	0,69025	0,42654
2808	VMA5A_MOUSE	Vwa5a	von Willebrand factor A domain-containing protein 5A	0,219931	0,174913
2809	UN45A_MOUSE	Unc45a	Protein unc-45 homolog A	0,205014	0,12565
2810	MAOM_MOUSE	Me2	NAD-dependent malic enzyme, mitochondrial	0,354534	0,204102
2811	TMED9_MOUSE	Tmed9	Transmembrane emp24 domain-containing protein 9	0,0413936	-0,0268021
2812	RBM10_MOUSE	Rbm10	RNA-binding protein 10	0,828193	0,241886
2813	LSR_MOUSE	Lsr	Lipolysis-stimulated lipoprotein receptor	0,197729	0,350111
2814	STK24_MOUSE	Stk24	Serine/threonine-protein kinase 24	0,241593	-0,465971
2815	ACON_MOUSE	Aco2	Aconitate hydratase, mitochondrial	0,0338953	-0,0253662
2816	DCTN2_MOUSE	Dctn2	Dynactin subunit 2	1,36358	0,432841
2817	NEUA_MOUSE	Cmas	N-acylneuraminate cytidylyltransferase	1,45545	0,556095
2818	DPP3_MOUSE	Dpp3	Dipeptidyl peptidase 3	0,241087	-0,116549
2819	SYHM_MOUSE	Hars2	Probable histidine--tRNA ligase, mitochondrial	1,05718	0,608889
2820	CIAO1_MOUSE	Ciao1	Probable cytosolic iron-sulfur protein assembly protein CIAO1	0,428993	0,31245
2821	EPN4_MOUSE	Clint1	Clathrin interactor 1	0,0955051	-0,0767277
2822	CRYL1_MOUSE	Cryl1	Lambda-crystallin homolog	0,261845	-0,545572
2823	PRP19_MOUSE	Prpf19	Pre-mRNA-processing factor 19	0,0691824	0,0526581
2824	NAMPT_MOUSE	Nampt	Nicotinamide phosphoribosyltransferase	1,15656	0,359895
2825	LACB2_MOUSE	Lactb2	Endoribonuclease LACTB2	0,513372	0,245379
2826	PPIF_MOUSE	Ppif	Peptidyl-prolyl cis-trans isomerase F, mitochondrial	0,668474	-0,340483
2827	DJB11_MOUSE	Dnajb11	DnaJ homolog subfamily B member 11	0,732947	0,54519
2828	TARA_MOUSE	Triobp	TRIO and F-actin-binding protein	0,802262	0,366582
2829	MLF2_MOUSE	Mlf2	Myeloid leukemia factor 2	1,19943	0,642433
2830	GAK_MOUSE	Gak	Cyclin-G-associated kinase	0,150877	0,0585354
2831	DHRS1_MOUSE	Dhrs1	Dehydrogenase/reductase SDR family member 1	2,0284	0,465301
2832	3HIDH_MOUSE	Hibadh	3-hydroxyisobutyrate dehydrogenase, mitochondrial	0,47864	0,172471
2833	GSTT3_MOUSE	Gstt3	Glutathione S-transferase theta-3	0,824337	-0,544962
2834	GMPR2_MOUSE	Gmpr2	GMP reductase 2	0,96238	0,378452
2835	IF2B_MOUSE	Eif2s2	Eukaryotic translation initiation factor 2 subunit 2	1,04811	0,60762
2836	F10A1_MOUSE	St13	Hsc70-interacting protein	1,17807	0,525565
2837	SNTB1_MOUSE	Sntb1	Beta-1-syntrophin	1,32621	0,146969
2838	DHRS4_MOUSE	Dhrs4	Dehydrogenase/reductase SDR family member 4	1,82733	0,6955
2839	MAT2B_MOUSE	Mat2b	Methionine adenosyltransferase 2 subunit beta	0,151401	-0,180743
2840	SARDH_MOUSE	Sardh	Sarcosine dehydrogenase, mitochondrial	0,102621	-0,0942528
2841	CSTF1_MOUSE	Cstf1	Cleavage stimulation factor subunit 1	0,272171	0,103218
2842	NDUAA_MOUSE	Ndufa10	NADH dehydrogenase [ubiquinone] 1 alpha subcomplex subunit 10, mitochc	1,62299	-0,663675
2843	ETFA_MOUSE	Etfa	Electron transfer flavoprotein subunit alpha, mitochondrial	0,0621159	-0,035841
2844	EI2BA_MOUSE	Eif2b1	Translation initiation factor eIF-2B subunit alpha	0,0144377	0,0129742
2845	PEX6_MOUSE	Pex6	Peroxisome assembly factor 2	1,04287	0,859903
2846	CSN1_MOUSE	Gps1	COP9 signalosome complex subunit 1	0,204096	-0,152526
2847	DDAH2_MOUSE	Ddah2	N(G),N(G)-dimethylarginine dimethylaminohydrolase 2	0,407847	-0,157195
2848	EI2BB_MOUSE	Eif2b2	Translation initiation factor eIF-2B subunit beta	0,04574	0,033839
2849	ABCF2_MOUSE	Abcf2	ATP-binding cassette sub-family F member 2	1,28259	0,501963
2850	RTCB_MOUSE	Rtcb	tRNA-splicing ligase RtcB homolog	0,436069	0,231028
2851	UBP16_MOUSE	Usp16	Ubiquitin carboxyl-terminal hydrolase 16	1,10534	0,526481
2852	CSTF3_MOUSE	Cstf3	Cleavage stimulation factor subunit 3	0,00714016	-0,0105984
2853	HGS_MOUSE	Hgs	Hepatocyte growth factor-regulated tyrosine kinase substrate	0,976788	0,483723
2854	CLP1_MOUSE	Clp1	Polyribonucleotide 5'-hydroxyl-kinase Clp1	1,29846	0,596835
2855	GPX7_MOUSE	Gpx7	Glutathione peroxidase 7	0,733951	0,320808

2856	PWP1_MOUSE	Pwp1	Periodic tryptophan protein 1 homolog	0,482574	0,482602
2857	CK5P3_MOUSE	Cdk5rap3	CDK5 regulatory subunit-associated protein 3	0,0872006	0,0607723
2858	DOHH_MOUSE	Dohh	Deoxyhypusine hydroxylase	0,492771	0,324611
2859	GRPE1_MOUSE	Grpel1	GrpE protein homolog 1, mitochondrial	0,294089	0,133847
2860	ABD12_MOUSE	Abhd12	Lysophosphatidylserine lipase ABHD12	0,670356	0,761686
2861	SERB_MOUSE	Psph	Phosphoserine phosphatase	1,24194	0,573192
2862	DPY30_MOUSE	Dpy30	Protein dpy-30 homolog	0,0708805	-0,0304218
2863	CH1B1_MOUSE	Chmp1b1	Charged multivesicular body protein 1b-1	0,115837	-0,0263027
2864	PARK7_MOUSE	Park7	Protein/nucleic acid deglycase DJ-1	0,499645	0,491
2865	NDUS5_MOUSE	Ndufs5	NADH dehydrogenase [ubiquinone] iron-sulfur protein 5	0,606608	-1,32013
2866	LIAS_MOUSE	Lias	Lipoyl synthase, mitochondrial	1,04202	0,753648
2867	PPIP2_MOUSE	Pstpip2	Proline-serine-threonine phosphatase-interacting protein 2	0,59916	0,5686
2868	RNPS1_MOUSE	Rnps1	RNA-binding protein with serine-rich domain 1	1,11121	0,424879
2869	HSP7E_MOUSE	Hspa14	Heat shock 70 kDa protein 14	1,94834	1,58064
2870	NCK1_MOUSE	Nck1	Cytoplasmic protein NCK1	0,418733	-0,200391
2871	DNJA3_MOUSE	Dnaja3	DnaJ homolog subfamily A member 3, mitochondrial	0,621764	0,53104
2872	NASP_MOUSE	Nasp	Nuclear autoantigenic sperm protein	0,500406	0,502045
2873	NOG1_MOUSE	Gtpbp4	Nucleolar GTP-binding protein 1	0,438758	0,27328
2874	RB6I2_MOUSE	Erc1	ELKS/Rab6-interacting/CAST family member 1	0,245839	0,144099
2875	DDX50_MOUSE	Ddx50	ATP-dependent RNA helicase DDX50	0,968582	0,501705
2876	ARBK1_MOUSE	Grk2	Beta-adrenergic receptor kinase 1	0,0470639	-0,0645077
2877	SYK_MOUSE	Kars	Lysine--tRNA ligase	0,17431	0,0783958
2878	PCCB_MOUSE	Pccb	Propionyl-CoA carboxylase beta chain, mitochondrial	0,0288177	-0,037582
2879	BRAP_MOUSE	Brap	BRCA1-associated protein	0,232335	-0,184884
2880	SRRT_MOUSE	Srrt	Serrate RNA effector molecule homolog	0,411871	-0,180456
2881	MCCA_MOUSE	Mccc1	Methylcrotonoyl-CoA carboxylase subunit alpha, mitochondrial	0,271384	0,162399
2882	MLXPL_MOUSE	Mlxipl	Carbohydrate-responsive element-binding protein	0,433206	0,410019
2883	PECR_MOUSE	Pecr	Peroxisomal trans-2-enoyl-CoA reductase	0,00671981	-0,00460129
2884	TYPH_MOUSE	Tymp	Thymidine phosphorylase	0,0170215	-0,0194809
2885	RT18B_MOUSE	Mrps18b	28S ribosomal protein S18b, mitochondrial	0,288041	-0,457663
2886	RT18A_MOUSE	Mrps18a	28S ribosomal protein S18a, mitochondrial	0,671666	0,426963
2887	RT05_MOUSE	Mrps5	28S ribosomal protein S5, mitochondrial	0,273508	0,198383
2888	RM43_MOUSE	Mrpl43	39S ribosomal protein L43, mitochondrial	0,206043	-0,0741123
2889	RM34_MOUSE	Mrpl34	39S ribosomal protein L34, mitochondrial	0,241448	0,264737
2890	RM27_MOUSE	Mrpl27	39S ribosomal protein L27, mitochondrial	0,0429644	-0,0295963
2891	RM09_MOUSE	Mrpl9	39S ribosomal protein L9, mitochondrial	0,0734539	0,0501068
2892	RM03_MOUSE	Mrpl3	39S ribosomal protein L3, mitochondrial	1,11324	-0,290558
2893	RM01_MOUSE	Mrpl1	39S ribosomal protein L1, mitochondrial	0,0790256	0,0577705
2894	SF3B1_MOUSE	Sf3b1	Splicing factor 3B subunit 1	0,764995	-0,245968
2895	ANR17_MOUSE	Ankrd17	Ankyrin repeat domain-containing protein 17	0,0293629	0,0113754
2896	PARD3_MOUSE	Pard3	Partitioning defective 3 homolog	1,6952	0,519549
2897	PG12B_MOUSE	Pla2g12b	Group XIIB secretory phospholipase A2-like protein	1,01998	1,15967
2898	NUDT7_MOUSE	Nudt7	Peroxisomal coenzyme A diphosphatase NUDT7	0,850481	0,867003
2899	HPBP1_MOUSE	Hspbp1	Hsp70-binding protein 1	0,890933	-0,408436
2900	NU155_MOUSE	Nup155	Nuclear pore complex protein Nup155	0,493085	-0,237752
2901	AAAD_MOUSE	Aadac	Arylacetamide deacetylase	0,155631	-0,0975368
2902	OGFR_MOUSE	Ogfr	Opioid growth factor receptor	0,652864	0,423294
2903	LPIN2_MOUSE	Lpin2	Phosphatidate phosphatase LPIN2	0,536035	-0,565951
2904	RRBP1_MOUSE	Rrbp1	Ribosome-binding protein 1	1,1641	0,267319
2905	UBXN6_MOUSE	Ubxn6	UBX domain-containing protein 6	0,669786	0,486582
2906	GDIR1_MOUSE	Arhgdia	Rho GDP-dissociation inhibitor 1	0,150321	-0,0446949
2907	DHX30_MOUSE	Dhx30	ATP-dependent RNA helicase DHX30	0,962119	0,478506

2908	PRP8_MOUSE	Prpf8	Pre-mRNA-processing-splicing factor 8	0,125546	0,0661201
2909	PRPK_MOUSE	Tp53rk	EKC/KEOPS complex subunit Tp53rk	0,655275	0,381578
2910	IF2B3_MOUSE	Igf2bp3	Insulin-like growth factor 2 mRNA-binding protein 3	0,276113	0,26427
2911	NDUA5_MOUSE	Ndufa5	NADH dehydrogenase [ubiquinone] 1 alpha subcomplex subunit 5	0,478008	0,276111
2912	COX6C_MOUSE	Cox6c	Cytochrome c oxidase subunit 6C	0,22045	-0,224301
2913	ATP5L_MOUSE	Atp5mg	ATP synthase subunit g, mitochondrial	0,586002	-0,22541
2914	RL17_MOUSE	Rpl17	60S ribosomal protein L17	1,14544	0,631869
2915	RM15_MOUSE	Mrpl15	39S ribosomal protein L15, mitochondrial	0,21041	-0,0734715
2916	NANP_MOUSE	Nanp	N-acylneuraminate-9-phosphatase	0,520394	0,170988
2917	MYDGF_MOUSE	Mydgf	Myeloid-derived growth factor	0,489316	0,403655
2918	NOP16_MOUSE	Nop16	Nucleolar protein 16	0,407999	0,139479
2919	LGUL_MOUSE	Glo1	Lactoylglutathione lyase	1,51275	0,328864
2920	GLOD4_MOUSE	Glod4	Glyoxalase domain-containing protein 4	0,542532	-0,162641
2921	ARPC5_MOUSE	Arpc5	Actin-related protein 2/3 complex subunit 5	0,633307	0,397522
2922	SSRB_MOUSE	Ssr2	Translocon-associated protein subunit beta	0,518595	0,200738
2923	MAP12_MOUSE	Metap1d	Methionine aminopeptidase 1D, mitochondrial	0,533759	0,619183
2924	ATG3_MOUSE	Atg3	Ubiquitin-like-conjugating enzyme ATG3	1,03855	0,791927
2925	RT16_MOUSE	Mrps16	28S ribosomal protein S16, mitochondrial	0,233785	-0,206552
2926	MRM2_MOUSE	Mrm2	rRNA methyltransferase 2, mitochondrial	1,06728	0,326455
2927	RM51_MOUSE	Mrpl51	39S ribosomal protein L51, mitochondrial	0,854457	1,29238
2928	AMPL_MOUSE	Lap3	Cytosol aminopeptidase	0,455348	0,284155
2929	COXM1_MOUSE	Cmc1	COX assembly mitochondrial protein homolog	0,155033	-0,252271
2930	RM24_MOUSE	Mrpl24	39S ribosomal protein L24, mitochondrial	0,589415	-0,135793
2931	CHMP3_MOUSE	Chmp3	Charged multivesicular body protein 3	0,49588	-0,431159
2932	LTOR1_MOUSE	Lamtor1	Ragulator complex protein LAMTOR1	0,319568	-0,248373
2933	RM49_MOUSE	Mrpl49	39S ribosomal protein L49, mitochondrial	0,778315	-0,258187
2934	NUDC2_MOUSE	Nudcd2	NudC domain-containing protein 2	0,727145	0,83367
2935	NCBP2_MOUSE	Ncbp2	Nuclear cap-binding protein subunit 2	0,868436	0,391335
2936	NDUC2_MOUSE	Ndufc2	NADH dehydrogenase [ubiquinone] 1 subunit C2	1,32547	-0,516661
2937	USE1_MOUSE	Use1	Vesicle transport protein USE1	2,51219	0,464207
2938	6PGL_MOUSE	Pgls	6-phosphogluconolactonase	1,02861	0,757648
2939	DECR_MOUSE	Decr1	2,4-dienoyl-CoA reductase, mitochondrial	0,467633	0,359429
2940	MTAP_MOUSE	Mtap	S-methyl-5'-thioadenosine phosphorylase	0,416822	0,178035
2941	QCR8_MOUSE	Uqcrq	Cytochrome b-c1 complex subunit 8	0,473108	0,284129
2942	NDUA2_MOUSE	Ndufa2	NADH dehydrogenase [ubiquinone] 1 alpha subcomplex subunit 2	0,176294	0,183871
2943	TXND9_MOUSE	Txndc9	Thioredoxin domain-containing protein 9	0,916972	0,403223
2944	VPS25_MOUSE	Vps25	Vacuolar protein-sorting-associated protein 25	1,11273	-0,367715
2945	TIM22_MOUSE	Timm22	Mitochondrial import inner membrane translocase subunit Tim22	1,2587	-0,583173
2946	FIS1_MOUSE	Fis1	Mitochondrial fission 1 protein	1,47104	0,934768
2947	TPPC5_MOUSE	Trappc5	Trafficking protein particle complex subunit 5	1,2635	0,376024
2948	SDHB_MOUSE	Sdhb	Succinate dehydrogenase [ubiquinone] iron-sulfur subunit, mitochondrial	0,692047	0,204164
2949	MCRI2_MOUSE	Mcrip2	MAPK regulated corepressor interacting protein 2	1,37058	0,557104
2950	BZW1_MOUSE	Bzw1	Basic leucine zipper and W2 domain-containing protein 1	1,01101	-0,423409
2951	NDUB4_MOUSE	Ndufb4	NADH dehydrogenase [ubiquinone] 1 beta subcomplex subunit 4	0,0880308	0,0687328
2952	SAR1B_MOUSE	Sar1b	GTP-binding protein SAR1b	0,725967	0,2853
2953	RAB5A_MOUSE	Rab5a	Ras-related protein Rab-5A	0,945987	-0,525987
2954	CH1B2_MOUSE	Chmp1b2	Charged multivesicular body protein 1b-2	0,363756	-0,140881
2955	NPS3B_MOUSE	Nipsnap3b	Protein NipSnap homolog 3B	0,259015	-0,179916
2956	RTRAF_MOUSE	RTRAF	RNA transcription, translation and transport factor protein	0,266928	-0,123285
2957	RM11_MOUSE	Mrpl11	39S ribosomal protein L11, mitochondrial	1,0475	0,527415
2958	CPSF5_MOUSE	Nudt21	Cleavage and polyadenylation specificity factor subunit 5	0,045875	0,032444
2959	PCYOX_MOUSE	Pcyox1	Prenylcysteine oxidase	0,095543	0,0640579

2960	NDUB5_MOUSE	Ndufb5	NADH dehydrogenase [ubiquinone] 1 beta subcomplex subunit 5, mitochonc	0,23777	0,13773
2961	BT3L4_MOUSE	Btf3l4	Transcription factor BTF3 homolog 4	0,42979	0,135126
2962	GMFB_MOUSE	Gmfb	Glia maturation factor beta	0,0772269	-0,0705147
2963	COTL1_MOUSE	Cotl1	Coactosin-like protein	0,980352	-0,523127
2964	RU2B_MOUSE	Snrpb2	U2 small nuclear ribonucleoprotein B''	0,168058	-0,112129
2965	DENR_MOUSE	Denr	Density-regulated protein	0,146317	-0,165851
2966	NDUB9_MOUSE	Ndufb9	NADH dehydrogenase [ubiquinone] 1 beta subcomplex subunit 9	0,000949562	-0,000593948
2967	RM18_MOUSE	Mrpl18	39S ribosomal protein L18, mitochondrial	0,0775847	0,0901375
2968	RM35_MOUSE	Mrpl35	39S ribosomal protein L35, mitochondrial	0,773606	0,793279
2969	MOFA1_MOUSE	Mrfap1	MORF4 family-associated protein 1	0,159476	0,281977
2970	TXD17_MOUSE	Txndc17	Thioredoxin domain-containing protein 17	0,789704	0,301485
2971	GLRX3_MOUSE	Glrx3	Glutaredoxin-3	0,064854	0,0483154
2972	TRAP1_MOUSE	Trap1	Heat shock protein 75 kDa, mitochondrial	0,178047	-0,0600727
2973	RM41_MOUSE	Mrpl41	39S ribosomal protein L41, mitochondrial	1,02381	-0,759836
2974	RM33_MOUSE	Mrpl33	39S ribosomal protein L33, mitochondrial	0,75347	0,708418
2975	CHCH5_MOUSE	Chchd5	Coiled-coil-helix-coiled-coil-helix domain-containing protein 5	0,436502	0,355867
2976	AT5F1_MOUSE	Atp5pb	ATP synthase F(0) complex subunit B1, mitochondrial	0,556291	0,478451
2977	LSM7_MOUSE	Lsm7	U6 snRNA-associated Sm-like protein LSm7	0,675341	-1,03159
2978	RS21_MOUSE	Rps21	40S ribosomal protein S21	0,306036	0,209008
2979	ACO13_MOUSE	Acot13	Acyl-coenzyme A thioesterase 13	0,430207	-0,218213
2980	PPP6_MOUSE	Ppp6c	Serine/threonine-protein phosphatase 6 catalytic subunit	0,16013	0,138279
2981	NOP10_MOUSE	Nop10	H/ACA ribonucleoprotein complex subunit 3	0,17144	-0,134076
2982	MTNA_MOUSE	Mri1	Methylthioribose-1-phosphate isomerase	0,329659	0,235551
2983	RBM7_MOUSE	Rbm7	RNA-binding protein 7	1,07296	0,653957
2984	TXD12_MOUSE	Txndc12	Thioredoxin domain-containing protein 12	0,33612	0,134657
2985	TIM16_MOUSE	Pam16	Mitochondrial import inner membrane translocase subunit TIM16	0,00935326	-0,00558662
2986	RT24_MOUSE	Mrps24	28S ribosomal protein S24, mitochondrial	0,39098	0,13838
2987	TIM14_MOUSE	Dnajc19	Mitochondrial import inner membrane translocase subunit TIM14	0,0675361	-0,0207211
2988	1433B_MOUSE	Ywhab	14-3-3 protein beta/alpha	0,766976	-0,296624
2989	YKT6_MOUSE	Ykt6	Synaptobrevin homolog YKT6	0,554554	-0,730363
2990	ARL8B_MOUSE	Arl8b	ADP-ribosylation factor-like protein 8B	0,119119	-0,0916393
2991	IFM3_MOUSE	Ifitm3	Interferon-induced transmembrane protein 3	0,0488266	-0,0463665
2992	CYB5B_MOUSE	Cyb5b	Cytochrome b5 type B	0,74163	0,377939
2993	NDUA6_MOUSE	Ndufa6	NADH dehydrogenase [ubiquinone] 1 alpha subcomplex subunit 6	0,113266	0,0231796
2994	NDUB3_MOUSE	Ndufb3	NADH dehydrogenase [ubiquinone] 1 beta subcomplex subunit 3	0,772279	-0,358015
2995	PSMD9_MOUSE	Psmd9	26S proteasome non-ATPase regulatory subunit 9	0,37176	0,162971
2996	RPP29_MOUSE	Pop4	Ribonuclease P protein subunit p29	0,223293	0,447009
2997	UFC1_MOUSE	Ufc1	Ubiquitin-fold modifier-conjugating enzyme 1	0,0408358	-0,0310154
2998	OXLD1_MOUSE	Oxld1	Oxidoreductase-like domain-containing protein 1	1,38931	-0,840195
2999	PPID_MOUSE	Ppid	Peptidyl-prolyl cis-trans isomerase D	0,282406	-0,189948
3000	TMEM9_MOUSE	Tmem9	Transmembrane protein 9	0,645433	0,528557
3001	VTA1_MOUSE	Vta1	Vacuolar protein sorting-associated protein VTA1 homolog	0,520457	0,199392
3002	WASC3_MOUSE	Washc3	WASH complex subunit 3	0,326267	0,15512
3003	HYPK_MOUSE	Hypk	Huntingtin-interacting protein K	0,114479	0,0435699
3004	KBRS2_MOUSE	Nkiras2	NF-kappa-B inhibitor-interacting Ras-like protein 2	1,37527	0,356972
3005	RL14_MOUSE	Rpl14	60S ribosomal protein L14	0,593146	0,293003
3006	G45IP_MOUSE	Gadd45gip1	Growth arrest and DNA damage-inducible proteins-interacting protein 1	0,471533	0,201465
3007	NDUB7_MOUSE	Ndufb7	NADH dehydrogenase [ubiquinone] 1 beta subcomplex subunit 7	0,502887	-0,795546
3008	M2OM_MOUSE	Slc25a11	Mitochondrial 2-oxoglutarate/malate carrier protein	0,964613	-0,324453
3009	UCRI_MOUSE	Uqcrfs1	Cytochrome b-c1 complex subunit Rieske, mitochondrial	0,366447	0,114927
3010	CHSP1_MOUSE	Carhsp1	Calcium-regulated heat stable protein 1	0,140622	0,0774952
3011	RT14_MOUSE	Mrps14	28S ribosomal protein S14, mitochondrial	0,34079	0,22729

3012	F136A_MOUSE	Fam136a	Protein FAM136A	1,38086	0,60627
3013	GOLP3_MOUSE	Golph3	Golgi phosphoprotein 3	1,40763	0,503898
3014	ATP5S_MOUSE	Dmac2l	ATP synthase subunit s, mitochondrial	1,29823	-0,246856
3015	EXOS5_MOUSE	Exosc5	Exosome complex component RRP46	1,27055	0,300498
3016	SNX24_MOUSE	Snx24	Sorting nexin-24	0,083879	0,0627338
3017	NHP2_MOUSE	Nhp2	H/ACA ribonucleoprotein complex subunit 2	0,00299617	0,00380688
3018	HIUH_MOUSE	Urah	5-hydroxyisourate hydrolase	0,0252522	-0,0206341
3019	MTFP1_MOUSE	Mtfp1	Mitochondrial fission process protein 1	0,272257	-0,33007
3020	MIC19_MOUSE	Chchd3	MICOS complex subunit Mic19	1,11697	0,432291
3021	LRC40_MOUSE	Lrrc40	Leucine-rich repeat-containing protein 40	0,529847	0,103696
3022	OCAD1_MOUSE	Ociad1	OCIA domain-containing protein 1	0,0296015	-0,0277191
3023	EMC2_MOUSE	Emc2	ER membrane protein complex subunit 2	1,21466	0,473907
3024	ECD_MOUSE	Ecd	Protein ecdysoneless homolog	0,637251	0,405288
3025	RRP44_MOUSE	Dis3	Exosome complex exonuclease RRP44	2,25215	0,476709
3026	SNW1_MOUSE	Snw1	SNW domain-containing protein 1	0,932573	0,392738
3027	RPR1B_MOUSE	Rprd1b	Regulation of nuclear pre-mRNA domain-containing protein 1B	0,114675	-0,123489
3028	RANB3_MOUSE	Ranbp3	Ran-binding protein 3	0,0805083	0,0356613
3029	SMC1A_MOUSE	Smc1a	Structural maintenance of chromosomes protein 1A	1,12433	0,571707
3030	ARPC2_MOUSE	Arpc2	Actin-related protein 2/3 complex subunit 2	0,311137	-0,0917629
3031	SMC3_MOUSE	Smc3	Structural maintenance of chromosomes protein 3	0,503368	0,286504
3032	MARC1_MOUSE	Marc1	Mitochondrial amidoxime-reducing component 1	0,58026	0,339017
3033	RAVR1_MOUSE	Raver1	Ribonucleoprotein PTB-binding 1	0,921975	0,443255
3034	GOGA1_MOUSE	Golga1	Golgin subfamily A member 1	0,800624	1,30245
3035	NUBPL_MOUSE	Nubpl	Iron-sulfur protein NUBPL	0,245363	0,0665047
3036	MFR1L_MOUSE	Mtfr1l	Mitochondrial fission regulator 1-like	0,554933	0,428344
3037	NDUF7_MOUSE	Ndufaf7	Protein arginine methyltransferase NDUFAF7, mitochondrial	0,473434	0,277349
3038	BCCIP_MOUSE	Bccip	BRCA2 and CDKN1A-interacting protein	0,196602	0,248809
3039	PUR9_MOUSE	Atic	Bifunctional purine biosynthesis protein PURH	0,560034	0,456456
3040	CD2B2_MOUSE	Cd2bp2	CD2 antigen cytoplasmic tail-binding protein 2	1,82025	2,27045
3041	SNX2_MOUSE	Snx2	Sorting nexin-2	1,12694	0,350622
3042	CTBL1_MOUSE	Ctnnbl1	Beta-catenin-like protein 1	0,411277	-0,140847
3043	PFD1_MOUSE	Pfdn1	Prefoldin subunit 1	0,410591	0,249056
3044	MSPD2_MOUSE	Mospd2	Motile sperm domain-containing protein 2	0,168219	-0,144393
3045	WDR73_MOUSE	Wdr73	WD repeat-containing protein 73	0,427667	-0,501622
3046	DDAH1_MOUSE	Ddah1	N(G),N(G)-dimethylarginine dimethylaminohydrolase 1	0,525563	-0,222716
3047	INT11_MOUSE	Ints11	Integrator complex subunit 11	1,18288	0,456213
3048	DDX28_MOUSE	Ddx28	Probable ATP-dependent RNA helicase DDX28	0,498538	0,734023
3049	PIN4_MOUSE	Pin4	Peptidyl-prolyl cis-trans isomerase NIMA-interacting 4	2,17182	0,713793
3050	SETD6_MOUSE	Setd6	N-lysine methyltransferase SETD6	1,31926	0,94779
3051	RNH2A_MOUSE	Rnaseh2a	Ribonuclease H2 subunit A	0,475885	0,220489
3052	RBM8A_MOUSE	Rbm8a	RNA-binding protein 8A	0,576387	-0,820043
3053	SNAG_MOUSE	Napg	Gamma-soluble NSF attachment protein	0,716157	0,525899
3054	IST1_MOUSE	Ist1	IST1 homolog	0,395831	-0,160492
3055	CJ143_MOUSE		Uncharacterized protein C10orf143 homolog	0,404084	-0,311963
3056	SGT1_MOUSE	Sugt1	Protein SGT1 homolog	0,486812	0,157674
3057	PSMD8_MOUSE	Psmd8	26S proteasome non-ATPase regulatory subunit 8	0,316433	0,187453
3058	CYGB_MOUSE	Cygb	Cytoglobin	0,151657	0,0912785
3059	ROA0_MOUSE	Hnrnpa0	Heterogeneous nuclear ribonucleoprotein A0	0,700224	-0,332498
3060	CP2U1_MOUSE	Cyp2u1	Cytochrome P450 2U1	0,00771043	0,00996475
3061	T3HPD_MOUSE	L3hypdh	Trans-L-3-hydroxyproline dehydratase	1,44823	0,535605
3062	TMED5_MOUSE	Tmed5	Transmembrane emp24 domain-containing protein 5	0,844991	1,81035
3063	KYNU_MOUSE	Kynu	Kynureninase	0,767708	-0,744346

3064	TBC15_MOUSE	Tbc1d15	TBC1 domain family member 15	0,371648	-0,78484
3065	COQ5_MOUSE	Coq5	2-methoxy-6-polyprenyl-1,4-benzoquinol methylase, mitochondrial	0,0470292	-0,016116
3066	MANF_MOUSE	Manf	Mesencephalic astrocyte-derived neurotrophic factor	2,55984	0,866473
3067	SYEM_MOUSE	Ears2	Probable glutamate--tRNA ligase, mitochondrial	0,128504	-0,109811
3068	ABCB8_MOUSE	Abcb8	ATP-binding cassette sub-family B member 8, mitochondrial	0,359525	-0,586245
3069	NIP7_MOUSE	Nip7	60S ribosome subunit biogenesis protein NIP7 homolog	0,989049	0,397232
3070	PBLD2_MOUSE	Pbld2	Phenazine biosynthesis-like domain-containing protein 2	0,760488	0,482023
3071	DHRS7_MOUSE	Dhrs7	Dehydrogenase/reductase SDR family member 7	0,192274	0,605262
3072	CENPV_MOUSE	Cenpv	Centromere protein V	0,535773	0,538649
3073	MPPB_MOUSE	Pmpcb	Mitochondrial-processing peptidase subunit beta	1,31135	-0,322819
3074	DHSD_MOUSE	Sdhd	Succinate dehydrogenase [ubiquinone] cytochrome b small subunit, mitochc	0,353742	0,668078
3075	RT22_MOUSE	Mrps22	28S ribosomal protein S22, mitochondrial	0,431383	0,139582
3076	CYBP_MOUSE	Cacybp	Calcyclin-binding protein	1,43769	0,593061
3077	RL11_MOUSE	Rpl11	60S ribosomal protein L11	0,146407	0,047242
3078	ILF2_MOUSE	Ilf2	Interleukin enhancer-binding factor 2	0,103138	0,0742306
3079	NDUS4_MOUSE	Ndufs4	NADH dehydrogenase [ubiquinone] iron-sulfur protein 4, mitochondrial	0,333347	-0,18998
3080	AHSP_MOUSE	Ahsp	Alpha-hemoglobin-stabilizing protein	0,262823	-0,172865
3081	RT28_MOUSE	Mrps28	28S ribosomal protein S28, mitochondrial	0,934852	0,386723
3082	TECR_MOUSE	Tecr	Very-long-chain enoyl-CoA reductase	0,0161109	-0,0185425
3083	GTPB8_MOUSE	Gtpbp8	GTP-binding protein 8	0,428364	0,22732
3084	SSRA_MOUSE	Ssr1	Translocon-associated protein subunit alpha	0,0362904	-0,0324898
3085	CHTOP_MOUSE	Chtop	Chromatin target of PRMT1 protein	2,66626	2,08366
3086	PAIRB_MOUSE	Serbp1	Plasminogen activator inhibitor 1 RNA-binding protein	0,345168	-0,102677
3087	RN181_MOUSE	Rnf181	E3 ubiquitin-protein ligase RNF181	0,898185	0,66977
3088	BIEA_MOUSE	Blvra	Biliverdin reductase A	0,289625	-0,0659889
3089	CREL2_MOUSE	Creld2	Cysteine-rich with EGF-like domain protein 2	0,917575	0,588742
3090	ZCHC8_MOUSE	Zcchc8	Zinc finger CCHC domain-containing protein 8	0,862022	0,288486
3091	TOM34_MOUSE	Tomm34	Mitochondrial import receptor subunit TOM34	0,90217	0,244733
3092	PXL2A_MOUSE	Prxl2a	Peroxiredoxin-like 2A	0,204953	0,126191
3093	RRS1_MOUSE	Rrs1	Ribosome biogenesis regulatory protein homolog	1,47423	0,563902
3094	LUC7L_MOUSE	Luc7l	Putative RNA-binding protein Luc7-like 1	0,476271	0,308821
3095	SYWM_MOUSE	Wars2	Tryptophan--tRNA ligase, mitochondrial	0,253735	0,137398
3096	SPCS2_MOUSE	Spcs2	Signal peptidase complex subunit 2	0,109783	0,0670372
3097	SSBP_MOUSE	Ssbp1	Single-stranded DNA-binding protein, mitochondrial	0,668037	0,262398
3098	AGM1_MOUSE	Pgm3	Phosphoacetylglucosamine mutase	0,823838	0,536994
3099	CB072_MOUSE		Uncharacterized protein C2orf72 homolog	0,640749	0,218247
3100	TM135_MOUSE	Tmem135	Transmembrane protein 135	0,136681	0,103483
3101	HDHD3_MOUSE	Hdhd3	Haloacid dehalogenase-like hydrolase domain-containing protein 3	0,36304	0,181342
3102	TPD54_MOUSE	Tpd52l2	Tumor protein D54	0,0365818	0,0319557
3103	REX1B_MOUSE	Rex1bd	Required for excision 1-B domain-containing protein	2,37478	1,29121
3104	CSN7A_MOUSE	Cops7a	COP9 signalosome complex subunit 7a	0,15811	-0,285129
3105	QCR1_MOUSE	Uqcrc1	Cytochrome b-c1 complex subunit 1, mitochondrial	0,123991	-0,137682
3106	OLA1_MOUSE	Ola1	Obg-like ATPase 1	0,499019	0,233215
3107	NNRD_MOUSE	Naxd	ATP-dependent (S)-NAD(P)H-hydrate dehydratase	0,308971	0,124687
3108	NSF1C_MOUSE	Nsfl1c	NSFL1 cofactor p47	0,583844	0,33171
3109	RM55_MOUSE	Mrpl55	39S ribosomal protein L55, mitochondrial	0,0999021	0,0433929
3110	GARS_MOUSE	Gars	Glycine--tRNA ligase	0,47268	0,13581
3111	IF3M_MOUSE	Mtif3	Translation initiation factor IF-3, mitochondrial	1,009	0,518348
3112	PSMG3_MOUSE	Psmg3	Proteasome assembly chaperone 3	0,206644	0,173025
3113	HS12B_MOUSE	Hspa12b	Heat shock 70 kDa protein 12B	0,487899	-0,333621
3114	PHS2_MOUSE	Pcbd2	Pterin-4-alpha-carbinolamine dehydratase 2	0,123474	-0,107612
3115	RL15_MOUSE	Rpl15	60S ribosomal protein L15	1,25049	0,352262

3116	GLYM_MOUSE	Shmt2	Serine hydroxymethyltransferase, mitochondrial	0,136258	0,104391
3117	NALD2_MOUSE	Naalad2	N-acetylated-alpha-linked acidic dipeptidase 2	0,303671	-0,220543
3118	EFTS_MOUSE	Tsfm	Elongation factor Ts, mitochondrial	1,19473	0,383027
3119	AL1B1_MOUSE	Aldh1b1	Aldehyde dehydrogenase X, mitochondrial	0,257063	-0,199332
3120	CMS1_MOUSE	Cmss1	Protein CMSS1	0,407103	0,24029
3121	MTREX_MOUSE	Mtrex	Exosome RNA helicase MTR4	0,224604	0,16221
3122	ERAL1_MOUSE	Eral1	GTPase Era, mitochondrial	0,386937	-0,37612
3123	CISY_MOUSE	Cs	Citrate synthase, mitochondrial	0,499342	-0,241584
3124	TOM70_MOUSE	Tomm70	Mitochondrial import receptor subunit TOM70	0,29378	0,15303
3125	ELP3_MOUSE	Elp3	Elongator complex protein 3	0,653376	0,240352
3126	RS19_MOUSE	Rps19	40S ribosomal protein S19	0,0999217	0,0317184
3127	5NT3A_MOUSE	Nt5c3a	Cytosolic 5'-nucleotidase 3A	0,219473	0,199269
3128	MPC2_MOUSE	Mpc2	Mitochondrial pyruvate carrier 2	0,872499	-0,672156
3129	CCD47_MOUSE	Ccdc47	Coiled-coil domain-containing protein 47	0,180993	0,0926632
3130	ODPB_MOUSE	Pdhb	Pyruvate dehydrogenase E1 component subunit beta, mitochondrial	0,483254	0,262476
3131	ACBD6_MOUSE	Acbd6	Acyl-CoA-binding domain-containing protein 6	1,20238	0,733465
3132	ARPIN_MOUSE	Arpin	Arpin	0,00640611	0,00295334
3133	SRSF9_MOUSE	Srsf9	Serine/arginine-rich splicing factor 9	0,0474382	-0,0360458
3134	TSTD3_MOUSE	Tstd3	Thiosulfate sulfurtransferase/rhodanese-like domain-containing protein 3	0,406874	-0,236328
3135	PBDC1_MOUSE	Pbdc1	Protein PBDC1	0,0618023	0,0357964
3136	TRM5_MOUSE	Trmt5	tRNA (guanine(37)-N1)-methyltransferase	0,133905	-0,097514
3137	PAPD1_MOUSE	Mtpap	Poly(A) RNA polymerase, mitochondrial	0,00407143	-0,00318871
3138	T2EA_MOUSE	Gtf2e1	General transcription factor IIE subunit 1	0,127469	0,0438919
3139	HNRPM_MOUSE	Hnrnpm	Heterogeneous nuclear ribonucleoprotein M	0,526587	0,378607
3140	LMAN1_MOUSE	Lman1	Protein ERGIC-53	1,5415	0,520318
3141	RFC5_MOUSE	Rfc5	Replication factor C subunit 5	0,835862	1,39429
3142	PGM1_MOUSE	Pgm1	Phosphoglucomutase-1	0,212143	-0,0703674
3143	RT30_MOUSE	Mrps30	28S ribosomal protein S30, mitochondrial	0,777004	-0,278422
3144	STX17_MOUSE	Stx17	Syntaxin-17	0,192478	-0,44768
3145	MRT4_MOUSE	Mrto4	mRNA turnover protein 4 homolog	0,276495	-0,187772
3146	SYRC_MOUSE	Rars	Arginine--tRNA ligase, cytoplasmic	1,50002	0,548258
3147	PTMS_MOUSE	Ptms	Parathymosin	1,05825	-0,505001
3148	SCOT1_MOUSE	Oxct1	Succinyl-CoA:3-ketoacid coenzyme A transferase 1, mitochondrial	0,695792	-0,432819
3149	MCES_MOUSE	Rnmt	mRNA cap guanine-N7 methyltransferase	1,01794	0,208901
3150	KPRA_MOUSE	Prpsap1	Phosphoribosyl pyrophosphate synthase-associated protein 1	1,53659	0,418174
3151	CY1_MOUSE	Cyc1	Cytochrome c1, heme protein, mitochondrial	0,150071	-0,120078
3152	DYL2_MOUSE	Dynll2	Dynein light chain 2, cytoplasmic	0,0802944	0,166827
3153	RM45_MOUSE	Mrpl45	39S ribosomal protein L45, mitochondrial	0,285335	0,171055
3154	SYTC_MOUSE	Tars	Threonine--tRNA ligase, cytoplasmic	0,2603	0,19886
3155	DDX56_MOUSE	Ddx56	Probable ATP-dependent RNA helicase DDX56	1,53076	1,08401
3156	HINT2_MOUSE	Hint2	Histidine triad nucleotide-binding protein 2, mitochondrial	0,581501	0,217753
3157	NH2L1_MOUSE	Snu13	NHP2-like protein 1	0,176194	0,0855991
3158	DUS12_MOUSE	Dusp12	Dual specificity protein phosphatase 12	0,681031	1,04797
3159	MTHFS_MOUSE	Mthfs	5-formyltetrahydrofolate cyclo-ligase	0,484219	-0,233985
3160	RT25_MOUSE	Mrps25	28S ribosomal protein S25, mitochondrial	0,917454	0,279792
3161	ILEUA_MOUSE	Serpinb1a	Leukocyte elastase inhibitor A	0,213106	-0,12339
3162	INT12_MOUSE	Ints12	Integrator complex subunit 12	1,00341	1,26795
3163	GAL3A_MOUSE	Gatd3a	Glutamine amidotransferase-like class 1 domain-containing protein 3A, mito	1,32828	0,372562
3164	CNDP2_MOUSE	Cndp2	Cytosolic non-specific dipeptidase	0,97157	0,704633
3165	RM28_MOUSE	Mrpl28	39S ribosomal protein L28, mitochondrial	0,693225	0,268923
3166	VPS28_MOUSE	Vps28	Vacuolar protein sorting-associated protein 28 homolog	0,0369639	0,0256382
3167	TMEDA_MOUSE	Tmed10	Transmembrane emp24 domain-containing protein 10	0,336741	0,209681

3168	TBCB_MOUSE	Tbcb	Tubulin-folding cofactor B	0,0433858	-0,0211899
3169	AKTS1_MOUSE	Akt1s1	Proline-rich AKT1 substrate 1	2,03457	0,442278
3170	RAB1B_MOUSE	Rab1b	Ras-related protein Rab-1B	0,392276	0,166418
3171	PMVK_MOUSE	Pmvk	Phosphomevalonate kinase	0,335214	0,591121
3172	NDUF4_MOUSE	Ndufaf4	NADH dehydrogenase [ubiquinone] 1 alpha subcomplex assembly factor 4	0,605716	0,363332
3173	RM53_MOUSE	Mrpl53	39S ribosomal protein L53, mitochondrial	0,494878	-0,256906
3174	MCEE_MOUSE	Mcee	Methylmalonyl-CoA epimerase, mitochondrial	0,206684	0,244992
3175	SARNP_MOUSE	Sarnp	SAP domain-containing ribonucleoprotein	1,09132	0,302116
3176	VATF_MOUSE	Atp6v1f	V-type proton ATPase subunit F	0,591597	0,422753
3177	CHCH2_MOUSE	Chchd2	Coiled-coil-helix-coiled-coil-helix domain-containing protein 2	0,229894	0,144785
3178	SEC13_MOUSE	Sec13	Protein SEC13 homolog	0,120723	-0,0578941
3179	MCA3_MOUSE	Eef1e1	Eukaryotic translation elongation factor 1 epsilon-1	0,100837	-0,114325
3180	FKB11_MOUSE	Fkbp11	Peptidyl-prolyl cis-trans isomerase FKBP11	0,184483	0,104688
3181	RM21_MOUSE	Mrpl21	39S ribosomal protein L21, mitochondrial	0,857643	0,300531
3182	RM13_MOUSE	Mrpl13	39S ribosomal protein L13, mitochondrial	0,86307	-0,19013
3183	CHRD1_MOUSE	Chordc1	Cysteine and histidine-rich domain-containing protein 1	1,381	0,659108
3184	MPH6_MOUSE	Mphosph6	M-phase phosphoprotein 6	0,438121	-0,388823
3185	ERP44_MOUSE	Erp44	Endoplasmic reticulum resident protein 44	1,66236	0,512495
3186	RL34_MOUSE	Rpl34	60S ribosomal protein L34	0,0616183	0,0219467
3187	MMAB_MOUSE	Mmab	Corrinoid adenosyltransferase	0,412079	-0,149883
3188	NXP20_MOUSE	Fam114a1	Protein Noxp20	1,94247	0,593818
3189	SPF27_MOUSE	Bcas2	Pre-mRNA-splicing factor SPF27	0,0584355	-0,0389454
3190	TOE1_MOUSE	Toe1	Target of EGR1 protein 1	0,635766	-0,562309
3191	ODO2_MOUSE	Dlst	Dihydrolipoyllysine-residue succinyltransferase component of 2-oxoglutarate	0,282693	0,132235
3192	UB2V2_MOUSE	Ube2v2	Ubiquitin-conjugating enzyme E2 variant 2	0,00191182	-0,00062027
3193	VP33A_MOUSE	Vps33a	Vacuolar protein sorting-associated protein 33A	1,16055	1,1039
3194	AACS_MOUSE	Aacs	Acetoacetyl-CoA synthetase	0,011989	-0,0265583
3195	COA3_MOUSE	Coa3	Cytochrome c oxidase assembly factor 3 homolog, mitochondrial	0,0714432	-0,0326885
3196	CORO7_MOUSE	Coro7	Coronin-7	0,479548	0,330099
3197	K1C20_MOUSE	Krt20	Keratin, type I cytoskeletal 20	1,6786	-0,915665
3198	RM19_MOUSE	Mrpl19	39S ribosomal protein L19, mitochondrial	0,384128	0,0966911
3199	PPAC_MOUSE	Acp1	Low molecular weight phosphotyrosine protein phosphatase	0,749074	0,852164
3200	HYEP_MOUSE	Ephx1	Epoxide hydrolase 1	1,63198	1,04018
3201	RUFY3_MOUSE	Rufy3	Protein RUFY3	0,95234	0,347061
3202	ATPD_MOUSE	Atp5f1d	ATP synthase subunit delta, mitochondrial	0,759583	1,02993
3203	STAG1_MOUSE	Stag1	Cohesin subunit SA-1	0,453631	-0,194468
3204	OXSM_MOUSE	Oxsm	3-oxoacyl-[acyl-carrier-protein] synthase, mitochondrial	0,573009	-0,357651
3205	EXOC2_MOUSE	Exoc2	Exocyst complex component 2	0,190681	0,0947956
3206	GCC1_MOUSE	Gcc1	GRIP and coiled-coil domain-containing protein 1	0,00549381	0,00347328
3207	CUL2_MOUSE	Cul2	Cullin-2	1,70075	0,362568
3208	RABL3_MOUSE	Rabl3	Rab-like protein 3	0,594496	0,151075
3209	SF3A3_MOUSE	Sf3a3	Splicing factor 3A subunit 3	0,316341	-0,118917
3210	SHPK_MOUSE	Shpk	Sedoheptulokinase	0,0546476	0,0310555
3211	WDR20_MOUSE	Wdr20	WD repeat-containing protein 20	0,540241	0,854868
3212	ATAD1_MOUSE	Atad1	ATPase family AAA domain-containing protein 1	0,19674	0,238572
3213	CUL5_MOUSE	Cul5	Cullin-5	0,342936	0,15665
3214	SYAP1_MOUSE	Syap1	Synapse-associated protein 1	0,707876	0,277859
3215	SC23B_MOUSE	Sec23b	Protein transport protein Sec23B	0,731507	0,434314
3216	D42E1_MOUSE	Sdr42e1	Short-chain dehydrogenase/reductase family 42E member 1	0,167105	0,226141
3217	NDUV2_MOUSE	Ndufv2	NADH dehydrogenase [ubiquinone] flavoprotein 2, mitochondrial	0,667985	-0,428911
3218	SYJ2B_MOUSE	Synj2bp	Synaptojanin-2-binding protein	0,323919	0,115182
3219	GHC1_MOUSE	Slc25a22	Mitochondrial glutamate carrier 1	0,0229642	-0,0261375

3220	NC2A_MOUSE	Drap1	Dr1-associated corepressor	0,0790139	-0,0309521
3221	IDH3A_MOUSE	Idh3a	Isocitrate dehydrogenase [NAD] subunit alpha, mitochondrial	0,157539	-0,130475
3222	NOSIP_MOUSE	Nosip	Nitric oxide synthase-interacting protein	0,481599	0,491455
3223	F162A_MOUSE	Fam162a	Protein FAM162A	0,90146	0,259869
3224	BORC6_MOUSE	Borcs6	BLOC-1-related complex subunit 6	0,571432	0,261064
3225	MSRA_MOUSE	Msra	Mitochondrial peptide methionine sulfoxide reductase	0,0524263	-0,00995598
3226	GLGB_MOUSE	Gbe1	1,4-alpha-glucan-branching enzyme	0,659656	0,436972
3227	ALKB7_MOUSE	Alkbh7	Alpha-ketoglutarate-dependent dioxygenase alkB homolog 7, mitochondrial	1,01811	0,673915
3228	NOP56_MOUSE	Nop56	Nucleolar protein 56	0,560396	0,139097
3229	TMX2_MOUSE	Tmx2	Thioredoxin-related transmembrane protein 2	0,181267	-0,12027
3230	PIR_MOUSE	Pir	Pirin	0,424365	0,283353
3231	CF226_MOUSE		Uncharacterized protein C6orf226 homolog	1,18066	0,524715
3232	TRIR_MOUSE	Trir	Telomerase RNA component interacting RNase	0,118481	-0,218983
3233	EXOS8_MOUSE	Exosc8	Exosome complex component RRP43	0,155796	-0,271322
3234	RM02_MOUSE	Mrpl2	39S ribosomal protein L2, mitochondrial	0,575327	0,276425
3235	PPIL2_MOUSE	Ppil2	RING-type E3 ubiquitin-protein ligase PPIL2	0,165046	0,179698
3236	SRP19_MOUSE	Srp19	Signal recognition particle 19 kDa protein	0,0415599	0,0453522
3237	ARMC1_MOUSE	Armc1	Armadillo repeat-containing protein 1	0,940566	0,473769
3238	ACAD8_MOUSE	Acad8	Isobutyryl-CoA dehydrogenase, mitochondrial	0,837672	0,138392
3239	OVCA2_MOUSE	Ovca2	Esterase OVCA2	0,640323	0,998449
3240	PRPS1_MOUSE	Prps1	Ribose-phosphate pyrophosphokinase 1	0,368891	-0,188517
3241	RTCA_MOUSE	RtcA	RNA 3'-terminal phosphate cyclase	0,0251332	0,031509
3242	LHPP_MOUSE	Lhpp	Phospholysine phosphohistidine inorganic pyrophosphate phosphatase	0,887452	-0,343359
3243	COX20_MOUSE	Cox20	Cytochrome c oxidase assembly protein COX20, mitochondrial	0,457334	0,342605
3244	ECHD3_MOUSE	Echdc3	Enoyl-CoA hydratase domain-containing protein 3, mitochondrial	1,15603	-0,402093
3245	RT09_MOUSE	Mrps9	28S ribosomal protein S9, mitochondrial	1,38856	0,482811
3246	RM30_MOUSE	Mrpl30	39S ribosomal protein L30, mitochondrial	0,0764264	-0,0404869
3247	APMAP_MOUSE	Apmap	Adipocyte plasma membrane-associated protein	0,17074	-0,154018
3248	ISCU_MOUSE	Iscu	Iron-sulfur cluster assembly enzyme ISCU, mitochondrial	0,381663	-0,503275
3249	RL22L_MOUSE	Rpl22l1	60S ribosomal protein L22-like 1	0,506081	-0,471292
3250	MED8_MOUSE	Med8	Mediator of RNA polymerase II transcription subunit 8	1,11442	0,996725
3251	GGCT_MOUSE	Ggct	Gamma-glutamylcyclotransferase	0,20041	0,105127
3252	IPYR_MOUSE	Ppa1	Inorganic pyrophosphatase	0,806809	0,234488
3253	PRXD1_MOUSE	Prorsd1	Prolyl-tRNA synthetase associated domain-containing protein 1	1,79708	0,532334
3254	RL37_MOUSE	Rpl37	60S ribosomal protein L37	1,20645	0,563623
3255	SOX_MOUSE	Pipox	Peroxisomal sarcosine oxidase	0,77408	0,30884
3256	DNJB4_MOUSE	Dnajb4	DnaJ homolog subfamily B member 4	0,824246	0,430097
3257	QCR7_MOUSE	Uqcrb	Cytochrome b-c1 complex subunit 7	0,758896	0,573188
3258	PPIH_MOUSE	Ppih	Peptidyl-prolyl cis-trans isomerase H	1,40337	0,457702
3259	TIM50_MOUSE	Timm50	Mitochondrial import inner membrane translocase subunit TIM50	1,13175	-0,399822
3260	U2AF1_MOUSE	U2af1	Splicing factor U2AF 35 kDa subunit	0,490573	0,842188
3261	ITPA_MOUSE	Itpa	Inosine triphosphate pyrophosphatase	0,520604	0,276025
3262	ARP5L_MOUSE	Arpc5l	Actin-related protein 2/3 complex subunit 5-like protein	0,665641	0,3855
3263	CHM4B_MOUSE	Chmp4b	Charged multivesicular body protein 4b	0,183679	0,161582
3264	NDUAB_MOUSE	Ndufa11	NADH dehydrogenase [ubiquinone] 1 alpha subcomplex subunit 11	0,0142919	0,0100376
3265	IN35_MOUSE	Ifi35	Interferon-induced 35 kDa protein homolog	0,609251	0,642086
3266	RL4_MOUSE	Rpl4	60S ribosomal protein L4	0,941883	0,731295
3267	EF1G_MOUSE	Eef1g	Elongation factor 1-gamma	0,469175	0,240144
3268	RM17_MOUSE	Mrpl17	39S ribosomal protein L17, mitochondrial	0,805341	0,472342
3269	ARFG3_MOUSE	Arfgap3	ADP-ribosylation factor GTPase-activating protein 3	0,460076	0,589906
3270	ORN_MOUSE	Rexo2	Oligoribonuclease, mitochondrial	1,10186	-0,504099
3271	BOLA1_MOUSE	Bola1	BolA-like protein 1	0,93963	0,315418

3272	GSDMD_MOUSE	Gsdmdc1	Gasdermin-D	0,280414	-0,120295
3273	SLIRP_MOUSE	Slirp	SRA stem-loop-interacting RNA-binding protein, mitochondrial	0,28239	0,137616
3274	SNX5_MOUSE	Snx5	Sorting nexin-5	0,189207	0,111529
3275	PSD12_MOUSE	Psmd12	26S proteasome non-ATPase regulatory subunit 12	0,288493	-0,274626
3276	OCAD2_MOUSE	Ociad2	OCIA domain-containing protein 2	1,48509	0,585347
3277	CUTC_MOUSE	Cutc	Copper homeostasis protein cutC homolog	1,28957	0,901056
3278	CC124_MOUSE	Ccdc124	Coiled-coil domain-containing protein 124	1,12926	0,675018
3279	EFHD2_MOUSE	Efhd2	EF-hand domain-containing protein D2	0,148721	-0,0950146
3280	ASCC1_MOUSE	Ascc1	Activating signal cointegrator 1 complex subunit 1	1,55343	-0,932485
3281	ATGA1_MOUSE	Atg101	Autophagy-related protein 101	1,70475	0,710223
3282	ATG7_MOUSE	Atg7	Ubiquitin-like modifier-activating enzyme ATG7	0,600527	0,274026
3283	ST1C2_MOUSE	Sult1c2	Sulfotransferase 1C2	0,211199	0,139217
3284	GATM_MOUSE	Gatm	Glycine amidinotransferase, mitochondrial	1,33214	0,28775
3285	ECHD1_MOUSE	Echdc1	Ethylmalonyl-CoA decarboxylase	0,156748	0,148378
3286	DDI1_MOUSE	Ddi1	Protein DDI1 homolog 1	0,64122	0,276029
3287	PHP14_MOUSE	Phpt1	14 kDa phosphohistidine phosphatase	0,537736	-0,260129
3288	DCPS_MOUSE	Dcps	m7GpppX diphosphatase	0,967179	-0,278422
3289	GBG12_MOUSE	Gng12	Guanine nucleotide-binding protein G(I)/G(S)/G(O) subunit gamma-12	2,02297	0,425973
3290	MTU1_MOUSE	Trmu	Mitochondrial tRNA-specific 2-thiouridylase 1	0,0202787	-0,0101307
3291	CNPY3_MOUSE	Cnpy3	Protein canopy homolog 3	1,56382	1,15258
3292	PRP4_MOUSE	Prpf4	U4/U6 small nuclear ribonucleoprotein Prp4	1,57105	0,492279
3293	CNN3_MOUSE	Cnn3	Calponin-3	0,241031	0,121421
3294	ANCHR_MOUSE	Zfyve19	Abscission/NoCut checkpoint regulator	1,08321	0,762745
3295	SNAA_MOUSE	Napa	Alpha-soluble NSF attachment protein	1,6511	0,467672
3296	RM12_MOUSE	Mrpl12	39S ribosomal protein L12, mitochondrial	0,308659	-0,107967
3297	CB39L_MOUSE	Cab39l	Calcium-binding protein 39-like	0,575027	-0,531552
3298	ATPO_MOUSE	Atp5po	ATP synthase subunit O, mitochondrial	0,0537801	0,0321728
3299	PHYD1_MOUSE	Phyhd1	Phytanoyl-CoA dioxygenase domain-containing protein 1	0,248633	0,401542
3300	MCTS1_MOUSE	Mcts1	Malignant T-cell-amplified sequence 1	1,34563	0,426051
3301	IAH1_MOUSE	Iah1	Isoamyl acetate-hydrolyzing esterase 1 homolog	0,635643	0,555993
3302	PHKG2_MOUSE	Phkg2	Phosphorylase b kinase gamma catalytic chain, liver/testis isoform	0,504789	0,271702
3303	CHM2A_MOUSE	Chmp2a	Charged multivesicular body protein 2a	1,1326	0,473997
3304	ZFPL1_MOUSE	Zfpl1	Zinc finger protein-like 1	0,827017	0,377651
3305	PXL2B_MOUSE	Prxl2b	Prostamide/prostaglandin F synthase	1,10855	0,714678
3306	QCR2_MOUSE	Uqcrc2	Cytochrome b-c1 complex subunit 2, mitochondrial	0,374622	-0,216872
3307	TYSD1_MOUSE	Tysnd1	Peroxisomal leader peptide-processing protease	1,29341	0,792881
3308	HUTI_MOUSE	Amdhd1	Probable imidazolonepropionase	0,010475	0,0061985
3309	DHDH_MOUSE	Dhdh	Trans-1,2-dihydrobenzene-1,2-diol dehydrogenase	0,448567	-0,367395
3310	CPN2_MOUSE	Cpn2	Carboxypeptidase N subunit 2	0,13636	0,0873016
3311	SELO_MOUSE	Selenoo	Protein adenylyltransferase SelO, mitochondrial	0,270687	-0,294241
3312	CMTR1_MOUSE	Cmtr1	Cap-specific mRNA (nucleoside-2'-O-)-methyltransferase 1	0,328585	0,241382
3313	KAP0_MOUSE	Prkar1a	cAMP-dependent protein kinase type I-alpha regulatory subunit	1,48594	0,687729
3314	ICA_MOUSE	Ica	Inhibitor of carbonic anhydrase	1,38128	0,39068
3315	PELP1_MOUSE	Pelp1	Proline-, glutamic acid- and leucine-rich protein 1	0,157045	-0,189741
3316	ALG2_MOUSE	Alg2	Alpha-1,3/1,6-mannosyltransferase ALG2	1,42537	0,405068
3317	SPB1_MOUSE	Ftsj3	pre-rRNA 2'-O-ribose RNA methyltransferase FTSJ3	1,34311	0,677904
3318	AL7A1_MOUSE	Aldh7a1	Alpha-aminoadipic semialdehyde dehydrogenase	0,678962	-0,258653
3319	CP27A_MOUSE	Cyp27a1	Sterol 26-hydroxylase, mitochondrial	0,0226128	0,0404713
3320	AP2B1_MOUSE	Ap2b1	AP-2 complex subunit beta	0,164537	0,138807
3321	PLIN3_MOUSE	Plin3	Perilipin-3	0,255267	0,201094
3322	RPN2_MOUSE	Rpn2	Dolichyl-diphosphooligosaccharide--protein glycosyltransferase subunit 2	0,233995	-0,143409
3323	SRPRA_MOUSE	Srpra	Signal recognition particle receptor subunit alpha	0,432225	0,182316

3324	WWP2_MOUSE	Wwp2	NEDD4-like E3 ubiquitin-protein ligase WWP2	0,219676	0,121574
3325	LMAN2_MOUSE	Lman2	Vesicular integral-membrane protein VIP36	0,296132	-0,370378
3326	TMPS6_MOUSE	Tmprss6	Transmembrane protease serine 6	0,951812	0,943768
3327	PGAM1_MOUSE	Pgam1	Phosphoglycerate mutase 1	0,363255	-0,11555
3328	BI2L1_MOUSE	Baiap2l1	Brain-specific angiogenesis inhibitor 1-associated protein 2-like protein 1	0,502342	0,193566
3329	ACO12_MOUSE	Acot12	Acetyl-coenzyme A thioesterase	0,362026	0,307811
3330	ACDSB_MOUSE	Acadsb	Short/branched chain specific acyl-CoA dehydrogenase, mitochondrial	0,0213172	-0,0107857
3331	GDAP2_MOUSE	Gdap2	Ganglioside-induced differentiation-associated protein 2	0,109626	0,0652554
3332	COASY_MOUSE	Coasy	Bifunctional coenzyme A synthase	1,31178	0,273847
3333	ABCG8_MOUSE	Abcg8	ATP-binding cassette sub-family G member 8	0,570163	-0,381804
3334	ECHP_MOUSE	Ehhadh	Peroxisomal bifunctional enzyme	1,58338	1,25259
3335	LONP2_MOUSE	Lonp2	Lon protease homolog 2, peroxisomal	2,1776	0,745652
3336	KCY_MOUSE	Cmpk1	UMP-CMP kinase	0,0740153	0,045985
3337	AKAP8_MOUSE	Akap8	A-kinase anchor protein 8	0,0600543	0,0593472
3338	XRN2_MOUSE	Xrn2	5'-3' exoribonuclease 2	0,546032	-0,190981
3339	MYPT1_MOUSE	Ppp1r12a	Protein phosphatase 1 regulatory subunit 12A	0,577748	0,353749
3340	TMM43_MOUSE	Tmem43	Transmembrane protein 43	0,659573	0,55016
3341	TPRGL_MOUSE	Tprg1l	Tumor protein p63-regulated gene 1-like protein	0,415924	0,238266
3342	KLC4_MOUSE	Klc4	Kinesin light chain 4	0,146728	0,0454594
3343	AMPD2_MOUSE	Ampd2	AMP deaminase 2	0,785469	0,54917
3344	M2GD_MOUSE	Dmgdh	Dimethylglycine dehydrogenase, mitochondrial	0,461684	0,290887
3345	CP4V2_MOUSE	Cyp4v2	Cytochrome P450 4V2	0,870051	0,473322
3346	PHLP_MOUSE	Pdcl	Phosducin-like protein	0,51627	-1,05952
3347	SYVN1_MOUSE	Syvn1	E3 ubiquitin-protein ligase synoviolin	0,851125	0,764727
3348	NVL_MOUSE	Nvl	Nuclear valosin-containing protein-like	0,955596	0,449198
3349	IKIP_MOUSE	Ikbip	Inhibitor of nuclear factor kappa-B kinase-interacting protein	0,79827	0,729555
3350	ERGI1_MOUSE	Ergic1	Endoplasmic reticulum-Golgi intermediate compartment protein 1	0,327219	0,0867889
3351	DJC10_MOUSE	Dnajc10	DnaJ homolog subfamily C member 10	0,920284	0,630971
3352	KC1D_MOUSE	Csnk1d	Casein kinase I isoform delta	0,179284	0,147754
3353	PRP17_MOUSE	Cdc40	Pre-mRNA-processing factor 17	1,31966	0,371184
3354	OCTC_MOUSE	Crot	Peroxisomal carnitine O-octanoyltransferase	0,668793	0,536782
3355	GNAI3_MOUSE	Gnai3	Guanine nucleotide-binding protein G(i) subunit alpha	0,135675	0,087941
3356	MPPA_MOUSE	Pmpca	Mitochondrial-processing peptidase subunit alpha	0,661784	0,292402
3357	NDUA9_MOUSE	Ndufa9	NADH dehydrogenase [ubiquinone] 1 alpha subcomplex subunit 9, mitochor	0,307429	-0,132618
3358	NDUS7_MOUSE	Ndufs7	NADH dehydrogenase [ubiquinone] iron-sulfur protein 7, mitochondrial	0,141413	-0,0999138
3359	RT15_MOUSE	Mrps15	28S ribosomal protein S15, mitochondrial	1,263	0,472221
3360	RT11_MOUSE	Mrps11	28S ribosomal protein S11, mitochondrial	0,405627	0,425813
3361	ISCA2_MOUSE	Isca2	Iron-sulfur cluster assembly 2 homolog, mitochondrial	0,241536	0,155876
3362	P5CR3_MOUSE	Pycr3	Pyrroline-5-carboxylate reductase 3	1,51737	0,638118
3363	ISC2B_MOUSE	Isoc2b	Isochorismatase domain-containing protein 2B	0,288252	0,225966
3364	TOM20_MOUSE	Tomm20	Mitochondrial import receptor subunit TOM20 homolog	0,874505	-1,19126
3365	6PGD_MOUSE	Pgd	6-phosphogluconate dehydrogenase, decarboxylating	0,68327	0,328265
3366	SYF1_MOUSE	Xab2	Pre-mRNA-splicing factor SYF1	0,24176	0,112762
3367	PK1IP_MOUSE	Pak1ip1	p21-activated protein kinase-interacting protein 1	0,140117	0,102641
3368	SSRG_MOUSE	Ssr3	Translocon-associated protein subunit gamma	0,0433394	0,0358173
3369	PBLD1_MOUSE	Pbld1	Phenazine biosynthesis-like domain-containing protein 1	0,657477	0,485455
3370	TR112_MOUSE	Trmt112	Multifunctional methyltransferase subunit TRM112-like protein	0,0807215	-0,0422596
3371	EIF3F_MOUSE	Eif3f	Eukaryotic translation initiation factor 3 subunit F	0,149215	0,109229
3372	LST8_MOUSE	Mlst8	Target of rapamycin complex subunit LST8	1,57853	0,442486
3373	NDUA8_MOUSE	Ndufa8	NADH dehydrogenase [ubiquinone] 1 alpha subcomplex subunit 8	0,176108	-0,104821
3374	NPL_MOUSE	Npl	N-acetylneuraminate lyase	0,445406	-0,215714
3375	MET15_MOUSE	Mettl15	Probable methyltransferase-like protein 15	0,211203	0,198206

3376	PUR6_MOUSE	Paics	Multifunctional protein ADE2	0,93066	-0,369893
3377	ETHE1_MOUSE	Ethe1	Persulfide dioxygenase ETHE1, mitochondrial	0,499834	-0,169765
3378	GSTK1_MOUSE	Gstk1	Glutathione S-transferase kappa 1	0,0932969	-0,08708
3379	NUD12_MOUSE	Nudt12	Peroxisomal NADH pyrophosphatase NUDT12	2,14497	0,469017
3380	NB5R3_MOUSE	Cyb5r3	NADH-cytochrome b5 reductase 3	0,573781	0,415266
3381	ASPD_MOUSE	Aspdh	Putative L-aspartate dehydrogenase	0,88294	0,391544
3382	MTL26_MOUSE	Mettl26	Methyltransferase-like 26	0,345282	0,226736
3383	MECR_MOUSE	Mecr	Enoyl-[acyl-carrier-protein] reductase, mitochondrial	0,482509	0,131243
3384	NDUBA_MOUSE	Ndufb10	NADH dehydrogenase [ubiquinone] 1 beta subcomplex subunit 10	0,0726943	-0,0484322
3385	AKCL2_MOUSE	Akr1e2	1,5-anhydro-D-fructose reductase	0,955496	-0,277024
3386	NDUS3_MOUSE	Ndufs3	NADH dehydrogenase [ubiquinone] iron-sulfur protein 3, mitochondrial	0,160159	-0,229047
3387	SDF2_MOUSE	Sdf2	Stromal cell-derived factor 2	0,43714	-0,96811
3388	CRIP2_MOUSE	Crip2	Cysteine-rich protein 2	0,287642	-0,202648
3389	RM04_MOUSE	Mrpl4	39S ribosomal protein L4, mitochondrial	0,208366	-0,118393
3390	HOGA1_MOUSE	Hoga1	4-hydroxy-2-oxoglutarate aldolase, mitochondrial	0,30332	0,165727
3391	DGAT2_MOUSE	Dgat2	Diacylglycerol O-acyltransferase 2	0,0646165	-0,0551239
3392	RMD1_MOUSE	Rmdn1	Regulator of microtubule dynamics protein 1	0,201123	0,1353
3393	ETFB_MOUSE	Etfb	Electron transfer flavoprotein subunit beta	1,0949	0,231752
3394	ATP5H_MOUSE	Atp5pd	ATP synthase subunit d, mitochondrial	0,227455	0,163927
3395	IYD1_MOUSE	Iyd	Iodotyrosine deiodinase 1	0,23763	0,0776756
3396	KEG1_MOUSE	Keg1	Glycine N-acyltransferase-like protein Keg1	0,146435	-0,088488
3397	MIC26_MOUSE	Apoo	MICOS complex subunit Mic26	0,0216661	0,0262684
3398	RARR2_MOUSE	Rarres2	Retinoic acid receptor responder protein 2	1,34783	0,379259
3399	MET7B_MOUSE	Mettl7b	Methyltransferase-like protein 7B	2,15112	0,739414
3400	SAC1_MOUSE	Sacm1l	Phosphatidylinositide phosphatase SAC1	0,61706	0,182521
3401	RAI14_MOUSE	Rai14	Ankycorbin	0,0381603	0,026453
3402	EMC7_MOUSE	Emc7	ER membrane protein complex subunit 7	0,157702	0,0601303
3403	CP4FE_MOUSE	Cyp4f14	Leukotriene-B4 omega-hydroxylase 3	0,0218927	0,0215343
3404	LACTB_MOUSE	Lactb	Serine beta-lactamase-like protein LACTB, mitochondrial	0,554812	0,653903
3405	NMNA1_MOUSE	Nmnat1	Nicotinamide/nicotinic acid mononucleotide adenylyltransferase 1	0,339347	0,616962
3406	ASC_MOUSE	Pycard	Apoptosis-associated speck-like protein containing a CARD	0,134181	-0,114148
3407	SERHL_MOUSE	Serhl	Serine hydrolase-like protein	0,143382	-0,105812
3408	PARVA_MOUSE	Parva	Alpha-parvin	0,230459	-0,094413
3409	AT131_MOUSE	Atp13a1	Manganese-transporting ATPase 13A1	1,31066	0,23872
3410	ARFG1_MOUSE	Arfgap1	ADP-ribosylation factor GTPase-activating protein 1	0,190251	0,148255
3411	SIL1_MOUSE	Sil1	Nucleotide exchange factor SIL1	0,237293	-0,233152
3412	XPO7_MOUSE	Xpo7	Exportin-7	0,00545379	0,00358734
3413	IPO7_MOUSE	Ipo7	Importin-7	0,296412	-0,296157
3414	STAR5_MOUSE	Stard5	StAR-related lipid transfer protein 5	0,951617	1,82236
3415	RENT1_MOUSE	Upf1	Regulator of nonsense transcripts 1	1,10713	0,308854
3416	CPSF1_MOUSE	Cpsf1	Cleavage and polyadenylation specificity factor subunit 1	0,0673355	-0,0374424
3417	UBL5_MOUSE	Ubl5	Ubiquitin-like protein 5	0,063212	-0,0643967
3418	DHB11_MOUSE	Hsd17b11	Estradiol 17-beta-dehydrogenase 11	0,297403	0,263759
3419	MMSA_MOUSE	Aldh6a1	Methylmalonate-semialdehyde dehydrogenase [acylating], mitochondrial	0,0139305	-0,00743065
3420	BCAP_MOUSE	Pik3ap1	Phosphoinositide 3-kinase adapter protein 1	0,533958	0,279132
3421	PESC_MOUSE	Pes1	Pescadillo homolog	0,361688	0,107467
3422	3BHS7_MOUSE	Hsd3b7	3 beta-hydroxysteroid dehydrogenase type 7	0,470348	-0,784278
3423	SCYL1_MOUSE	Scyl1	N-terminal kinase-like protein	0,0799518	0,103183
3424	DPYS_MOUSE	Dpys	Dihydropyrimidinase	0,371633	0,163741
3425	C43BP_MOUSE	Col4a3bp	Collagen type IV alpha-3-binding protein	0,0978873	0,0517319
3426	ERAP1_MOUSE	Erap1	Endoplasmic reticulum aminopeptidase 1	0,0472052	0,0223763
3427	VPS35_MOUSE	Vps35	Vacuolar protein sorting-associated protein 35	0,612226	-0,283379

3428	RM46_MOUSE	Mrpl46	39S ribosomal protein L46, mitochondrial	0,236875	-0,0914696
3429	MAGI3_MOUSE	Magi3	Membrane-associated guanylate kinase, WW and PDZ domain-containing p	0,528624	-0,581143
3430	MVP_MOUSE	Mvp	Major vault protein	1,53739	0,729047
3431	EHD4_MOUSE	Ehd4	EH domain-containing protein 4	0,859095	0,498403
3432	OGA_MOUSE	Oga	Protein O-GlcNAcase	0,0952587	-0,0716949
3433	MYCBP_MOUSE	Mycbp	c-Myc-binding protein	0,672384	0,38535
3434	SET_MOUSE	Set	Protein SET	0,356348	0,159284
3435	KI13A_MOUSE	Kif13a	Kinesin-like protein KIF13A	0,238597	0,170543
3436	RPGF4_MOUSE	Rapgef4	Rap guanine nucleotide exchange factor 4	0,921181	-0,462106
3437	STX12_MOUSE	Stx12	Syntaxin-12	0,197444	0,170055
3438	TOR1A_MOUSE	Tor1a	Torsin-1A	0,145641	-0,0877811
3439	TOR1B_MOUSE	Tor1b	Torsin-1B	0,321828	0,364919
3440	FL2D_MOUSE	Wtap	Pre-mRNA-splicing regulator WTAP	0,577247	0,21921
3441	SYCC_MOUSE	Cars	Cysteine--tRNA ligase, cytoplasmic	2,89871	0,687124
3442	ELP4_MOUSE	Elp4	Elongator complex protein 4	0,844159	0,408858
3443	RT29_MOUSE	Dap3	28S ribosomal protein S29, mitochondrial	0,22493	-0,112862
3444	TFP11_MOUSE	Tfip11	Tuftelin-interacting protein 11	0,69619	-0,894053
3445	SNP29_MOUSE	Snap29	Synaptosomal-associated protein 29	1,28179	0,457086
3446	MESD_MOUSE	Mesd	LRP chaperone MESD	0,447232	0,270618
3447	LIMA1_MOUSE	Lima1	LIM domain and actin-binding protein 1	0,219506	0,170894
3448	RDH14_MOUSE	Rdh14	Retinol dehydrogenase 14	0,601829	0,208233
3449	XPO2_MOUSE	Cse1l	Exportin-2	0,00069973	0,000373459
3450	GCYA1_MOUSE	Gucy1a1	Guanylate cyclase soluble subunit alpha-1	0,409831	-0,21497
3451	SEP15_MOUSE	Selenof	Selenoprotein F	0,586212	0,490669
3452	NDUAD_MOUSE	Ndufa13	NADH dehydrogenase [ubiquinone] 1 alpha subcomplex subunit 13	0,075061	0,0418331
3453	RBP2_MOUSE	Ranbp2	E3 SUMO-protein ligase RanBP2	0,144404	-0,0578262
3454	UBE4B_MOUSE	Ube4b	Ubiquitin conjugation factor E4 B	0,0489936	-0,131072
3455	ARHG7_MOUSE	Arhgef7	Rho guanine nucleotide exchange factor 7	0,279807	0,0961754
3456	SHIP1_MOUSE	Inpp5d	Phosphatidylinositol 3,4,5-trisphosphate 5-phosphatase 1	0,83282	0,839963
3457	NEK7_MOUSE	Nek7	Serine/threonine-protein kinase Nek7	2,20327	0,599974
3458	HRG_MOUSE	Hrg	Histidine-rich glycoprotein	0,150989	0,160001
3459	DYSF_MOUSE	Dysf	Dysferlin	0,66316	0,410694
3460	LRBA_MOUSE	Lrba	Lipopolysaccharide-responsive and beige-like anchor protein	0,156885	0,0869949
3461	SDF2L_MOUSE	Sdf2l1	Stromal cell-derived factor 2-like protein 1	1,72384	0,611328
3462	PSMG2_MOUSE	Psmg2	Proteasome assembly chaperone 2	1,34187	-0,500664
3463	AN32B_MOUSE	Anp32b	Acidic leucine-rich nuclear phosphoprotein 32 family member B	0,795828	2,24936
3464	BRD4_MOUSE	Brd4	Bromodomain-containing protein 4	0,681837	0,348977
3465	DDX24_MOUSE	Ddx24	ATP-dependent RNA helicase DDX24	0,435186	-0,308675
3466	AGK_MOUSE	Agk	Acylglycerol kinase, mitochondrial	0,592873	-0,654194
3467	PGPI_MOUSE	Pgpep1	Pyroglutamyl-peptidase 1	0,381592	-0,26106
3468	DKC1_MOUSE	Dkc1	H/ACA ribonucleoprotein complex subunit DKC1	0,367775	0,139245
3469	GTF2I_MOUSE	Gtf2i	General transcription factor II-I	0,831624	0,394237
3470	PYGL_MOUSE	Pygl	Glycogen phosphorylase, liver form	0,294819	-0,179134
3471	DPP2_MOUSE	Dpp7	Dipeptidyl peptidase 2	1,85085	0,742417
3472	RN114_MOUSE	Rnf114	E3 ubiquitin-protein ligase RNF114	0,763338	0,661261
3473	PALLD_MOUSE	Palld	Palladin	1,9291	0,71303
3474	CBPB2_MOUSE	Cpb2	Carboxypeptidase B2	0,0846888	0,0720917
3475	IVD_MOUSE	Ivd	Isovaleryl-CoA dehydrogenase, mitochondrial	0,0155227	-0,0198994
3476	TMOD3_MOUSE	Tmod3	Tropomodulin-3	1,32624	0,394094
3477	PGTA_MOUSE	Rabggta	Geranylgeranyl transferase type-2 subunit alpha	2,17157	0,700645
3478	NHRF2_MOUSE	Slc9a3r2	Na(+)/H(+) exchange regulatory cofactor NHE-RF2	1,49006	0,644034
3479	IDE_MOUSE	Ide	Insulin-degrading enzyme	0,109528	-0,0994186

3480	CLPX_MOUSE	Clpx	ATP-dependent Clp protease ATP-binding subunit clpX-like, mitochondrial	2,18049	0,480718
3481	CWC15_MOUSE	Cwc15	Spliceosome-associated protein CWC15 homolog	0,351294	-0,319922
3482	PALMD_MOUSE	Palmd	Palmdelphin	0,802525	0,581081
3483	DYHC1_MOUSE	Dync1h1	Cytoplasmic dynein 1 heavy chain 1	2,06019	0,448226
3484	INO1_MOUSE	Isyna1	Inositol-3-phosphate synthase 1	1,09215	0,511116
3485	NIT2_MOUSE	Nit2	Omega-amidase NIT2	0,618297	0,420358
3486	SELB_MOUSE	Eefsec	Selenocysteine-specific elongation factor	0,0712565	-0,063533
3487	STK3_MOUSE	Stk3	Serine/threonine-protein kinase 3	0,320388	0,255798
3488	ABCBA_MOUSE	Abcb10	ATP-binding cassette sub-family B member 10, mitochondrial	1,24295	0,490829
3489	NQO2_MOUSE	Nqo2	Ribosyldihydronicotinamide dehydrogenase [quinone]	0,0413149	0,0325157
3490	NGLY1_MOUSE	Ngly1	Peptide-N(4)-(N-acetyl-beta-glucosaminyl)asparagine amidase	0,488518	0,498256
3491	SPHK2_MOUSE	Sphk2	Sphingosine kinase 2	0,311531	0,315557
3492	COPB_MOUSE	Copb1	Coatomer subunit beta	0,200314	-0,223203
3493	CCD22_MOUSE	Ccdc22	Coiled-coil domain-containing protein 22	0,607973	0,270214
3494	NUP50_MOUSE	Nup50	Nuclear pore complex protein Nup50	0,474624	0,273248
3495	AK1A1_MOUSE	Akr1a1	Aldo-keto reductase family 1 member A1	0,242267	0,136329
3496	DDX21_MOUSE	Ddx21	Nucleolar RNA helicase 2	1,50764	0,494812
3497	RT34_MOUSE	Mrps34	28S ribosomal protein S34, mitochondrial	0,941336	0,260756
3498	NHRF3_MOUSE	Pdzk1	Na(+)/H(+) exchange regulatory cofactor NHE-RF3	0,659027	0,496889
3499	RALB_MOUSE	Ralb	Ras-related protein Ral-B	0,528798	-0,258507
3500	ENY2_MOUSE	Eny2	Transcription and mRNA export factor ENY2	0,579308	0,279481
3501	ACINU_MOUSE	Acin1	Apoptotic chromatin condensation inducer in the nucleus	0,0728633	0,0303104
3502	HTRA2_MOUSE	Htra2	Serine protease HTRA2, mitochondrial	0,805087	0,65816
3503	CMLO1_MOUSE	Cml1	Probable N-acetyltransferase CML1	0,0118185	0,0192696
3504	FLII_MOUSE	Flii	Protein flightless-1 homolog	0,227742	0,0545494
3505	RPF2_MOUSE	Rpf2	Ribosome production factor 2 homolog	0,128685	-0,0454281
3506	WDR12_MOUSE	Wdr12	Ribosome biogenesis protein WDR12	0,429088	0,281584
3507	RIOX1_MOUSE	Riox1	Ribosomal oxygenase 1	0,216011	0,107052
3508	RNAS4_MOUSE	Rnase4	Ribonuclease 4	0,0577409	-0,0643097
3509	RL38_MOUSE	Rpl38	60S ribosomal protein L38	0,405373	-0,433167
3510	LANC2_MOUSE	Lancl2	LanC-like protein 2	0,201409	-0,465059
3511	SYSM_MOUSE	Sars2	Serine--tRNA ligase, mitochondrial	1,37924	0,437946
3512	CBPN_MOUSE	Cpn1	Carboxypeptidase N catalytic chain	1,70126	0,662394
3513	SH3L1_MOUSE	Sh3bgrl	SH3 domain-binding glutamic acid-rich-like protein	0,14362	-0,103491
3514	PROF2_MOUSE	Pfn2	Profilin-2	0,380968	0,247778
3515	P2RX4_MOUSE	P2rx4	P2X purinoceptor 4	0,574056	0,259809
3516	PSMG1_MOUSE	Psmg1	Proteasome assembly chaperone 1	0,205811	0,103117
3517	GNA1_MOUSE	Gnpnat1	Glucosamine 6-phosphate N-acetyltransferase	0,325913	-0,158993
3518	PDK2_MOUSE	Pdk2	[Pyruvate dehydrogenase (acetyl-transferring)] kinase isozyme 2, mitochonc	0,446553	-0,377381
3519	SHLB1_MOUSE	Sh3glb1	Endophilin-B1	0,486607	0,35005
3520	PRELP_MOUSE	Prelp	Prolargin	0,62358	0,234159
3521	MYG1_MOUSE	Myg1	UPF0160 protein MYG1, mitochondrial	1,31097	0,421009
3522	HSPB8_MOUSE	Hspb8	Heat shock protein beta-8	0,938896	0,579964
3523	UCHL3_MOUSE	Uchl3	Ubiquitin carboxyl-terminal hydrolase isozyme L3	0,628911	0,523218
3524	AP3M1_MOUSE	Ap3m1	AP-3 complex subunit mu-1	0,194828	-0,0764469
3525	IQGA1_MOUSE	Iqgap1	Ras GTPase-activating-like protein IQGAP1	0,390248	-0,294669
3526	RM39_MOUSE	Mrpl39	39S ribosomal protein L39, mitochondrial	0,201281	0,116987
3527	CP39A_MOUSE	Cyp39a1	24-hydroxycholesterol 7-alpha-hydroxylase	0,30088	0,192792
3528	NDUF3_MOUSE	Ndufaf3	NADH dehydrogenase [ubiquinone] 1 alpha subcomplex assembly factor 3	0,633719	0,371091
3529	MBNL1_MOUSE	Mbnl1	Muscleblind-like protein 1	0,369936	0,3098
3530	HYOU1_MOUSE	Hyou1	Hypoxia up-regulated protein 1	2,99603	0,717128
3531	ADRM1_MOUSE	Adrm1	Proteasomal ubiquitin receptor ADRM1	0,0049472	0,00276375

3532	TFR2_MOUSE	Tfr2	Transferrin receptor protein 2	0,273123	0,190767
3533	AATF_MOUSE	Aatf	Protein AATF	0,98087	0,432796
3534	NUDT5_MOUSE	Nudt5	ADP-sugar pyrophosphatase	2,20214	0,387336
3535	HIP1R_MOUSE	Hip1r	Huntingtin-interacting protein 1-related protein	1,44783	0,488779
3536	LEG8_MOUSE	Lgals8	Galectin-8	2,56276	0,87243
3537	ISG20_MOUSE	Isg20	Interferon-stimulated gene 20 kDa protein	1,05301	0,629499
3538	HMGN5_MOUSE	Hmgn5	High mobility group nucleosome-binding domain-containing protein 5	0,136937	-0,05839
3539	MPP6_MOUSE	Mpp6	MAGUK p55 subfamily member 6	1,49002	0,638602
3540	MSRB1_MOUSE	Msrb1	Methionine-R-sulfoxide reductase B1	1,38695	-0,891159
3541	TGM1_MOUSE	Tgm1	Protein-glutamine gamma-glutamyltransferase K	0,493815	0,377078
3542	SCLY_MOUSE	Scly	Selenocysteine lyase	0,0846679	-0,0356136
3543	SART3_MOUSE	Sart3	Squamous cell carcinoma antigen recognized by T-cells 3	0,666778	0,419762
3544	AL9A1_MOUSE	Aldh9a1	4-trimethylaminobutyraldehyde dehydrogenase	1,15339	0,590831
3545	GRB14_MOUSE	Grb14	Growth factor receptor-bound protein 14	1,26517	0,429961
3546	MTOR_MOUSE	Mtor	Serine/threonine-protein kinase mTOR	0,546292	-0,357514
3547	CD2AP_MOUSE	Cd2ap	CD2-associated protein	0,788868	0,478403
3548	TRXR2_MOUSE	Txnrd2	Thioredoxin reductase 2, mitochondrial	0,0511877	0,0293552
3549	BAG3_MOUSE	Bag3	BAG family molecular chaperone regulator 3	0,610138	0,220322
3550	CUL3_MOUSE	Cul3	Cullin-3	0,0704336	0,05994
3551	PNKP_MOUSE	Pnkp	Bifunctional polynucleotide phosphatase/kinase	0,595202	0,509978
3552	AUHM_MOUSE	Auh	Methylglutaconyl-CoA hydratase, mitochondrial	0,713761	0,249685
3553	RABX5_MOUSE	Rabgef1	Rab5 GDP/GTP exchange factor	1,00064	0,824152
3554	NT5C_MOUSE	Nt5c	5'(3')-deoxyribonucleotidase, cytosolic type	0,0221539	-0,0142536
3555	MINK1_MOUSE	Mink1	Misshapen-like kinase 1	0,0849776	0,0494381
3556	REEP6_MOUSE	Reep6	Receptor expression-enhancing protein 6	1,97781	0,68267
3557	ARPC3_MOUSE	Arpc3	Actin-related protein 2/3 complex subunit 3	0,176996	0,16101
3558	UBP14_MOUSE	Usp14	Ubiquitin carboxyl-terminal hydrolase 14	0,664924	-0,183026
3559	ZN207_MOUSE	Znf207	BUB3-interacting and GLEBS motif-containing protein ZNF207	0,536626	0,369172
3560	STA10_MOUSE	Stard10	START domain-containing protein 10	0,182362	0,0993446
3561	TPC2L_MOUSE	Trappc2l	Trafficking protein particle complex subunit 2-like protein	1,10579	0,214978
3562	EDF1_MOUSE	Edf1	Endothelial differentiation-related factor 1	0,218944	0,332273
3563	TRXR1_MOUSE	Txnrd1	Thioredoxin reductase 1, cytoplasmic	0,0461917	0,0301991
3564	MY18A_MOUSE	Myo18a	Unconventional myosin-XVIIIa	1,31848	0,394786
3565	HAOX2_MOUSE	Hao2	Hydroxyacid oxidase 2	0,247559	0,335923
3566	GLRX1_MOUSE	Glrx	Glutaredoxin-1	0,108429	0,135981
3567	RHOA_MOUSE	Rhoa	Transforming protein RhoA	0,522323	-0,334977
3568	ACSL4_MOUSE	Acsl4	Long-chain-fatty-acid--CoA ligase 4	0,119025	0,118695
3569	PSA6_MOUSE	Psma6	Proteasome subunit alpha type-6	0,952878	0,235248
3570	HPLN1_MOUSE	Hapln1	Hyaluronan and proteoglycan link protein 1	2,73063	1,06107
3571	PPCE_MOUSE	Prep	Prolyl endopeptidase	0,383536	-0,189686
3572	PIN1_MOUSE	Pin1	Peptidyl-prolyl cis-trans isomerase NIMA-interacting 1	1,02607	0,709307
3573	FAK2_MOUSE	Ptk2b	Protein-tyrosine kinase 2-beta	0,222888	-0,465947
3574	ST1B1_MOUSE	Sult1b1	Sulfotransferase family cytosolic 1B member 1	0,309738	-0,258252
3575	CD5L_MOUSE	Cd5l	CD5 antigen-like	2,84374	1,56921
3576	NAGAB_MOUSE	Naga	Alpha-N-acetylgalactosaminidase	0,620953	-0,335906
3577	SON_MOUSE	Son	Protein SON	0,234504	0,225341
3578	DGUOK_MOUSE	Dguok	Deoxyguanosine kinase, mitochondrial	1,19161	-1,1492
3579	LSM4_MOUSE	Lsm4	U6 snRNA-associated Sm-like protein LSm4	0,180461	0,101962
3580	DRG2_MOUSE	Drg2	Developmentally-regulated GTP-binding protein 2	0,324212	0,115246
3581	FETUB_MOUSE	Fetub	Fetuin-B	0,608408	-0,228405
3582	ACOX2_MOUSE	Acox2	Peroxisomal acyl-coenzyme A oxidase 2	0,136701	-0,0785992
3583	F16P1_MOUSE	Fbp1	Fructose-1,6-bisphosphatase 1	1,13941	0,261461

3584	LIMD1_MOUSE	Limd1	LIM domain-containing protein 1	1,33855	0,446235
3585	HACL1_MOUSE	Hacl1	2-hydroxyacyl-CoA lyase 1	0,965274	0,438124
3586	TBL1X_MOUSE	Tbl1x	F-box-like/WD repeat-containing protein TBL1X	0,920226	-0,338957
3587	GNMT_MOUSE	Gnmt	Glycine N-methyltransferase	1,4973	-1,11349
3588	RAE1_MOUSE	Chm	Rab proteins geranylgeranyltransferase component A 1	0,627732	0,2085
3589	ACSA_MOUSE	Acss2	Acetyl-coenzyme A synthetase, cytoplasmic	0,199605	-0,468626
3590	COPG2_MOUSE	Copg2	Coatomer subunit gamma-2	0,161345	-0,085815
3591	CPSF3_MOUSE	Cpsf3	Cleavage and polyadenylation specificity factor subunit 3	0,13738	0,0553249
3592	KI21A_MOUSE	Kif21a	Kinesin-like protein KIF21A	1,6782	0,67179
3593	NDK7_MOUSE	Nme7	Nucleoside diphosphate kinase 7	1,48867	1,28373
3594	ABHD2_MOUSE	Abhd2	Monoacylglycerol lipase ABHD2	0,567905	0,891261
3595	TRIP4_MOUSE	Trip4	Activating signal cointegrator 1	0,00984812	-0,00886002
3596	PLEC_MOUSE	Plec	Plectin	0,976681	-0,164904
3597	CNPY2_MOUSE	Cnpy2	Protein canopy homolog 2	0,154235	0,127875
3598	CMC2_MOUSE	Slc25a13	Calcium-binding mitochondrial carrier protein Aralar2	0,14192	0,0911034
3599	ZO3_MOUSE	Tjp3	Tight junction protein ZO-3	2,45322	0,829159
3600	EHD3_MOUSE	Ehd3	EH domain-containing protein 3	0,00813572	0,00262375
3601	PEX3_MOUSE	Pex3	Peroxisomal biogenesis factor 3	1,43724	0,484442
3602	MACF1_MOUSE	Macf1	Microtubule-actin cross-linking factor 1	0,369332	0,274777
3603	MYO9B_MOUSE	Myo9b	Unconventional myosin-IXb	1,46468	1,93466
3604	ABCBB_MOUSE	Abcb11	Bile salt export pump	0,0517065	0,0283142
3605	NAA10_MOUSE	Naa10	N-alpha-acetyltransferase 10	0,595576	0,535133
3606	VAPB_MOUSE	Vapb	Vesicle-associated membrane protein-associated protein B	0,46872	0,136458
3607	PO210_MOUSE	Nup210	Nuclear pore membrane glycoprotein 210	1,13157	0,411907
3608	CLIC4_MOUSE	Clic4	Chloride intracellular channel protein 4	0,229047	0,0754646
3609	ADDG_MOUSE	Add3	Gamma-adducin	0,592472	0,802506
3610	ADDA_MOUSE	Add1	Alpha-adducin	0,512483	0,242399
3611	VKGC_MOUSE	Ggcx	Vitamin K-dependent gamma-carboxylase	0,247449	-0,440923
3612	GOGA5_MOUSE	Golga5	Golgin subfamily A member 5	1,13255	0,461129
3613	RDH11_MOUSE	Rdh11	Retinol dehydrogenase 11	0,195636	0,28809
3614	NDRG2_MOUSE	Ndrg2	Protein NDRG2	0,243424	-0,181444
3615	DNJC7_MOUSE	Dnajc7	DnaJ homolog subfamily C member 7	1,28951	0,433998
3616	DJB12_MOUSE	Dnajb12	DnaJ homolog subfamily B member 12	0,701557	0,290143
3617	DNJA2_MOUSE	Dnaja2	DnaJ homolog subfamily A member 2	0,311789	-0,13917
3618	DNJB1_MOUSE	Dnajb1	DnaJ homolog subfamily B member 1	2,50894	0,896761
3619	ACOT3_MOUSE	Acot3	Acyl-coenzyme A thioesterase 3	0,547267	0,716725
3620	ACOT2_MOUSE	Acot2	Acyl-coenzyme A thioesterase 2, mitochondrial	1,7491	1,34801
3621	QKI_MOUSE	Qki	Protein quaking	0,945616	0,362001
3622	SPAST_MOUSE	Spast	Spastin	1,56645	0,983284
3623	ADH4_MOUSE	Adh4	All-trans-retinol dehydrogenase [NAD(+)] ADH4	1,11296	0,909702
3624	TOLIP_MOUSE	Tollip	Toll-interacting protein	0,984862	0,259751
3625	NAGK_MOUSE	Nagk	N-acetyl-D-glucosamine kinase	0,230919	-0,225806
3626	NFU1_MOUSE	Nfu1	NFU1 iron-sulfur cluster scaffold homolog, mitochondrial	0,578412	0,360226
3627	VNN3_MOUSE	Vnn3	Vascular non-inflammatory molecule 3	0,947312	2,00099
3628	DCNL1_MOUSE	Dcun1d1	DCN1-like protein 1	1,32864	-0,483506
3629	IIGP1_MOUSE	Iigp1	Interferon-inducible GTPase 1	0,0039483	0,00351563
3630	VPS29_MOUSE	Vps29	Vacuolar protein sorting-associated protein 29	0,649251	-0,324362
3631	ARP10_MOUSE	Actr10	Actin-related protein 10	0,912525	0,928305
3632	DCTN5_MOUSE	Dctn5	Dynactin subunit 5	1,09454	0,457962
3633	DIC_MOUSE	Slc25a10	Mitochondrial dicarboxylate carrier	0,655667	-0,380078
3634	EIF3I_MOUSE	Eif3i	Eukaryotic translation initiation factor 3 subunit I	0,622268	0,403186
3635	COPG1_MOUSE	Copg1	Coatomer subunit gamma-1	0,393544	0,34468

3636	TSNAX_MOUSE	Tsnax	Translin-associated protein X	0,326685	0,253603
3637	ECSIT_MOUSE	Ecsit	Evolutionarily conserved signaling intermediate in Toll pathway, mitochondri	1,28354	0,389082
3638	FBX6_MOUSE	Fbxo6	F-box only protein 6	1,68206	0,550179
3639	AFAD_MOUSE	Afdn	Afadin	0,442843	0,250459
3640	H2AY_MOUSE	H2afy	Core histone macro-H2A.1	0,771707	0,363255
3641	NUMB_MOUSE	Numb	Protein numb homolog	1,76442	0,377579
3642	SGK2_MOUSE	Sgk2	Serine/threonine-protein kinase Sgk2	0,514956	-0,46677
3643	UB2L6_MOUSE	Ube2l6	Ubiquitin/ISG15-conjugating enzyme E2 L6	0,0651019	0,0294647
3644	AT11C_MOUSE	Atp11c	Phospholipid-transporting ATPase 11C	0,300271	-0,148244
3645	KIME_MOUSE	Mvk	Mevalonate kinase	0,212309	-0,273238
3646	ZRAB2_MOUSE	Zranb2	Zinc finger Ran-binding domain-containing protein 2	0,926987	0,324275
3647	AMFR_MOUSE	Amfr	E3 ubiquitin-protein ligase AMFR	1,28817	0,667955
3648	NUBP1_MOUSE	Nubp1	Cytosolic Fe-S cluster assembly factor NUBP1	0,570698	0,285083
3649	NUBP2_MOUSE	Nubp2	Cytosolic Fe-S cluster assembly factor NUBP2	1,28174	0,634407
3650	GLYG_MOUSE	Gyg1	Glycogenin-1	0,0222493	0,0233704
3651	GPC6_MOUSE	Gpc6	Glypican-6	1,92139	0,636625
3652	H17B6_MOUSE	Hsd17b6	17-beta-hydroxysteroid dehydrogenase type 6	0,13104	0,0833057
3653	TBL2_MOUSE	Tbl2	Transducin beta-like protein 2	0,264492	0,16232
3654	PEX14_MOUSE	Pex14	Peroxisomal membrane protein PEX14	1,23419	0,50098
3655	PLOD3_MOUSE	Plod3	Multifunctional procollagen lysine hydroxylase and glycosyltransferase LH3	1,55939	0,484822
3656	PLOD1_MOUSE	Plod1	Procollagen-lysine,2-oxoglutarate 5-dioxygenase 1	0,0587395	-0,0576252
3657	ACOX1_MOUSE	Acox1	Peroxisomal acyl-coenzyme A oxidase 1	1,19822	0,743946
3658	YLPM1_MOUSE	Ylpm1	YLP motif-containing protein 1	0,150238	0,0919342
3659	RAB9A_MOUSE	Rab9a	Ras-related protein Rab-9A	1,42643	0,259368
3660	GALK1_MOUSE	Galk1	Galactokinase	0,0314869	-0,0223038
3661	ESTD_MOUSE	Esd	S-formylglutathione hydrolase	0,602126	0,230153
3662	DEST_MOUSE	Dstn	Destrin	0,10989	-0,0886917
3663	TMED2_MOUSE	Tmed2	Transmembrane emp24 domain-containing protein 2	0,0646647	0,160847
3664	MO4L2_MOUSE	Morf4l2	Mortality factor 4-like protein 2	1,80465	1,21347
3665	ARC1A_MOUSE	Arpc1a	Actin-related protein 2/3 complex subunit 1A	0,00235371	-0,00109558
3666	TEBP_MOUSE	Ptges3	Prostaglandin E synthase 3	0,220217	-0,140366
3667	ACOT9_MOUSE	Acot9	Acyl-coenzyme A thioesterase 9, mitochondrial	0,759401	0,343981
3668	GUAD_MOUSE	Gda	Guanine deaminase	0,645173	0,420757
3669	SQOR_MOUSE	Sqor	Sulfide:quinone oxidoreductase, mitochondrial	0,52755	0,326723
3670	TYK2_MOUSE	Tyk2	Non-receptor tyrosine-protein kinase TYK2	0,0101065	0,00742416
3671	MTA2_MOUSE	Mta2	Metastasis-associated protein MTA2	0,310963	0,133774
3672	PR40A_MOUSE	Prpf40a	Pre-mRNA-processing factor 40 homolog A	1,47613	0,396885
3673	NSDHL_MOUSE	Nsdhl	Sterol-4-alpha-carboxylate 3-dehydrogenase, decarboxylating	0,296291	0,296435
3674	PSA4_MOUSE	Psma4	Proteasome subunit alpha type-4	0,319607	0,345562
3675	PSB3_MOUSE	Psmb3	Proteasome subunit beta type-3	0,193753	-0,203835
3676	PSB2_MOUSE	Psmb2	Proteasome subunit beta type-2	0,00293653	-0,00200119
3677	PSA1_MOUSE	Psma1	Proteasome subunit alpha type-1	1,55385	0,241854
3678	MRP6_MOUSE	Abcc6	Multidrug resistance-associated protein 6	1,00494	-0,556313
3679	SAE1_MOUSE	Sae1	SUMO-activating enzyme subunit 1	0,0922597	-0,0502357
3680	SEPT6_MOUSE	Septin6	Septin-6	0,94677	-0,573514
3681	PTPS_MOUSE	Pts	6-pyruvoyl tetrahydrobiopterin synthase	0,487541	1,10671
3682	VINEX_MOUSE	Sorbs3	Vinexin	0,045354	0,0325035
3683	TPSN_MOUSE	Tapbp	Tapasin	0,762825	0,403531
3684	HEBP1_MOUSE	Hebp1	Heme-binding protein 1	0,0459257	-0,0222267
3685	PEPL_MOUSE	Ppl	Periplakin	1,37297	1,51056
3686	MYO1C_MOUSE	Myo1c	Unconventional myosin-Ic	0,650815	0,190808
3687	FIZ1_MOUSE	Fiz1	Flt3-interacting zinc finger protein 1	0,859204	-0,681937

3688	NFKB2_MOUSE	Nfkb2	Nuclear factor NF-kappa-B p100 subunit	0,736547	0,448922
3689	LYPA2_MOUSE	Lypla2	Acyl-protein thioesterase 2	0,0445846	0,0683949
3690	RUVB2_MOUSE	Ruvbl2	RuvB-like 2	0,643876	0,295498
3691	KAD2_MOUSE	Ak2	Adenylate kinase 2, mitochondrial	2,16623	0,531147
3692	KAD3_MOUSE	Ak3	GTP:AMP phosphotransferase AK3, mitochondrial	1,26707	-0,311084
3693	AKA12_MOUSE	Akap12	A-kinase anchor protein 12	0,134607	0,284675
3694	TIM23_MOUSE	Timm23	Mitochondrial import inner membrane translocase subunit Tim23	0,794676	0,183559
3695	PRKRA_MOUSE	Prkra	Interferon-inducible double-stranded RNA-dependent protein kinase activatc	0,138753	-0,0789082
3696	SKP1_MOUSE	Skp1	S-phase kinase-associated protein 1	0,138157	0,0450584
3697	CUL1_MOUSE	Cul1	Cullin-1	0,732985	0,301837
3698	MD1L1_MOUSE	Mad1l1	Mitotic spindle assembly checkpoint protein MAD1	0,230625	-0,164329
3699	NRF1_MOUSE	Nrf1	Nuclear respiratory factor 1	0,604038	0,578453
3700	HAOX1_MOUSE	Hao1	Hydroxyacid oxidase 1	2,68618	0,942296
3701	PFD5_MOUSE	Pfdn5	Prefoldin subunit 5	0,582059	-0,532815
3702	MAN1_MOUSE	Lemd3	Inner nuclear membrane protein Man1	2,36057	0,592836
3703	NCOR2_MOUSE	Ncor2	Nuclear receptor corepressor 2	0,0369983	0,0381157
3704	TRUA_MOUSE	Pus1	tRNA pseudouridine synthase A	0,377933	0,340916
3705	PDC6I_MOUSE	Pdcd6ip	Programmed cell death 6-interacting protein	1,71807	0,176551
3706	PROD_MOUSE	Prodh	Proline dehydrogenase 1, mitochondrial	0,310904	-0,330989
3707	CCS_MOUSE	Ccs	Copper chaperone for superoxide dismutase	0,209644	0,125421
3708	SYFB_MOUSE	Farsb	Phenylalanine--tRNA ligase beta subunit	0,781484	0,359204
3709	HOIL1_MOUSE	Rbck1	RanBP-type and C3HC4-type zinc finger-containing protein 1	1,10592	0,730296
3710	PYGM_MOUSE	Pygm	Glycogen phosphorylase, muscle form	0,38879	-0,670576
3711	DCTN6_MOUSE	Dctn6	Dynactin subunit 6	1,76213	0,714344
3712	CHIP_MOUSE	Stub1	STIP1 homology and U box-containing protein 1	0,761429	0,308543
3713	SEM4G_MOUSE	Sema4g	Semaphorin-4G	0,116527	0,139666
3714	IF4H_MOUSE	Eif4h	Eukaryotic translation initiation factor 4H	0,97111	0,248043
3715	RFC2_MOUSE	Rfc2	Replication factor C subunit 2	1,26837	1,27543
3716	ARL3_MOUSE	Arl3	ADP-ribosylation factor-like protein 3	0,45374	0,402798
3717	COR1B_MOUSE	Coro1b	Coronin-1B	0,789011	-0,1881
3718	COR1C_MOUSE	Coro1c	Coronin-1C	1,42529	0,528622
3719	SUCA_MOUSE	Suclg1	Succinate--CoA ligase [ADP/GDP-forming] subunit alpha, mitochondrial	0,802214	-0,410253
3720	UCHL5_MOUSE	Uchl5	Ubiquitin carboxyl-terminal hydrolase isozyme L5	0,266402	-0,111002
3721	PREB_MOUSE	Preb	Prolactin regulatory element-binding protein	0,203681	0,166021
3722	ECI2_MOUSE	Eci2	Enoyl-CoA delta isomerase 2, mitochondrial	1,44588	0,745673
3723	KAD4_MOUSE	Ak4	Adenylate kinase 4, mitochondrial	0,618685	0,548356
3724	CATZ_MOUSE	Ctsz	Cathepsin Z	0,814712	0,527401
3725	ENTP5_MOUSE	Entpd5	Ectonucleoside triphosphate diphosphohydrolase 5	0,168187	-0,183372
3726	FA50A_MOUSE	Fam50a	Protein FAM50A	0,971858	0,367646
3727	ARC1B_MOUSE	Arpc1b	Actin-related protein 2/3 complex subunit 1B	0,864564	0,262572
3728	ASAH1_MOUSE	Asah1	Acid ceramidase	0,155704	-0,0696484
3729	VAPA_MOUSE	Vapa	Vesicle-associated membrane protein-associated protein A	0,899595	-0,752294
3730	GSK3B_MOUSE	Gsk3b	Glycogen synthase kinase-3 beta	0,889503	0,433144
3731	DECR2_MOUSE	Decr2	Peroxisomal 2,4-dienoyl-CoA reductase	0,171634	0,210611
3732	SNX1_MOUSE	Snx1	Sorting nexin-1	0,61992	-0,372422
3733	NDK3_MOUSE	Nme3	Nucleoside diphosphate kinase 3	0,504796	-0,384934
3734	T10B_MOUSE	Timm10b	Mitochondrial import inner membrane translocase subunit Tim10 B	0,491584	0,52986
3735	TIM9_MOUSE	Timm9	Mitochondrial import inner membrane translocase subunit Tim9	0,626091	0,397252
3736	TIM8A_MOUSE	Timm8a1	Mitochondrial import inner membrane translocase subunit Tim8 A	0,442632	0,384115
3737	BUB3_MOUSE	Bub3	Mitotic checkpoint protein BUB3	1,28141	0,311586
3738	TAGL2_MOUSE	Tagln2	Transgelin-2	0,125008	0,0513172
3739	RBPMS_MOUSE	Rbpms	RNA-binding protein with multiple splicing	0,772958	1,89584

3740	ORNT1_MOUSE	Slc25a15	Mitochondrial ornithine transporter 1	1,46543	-0,634962
3741	PACN2_MOUSE	Pacsin2	Protein kinase C and casein kinase substrate in neurons protein 2	0,0287296	-0,00924187
3742	PSD13_MOUSE	Psmd13	26S proteasome non-ATPase regulatory subunit 13	0,0367071	0,0202099
3743	CBPQ_MOUSE	Cpq	Carboxypeptidase Q	0,0671763	0,0357693
3744	EHD1_MOUSE	Ehd1	EH domain-containing protein 1	0,0471351	-0,0214844
3745	MAAI_MOUSE	Gstz1	Maleylacetoacetate isomerase	0,105231	0,0512672
3746	STAT2_MOUSE	Stat2	Signal transducer and activator of transcription 2	0,458434	-0,865554
3747	S12A7_MOUSE	Slc12a7	Solute carrier family 12 member 7	0,461445	-0,921795
3748	APC7_MOUSE	Anapc7	Anaphase-promoting complex subunit 7	0,73635	0,458334
3749	AADAT_MOUSE	Aadat	Kynurenine/alpha-aminoadipate aminotransferase, mitochondrial	0,310446	-0,131392
3750	MTNB_MOUSE	Apip	Methylthioribulose-1-phosphate dehydratase	0,606259	-0,381432
3751	CAH14_MOUSE	Ca14	Carbonic anhydrase 14	0,48041	-0,409881
3752	CELF2_MOUSE	Celf2	CUGBP Elav-like family member 2	2,20958	0,65224
3753	VNN1_MOUSE	Vnn1	Pantetheinase	2,60823	1,44941
3754	LICH_MOUSE	Lipa	Lysosomal acid lipase/cholesteryl ester hydrolase	0,651883	-0,989815
3755	IF2G_MOUSE	Eif2s3x	Eukaryotic translation initiation factor 2 subunit 3, X-linked	1,03453	0,422683
3756	IF2H_MOUSE	Eif2s3y	Eukaryotic translation initiation factor 2 subunit 3, Y-linked	0,985359	0,6
3757	ITSN1_MOUSE	Itsn1	Intersectin-1	0,532578	0,262472
3758	ITSN2_MOUSE	Itsn2	Intersectin-2	0,476536	0,399938
3759	FADS2_MOUSE	Fads2	Acyl-CoA 6-desaturase	2,10852	0,991335
3760	BPNT1_MOUSE	Bpnt1	3'(2'),5'-bisphosphate nucleotidase 1	0,820366	0,39427
3761	ZO2_MOUSE	Tjp2	Tight junction protein ZO-2	0,312784	0,133317
3762	NU160_MOUSE	Nup160	Nuclear pore complex protein Nup160	0,788068	-0,757922
3763	AIFM1_MOUSE	Aifm1	Apoptosis-inducing factor 1, mitochondrial	0,349976	0,317495
3764	DCTN3_MOUSE	Dctn3	Dynactin subunit 3	0,709949	-1,7673
3765	ADNP_MOUSE	Adnp	Activity-dependent neuroprotector homeobox protein	0,101426	-0,0317898
3766	STAU1_MOUSE	Stau1	Double-stranded RNA-binding protein Staufen homolog 1	0,293919	0,211543
3767	P5CS_MOUSE	Aldh18a1	Delta-1-pyrroline-5-carboxylate synthase	0,793888	0,495255
3768	PLF4_MOUSE	Pf4	Platelet factor 4	0,930852	0,700634
3769	HNRDL_MOUSE	Hnrnpdl	Heterogeneous nuclear ribonucleoprotein D-like	0,130664	-0,147016
3770	COG1_MOUSE	Cog1	Conserved oligomeric Golgi complex subunit 1	0,250561	-0,23396
3771	PLCB1_MOUSE	Plcb1	1-phosphatidylinositol 4,5-bisphosphate phosphodiesterase beta-1	0,170036	-0,302483
3772	EIF3G_MOUSE	Eif3g	Eukaryotic translation initiation factor 3 subunit G	0,267582	0,17924
3773	SAE2_MOUSE	Uba2	SUMO-activating enzyme subunit 2	1,12273	0,685062
3774	VATC1_MOUSE	Atp6v1c1	V-type proton ATPase subunit C 1	0,75051	0,414503
3775	VPP1_MOUSE	Atp6v0a1	V-type proton ATPase 116 kDa subunit a isoform 1	0,605262	0,321232
3776	NFS1_MOUSE	Nfs1	Cysteine desulfurase, mitochondrial	0,601994	0,146434
3777	ARI1_MOUSE	Arih1	E3 ubiquitin-protein ligase ARIH1	2,07604	0,498802
3778	ARI2_MOUSE	Arih2	E3 ubiquitin-protein ligase ARIH2	0,140768	0,0724846
3779	KS6B2_MOUSE	Rps6kb2	Ribosomal protein S6 kinase beta-2	1,59426	0,444613
3780	RED_MOUSE	Ik	Protein Red	0,349207	0,13715
3781	DX39B_MOUSE	Ddx39b	Spliceosome RNA helicase Ddx39b	0,385188	0,168601
3782	NDUA7_MOUSE	Ndufa7	NADH dehydrogenase [ubiquinone] 1 alpha subcomplex subunit 7	0,718387	0,481554
3783	KANK3_MOUSE	Kank3	KN motif and ankyrin repeat domain-containing protein 3	0,110141	0,104699
3784	ABHGA_MOUSE	Abhd16a	Phosphatidylserine lipase ABHD16A	0,50091	0,164896
3785	CLIC1_MOUSE	Clic1	Chloride intracellular channel protein 1	0,828058	0,246205
3786	SYVC_MOUSE	Vars	Valine--tRNA ligase	0,95116	0,210938
3787	BAG6_MOUSE	Bag6	Large proline-rich protein BAG6	0,523517	0,277671
3788	APOM_MOUSE	Apom	Apolipoprotein M	1,06808	0,37858
3789	AP3B1_MOUSE	Ap3b1	AP-3 complex subunit beta-1	1,41642	0,15208
3790	TSP4_MOUSE	Thbs4	Thrombospondin-4	0,130904	0,220282
3791	ILF3_MOUSE	Ilf3	Interleukin enhancer-binding factor 3	0,00852094	-0,00598183

3792	TRIP6_MOUSE	Trip6	Thyroid receptor-interacting protein 6	0,378702	-0,51284
3793	USO1_MOUSE	Uso1	General vesicular transport factor p115	0,431697	0,13344
3794	STRAP_MOUSE	Strap	Serine-threonine kinase receptor-associated protein	0,360241	0,14347
3795	HNRPC_MOUSE	Hnrnpc	Heterogeneous nuclear ribonucleoproteins C1/C2	1,25376	0,334887
3796	PX11B_MOUSE	Pex11b	Peroxisomal membrane protein 11B	1,71338	0,691398
3797	MOC2B_MOUSE	Mocs2	Molybdopterin synthase catalytic subunit	0,476796	0,63034
3798	FKBP9_MOUSE	Fkbp9	Peptidyl-prolyl cis-trans isomerase FKBP9	0,668155	0,316656
3799	BAZ1B_MOUSE	Baz1b	Tyrosine-protein kinase BAZ1B	1,57231	0,841904
3800	ATE1_MOUSE	Ate1	Arginyl-tRNA--protein transferase 1	0,58189	0,480737
3801	MTM1_MOUSE	Mtm1	Myotubularin	0,558151	0,28591
3802	MTMR2_MOUSE	Mtmr2	Myotubularin-related protein 2	0,210555	0,0889141
3803	SE1L1_MOUSE	Sel1l	Protein sel-1 homolog 1	0,57933	0,309701
3804	HTAI2_MOUSE	Htatip2	Oxidoreductase HTATIP2	0,250436	-0,0816216
3805	LETM1_MOUSE	Letm1	Mitochondrial proton/calcium exchanger protein	0,385373	0,201164
3806	SUCB2_MOUSE	Suclg2	Succinate--CoA ligase [GDP-forming] subunit beta, mitochondrial	0,43457	-0,156327
3807	SUCB1_MOUSE	Sucla2	Succinate--CoA ligase [ADP-forming] subunit beta, mitochondrial	0,0247226	0,0188694
3808	PMM2_MOUSE	Pmm2	Phosphomannomutase 2	1,28028	0,393249
3809	ACL6A_MOUSE	Actl6a	Actin-like protein 6A	0,105846	0,0491535
3810	RM40_MOUSE	Mrpl40	39S ribosomal protein L40, mitochondrial	0,562147	-0,471907
3811	U119A_MOUSE	Unc119	Protein unc-119 homolog A	2,43063	2,4093
3812	PSA7_MOUSE	Psma7	Proteasome subunit alpha type-7	0,473122	-0,138553
3813	PSA5_MOUSE	Psma5	Proteasome subunit alpha type-5	0,299601	0,310252
3814	PCKGC_MOUSE	Pck1	Phosphoenolpyruvate carboxykinase, cytosolic [GTP]	3,13945	-0,817995
3815	HDAC6_MOUSE	Hdac6	Histone deacetylase 6	0,272162	0,261075
3816	DNPEP_MOUSE	Dnpep	Aspartyl aminopeptidase	1,11639	0,417139
3817	HNRPF_MOUSE	Hnrnpf	Heterogeneous nuclear ribonucleoprotein F	0,586052	-0,410765
3818	PSD10_MOUSE	Psmd10	26S proteasome non-ATPase regulatory subunit 10	1,30636	1,46225
3819	KEAP1_MOUSE	Keap1	Kelch-like ECH-associated protein 1	1,79087	0,680231
3820	PLPHP_MOUSE	Plpbp	Pyridoxal phosphate homeostasis protein	0,576825	0,314813
3821	MCAT_MOUSE	Slc25a20	Mitochondrial carnitine/acylcarnitine carrier protein	0,614874	0,26517
3822	SNUT1_MOUSE	Sart1	U4/U6.U5 tri-snRNP-associated protein 1	1,21419	0,786573
3823	TOP3B_MOUSE	Top3b	DNA topoisomerase 3-beta-1	1,03996	0,237527
3824	ITPR2_MOUSE	Itpr2	Inositol 1,4,5-trisphosphate receptor type 2	0,927312	-0,667368

5. Supplementary Table S5 (Proteomic analysis; Overview of all significantly altered pathways in the offspring by maternal diet)

**A list of significantly altered pathways in the offspring by maternal diet**									
**Source: EnrichR database, (https://maayanlab.cloud/Enrichr/; accessed on: 12th december, 2021)**									
**Pathway: Wiki Pathway**									
**Term**	**P-value**	**Adjusted P-value**	**Old P-value**	**Old Adjusted P-value**	**Odds Ratio**	**Combined Score**	**Genes**	**Negative Log10**	
Caloric restriction and aging WP4191	0,003196032	0.032576852487108024	0	0	40.785.714.285.714.200	23.434.840.743.955.800	IGF1	2,4953889	
Trans-sulfuration pathway WP2333	0,003993646	0.032576852487108024	0	0	3.171.904.761.904.760	17.518.590.547.314.000	CSAD	2,3986304	
Liver X receptor pathway WP2874	0,003993646	0.032576852487108024	0	0	3.171.904.761.904.760	17.518.590.547.314.000	CYP7A1	2,3986304	
Methionine metabolism leading to sulfur amino acids and related disorders WP4292	0,004392244	0.032576852487108024	0	0	28.545.714.285.714.200	154.943.708.775.113	CSAD	2,3573135	
Bile acids synthesis and enterohepatic circulation WP4389	0,004790703	0.032576852487108024	0	0	2.594.935.064.935.060	1.385.975.083.259.070	CYP7A1	2,3196007	
Genes targeted by miRNAs in adipocytes WP1992	0,005189022	0.032576852487108024	0	0	23.785.714.285.714.200	1.251.416.370.420.650	IGF1	2,2849145	
Cysteine and methionine catabolism WP4504	0,005985243	0.032576852487108024	0	0	20.385.714.285.714.200	1.043.434.298.452.160	CSAD	2,2229182	
Drug Induction of Bile Acid Pathway WP2289	0,006780906	0.032576852487108024	0	0	17.835.714.285.714.200	8.906.521.752.159.380	CYP7A1	2,1687123	
Extracellular vesicles in the crosstalk of cardiac cells WP4300	0,007576012	0.032576852487108024	0	0	15.852.380.952.380.900	7.740.350.338.275.500	IGF1	2,1205593	
Farnesoid X receptor pathway WP2879	0,007576012	0.032576852487108024	0	0	15.852.380.952.380.900	7.740.350.338.275.500	CYP7A1	2,1205593	
Methionine De Novo and Salvage Pathway WP3580	0,008767628	0.033201526013009026	0	0	13.585.714.285.714.200	6.435.130.349.149.830	TAT	2,0571179	
PPAR-alpha pathway WP2878	0,010354502	0.033201526013009026	0	0	11.409.714.285.714.200	5.214.620.402.892.070	CYP7A1	1,9848708	
7-oxo-C and 7beta-HC pathways WP5064	0,010750873	0.033201526013009026	0	0	1.097.032.967.032.960	49.725.962.914.000.300	CYP7A1	1,9685563	
Statin inhibition of cholesterol production WP430	0,011543199	0.033201526013009026	0	0	10.185.714.285.714.200	4.544.518.266.085.440	CYP7A1	1,9376738	
Oligodendrocyte specification and differentiation, leading to myelin components for CNS WP4304	0,011939153	0.033201526013009026	0	0	9.833.990.147.783.250	435.442.406.227.662	IGF1	1,9230265	
Nuclear Receptors in Lipid Metabolism and Toxicity WP299	0,013126185	0.033201526013009026	0	0	8.910.714.285.714.280	3.861.142.784.895.610	CYP7A1	1,8818615	
Fluoropyrimidine Activity WP1601	0,013126185	0.033201526013009026	0	0	8.910.714.285.714.280	3.861.142.784.895.610	UPP2	1,8818615	
Factors and pathways affecting insulin-like growth factor (IGF1)-Akt signaling WP3850	0,014706953	0.03513327604364505	0	0	7.919.047.619.047.610	3.341.390.605.677.810	IGF1	1,8324773	
Oxysterols derived from cholesterol WP4545	0,018255584	0.04032335834993025	0	0	6.332.380.952.380.950	25.350.320.962.011.800	CYP7A1	1,7386043	
Differentiation Pathway WP2848	0,019042649	0.04032335834993025	0	0	6.062.310.030.395.130	24.013.259.456.270.100	IGF1	1,7202726	
One-carbon metabolism and related pathways WP3940	0.020615122198689464	0.04032335834993025	0	0	55.857.142.857.142.800	21.682.236.865.801.600	CSAD		
Cardiac Progenitor Differentiation WP2406	0.02100789576309119	0.04032335834993025	0	0	5.478.021.978.021.970	21.160.815.123.277.500	IGF1		
Cardiac Hypertrophic Response WP2795	0.02179302948367254	0.04032335834993025	0	0	5.274.603.174.603.170	2.018.150.262.928.950	IGF1		
Oxidation by Cytochrome P450 WP43	0.024145126293327844	0.04032335834993025	0	0	47.457.142.857.142.800	17.671.486.865.577.200	CYP7A1		
Lung fibrosis WP3624	0.02492805820599394	0.04032335834993025	0	0	4.592.165.898.617.510	1.695.318.022.982.440	IGF1		
Endochondral Ossification with Skeletal Dysplasias WP4808	0.025319318033677134	0.04032335834993025	0	0	4.519.047.619.047.610	16.612.866.893.112.000	IGF1		
Endochondral Ossification WP474	0.025319318033677134	0.04032335834993025	0	0	4.519.047.619.047.610	16.612.866.893.112.000	IGF1		
PPAR signaling pathway WP3942	0.026492273471440286	0.04068456283114044	0	0	4.312.987.012.987.010	1.566.003.384.261.290	CYP7A1		
MECP2 and Associated Rett Syndrome WP3584	0.02844445487658815	0.04217626067907898	0	0	4.008.249.496.981.890	14.268.574.756.562.200	IGF1		
EGFR Tyrosine Kinase Inhibitor Resistance WP4806	0.03311572207601827	0.04449925153964955	0	0	3.426.678.141.135.970	116.772.525.740.896	IGF1		
MicroRNAs in cardiomyocyte hypertrophy WP1544	0.03311572207601827	0.04449925153964955	0	0	3.426.678.141.135.970	116.772.525.740.896	IGF1		
Apoptosis WP254	0.03311572207601827	0.04449925153964955	0	0	3.426.678.141.135.970	116.772.525.740.896	IGF1		
Amino Acid metabolism WP3925	0.03583154305979738	0.046689586411251136	0	0	31.590.476.190.476.100	10.516.237.910.185.800	TAT		
Senescence and Autophagy in Cancer WP615	0.0412431688635261	0.05216047826857713	0	0	27.318.681.318.681.300	8.709.932.616.586.790	IGF1		

5. Supplementary Table S5 (Proteomic analysis; Overview of all significantly altered pathways in the offspring by maternal diet)

Adipogenesis WP236	0.05084071155533055	0.061639150861224734	0	0	21.996.677.740.863.700	6.552.937.520.714.520	IGF1		
Angiopoietin Like Protein 8 Regulatory Pathway WP3915	0.05160487048846722	0.061639150861224734	0	0	2.165.866.957.470.010	641.993.119.748.582	CYP7A1		
Breast cancer pathway WP4262	0.05997515874305551	0.06970086016084828	0	0	18.523.809.523.809.500	521.227.550.627.593	IGF1		
Vitamin D Receptor Pathway WP2877	0.07053472095805195	0.07818273305050236	0	0	15.636.148.382.004.700	41.461.595.899.019.500	CYP7A1		
Metapathway biotransformation Phase I and II WP702	0.07090992067371144	0.07818273305050236	0	0	1.554.945.054.945.050	4.114.920.963.136.880	CYP7A1		
Focal Adhesion WP306	0.07652203515417907	0.0822611877907425	0	0	14.354.604.786.076.800	3.689.386.843.909.950	IGF1		
Focal Adhesion-PI3K-Akt-mTOR-signaling pathway WP3932	0.1149831829216247	0.12059211867389907	0	0	9.314.096.499.526.960	2.014.610.568.709.960	IGF1		
Nuclear Receptors Meta-Pathway WP2882	0.1207191050086845	0.12359336941365318	0	0	8.838.274.932.614.550	18.686.666.393.634.900	CYP7A1		
PI3K-Akt signaling pathway WP4172	0.1281981235974174	0.1281981235974174	0	0	8.281.921.618.204.800	170.125.442.594.961	IGF1		
**KEGG Pathway**									
Term	P-value	Adjusted P-value	Old P-value	Old Adjusted P-value	Odds Ratio	Combined Score	Genes		
Phenylalanine, tyrosine and tryptophan biosynthesis	0,002397859	0.06238433538489801	0	0	5.710.571.428.571.420	34.452.899.826.083.200	TAT	2,6201764	
Taurine and hypotaurine metabolism	0,004392244	0.06238433538489801	0	0	28.545.714.285.714.200	154.943.708.775.113	CSAD	2,3573135	
Ubiquinone and other terpenoid-quinone biosynthesis	0,004392244	0.06238433538489801	0	0	28.545.714.285.714.200	154.943.708.775.113	TAT	2,3573135	
Phenylalanine metabolism	0,006780906	0.06238433538489801	0	0	17.835.714.285.714.200	8.906.521.752.159.380	TAT	2,1687123	
Primary bile acid biosynthesis	0,006780906	0.06238433538489801	0	0	17.835.714.285.714.200	8.906.521.752.159.380	CYP7A1	2,1687123	
Asthma	0,012334969	0.06786500235638772	0	0	9.505.714.285.714.280	4.178.062.796.500.490	PRG2	1,9088619	
Tyrosine metabolism	0,014311969	0.06786500235638772	0	0	8.145.714.285.714.280	34.592.071.940.977.700	TAT	1,8443006	
Aldosterone-regulated sodium reabsorption	0,014706953	0.06786500235638772	0	0	7.919.047.619.047.610	3.341.390.605.677.810	IGF1	1,8324773	
Cysteine and methionine metabolism	0,019829161	0.06786500235638772	0	0	58.142.857.142.857.100	2.279.549.801.512.960	TAT	1,7026957	
Cholesterol metabolism	0,019829161	0.06786500235638772	0	0	58.142.857.142.857.100	2.279.549.801.512.960	CYP7A1	1,7026957	
Ovarian steroidogenesis	0.020222210774875234	0.06786500235638772	0	0	5.697.714.285.714.280	22.226.633.779.925.100	IGF1	#WERT!	
Pyrimidine metabolism	0.022185389719098877	0.06786500235638772	0	0	51.784.415.584.415.500	19.721.169.430.337.900	UPP2	#WERT!	
Long-term depression	0.023753454121096102	0.06786500235638772	0	0	4.826.392.251.815.980	18.050.838.890.404.800	IGF1	#WERT!	
Steroid hormone biosynthesis	0.024145126293327844	0.06786500235638772	0	0	47.457.142.857.142.800	17.671.486.865.577.200	CYP7A1	#WERT!	
Melanoma	0.02844445487658815	0.06786500235638772	0	0	4.008.249.496.981.890	14.268.574.756.562.200	IGF1	#WERT!	
p53 signaling pathway	0,02883448	0.06786500235638772	0	0	39.523.809.523.809.500	14.015.867.684.434.900	IGF1	1,5400879	
PPAR signaling pathway	0,029224368	0.06786500235638772	0	0	38.980.430.528.375.700	13.770.820.955.371.100	CYP7A1	1,5342549	
Glioma	0.029614118926114725	0.06786500235638772	0	0	3.845.173.745.173.740	13.533.104.533.059.200	IGF1	#WERT!	
Hypertrophic cardiomyopathy	0.03544397773863309	0.06786500235638772	0	0	31.947.030.497.592.200	1.066.967.538.219.120	IGF1	#WERT!	
Bile secretion	0.03544397773863309	0.06786500235638772	0	0	31.947.030.497.592.200	1.066.967.538.219.120	CYP7A1	#WERT!	
Dilated cardiomyopathy	0.03776732657425956	0.06786500235638772	0	0	299.203.007.518.797	9.802.820.827.717.790	IGF1	#WERT!	
Prostate cancer	0.03815407494862228	0.06786500235638772	0	0	29.607.142.857.142.800	967.005.617.556.944	IGF1	#WERT!	
Inflammatory mediator regulation of TRP channels	0.03854068730993136	0.06786500235638772	0	0	2.930.044.182.621.500	9.540.343.353.076.330	IGF1	#WERT!	
Progesterone-mediated oocyte maturation	0.03931350415205654	0.06786500235638772	0	0	28.705.627.705.627.700	9.289.678.500.716.380	IGF1	#WERT!	
Pancreatic secretion	0.04008577742562173	0.06786500235638772	0	0	28.134.370.579.915.100	905.007.775.576.607	PRSS2	#WERT!	
Longevity regulating pathway	0.04008577742562173	0.06786500235638772	0	0	28.134.370.579.915.100	905.007.775.576.607	IGF1	#WERT!	
Protein digestion and absorption	0.04047171032468564	0.06786500235638772	0	0	27.857.142.857.142.800	8.934.209.308.449.350	PRSS2	#WERT!	
Drug metabolism	0.04239933910860823	0.06786500235638772	0	0	26.548.731.642.189.500	8.391.051.867.901.870	UPP2	#WERT!	
HIF-1 signaling pathway	0.042784458007287904	0.06786500235638772	0	0	263.015.873.015.873	8.289.156.631.877.400	IGF1	#WERT!	
Growth hormone synthesis, secretion and action	0,0466282	0.0697594900843374	0	0	2.406.053.268.765.130	7.375.876.051.746.780	IGF1	1,3313514	
AMPK signaling pathway	0,04701183	0.0697594900843374	0	0	23.857.142.857.142.800	7.293.977.888.059.270	IGF1	1,3277928	
Oocyte meiosis	0.050458430115721514	0.07140156010598374	0	0	22.169.642.857.142.800	6.621.197.613.100.180	IGF1	#WERT!	

5. Supplementary Table S5 (Proteomic analysis; Overview of all significantly altered pathways in the offspring by maternal diet)

FoxO signaling pathway	0,051222858	0.07140156010598374	0	0	21.826.373.626.373.600	6.485.858.386.485.300	IGF1	1,2905362	
Signaling pathways regulating pluripotency of stem cells	0.05579813022483266	0.07533344408071158	0	0	19.969.818.913.480.800	5.763.319.530.748.080	IGF1	#WERT!	
Breast cancer	0.05731892484401968	0.07533344408071158	0	0	19.418.786.692.759.200	55.520.727.497.935.700	IGF1		
mTOR signaling pathway	0.05997515874305551	0.07663492506057092	0	0	18.523.809.523.809.500	521.227.550.627.593	IGF1		
Influenza A	0.0667754258809193	0.08301809704114291	0	0	1.655.889.724.310.770	4.481.533.303.064.930	PRSS2		
Transcriptional misregulation in cancer	0.07428075901853666	0.0899188135487549	0	0	1.481.002.243.829.460	3.850.462.656.889.650	IGF1		
Focal adhesion	0.07764089162771914	0.09086721931389949	0	0	14.137.142.857.142.800	3.612.974.516.911.650	IGF1		
Proteoglycans in cancer	0.07913085511201665	0.09086721931389949	0	0	13.857.142.857.142.800	3.515.075.472.727.730	IGF1		
Rap1 signaling pathway	0.08099034764934519	0.09086721931389949	0	0	13.522.214.627.477.700	3.398.707.630.579.840	IGF1		
Ras signaling pathway	0.08913314087389414	0.09762201143331263	0	0	12.220.779.220.779.200	29.545.249.903.179.500	IGF1		
MAPK signaling pathway	0.11174236079929967	0.11953833945971591	0	0	96.045.831.301.804	2.104.901.451.466.680	IGF1		
Neuroactive ligand-receptor interaction	0.12855287461902729	0.13439618710171033	0	0	8.257.142.857.142.850	16.938.826.579.242.500	PRSS2		
PI3K-Akt signaling pathway	0.1331531570366007	0.13611211608185847	0	0	7.947.794.415.216.510	16.024.782.268.271.000	IGF1		
Pathways in cancer	0.19370711172776245	0.19370711172776245	0	0	5.245.822.102.425.870	8.610.534.334.425.610	IGF1		
**GO Molecular Function**									
Term	P-value	Adjusted P-value	Old P-value	Old Adjusted P-value	Odds Ratio	Combined Score	Genes		
insulin-like growth factor receptor binding (GO:0005159)	0,005587202	0.03624431640160541	0	0	21.954.945.054.945.000	11.388.637.280.375.100	IGF1	2,2528056	
carboxy-lyase activity (GO:0016831)	0,009164555	0.03624431640160541	0	0	12.967.532.467.532.400	6.084.900.471.331.720	CSAD	2,0378886	
steroid hydroxylase activity (GO:0008395)	0,014311969	0.03624431640160541	0	0	8.145.714.285.714.280	34.592.071.940.977.700	CYP7A1	1,8443006	
oxidoreductase activity, acting on paired donors, with incorporation or reduction of molecular oxygen, NAD(P)H as one donor, and incorporation of one atom of oxygen (GO:0016709)	0,014311969	0.03624431640160541	0	0	8.145.714.285.714.280	34.592.071.940.977.700	CYP7A1	1,8443006	
pentosyltransferase activity (GO:0016763)	0,015101799	0.03624431640160541	0	0	7.704.633.204.633.200	3.230.507.581.480.840	UPP2	1,8209713	
hormone activity (GO:0005179)	0,030782551	0.06156510147875503	0	0	3.694.805.194.805.190	1.286.090.483.496.380	IGF1	1,5116954	
serine-type endopeptidase activity (GO:0004252)	0,041243169	0.07070257519461617	0	0	27.318.681.318.681.300	8.709.932.616.586.790	PRSS2	1,384648	
serine-type peptidase activity (GO:0008236)	0,048927957	0.07339193523442711	0	0	22.889.400.921.658.900	690.666.232.697.606	PRSS2	1,3104429	
receptor ligand activity (GO:0048018)	0,116420224	0.142944651285175	0	0	919.047.619.047.619	19.764.569.490.964.600	IGF1	0,9339716	
endopeptidase activity (GO:0004175)	0,119288183	0.142944651285175	0	0	8.952.684.258.416.740	19.035.313.745.752.000	PRSS2	0,9234026	
calcium ion binding (GO:0005509)	0.13103259701141043	0.142944651285175	0	0	8.087.690.407.575.130	16.436.687.254.775.500	PRSS2		
metal ion binding (GO:0046872)	0.18905619939244717	0.18905619939244717	0	0	5.392.026.578.073.080	8.981.557.750.667.950	PRSS2		
**GO Biological Process**									
Term	P-value	Adjusted P-value	Old P-value	Old Adjusted P-value	Odds Ratio	Combined Score	Genes		
positive regulation of cell growth (GO:0030307)	7,07E-04	0.029226028922216006	0	0	6.630.666.666.666.660	48.103.292.886.084.700	3,150642		
negative regulation of smooth muscle cell apoptotic process (GO:0034392)	0,001998563	0.029226028922216006	0	0	7.138.571.428.571.420	4.436.855.317.641.390	IGF1	2,6992821	

5. Supplementary Table S5 (Proteomic analysis; Overview of all significantly altered pathways in the offspring by maternal diet)

positive regulation of glycoprotein metabolic process (GO:1903020)	0,001998563	0.0292260289 22216006	0	0	7.138.571.428. 571.420	4.436.855.317. 641.390	IGF1	2,699 2821	
taurine metabolic process (GO:0019530)	0,002397859	0.0292260289 22216006	0	0	5.710.571.428. 571.420	34.452.899.82 6.083.200	CSA D	2,620 1764	
tyrosine catabolic process (GO:0006572)	0,002397859	0.0292260289 22216006	0	0	5.710.571.428. 571.420	34.452.899.82 6.083.200	TAT	2,620 1764	
regulation of vascular associated smooth muscle cell apoptotic process (GO:1905459)	0,002797015	0.0292260289 22216006	0	0	47.585.714.28 5.714.200	27.976.605.00 2.155.400	IGF1	2,553 3052	
regulation of bile acid metabolic process (GO:1904251)	0,002797015	0.0292260289 22216006	0	0	47.585.714.28 5.714.200	27.976.605.00 2.155.400	CYP7 A1	2,553 3052	
positive regulation of insulin-like growth factor receptor signaling pathway (GO:0043568)	0,003196032	0.0292260289 22216006	0	0	40.785.714.28 5.714.200	23.434.840.74 3.955.800	IGF1	2,495 3889	
negative regulation of muscle cell apoptotic process (GO:0010656)	0,003196032	0.0292260289 22216006	0	0	40.785.714.28 5.714.200	23.434.840.74 3.955.800	IGF1	2,495 3889	
regulation of smooth muscle cell apoptotic process (GO:0034391)	0,003594909	0.0292260289 22216006	0	0	35.685.714.28 5.714.200	20.084.764.65 9.134.400	IGF1	2,444 3121	
sterol catabolic process (GO:0016127)	0.00359490866 6217628	0.0292260289 22216006	0	0	35.685.714.28 5.714.200	20.084.764.65 9.134.400	CYP7 A1	#WER T!	
cholesterol catabolic process (GO:0006707)	0.00359490866 6217628	0.0292260289 22216006	0	0	35.685.714.28 5.714.200	20.084.764.65 9.134.400	CYP7 A1		
regulation of glycoprotein biosynthetic process (GO:0010559)	0.00399364626 13403625	0.0292260289 22216006	0	0	3.171.904.761. 904.760	17.518.590.54 7.314.000	IGF1		
regulation of bile acid biosynthetic process (GO:0070857)	0.00399364626 13403625	0.0292260289 22216006	0	0	3.171.904.761. 904.760	17.518.590.54 7.314.000	CYP7 A1		
nucleoside metabolic process (GO:0009116)	0.00399364626 13403625	0.0292260289 22216006	0	0	3.171.904.761. 904.760	17.518.590.54 7.314.000	UPP2		
alcohol catabolic process (GO:0046164)	0.00439224439 6163033	0.0292260289 22216006	0	0	28.545.714.28 5.714.200	154.943.708.7 75.113	CYP7 A1		
pyrimidine nucleoside catabolic process (GO:0046135)	0.00439224439 6163033	0.0292260289 22216006	0	0	28.545.714.28 5.714.200	154.943.708.7 75.113	UPP2		
pyrimidine nucleoside salvage (GO:0043097)	0.00439224439 6163033	0.0292260289 22216006	0	0	28.545.714.28 5.714.200	154.943.708.7 75.113	UPP2		
pyrimidine-containing compound salvage (GO:0008655)	0.00439224439 6163033	0.0292260289 22216006	0	0	28.545.714.28 5.714.200	154.943.708.7 75.113	UPP2		
myotube cell development (GO:0014904)	0.00439224439 6163033	0.0292260289 22216006	0	0	28.545.714.28 5.714.200	154.943.708.7 75.113	IGF1		
insulin-like growth factor receptor signaling pathway (GO:0048009)	0.00439224439 6163033	0.0292260289 22216006	0	0	28.545.714.28 5.714.200	154.943.708.7 75.113	IGF1		
cellular response to cholesterol (GO:0071397)	0.00439224439 6163033	0.0292260289 22216006	0	0	28.545.714.28 5.714.200	154.943.708.7 75.113	CYP7 A1		
nucleoside catabolic process (GO:0009164)	0.00479070309 341298	0.0292260289 22216006	0	0	2.594.935.064. 935.060	1.385.975.083. 259.070	UPP2		
nucleoside salvage (GO:0043174)	0.00479070309 341298	0.0292260289 22216006	0	0	2.594.935.064. 935.060	1.385.975.083. 259.070	UPP2		
tyrosine metabolic process (GO:0006570)	0.00479070309 341298	0.0292260289 22216006	0	0	2.594.935.064. 935.060	1.385.975.083. 259.070	TAT		
positive regulation of calcineurin-mediated signaling (GO:0106058)	0.00518902237 9499861	0.0292260289 22216006	0	0	23.785.714.28 5.714.200	1.251.416.370. 420.650	IGF1		
positive regulation of calcineurin-NFAT signaling cascade (GO:0070886)	0.00518902237 9499861	0.0292260289 22216006	0	0	23.785.714.28 5.714.200	1.251.416.370. 420.650	IGF1		
pyrimidine deoxyribonucleotide catabolic process (GO:0009223)	0.00518902237 9499861	0.0292260289 22216006	0	0	23.785.714.28 5.714.200	1.251.416.370. 420.650	UPP2		
2-oxoglutarate metabolic process (GO:0006103)	0.00558720228 37974	0.0292260289 22216006	0	0	21.954.945.05 4.945.000	11.388.637.28 0.375.100	TAT		
pyrimidine nucleoside biosynthetic process (GO:0046134)	0.00558720228 37974	0.0292260289 22216006	0	0	21.954.945.05 4.945.000	11.388.637.28 0.375.100	UPP2		
positive regulation of glycogen biosynthetic process (GO:0045725)	0.00558720228 37974	0.0292260289 22216006	0	0	21.954.945.05 4.945.000	11.388.637.28 0.375.100	IGF1		
positive regulation of glycoprotein biosynthetic process (GO:0010560)	0.00558720228 37974	0.0292260289 22216006	0	0	21.954.945.05 4.945.000	11.388.637.28 0.375.100	IGF1		
negative regulation of neuroinflammatory response (GO:0150079)	0.00558720228 37974	0.0292260289 22216006	0	0	21.954.945.05 4.945.000	11.388.637.28 0.375.100	IGF1		
pyrimidine nucleoside metabolic process (GO:0006213)	0.00598524283 64459065	0.0292260289 22216006	0	0	20.385.714.28 5.714.200	1.043.434.298. 452.160	UPP2		
positive regulation of glycogen metabolic process (GO:0070875)	0.00598524283 64459065	0.0292260289 22216006	0	0	20.385.714.28 5.714.200	1.043.434.298. 452.160	IGF1		
myoblast differentiation (GO:0045445)	0.00598524283 64459065	0.0292260289 22216006	0	0	20.385.714.28 5.714.200	1.043.434.298. 452.160	IGF1		
cellular response to sterol (GO:0036315)	0.00638314407 0851652	0.0292260289 22216006	0	0	19.025.714.28 5.714.200	9.615.775.796. 687.340	CYP7 A1		
aromatic amino acid family catabolic process (GO:0009074)	0.00638314407 0851652	0.0292260289 22216006	0	0	19.025.714.28 5.714.200	9.615.775.796. 687.340	TAT		
negative regulation of release of cytochrome c from mitochondria (GO:0090201)	0.00638314407 0851652	0.0292260289 22216006	0	0	19.025.714.28 5.714.200	9.615.775.796. 687.340	IGF1		
positive regulation of cardiac muscle tissue growth (GO:0055023)	0.00678090602 009761	0.0292260289 22216006	0	0	17.835.714.28 5.714.200	8.906.521.752. 159.380	IGF1		
regulation of steroid biosynthetic process (GO:0050810)	0.00678090602 009761	0.0292260289 22216006	0	0	17.835.714.28 5.714.200	8.906.521.752. 159.380	CYP7 A1		
pyrimidine-containing compound catabolic process (GO:0072529)	0.00678090602 009761	0.0292260289 22216006	0	0	17.835.714.28 5.714.200	8.906.521.752. 159.380	UPP2		
positive regulation of smooth muscle cell migration (GO:0014911)	0.00678090602 009761	0.0292260289 22216006	0	0	17.835.714.28 5.714.200	8.906.521.752. 159.380	IGF1		
positive regulation of muscle hypertrophy (GO:0014742)	0.00678090602 009761	0.0292260289 22216006	0	0	17.835.714.28 5.714.200	8.906.521.752. 159.380	IGF1		
negative regulation of amyloid-beta formation (GO:1902430)	0.00678090602 009761	0.0292260289 22216006	0	0	17.835.714.28 5.714.200	8.906.521.752. 159.380	IGF1		
positive regulation of cardiac muscle hypertrophy (GO:0010613)	0.00717852872 0410204	0.0292260289 22216006	0	0	16.785.714.28 5.714.200	8.286.537.822. 644.390	IGF1		
regulation of multicellular organism growth (GO:0040014)	0.00717852872 0410204	0.0292260289 22216006	0	0	16.785.714.28 5.714.200	8.286.537.822. 644.390	IGF1		

5. Supplementary Table S5 (Proteomic analysis; Overview of all significantly altered pathways in the offspring by maternal diet)

regulation of insulin-like growth factor receptor signaling pathway (GO:0043567)	0.00757601220 63041925	0.0292260289 22216006	0	0	15.852.380.95 2.380.900	7.740.350.338. 275.500	IGF1		
response to cholesterol (GO:0070723)	0.00757601220 63041925	0.0292260289 22216006	0	0	15.852.380.95 2.380.900	7.740.350.338. 275.500	CYP7 A1		
regulation of neuroinflammatory response (GO:0150077)	0.00757601220 63041925	0.0292260289 22216006	0	0	15.852.380.95 2.380.900	7.740.350.338. 275.500	IGF1		
negative regulation of amyloid precursor protein catabolic process (GO:1902992)	0.00757601220 63041925	0.0292260289 22216006	0	0	15.852.380.95 2.380.900	7.740.350.338. 275.500	IGF1		
positive regulation of activated T cell proliferation (GO:0042104)	0.00797335651 42536	0.0292260289 22216006	0	0	15.017.293.23 3.082.700	7.255.830.081. 396.660	IGF1		
regulation of smooth muscle cell migration (GO:0014910)	0.00797335651 42536	0.0292260289 22216006	0	0	15.017.293.23 3.082.700	7.255.830.081. 396.660	IGF1		
positive regulation of purine nucleotide metabolic process (GO:1900544)	0.00837056168 1605555	0.0292260289 22216006	0	0	14.265.714.28 5.714.200	6.823.340.060. 288.630	IGF1		
positive regulation of transcription regulatory region DNA binding (GO:2000679)	0.00837056168 1605555	0.0292260289 22216006	0	0	14.265.714.28 5.714.200	6.823.340.060. 288.630	IGF1		
positive regulation of glycolytic process (GO:0045821)	0.00837056168 1605555	0.0292260289 22216006	0	0	14.265.714.28 5.714.200	6.823.340.060. 288.630	IGF1		
positive regulation of vascular associated smooth muscle cell proliferation (GO:1904707)	0.00837056168 1605555	0.0292260289 22216006	0	0	14.265.714.28 5.714.200	6.823.340.060. 288.630	IGF1		
myotube differentiation (GO:0014902)	0.00837056168 1605555	0.0292260289 22216006	0	0	14.265.714.28 5.714.200	6.823.340.060. 288.630	IGF1		
negative regulation of reproductive process (GO:2000242)	0.00837056168 1605555	0.0292260289 22216006	0	0	14.265.714.28 5.714.200	6.823.340.060. 288.630	IGF1		
glutamate metabolic process (GO:0006536)	0.00876762774 5742801	0.0296324087 27675856	0	0	13.585.714.28 5.714.200	6.435.130.349. 149.830	TAT		
ERK1 and ERK2 cascade (GO:0070371)	0.00916455474 5730224	0.0296324087 27675856	0	0	12.967.532.46 7.532.400	6.084.900.471. 331.720	IGF1		
positive regulation of carbohydrate metabolic process (GO:0045913)	0.00956134271 7827156	0.0296324087 27675856	0	0	12.403.105.59 0.062.100	5.767.477.724. 354.010	IGF1		
regulation of cardiac muscle hypertrophy (GO:0010611)	0.00956134271 7827156	0.0296324087 27675856	0	0	12.403.105.59 0.062.100	5.767.477.724. 354.010	IGF1		
protein kinase B signaling (GO:0043491)	0.00956134271 7827156	0.0296324087 27675856	0	0	12.403.105.59 0.062.100	5.767.477.724. 354.010	IGF1		
cellular response to hexose stimulus (GO:0071331)	0.00995799170 1556103	0.0296324087 27675856	0	0	11.885.714.28 5.714.200	5.478.577.209. 909.950	CYP7 A1		
regulation of glycogen biosynthetic process (GO:0005979)	0.01035450173 6256563	0.0296324087 27675856	0	0	11.409.714.28 5.714.200	5.214.620.402. 892.070	IGF1		
proteoglycan metabolic process (GO:0006029)	0.01035450173 6256563	0.0296324087 27675856	0	0	11.409.714.28 5.714.200	5.214.620.402. 892.070	IGF1		
negative regulation of cell development (GO:0010721)	0.01035450173 6256563	0.0296324087 27675856	0	0	11.409.714.28 5.714.200	5.214.620.402. 892.070	IGF1		
negative regulation of interleukin-1 beta production (GO:0032691)	0.01035450173 6256563	0.0296324087 27675856	0	0	11.409.714.28 5.714.200	5.214.620.402. 892.070	IGF1		
positive regulation of glucose import (GO:0046326)	0.01035450173 6256563	0.0296324087 27675856	0	0	11.409.714.28 5.714.200	5.214.620.402. 892.070	IGF1		
positive regulation of nuclear division (GO:0051785)	0.01075087286 0949598	0.0296324087 27675856	0	0	1.097.032.967. 032.960	49.725.962.91 4.000.300	IGF1		
regulation of transcription regulatory region DNA binding (GO:2000677)	0.01075087286 0949598	0.0296324087 27675856	0	0	1.097.032.967. 032.960	49.725.962.91 4.000.300	IGF1		
positive regulation of striated muscle cell differentiation (GO:0051155)	0.01075087286 0949598	0.0296324087 27675856	0	0	1.097.032.967. 032.960	49.725.962.91 4.000.300	IGF1		
bile acid biosynthetic process (GO:0006699)	0.01075087286 0949598	0.0296324087 27675856	0	0	1.097.032.967. 032.960	49.725.962.91 4.000.300	CYP7 A1		
regulation of calcineurin-NFAT signaling cascade (GO:0070884)	0.01114710511 3416074	0.0296324087 27675856	0	0	10.563.492.06 3.492.000	4.749.953.903. 533.840	IGF1		
positive regulation of fibroblast proliferation (GO:0048146)	0.01114710511 3416074	0.0296324087 27675856	0	0	10.563.492.06 3.492.000	4.749.953.903. 533.840	IGF1		
alpha-amino acid catabolic process (GO:1901606)	0.01193915316 674268	0.0296324087 27675856	0	0	9.833.990.147. 783.250	435.442.406.2 27.662	TAT		
positive regulation of small GTPase mediated signal transduction (GO:0051057)	0.01193915316 674268	0.0296324087 27675856	0	0	9.833.990.147. 783.250	435.442.406.2 27.662	IGF1		
negative regulation of interleukin-1 production (GO:0032692)	0.01193915316 674268	0.0296324087 27675856	0	0	9.833.990.147. 783.250	435.442.406.2 27.662	IGF1		
activation of protein kinase B activity (GO:0032148)	0.01233496904 2911155	0.0296324087 27675856	0	0	9.505.714.285. 714.280	4.178.062.796. 500.490	IGF1		
proteoglycan biosynthetic process (GO:0030166)	0.01233496904 2911155	0.0296324087 27675856	0	0	9.505.714.285. 714.280	4.178.062.796. 500.490	IGF1		
regulation of developmental growth (GO:0048638)	0.01233496904 2911155	0.0296324087 27675856	0	0	9.505.714.285. 714.280	4.178.062.796. 500.490	IGF1		
cellular response to glucose stimulus (GO:0071333)	0.01273064620 9021314	0.0296324087 27675856	0	0	9.198.617.511. 520.730	4.014.040.374. 155.010	CYP7 A1		
positive regulation of glucose transmembrane transport (GO:0010828)	0.01273064620 9021314	0.0296324087 27675856	0	0	9.198.617.511. 520.730	4.014.040.374. 155.010	IGF1		
regulation of cellular biosynthetic process (GO:0031326)	0.01273064620 9021314	0.0296324087 27675856	0	0	9.198.617.511. 520.730	4.014.040.374. 155.010	CYP7 A1		
positive regulation of ATP metabolic process (GO:1903580)	0.01312618470 2817524	0.0296324087 27675856	0	0	8.910.714.285. 714.280	3.861.142.784. 895.610	IGF1		
bile acid metabolic process (GO:0008206)	0.01312618470 2817524	0.0296324087 27675856	0	0	8.910.714.285. 714.280	3.861.142.784. 895.610	CYP7 A1		
primary alcohol metabolic process (GO:0034308)	0.01312618470 2817524	0.0296324087 27675856	0	0	8.910.714.285. 714.280	3.861.142.784. 895.610	IGF1		
muscle cell development (GO:0055001)	0.01312618470 2817524	0.0296324087 27675856	0	0	8.910.714.285. 714.280	3.861.142.784. 895.610	IGF1		
regulation of activated T cell proliferation (GO:0046006)	0.01352158456 5055974	0.0296324087 27675856	0	0	8.640.259.740. 259.740	3.718.308.142. 207.310	IGF1		
regulation of amyloid-beta formation (GO:1902003)	0.01352158456 5055974	0.0296324087 27675856	0	0	8.640.259.740. 259.740	3.718.308.142. 207.310	IGF1		
positive regulation of DNA binding (GO:0043388)	0.01352158456 5055974	0.0296324087 27675856	0	0	8.640.259.740. 259.740	3.718.308.142. 207.310	IGF1		

5. Supplementary Table S5 (Proteomic analysis; Overview of all significantly altered pathways in the offspring by maternal diet)

negative regulation of mitochondrion organization (GO:0010823)	0.013521584565055974	0.029632408727675856	0	0	8.640.259.740.259.740	3.718.308.142.207.310	IGF1		
phosphatidylinositol 3-kinase signaling (GO:0014065)	0.013521584565055974	0.029632408727675856	0	0	8.640.259.740.259.740	3.718.308.142.207.310	IGF1		
cellular response to amyloid-beta (GO:1904646)	0.013916845835577172	0.030177581496093658	0	0	8.385.714.285.714.280	3.584.603.752.954.010	IGF1		
positive regulation of mitotic nuclear division (GO:0045840)	0.014311968554805366	0.0302963226906595	0	0	8.145.714.285.714.280	34.592.071.940.977.700	IGF1		
neutrophil degranulation (GO:0043312)	0.014681417748872256	0.0302963226906595	0	0	13.578.983.994.432.800	57.319.236.307.803.200			
regulation of glucose import (GO:0046324)	0.014706952762456068	0.0302963226906595	0	0	7.919.047.619.047.610	3.341.390.605.677.810	IGF1		
regulation of vascular associated smooth muscle cell proliferation (GO:1904705)	0.014706952762456068	0.0302963226906595	0	0	7.919.047.619.047.610	3.341.390.605.677.810	IGF1		
glutamine family amino acid metabolic process (GO:0009064)	0.014706952762456068	0.0302963226906595	0	0	7.919.047.619.047.610	3.341.390.605.677.810	TAT		
neutrophil activation involved in immune response (GO:0002283)	0.014914864833288563	0.03042041738274697	0	0	1.346.376.811.594.200	5.662.048.901.383.080			
neutrophil mediated immunity (GO:0002446)	0.015091030566676898	0.030477963693484714	0	0	13.378.600.823.045.200	56.105.232.406.378.800			
regulation of release of cytochrome c from mitochondria (GO:0090199)	0.01628550529596983	0.03257101059193966	0	0	7.125.714.285.714.280	293.399.847.205.648	IGF1		
cellular glucose homeostasis (GO:0001678)	0.017073951550447674	0.033819557878771354	0	0	6.785.714.285.714.280	2.761.922.295.799.090	CYP7A1		
positive regulation of osteoblast differentiation (GO:0045669)	0.01746796731842876	0.033947181769776645	0	0	6.627.574.750.830.560	2.682.435.666.902.730	IGF1		
response to amyloid-beta (GO:1904645)	0.01746796731842876	0.033947181769776645	0	0	6.627.574.750.830.560	2.682.435.666.902.730	IGF1		
regulation of fibroblast proliferation (GO:0048145)	0.018255584335152574	0.03450137956918743	0	0	6.332.380.952.380.950	25.350.320.962.011.800	IGF1		
negative regulation of tumor necrosis factor production (GO:0032720)	0.018255584335152574	0.03450137956918743	0	0	6.332.380.952.380.950	25.350.320.962.011.800	IGF1		
positive regulation of smooth muscle cell proliferation (GO:0048661)	0.018255584335152574	0.03450137956918743	0	0	6.332.380.952.380.950	25.350.320.962.011.800	IGF1		
regulation of DNA binding (GO:0051101)	0.01864918566892	0.034924838616341096	0	0	6.194.409.937.888.190	2.466.584.798.233.950	IGF1		
negative regulation of tumor necrosis factor superfamily cytokine production (GO:1903556)	0.019042648937520508	0.03502487215293951	0	0	6.062.310.030.395.130	24.013.259.456.270.100	IGF1		
cellular response to alcohol (GO:0097306)	0.019042648937520508	0.03502487215293951	0	0	6.062.310.030.395.130	24.013.259.456.270.100	CYP7A1		
regulation of smooth muscle cell proliferation (GO:0048660)	0.01943597418461227	0.035121146333597614	0	0	59.357.142.857.142.800	2.339.045.135.601.690	IGF1		
response to glucose (GO:0009749)	0.01943597418461227	0.035121146333597614	0	0	59.357.142.857.142.800	2.339.045.135.601.690	CYP7A1		
organic hydroxy compound biosynthetic process (GO:1901617)	0.01982916145118511	0.03552006312125333	0	0	58.142.857.142.857.100	2.279.549.801.512.960	CYP7A1		
inositol lipid-mediated signaling (GO:0048017)	0.020222210774875234	0.035911857065726714	0	0	5.697.714.285.714.280	22.226.633.779.925.100	IGF1		
cellular response to peptide (GO:1901653)	0.022577612257970867	0.03908393382472268	0	0	50.857.142.857.142.800	19.278.907.770.536.500	IGF1		
regulation of mitotic nuclear division (GO:0007088)	0.022577612257970867	0.03908393382472268	0	0	50.857.142.857.142.800	19.278.907.770.536.500	IGF1		
dicarboxylic acid metabolic process (GO:0043648)	0.022577612257970867	0.03908393382472268	0	0	50.857.142.857.142.800	19.278.907.770.536.500	TAT		
muscle organ development (GO:0007517)	0.0229696971452446	0.03943131343266989	0	0	49.962.406.015.037.500	18.853.710.846.919.200	IGF1		
positive regulation of tyrosine phosphorylation of STAT protein (GO:0042531)	0.023361644422266112	0.03977271695030429	0	0	4.909.852.216.748.760	18.444.644.096.582.100	IGF1		
positive regulation of cellular process (GO:0048522)	0.024091055108044237	0.040678338952927155	0	0	10.363.295.880.149.800	3.861.275.609.472.420			
positive regulation of Ras protein signal transduction (GO:0046579)	0.02492805820599394	0.04136072060304657	0	0	4.592.165.898.617.510	1.695.318.022.982.440	IGF1		
monocarboxylic acid biosynthetic process (GO:0072330)	0.02492805820599394	0.04136072060304657	0	0	4.592.165.898.617.510	1.695.318.022.982.440	CYP7A1		
regulation of extrinsic apoptotic signaling pathway (GO:2001236)	0.025319318033677134	0.04136072060304657	0	0	4.519.047.619.047.610	16.612.866.893.112.000	IGF1		
regulation of cellular ketone metabolic process (GO:0010565)	0.025319318033677134	0.04136072060304657	0	0	4.519.047.619.047.610	16.612.866.893.112.000	CYP7A1		
steroid biosynthetic process (GO:0006694)	0.025710440490540786	0.04136072060304657	0	0	44.482.142.857.142.800	16.284.281.408.743.700	CYP7A1		
extracellular matrix disassembly (GO:0022617)	0.026101425623281816	0.04136072060304657	0	0	4.379.560.439.560.430	15.966.849.674.970.800	PRSS2		
positive regulation of T cell proliferation (GO:0042102)	0.026101425623281816	0.04136072060304657	0	0	4.379.560.439.560.430	15.966.849.674.970.800	IGF1		
cellular component disassembly (GO:0022411)	0.026101425623281816	0.04136072060304657	0	0	4.379.560.439.560.430	15.966.849.674.970.800	PRSS2		
regulation of tyrosine phosphorylation of STAT protein (GO:0042509)	0.026882984076400464	0.04185398360412257	0	0	4.248.400.852.878.460	15.363.329.523.076.900	IGF1		
positive regulation of protein transport (GO:0051222)	0.027273557473830964	0.04185398360412257	0	0	41.857.142.857.142.800	1.507.626.325.151.400	IGF1		
sterol metabolic process (GO:0016125)	0.02766399371512237	0.04185398360412257	0	0	4.124.844.720.496.890	1.479.839.017.165.650	CYP7A1		
wound healing (GO:0042060)	0.02766399371512237	0.04185398360412257	0	0	4.124.844.720.496.890	1.479.839.017.165.650	IGF1		
regulation of glycolytic process (GO:0006110)	0.028054292838003377	0.04185398360412257	0	0	4.065.714.285.714.280	14.529.291.931.699.200	IGF1		
cholesterol homeostasis (GO:0042632)	0.028054292838003377	0.04185398360412257	0	0	4.065.714.285.714.280	14.529.291.931.699.200	CYP7A1		
phosphatidylinositol-mediated signaling (GO:0048015)	0.028054292838003377	0.04185398360412257	0	0	4.065.714.285.714.280	14.529.291.931.699.200	IGF1		

5. Supplementary Table S5 (Proteomic analysis; Overview of all significantly altered pathways in the offspring by maternal diet)

sterol homeostasis (GO:0055092)	0.02844445487658815	0.04185398360412257	0	0	4.008.249.496.981.890	14.268.574.756.562.200	CYP7A1		
positive regulation of protein secretion (GO:0050714)	0.02844445487658815	0.04185398360412257	0	0	4.008.249.496.981.890	14.268.574.756.562.200	IGF1		
positive regulation of secretion by cell (GO:1903532)	0.02844445487658815	0.04185398360412257	0	0	4.008.249.496.981.890	14.268.574.756.562.200	IGF1		
positive regulation of growth (GO:0045927)	0.030003733059445183	0.043783226045979264	0	0	3.793.714.285.714.280	1.330.240.674.608.420	PRSS2		
positive regulation of phosphatidylinositol 3-kinase signaling (GO:0014068)	0.03039321031347104	0.043783226045979264	0	0	3.743.609.022.556.390	13.078.433.041.717.300	IGF1		
cholesterol metabolic process (GO:0008203)	0.03039321031347104	0.043783226045979264	0	0	3.743.609.022.556.390	13.078.433.041.717.300	CYP7A1		
negative regulation of apoptotic signaling pathway (GO:2001234)	0.030782550739377516	0.04403614897438728	0	0	3.694.805.194.805.190	1.286.090.483.496.380	IGF1		
positive regulation of cell adhesion (GO:0045785)	0.0315608212516243	0.044531021765990456	0	0	3.600.904.159.132.000	12.444.144.169.317.200	PRSS2		
negative regulation of extrinsic apoptotic signaling pathway (GO:2001237)	0.0315608212516243	0.044531021765990456	0	0	3.600.904.159.132.000	12.444.144.169.317.200	IGF1		
regulation of interleukin-1 beta production (GO:0032651)	0.032727201789121446	0.04524700381583233	0	0	3.468.641.114.982.570	118.611.871.734.907	IGF1		
glycoprotein biosynthetic process (GO:0009101)	0.032727201789121446	0.04524700381583233	0	0	3.468.641.114.982.570	118.611.871.734.907	IGF1		
regulation of osteoblast differentiation (GO:0045667)	0.032727201789121446	0.04524700381583233	0	0	3.468.641.114.982.570	118.611.871.734.907	IGF1		
regulation of Ras protein signal transduction (GO:0046578)	0.03389235304552857	0.04623724985019129	0	0	33.457.142.857.142.800	11.323.790.361.856.500	IGF1		
negative regulation of cellular amide metabolic process (GO:0034249)	0.03389235304552857	0.04623724985019129	0	0	33.457.142.857.142.800	11.323.790.361.856.500	IGF1		
positive regulation of binding (GO:0051099)	0.03544397773863309	0.04803591719841063	0	0	31.947.030.497.592.200	1.066.967.538.219.120	IGF1		
regulation of peptidyl-tyrosine phosphorylation (GO:0050730)	0.036218972118804385	0.0487654134410046	0	0	3.124.175.824.175.820	10.366.553.385.683.800	IGF1		
regulation of epithelial cell proliferation (GO:0050678)	0.03660626495698383	0.048966821955445904	0	0	30.900.621.118.012.400	1.022.049.130.477.510	IGF1		
positive regulation of cell cycle process (GO:0090068)	0.039699708710484755	0.052762193511999095	0	0	28.417.142.857.142.800	9.168.539.448.196.990	IGF1		
steroid metabolic process (GO:0008202)	0.04085750745521912	0.05395286240881499	0	0	2.758.529.819.694.860	8.820.853.410.483.190	CYP7A1		
small GTPase mediated signal transduction (GO:0007264)	0.041628694578224525	0.05427538660198894	0	0	27.057.142.857.142.800	8.601.372.572.734.710	IGF1		
regulation of phosphatidylinositol 3-kinase signaling (GO:0014066)	0.041628694578224525	0.05427538660198894	0	0	27.057.142.857.142.800	8.601.372.572.734.710	IGF1		
activation of protein kinase activity (GO:0032147)	0.04470802045537426	0.05792359882897545	0	0	25.131.479.140.328.600	7.809.864.401.057.700	IGF1		
positive regulation of epithelial cell proliferation (GO:0050679)	0.048161911106035224	0.06183533193575454	0	0	2.326.697.892.271.660	705.730.932.589.798	IGF1		
regulation of tumor necrosis factor production (GO:0032680)	0.048545001420476706	0.06183533193575454	0	0	23.076.655.052.264.700	6.981.297.485.230.680	IGF1		
platelet degranulation (GO:0002576)	0.0489279568229514	0.06183533193575454	0	0	22.889.400.921.658.900	690.666.232.697.606	IGF1		
regulation of protein secretion (GO:0050708)	0.0489279568229514	0.06183533193575454	0	0	22.889.400.921.658.900	690.666.232.697.606	IGF1		
positive regulation of macromolecule biosynthetic process (GO:0010557)	0.050458430115721514	0.06338071099901604	0	0	22.169.642.857.142.800	6.621.197.613.100.180	IGF1		
regulation of cell adhesion (GO:0030155)	0.05198674804787824	0.06490466725977526	0	0	21.493.506.493.506.400	6.355.127.863.735.060	PRSS2		
positive regulation of peptidyl-tyrosine phosphorylation (GO:0050731)	0.052368491061118044	0.06498740456982119	0	0	21.330.827.067.669.100	6.291.421.182.271.060	IGF1		
positive regulation of cellular catabolic process (GO:0031331)	0.055036928219595174	0.06788986355231501	0	0	20.257.142.857.142.800	5.874.066.816.819.040	IGF1		
Ras protein signal transduction (GO:0007265)	0.055417596312302815	0.06795252881151417	0	0	20.112.462.006.079.000	5.818.249.888.054.840	IGF1		
monocarboxylic acid metabolic process (GO:0032787)	0.05579813022483266	0.06801428891310964	0	0	19.969.818.913.480.800	5.763.319.530.748.080	IGF1		
skeletal system development (GO:0001501)	0.06149006445754368	0.07451148987208234	0	0	18.048.225.659.690.600	50.334.329.635.540.900	IGF1		
regulation of MAPK cascade (GO:0043408)	0.06451346874924052	0.07771797989674589	0	0	17.166.233.766.233.700	47.050.608.420.266.300	IGF1		
protein stabilization (GO:0050821)	0.06940832625547197	0.08261774885366903	0	0	15.902.086.677.367.500	42.422.766.986.769.000	IGF1		
positive regulation of cellular biosynthetic process (GO:0031328)	0.06978392378902142	0.08261774885366903	0	0	1.581.245.011.971.260	420.983.020.967.207	IGF1		
regulated exocytosis (GO:0045055)	0.06978392378902142	0.08261774885366903	0	0	1.581.245.011.971.260	420.983.020.967.207	IGF1		
negative regulation of macromolecule metabolic process (GO:0010605)	0.07502837931017566	0.0883191207879782	0	0	14.655.070.318.282.700	37.955.003.157.820.300	IGF1		
regulation of macromolecule metabolic process (GO:0060255)	0.07726807133802915	0.0904387653161023	0	0	14.208.901.651.112.700	3.638.152.974.515.550	IGF1		
negative regulation of inflammatory response (GO:0050728)	0.08173322422814254	0.0951245434519625	0	0	1.339.268.788.083.950	33.539.237.258.401.200	IGF1		
regulation of cell growth (GO:0001558)	0.08358811737340943	0.09673680999394574	0	0	13.079.365.079.365.000	3.246.107.330.597.870	PRSS2		
positive regulation of cell motility (GO:2000147)	0.08506967055696874	0.09790140857394167	0	0	12.838.961.038.961.000	31.638.855.311.433.900	IGF1		
positive regulation of signal transduction (GO:0009967)	0.09648082675964229	0.11022348182860886	0	0	11.235.628.912.919.700	26.273.517.981.296.400	IGF1		
positive regulation of phosphorylation (GO:0042327)	0.09684684568436021	0.11022348182860886	0	0	1.119.047.619.047.610	26.125.559.419.255.800	IGF1		
positive regulation of cell differentiation (GO:0045597)	0.09867499486063239	0.11168708209500149	0	0	10.969.983.324.068.900	25.405.644.473.217.400	IGF1		
positive regulation of cell migration (GO:0030335)	0.10268552958497451	0.1155913611721571	0	0	10.513.859.275.053.300	23.930.427.629.401.800	IGF1		

5. Supplementary Table S5 (Proteomic analysis; Overview of all significantly altered pathways in the offspring by maternal diet)

positive regulation of MAPK cascade (GO:0043410)	0.1045033320824488	0.11699829570100247	0	0	1.031.868.131.868.130	2.330.511.655.471.740	IGF1		
proteolysis (GO:0006508)	0.10921454706437869	0.1216118740284433	0	0	9.843.156.843.156.840	21.797.090.178.084.400	PRSS2		
extracellular matrix organization (GO:0030198)	0.1139040603715798	0.1261518087986314	0	0	94.089.823.220.258	204.400.615.348.683	PRSS2		
MAPK cascade (GO:0000165)	0.1149831829216247	0.12666596621312667	0	0	9.314.096.499.526.960	2.014.610.568.709.960	IGF1		
negative regulation of gene expression (GO:0010629)	0.12179096137390887	0.133451798101198	0	0	87.543.391.188.251	1.843.181.572.708.940	IGF1		
positive regulation of protein phosphorylation (GO:0001934)	0.13913684107142335	0.15165179503022863	0	0	7.576.061.776.061.770	14.942.246.655.144.300	IGF1		
negative regulation of programmed cell death (GO:0043069)	0.14263974603941806	0.15465151412694803	0	0	7.372.932.330.827.060	1.435.829.236.085.330	IGF1		
positive regulation of macromolecule metabolic process (GO:0010604)	0.14368818255703167	0.15497259479973047	0	0	7.314.061.917.195.070	14.190.082.663.446.400	IGF1		
transmembrane receptor protein tyrosine kinase signaling pathway (GO:0007169)	0.15064913556710083	0.16163396836886862	0	0	6.943.991.492.378.580	13.143.599.258.783.100	IGF1		
regulation of cell migration (GO:0030334)	0.15203536892875155	0.16227609326074	0	0	6.874.341.874.341.870	12.948.799.729.129.400	IGF1		
cellular protein metabolic process (GO:0044267)	0.15514715809285315	0.16474388952127705	0	0	6.722.527.472.527.470	12.526.631.345.187.200	IGF1		
positive regulation of cell population proliferation (GO:0008284)	0.17462414847531177	0.1844747414662268	0	0	5.895.197.825.430.380	10.287.823.724.852.200	IGF1		
positive regulation of gene expression (GO:0010628)	0.17732607109808496	0.18637331962349746	0	0	5.794.772.794.772.790	1.002.359.534.647.410	IGF1		
negative regulation of apoptotic process (GO:0043066)	0.17833729496299572	0.1864846840729803	0	0	5.757.969.303.423.840	9.927.191.691.507.290	IGF1		
positive regulation of nucleic acid-templated transcription (GO:1903508)	0.1870557763520291	0.19461358549756563	0	0	5.457.142.857.142.850	9.148.072.900.906.000	IGF1		
positive regulation of intracellular signal transduction (GO:1902533)	0.198664316346586	0.20565250837887797	0	0	5.097.509.829.619.920	8.238.283.067.939.570	IGF1		
regulation of apoptotic process (GO:0042981)	0.2610308087883377	0.2688617330519878	0	0	3.711.393.869.288.600	4.984.835.595.660.660	IGF1		
regulation of cell population proliferation (GO:0042127)	0.2677585596699408	0.2744192203582478	0	0	3.600.262.123.197.900	4.743.955.956.365.440	IGF1		
positive regulation of transcription by RNA polymerase II (GO:0045944)	0.31048641241381475	0.31663465820418735	0	0	3.005.985.194.518.820	35.158.457.908.428.100	IGF1		
regulation of gene expression (GO:0010468)	0.3583792552790436	0.36367550043095065	0	0	25.064.935.064.935.000	2.572.072.101.808.580	IGF1		
positive regulation of transcription, DNA-templated (GO:0045893)	0.3860609366047682	0.38984584774795217	0	0	22.733.865.119.651.900	21.637.184.719.489.500	IGF1		
regulation of transcription by RNA polymerase II (GO:0006357)	0.6074688551572656	0.6104321178653498	0	0	11.523.809.523.809.500	0.5744093242839072	IGF1		
regulation of transcription, DNA-templated (GO:0006355)	0.6141263889203222	0.6141263889203222	0	0	11.304.375.517.482.900	0.55114994569996	IGF1		
